# Enantioselective Synthesis of Spirolactones and Spirolactams Under Low Catalyst Loadings

**DOI:** 10.1021/jacs.6c01407

**Published:** 2026-03-05

**Authors:** Takeru Saito, Antonio Navarro, Huw M. L. Davies

**Affiliations:** † Department of Chemistry, 1371 Emory University, Atlanta, Georgia 30322, United States; ‡ Lilly Research Laboratories, Eli Lilly and Company, Indianapolis, Indiana 46285, United States

## Abstract

The stereoselective construction of spirocyclic compounds as medicinally relevant scaffolds is of considerable current interest. One underexplored area in this field is the asymmetric synthesis of spirocyclopropanes via transition-metal catalysis, using α-diazo lactones and lactams as carbene precursors. In this study, we report a Rh_2_(*S*-*p*-PhTPCP)_4_-catalyzed [2 + 1] cyclopropanation between 3-diazo δ-lactones and lactams and a wide range of alkene substrates, enabling the facile synthesis of 5-oxa and 5-azaspiro[2.5]­octanones in yields up to 98%, reasonable levels of diastereoselectivity (up to 11:1 d.r.), and high levels of enantioselectivity (up to 98% *ee*). The reaction can be run with 0.00005 mol % of catalyst, resulting in 1,740,000 TON, while maintaining stereocontrol. The ring size of the diazo compound, in conjunction with the reaction temperature, has a considerable influence on the reaction. While the reaction with the 5-membered system gave diminished stereoselectivity, the 7-membered counterpart gave enhanced stereocontrol (>30:1 d.r., 99% *ee*), but the reaction needed to be carried out at low temperatures to avoid a side-reaction caused by a 1,2-hydride shift.

## Graphical abstract

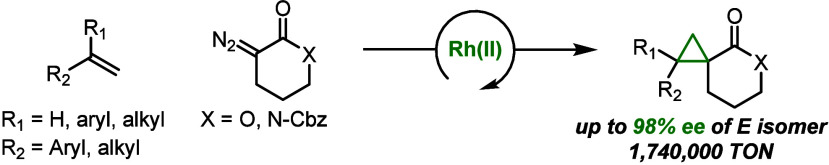

## Introduction

Spirocyclic compounds have recently been a focus of attention in the synthetic community as medicinally relevant scaffolds.^1^ These conformationally defined substructures offer a direct trajectory into deep binding pockets, leveraging their rigid, sp^3^-rich frameworks to enhance target engagement and physiochemical properties.^1,2^ One system of considerable interest has been spirocycles where one of the rings is a cyclopropane (Figure 1A). The indolinone substructure (**1**) has been extensively incorporated into drug candidates.^3^ While general methods are available for the construction of spirocyclopropanes containing substructures related to **2** and **3**,^4^ their enantioselective synthesis has not been extensively explored.^5^

**Figure 1:**
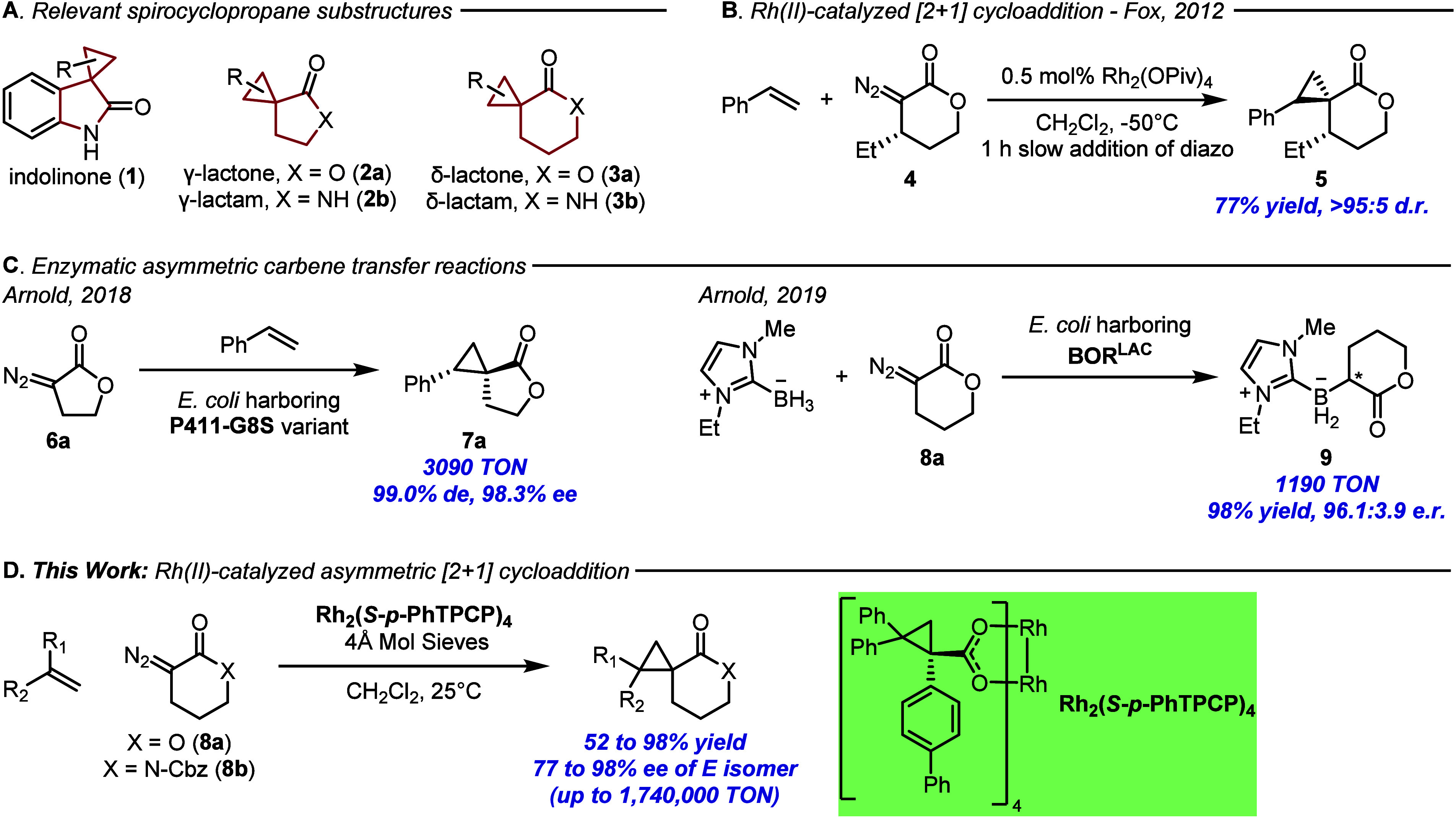
Prior and Current Work in Asymmetric Carbene Transfer Reactions with Diazo Lactones and Lactams.

An attractive method would be cyclopropanation using α-diazo lactones or lactams as carbene precursors. While there have been a few studies demonstrating the feasibility of this approach,^6^ the carbene is often prone to a 1,2-hydride shift (β-hydride elimination) to afford the α,β-unsaturated enone as a major side-product.^6c^ Fox, however, demonstrated that careful selection of reaction conditions in the cyclopropanation with the α-diazo-δ-lactone **4** could effectively control the diastereoselective formation of the spirocycle **5** over the elimination product (Figure 1B).^6c^

To date, the only effective enantioselective entry to compounds related to **2** and **3** using cyclopropanation with a 3-diazo γ-lactone or lactam has been achieved using enzymatic processes. The 5-membered γ-lactone **6a** was employed by Arnold and co-workers in an enzymatic cyclopropanation reaction with styrene-type substrates to form spirocycle **7** with high enantioselectivity (Figure 1C).^7^ The 6-membered 3-diazo δ-lactone **8a** was also employed by Arnold, in this case in an enzymatic B–H insertion reaction to form **9** (Figure 1C), but the study was not extended to enantioselective cyclopropanation.^8^ As far as we are aware, the corresponding diazo lactams to **6a** and **8a** have not been reported as substrates for enantioselective cyclopropanations, although diazo compounds derived from γ-lactams^9^ and δ-lactams^10^ have been used in the synthesis of various racemic compounds, typically by means of X–H insertion.

We have generated several chiral dirhodium catalysts capable of high levels of enantioselectivity for a range of reactions with donor/acceptor carbenes, including cyclopropanation, C–H functionalization, and various ylide generation followed by subsequent rearrangements.^11^ The combination of the donor and acceptor groups around the carbene has been shown to be highly favorable for high asymmetric induction with these catalysts. Their extension to other types of carbene intermediates has been less extensive.^12^ Therefore, the enantioselective cyclopropanation using α-diazo lactones and lactams is an interesting challenge for our catalysts (Figure 1D), leading to the ready access of enantioselective chiral building blocks for pharmaceutical applications.

These α-diazo lactones and lactams do not contain the typical aryl or vinyl functionality characteristic of the donor group in donor/acceptor carbenes. Therefore, this study would represent an expanded range to the enantioselective reaction capacity of the dirhodium tetracarboxylate catalysts.

## Results and Discussion

Initial evaluation of the catalysts began with the γ-lactone **6a**, previously utilized in Arnold’s study.^7^ The standard reaction was the cyclopropanation of styrene with 0.5 mol % of catalyst in dichloromethane at 25 °C (Table 1). The cyclopropanation to form **7a** proceeded in reasonable yield with a range of dirhodium catalysts without any evidence of the competing 1,2-hydride migration of the carbene (entries 1–7). Synthetically, with fast addition of **7a**, we observed a significant amount of carbene dimerization, thus we elected to run the reaction with a 3 h slow addition of the diazo compound **6a** to minimize this side reaction. Even though a range of our optimum chiral dirhodium catalysts (see SI page S5, S6 for the structure of the catalysts) were examined, overall, the reaction was not very effective, resulting in low diastereoselectivity (1.4–2.5:1 d.r. (*E*:*Z*)) and enantioselectivity (5–42% *ee*). The best result was obtained with the triarylcyclopropane carboxylate catalyst, Rh_2_(*S*-*p*-PhTPCP)_4_ (entry 7),^13^ which in the reaction with aryldiazoacetates is capable of extremely high levels of enantioselectivity.^14^ Subtle improvements were observed when exchanging the solvent to 1,2-dichloroethane (see SI Figure S1). We then explored the Rh_2_(*S*-*p*-PhTPCP)_4_-catalyzed reaction with the corresponding α-diazo γ-lactam, protected with either N-Cbz **(6b**) or N-Boc (**6c**) (entries 8 and 9) (see SI Figures S2, S6–S8 for details). Both gave poor diastereocontrol (1.2–1.3:1 d.r.) but the N-Cbz cyclopropane **7b** had a slight improvement in enantiocontrol (52% *ee*).

**Table 1:**
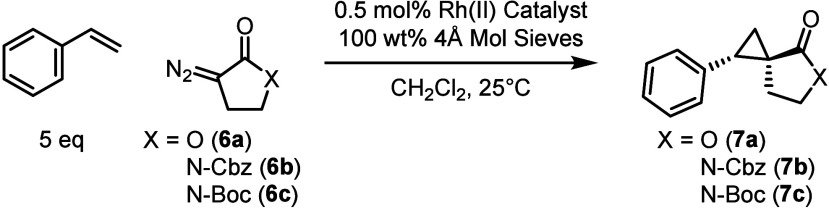
Styrene Cyclopropanation Catalyst Screen with Diazo **6a**–**6c**

**Entry**	**X** ^a^	**Rh(II) Catalyst**	**Yield (%)** ^b^	**d.r. (** ***E*** **:** ***Z*** **)**	***ee*** **(%)** ^c^
1	O	Rh_2_(*S*-DOSP)_4_	77	2:1	5
2	O	Rh_2_(*S*-TPPTTL)_4_	53	1.3:1	18
3	O	Rh_2_(*S*-NTTL)_4_	43	2.5:1	–10
4	O	Rh_2_(*S*-TCPTAD)_4_	67	1.7:1	–2
5	O	Rh_2_(*S*-2-Cl,5-BrTPCP)_4_	77	1.4:1	–8
6	O	Rh_2_(*S*-*p*-BrTPCP)_4_	58	1.5:1	40
7	O	Rh_2_(*S*-*p*-PhTPCP)_4_	69	2:1	42
8	N-Cbz	Rh_2_(*S*-*p*-PhTPCP)_4_	51	1.3:1	52
9	N-Boc	Rh_2_(*S*-*p*-PhTPCP)_4_	50	1.2:1	34

a 3 h slow addition of diazo when X = O, fast diazo addition when X = N-PG.

b Yield reported as a mixture of diastereomers.

c Enantioselectivity of *E*-diastereomer.

The next series of experiments focused on cyclopropanation with the α-diazo δ-lactone **8a**. The carbene derived from **8a** was anticipated to be more conformationally defined than that derived from γ-lactone **6a** and therefore, it was conceivable that this system would see a more significant influence by the chiral catalysts. A series of catalysts were examined using the same standard conditions that were utilized with **6a**, and the results are summarized in Table 2. All catalyst families examined gave better diastereoselectivity with this system, generating the spirocycle **10a** in 4–11:1 d.r. (*E*:*Z*, entries 1–8). Once more, Rh_2_(*S*-*p*-PhTPCP)_4_ appeared to be the optimal catalyst, affording the *E*-diastereomer in 7:1 d.r. (*E*:*Z*) and 90% *ee* (entry 7). Further optimization by conducting the reaction at −78 °C, generated **10a** in an enhanced 11:1 d.r. (*E*:*Z*) and 94% *ee* (entry 8). Other members of the triarylcyclopropane carboxylate family of catalysts were examined (entries 5–6), but they all showed diminished stereoselectivity. In this system, we saw that the reactions in more polar solvents such as dichloromethane, dichloroethane, and dimethyl carbonate had similar effects on enantioselectivity (see SI Figure 3). For the α-diazo δ-lactam **8b**, we elected to protect the nitrogen with an N-Cbz group, as this gave higher enantioselectivity over the N-Boc in the γ-lactam reaction (Table 2). We introduced the diazo compound quickly to the reaction mixture (dropwise over 1 min), which did not show any deleterious side reactions. The reaction with Rh_2_(*S*-*p*-PhTPCP)_4_ at 25 °C generated **10b** in 8:1 d.r. (*E*:*Z*) and 96% *ee* (entry 9) and we thus decided to use this catalyst in further reactions with the lactam **8b**. Running the reaction in dichloromethane at cryogenic temperatures maintained the 8:1 d.r. (*E*:*Z)* and improved enantioselectivity to 98% *ee* with 75% yield (entry 10).

**Table 2:**
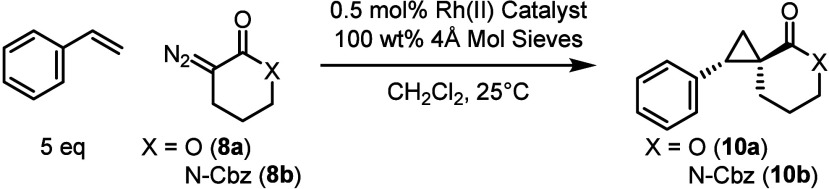
Catalyst Screen with **8a** and **8b** in Styrene Cyclopropanation

**Entry**	**X** ^a^	**Rh(II) Catalyst**	**Yield (%)** ^b^	**d.r. (** ***E*** **:** ***Z*** **)**	***ee*** **(%)** ^c^
1	O	Rh_2_(*S*-DOSP)_4_	74	6.5:1	5
2	O	Rh_2_(*S*-TPPTTL)_4_	66	4:1	–7
3	O	Rh_2_(*S*-NTTL)_4_	77	7:1	34
4	O	Rh_2_(*S*-TCPTAD)_4_	70	7:1	20
5	O	Rh_2_(*S*-2-Cl,5-BrTPCP)_4_	52	4:1	–30
6	O	Rh_2_(*S*-*p*-BrTPCP)_4_	41	4:1	87
7	O	Rh_2_(*S*-*p*-PhTPCP)_4_	85	7:1	90
8^d^	O	Rh_2_(*S*-*p*-PhTPCP)_4_	69	11:1	94
9	N-Cbz	Rh_2_(*S*-*p*-PhTPCP)_4_	76	8:1	96
10^d^	N-Cbz	Rh_2_(*S*-*p*-PhTPCP)_4_	75	8:1	98

a 3 h slow addition of diazo when X = O, fast diazo addition when X = N-PG.

b Yield reported as a mixture of diastereomers.

c Enantioselectivity of *E*-diastereomer.

d Reaction run at −78 °C and left to naturally warm to 25 °C.

An alternative entry into these spirocyclopropane scaffolds is to utilize the unsaturated lactone **11**. This would generate a donor/acceptor carbene and thus, could potentially give even higher stereoselectivity in the cyclopropanation. Doyle reported the use of this diazo compound with dirhodium tetracarboxamidate catalyst, Rh_2_(*S*,*R*-MenthAZ)_4_ in styrene cyclopropanation,^6a^ and found that **12** could be generated with good enantioselectivity (84% *ee*, Table 3, entry 2). The dirhodium tetracarboxylate catalysts examined, however, could only achieve low levels of enantioinduction, both in Doyle’s study (14% *ee*, Table 3 entry 1)^6a^ and this current study (31% *ee*, Table 3 entry 3). Therefore, in this case, **8a** appears to have a broader potential than **11** for highly enantioselective cyclopropanation.

**Table 3:**
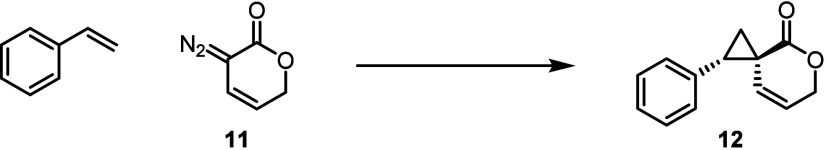
Reaction with Unsaturated 3-Diazo Lactone **11**

**Entry**	**Rh(II) Catalyst**	**Yield (%)**	**d.r. (** ***E*** **:** ***Z*** **)**	***ee*** **(%)** ^a^
1^b^,^c^	Rh_2_(*S*-DOSP)_4_	60	>20:1	14
2^b^,^c^	Rh_2_(*S*,*R*-MenthAZ)_4_	74	>20:1	84
3^d^	Rh_2_(*S*-*p*-PhTPCP)_4_	81	3:1	31

a Enantioselectivity of *E*-diastereomer.

b Doyle, *J. Am. Chem. Soc.* **2006**, 128, 16038–16039.

c 2.5 eq styrene, 1.0 mol % Rh_2_L_4_, CH_2_Cl_2_, reflux, 8 h slow addition of diazo.

d 5.0 eq styrene, 0.5 mol % Rh_2_(*S*-*p*-PhTPCP)_4_, CH_2_Cl_2_, 25 °C, 3 h slow addition of diazo.

We next examined the reaction with α-diazo ε-caprolactone **13**. Under the standard conditions at 25 °C with styrene, increasing the size of the lactone to the 7-membered ring resulted in the formation of a mixture of products, the desired cyclopropane **14a** as well as the α,ß-unsaturated side-product **15** in a 3:1 (**14a**:**15**) ratio (Table 4, see SI Figure 4 for details). It is established that reactions with acyclic alkyl-substituted α-diazo carbonyls, an intramolecular 1,2-hydride shift is commonly observed.^15^ In this study, the elimination pathway was not seen until a 7-membered ring system was used. Presumably, the larger ring is sufficiently flexible to accommodate a favorable alignment for a 1,2-hydride shift to occur. Fox demonstrated (Figure 1B) that even though ß-tertiary carbons have a higher propensity for elimination, the choice of catalyst ligand and temperature strongly influence the selectivity of the inter- and intramolecular reactions.^6c^ Following the Fox precedence, repeating the reaction at −78 °C resulted in clean formation of cyclopropane **14a** in >30:1 d.r. (*E*:*Z*) with trace amounts of the elimination product **15**. Furthermore, **14a** was formed with extremely high enantiocontrol (99% *ee*). In order to illustrate the potential scope of this transformation, the reaction was also conducted with *o*-chlorostyrene, which generated **14b** in 70% yield, with excellent stereocontrol (>30:1 d.r. and 99% *ee*).

**Table 4:**
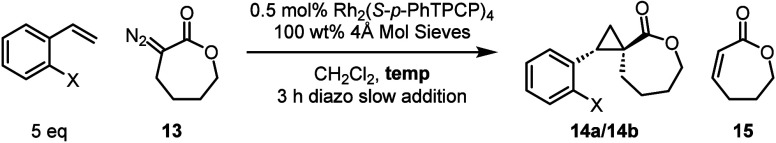
Reaction with α-Diazo ε-Caprolactone **13**

**Entry**	**Temp (°C)**	**X**	**Product**	**Yield (%)**	**14:15**	**d.r. (** ***E*** **:** ***Z*** **)**	***ee*** **(%)**
1	25	H	**14a**	29	3:1	15:1	98
2	–78 to 25	H	**14a**	49	>30:1	>30:1	99
3	–78 to 25	Cl	**14b**	70	>30:1	>30:1	99

Having established the optimum reaction conditions, a substrate scope was conducted, the results of which are shown in Table 5. These reactions were conducted at ambient temperature, as some of the substrates were not sufficiently soluble for the reactions to be conducted at lower temperatures. Styrene derivatives with various electron-donating and withdrawing groups, as well as naphthalene and heterocyclic derivatives were examined (**16–27**). Overall, the lactone **8a** and lactam **8b** reactions gave similar results; while the diastereoselectivity was moderate (3:1 to 9:1 d.r. (*E*:*Z*)), the enantioselectivity was generally high (80 to 98% *ee*). In addition to styrene-type substrates, the reaction is also effective with 1,1-disubtituted alkenes. 1,1-Diphenylethylene aligned with its monosubstituted styrene counterpart, **28a** in 62% yield and 90% *ee*. We also subjected N-Boc-*exo*-methylene substrates to the reaction, as the products generated from these reactions would be of pharmaceutical interest. Products **29a** and **30a** were both formed in 77% *ee*, furnishing a 10-azadispiro[5.5.1]­tridecane and 12-azatrispiro[5.5.2.1]­hexadecane scaffold, in 59% and 61% yield, respectively. The relative stereochemistry of these compounds can be readily determined from the ^1^H NMR due to the distinctive shielding caused by the aryl group. This was confirmed by the X-ray structure of **24a**, which was also used to determine its absolute configuration. The absolute configurations of the other cyclopropane products are tentatively assigned by analogy.

**Table 5:**
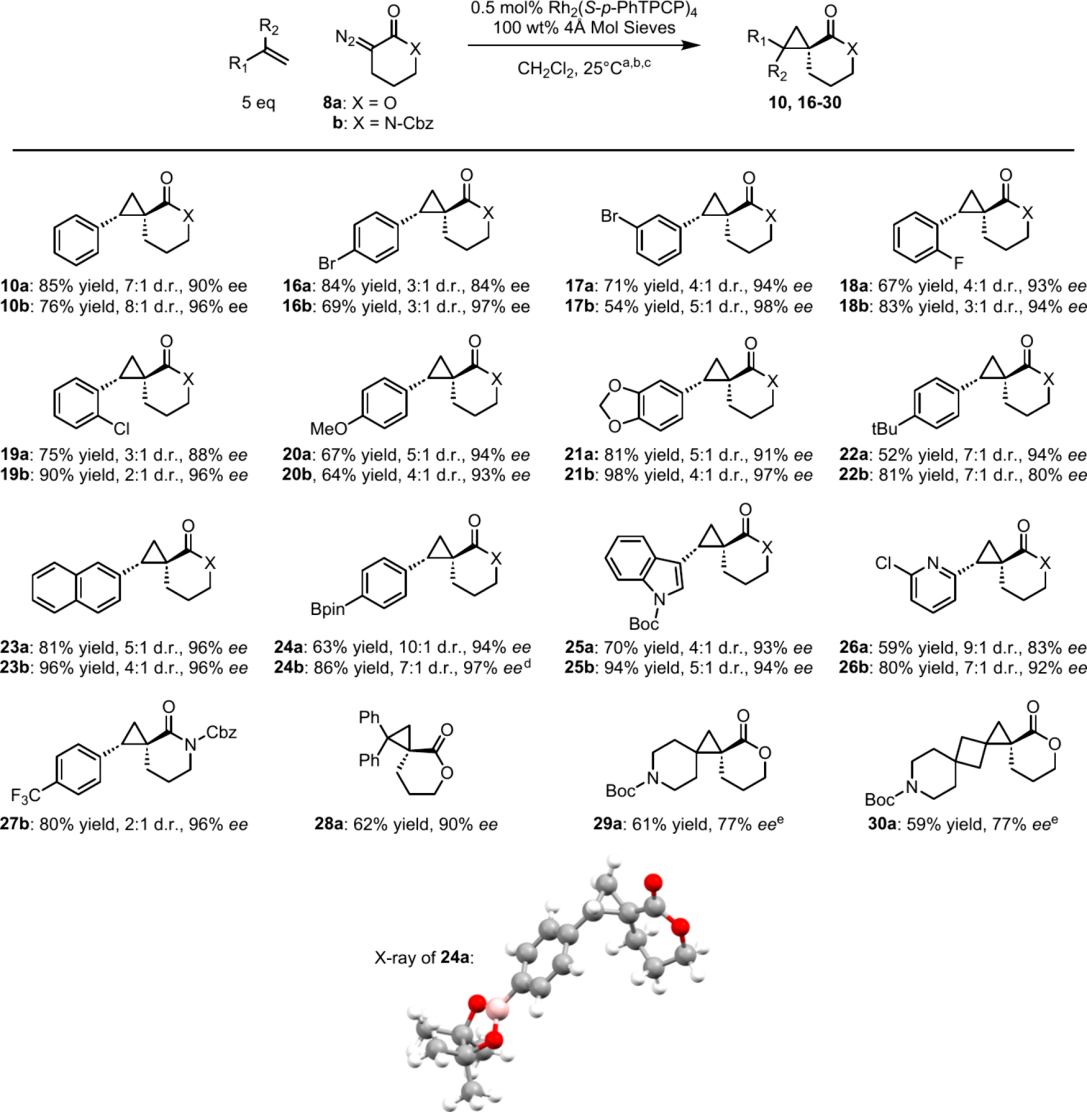
Substrate Scope with **8a** and **8b**

a 3 h slow addition of diazo when X = O, fast diazo addition when X = N-PG.

b Yield reported as a mixture of diastereomers.

c Enantioselectivity of E-diastereomer.

d Converted to **16b** for chiral HPLC analysis (see SI page S87 for details).

e 3 eq of trap was used.

Lastly, we examined the reaction with the 1,2-disubstituted cyclic alkene, N-Boc-2,5-dihydropyrrole (**31**, Scheme 1). The cyclopropanation of **31** offers ready access to a range of pharmaceutically relevant scaffolds.^16^ The reaction of this substrate with **8a** and **8b** proceeded with exclusive formation of the *exo*-diastereomer, resulting in a 3-azaspiro­[bicyclo[3.1.0]­hexane]-6-lactone (**32a**) or N-Cbz lactam (**32b**) substructure. In previous studies, we have shown that the reaction of **31** with an acceptor-only carbene results in cyclopropanation,^16^ whereas the reaction with a donor/acceptor carbene results in C–H functionalization.^17^ Consequently, the carbenes derived from **8a** and **8b** are more similar to acceptor-only carbenes although the cyclopropanation of **31** is more diastereoselective than what was observed with acceptor-only carbenes derived from α-diazoacetates.^16^

**Scheme 1:**
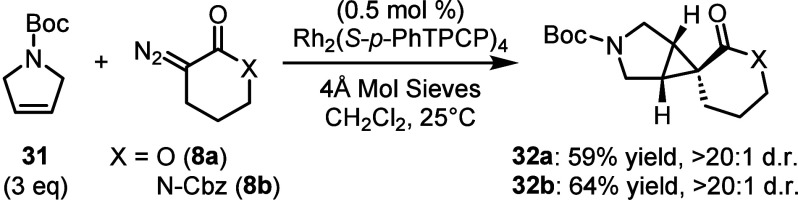
Reaction of **8a** and **8b** with 2,5-Dihydropyrrole (**31**)

To demonstrate the practicality of this methodology, we explored whether the reaction can be conducted with low catalyst loading.^14,16,18^ We have previously demonstrated that Rh_2_(*S*-*p*-PhTPCP)_4_-catalyzed cyclopropanation reactions with aryldiazoacetates retains high levels of enantioselectivity down to a catalyst loading of 0.001 mol %.^14^ However, high TON catalysis with acceptor-only carbenes has tended to be less accessible as the carbene is more reactive.^16,19^ Accordingly, we conducted a series of experiments on the cyclopropanation of styrene with the α-diazo lactone **8a**, with steadily decreasing amounts of Rh_2_(*S*-*p*-PhTPCP)_4_ (Table 6). The reactions were carried out at 1.0 mmol scale and in dichloromethane at 25 °C. Even though extremely low catalyst loadings were not expected to be compatible with an acceptor carbene, remarkably, the reactions went readily to completion down to a catalyst loading of 0.0005 mol %, retaining similar yields and levels of diastereoselectivity (8:1 to 9:1 d.r. (*E*:*Z*)) and enantioselectivity (90% *ee*) (entries 1–6). Initially, attempted reactions with even lower catalyst ratios showed some variability in reaction completion. In response to this, a highly purified sample of diazolactone **8a** was prepared (see SI Figure S5). Using this new sample, highly reproducible reactions (repeated 4 times) were obtained at the 0.0001 mol % (entry 7) and 0.00005 mol % (entry 8) catalyst loading, achieving even higher yield (90%) and the same levels of diastereoselectivity and enantioselectivity. New glassware, syringes, needles, septa, and magnetic stirrers were used in these reactions to avoid any cross contamination. A control experiment was conducted in the absence of catalyst (entry 9), and after 90 h, the diazolactone 8a remained unchanged. Thus, under the optimized conditions, a reaction at ambient temperature can be conducted with a catalyst turnover number of 1,740,000.

**Table 6:**
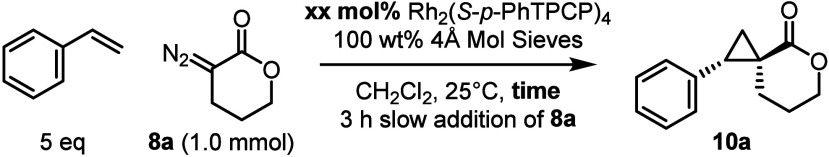
Low Catalyst Loading Studies with **8a**

**Entry**	**xx** **(mol %)**	**Time (h)** ^a^	**Yield (%)** ^b^	**d.r. (** ***E*** **:** ***Z*** **)**	***ee*** **(%)** ^c^	**TON**
1	0.5	1	79	9:1	90	170
2	0.05	3	79	8:1	90	1,600
3	0.01	3	82	8:1	90	8,200
4	0.005	20	82	8:1	90	16,000
5	0.001	20	85	8:1	90	86,000
6	0.0005	90	76	8:1	90	150,000
7^d^	0.0001	90	87 ± 8	9:1	90	870,000
8^d^	**0.00005**	**90**	**87 ± 5**	**9:1**	**90**	**1,740,000**
9	*no catalyst*	90	0^e^	–	–	–

a Time stirred after complete addition of diazo.

b Yield reported as a mixture of diastereomers.

c Enantioselectivity of *E*-diastereomer.

d Reaction conducted with extensively purified diazolactone **8a**. These reactions were repeated four times.

e No reaction – diazolactone **8a** was not consumed.

## Conclusion

In conclusion, these studies showed that highly enantioselective cyclopropanations can be conducted with α-diazo δ-lactones and δ-lactams as carbene precursors. Even higher levels of diastereoselectivity and enantioselectivity can be obtained with the 7-membered counterpart (>30:1 d.r., 99% *ee*), although the reaction needed to be carried out at low temperatures to avoid a competing 1,2-hydride shift. The optimum catalyst for these reactions is Rh_2_(*S*-*p*-PhTPCP)_4_, and it is capable of operating under extremely low catalyst loading. Complete consumption of the diazo compound can be achieved at 25 °C with a catalyst loading of 0.00005 mol %, resulting in 1,740,000 catalyst turnover numbers (TON). The carbenes used in these studies are not the conventional donor/acceptor carbenes, lacking resonance stabilization from the donor group of the highly electrophilic carbene intermediate. These studies show expansion of the range of carbene precursors that can be effective with the chiral dirhodium tetracarboxylate catalysts.

## Supplementary Material



## Notes

Complete experimental procedures, materials, compound characterizations, and crystal structure for compound **24a** (PDF)
