# Supplementary material for: Enantioselective Synthesis of Spirolactones and Spirolactams Under Low Catalyst Loadings
Source: J Am Chem Soc. 2026 Mar 5;148(10):11352–8. doi: 10.1021/jacs.6c01407 (PMC13003446; doi:10.1021/jacs.6c01407)

Enantioselective Synthesis of Spirolactones and Spirolactams Under Low Catalyst Loadings

Takeru Saito[1], Antonio Navarro[2], Huw M. L. Davies[1*]

[1]Department of Chemistry, Emory University, Atlanta, Georgia 30322, United States
[2]Lilly Research Laboratories, Eli Lilly and Company, Indianapolis, Indiana 46285, United States

Corresponding author email: hmdavie@emory.edu

Supporting Information
Complete experimental procedures, materials, and compound characterizations

Table of Contents

General Considerations S2
Supplementary Information: Figures and Tables S7
Supplementary Information: Determination of Diastereoselectivity S13
References S46
Experimental Procedures S48
Spectra and Chromatograms S101
X-ray Structure of 24a S224

General Considerations

All reactions were performed under a positive pressure of inert atmosphere (nitrogen or argon in a balloon) in flame-dried glassware fitted with rubber septa or septa-lined plastic vial caps, unless specified otherwise. Ambient temperature was measured as 25°C. Unless otherwise noted, refluxed reactions were done so on metal heating blocks in round-bottom flasks (RBFs) equipped with Heidolph Radleys findensers. -78°C was achieved via acetone and dry ice in a vacuum-sealed dewar. Solvents denoted as 'SPS grade' [dichloromethane (CH_2Cl_2), acetonitrile (MeCN), toluene (PhMe), tetrahydrofuran (THF), α,α,α-trifluorotoluene (TFT)] were dried by passing through alumina columns under argon in a Glass Contour Solvent Purification System (SPS). Solvents denoted as 'distilled' were done so at ambient pressure from powdered calcium hydride (CaH_2) and stored in a flask under an inert atmosphere over activated 4Å molecular sieves (Mol Sieves), bubbling with argon for 15 min, then letting sit at least 24 h before use. 4Å Mol Sieves were activated by high temperature (> 200°C) under vacuum for at least 12 h and stored in an oven until use. The amount of 4Å Mol Sieves denoted as '100 wt%' was calculated as 1 g of 4Å Mol Sieves for 1 mmol of diazo utilized. All reagents were purchased from commercial suppliers (Sigma Aldrich, Oakwood, Ambeed, TCI, Alfa Aesar, CombiBlocks, Acros) and used directly without further purification, unless otherwise noted. Reactions were monitored by thin-layer chromatography (TLC) on silica-coated aluminum sheets, visualizing with 254 nm UV light and staining, as indicated. Automated flash column chromatography was performed on Biotage Isolera flash chromatography systems using Silicycle SiliaFlash P60 silica gel (60 Å pore size, 40-63 μm particle size, 230-400 mesh) and reagent grade solvents.

NMR Spectroscopy

Raw FID files were processed and analyzed using the MestReNova 15.1 software from Mestrelab Research S. L.. All ^{1}H NMR spectra were recorded at 400 MHz on Bruker AVIII-400 or Bruker NEO-400 spectrometers. ^{13}C NMR spectra were recorded at 101 MHz, while ^{19}F NMR spectra were recorded at 376 MHz. NMR spectra were obtained using deuterated chloroform ($CDCl_3$), with or without 0.03% TMS, with residual solvent serving as internal standard (7.26 ppm for ^{1}H and 77.16 ppm for ^{13}C). ^{1}H NMR chemical shifts are reported in parts per million (ppm). Abbreviations for signal multiplicity are as follows: s = singlet, d = doublet, t = triplet, q = quartet, p = pentet, hept = heptet, m = multiplet, dd = doublet of doublets, br s = broad singlet, app = apparent multiplicity, etc. ^{19}F NMR and ^{13}C NMR chemical shifts are reported in parts per million (ppm) and when appropriate, multiplicity. Coupling constants (J values) were calculated directly from the spectra. All deuterated chloroform ($CDCl_3$) utilized was purchased from Sigma Aldrich, neutralized with a small addition of oven-dried potassium carbonate (K_2CO_3), and dried with activated 4Å Mol Sieves, letting sit overnight before use.

Determination of Diastereoselectivity
Diastereoselectivity was determined from the crude ^{1}H NMR prior to any chromatographic purification.

Determination of Enantioselectivity
Enantiomeric excess was determined by either one of two instruments in the 210 nm UV trace, based on the racemic trace. Samples were dissolved in HPLC grade solvent mixes of 100% heptane or 20:80 IPA:Heptane, depending on sample solubility.

Chiral SFC: Waters Acquity UPC^{2} supercritical fluid chromatography system with supercritical fluid CO_2 with a co-solvent of 1:1 MeOH:IPA with 0.2% FA. The default pressure for SFC runs is 2000 psi, unless otherwise specified.

SFC columns:
ChiralCel OZ-3 (3.0 µm, 3.0 mm x 150 mm)
ChiralCel OJ-3 (3.0 µm, 3.0 mm x 150 mm)

Chiral HPLC: Agilent 1100 Chiral HPLC system with an isocratic percentage of IPA in *n*-hexane solvent system with chiral HPLC columns.

Chiral HPLC columns:
ChiralPak AD-H (5 µm particle size, 4.6 mm x 250 mm)
ChiralPak AS-H (5 µm particle size, 4.6 mm x 250 mm)
ChiralCel OD-H (5 µm particle size, 4.6 mm x 250 mm)
ChiralCel OD (5 µm particle size, 4.6 mm x 250 mm)

Optical rotation
Optical rotations were measured on Rudolph Research Analytical Automatic Polarimeter APIV-1W using the scale: Specific Rotation setting (wavelength 589 nm, cell length 100 mm, Quartz temperature correction, and multiples of 5s). All samples were prepared in chloroform ($CHCl_3$).

Samples measured as a mixture of diastereomers are denoted as such.
Reported as: $[\alpha]^{\mathbf{temp}}_{D}$ = **angle**° (c = **concentration**, $CHCl_3$, **enantioselectivity**)

HRMS: High-resolution mass spectra were taken on a Thermo Finnigan LTQ-FTMS spectrometer with positive (+p) Atmospheric Pressure Chemical Ionization (APCI).

Abbreviations
Boc = *tert*-butyloxycarbonyl
Cbz = Carbobenzoxy
CV = Column Volume
d.r. = diastereomeric ratio
RBF = Round-bottom flask
R_f = Retention factor
TLC = Thin-Layer Chromatography
UV = Ultraviolet

Chemical Abbreviations
p-ABSA = *para*-Acetamidobenzenesulfonyl azide (Cas No. 2158-14-7)
Cbz-OSu = N-(Benzyloxycarbonyloxy)succinimide (Cas No. 13139-17-8)
DBU = 1,8-Diazabicyclo[5.4.0]undec-7-ene (Cas No. 6674-22-2)
DCC = Dicyclohexylcarbodiimide (Cas No. 538-75-0)
FA = Formic Acid (Cas No. 64-18-6)
LiHMDS = Lithium bis(trimethylsilyl)amide/Lithium hexamethyldisilazide (Cas No. 4039-32-1)
NaH = Sodium hydride (Cas No. 7646-69-7)
NaOH = Sodium hydroxide (Cas No. 1310-73-2)
o-NBSA = *ortho*-Nitrobenzenesulfonyl azide (Cas No. 6655-31-8)
TBAB = Tetrabutylammonium bromide (Cas No. 1643-19-2)
TEA = Triethylamine (Cas No. 121-44-8)

Solvent Abbreviations
DCE = 1,2-Dichloroethane
DCM/CH_2Cl_2 = Dichloromethane
DMC = Dimethylcarbonate
EtOAc = Ethyl Acetate
EtOH = Ethanol
Et_2O = Diethyl ether
Hex = Hexanes
IPA = Isopropanol
MeCN = Acetonitrile
MeOH = Methanol
PhMe = Toluene
THF = Tetrahydrofuran
TFT = α,α,α-Trifluorotoluene

Dirhodium Catalysts Utilized

Rhodium Catalyst	MW (g/mol)	Cas No. and/or Reference
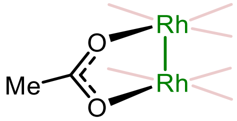 $Rh_2(OAc)_4$	441.99	15956-28-2
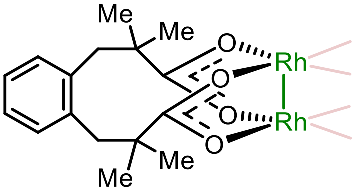 $Rh_2(esp)_2$	758.47	819050-89-0<br><br>*J. Am. Chem. Soc.* **2004**, 126, 15378–15379[1]
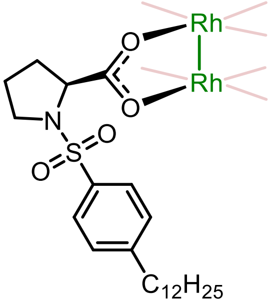 Rh_2(*S*-DOSP)$_4$	1896.22	179162-34-6<br><br>*J. Am. Chem. Soc.* **1996**, *118*, 6897–6907[2]
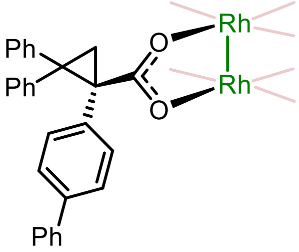 Rh_2(*S*-*p*-PhTPCP)$_4$	1763.71	1415389-64-8<br>(*R* catalyst)<br><br>*ACS Catal.* **2018**, *8*, 678–682[3]
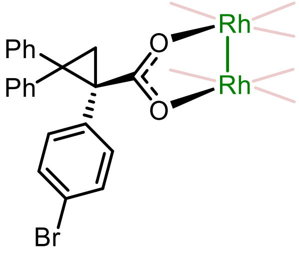 Rh_2(*S*-*p*-BrTPCP)$_4$	1774.87	1345974-62-0<br>(*R* catalyst)<br><br>*J. Am. Chem. Soc.* **2014**, *136*, 9792–9796[4]
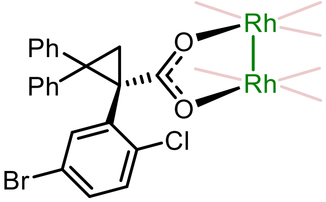 Rh_2(*S*-2-Cl,5-BrTPCP)$_4$	1912.67	*J. Am. Chem. Soc.* **2018**, *140*, 12247-12255[5]

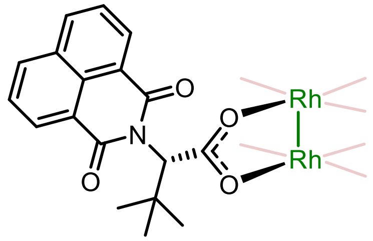 Rh₂(*S*-NTTL)₄	1447.13	*Chem Eur J*, **2010**, *16*, 3291[6]
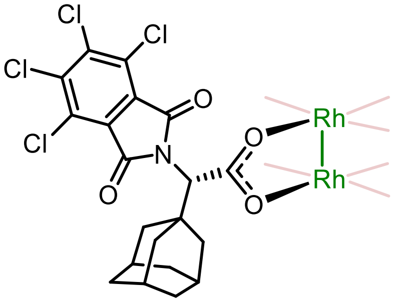 Rh₂(*S*-TCPTAD)₄	2110.44	915797-21-6 *Org. Lett.* **2006**, *8*, 5013–5016[7]
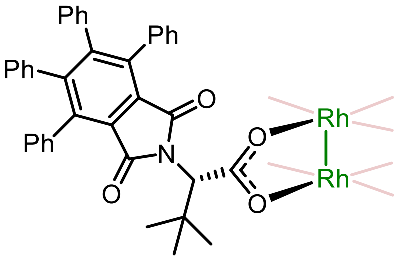 Rh₂(*S*-TPPTTL)₄	2464.45	*Nature*, **2018**, *564*, 395-399[8]

Supplementary Information: Figures and Tables

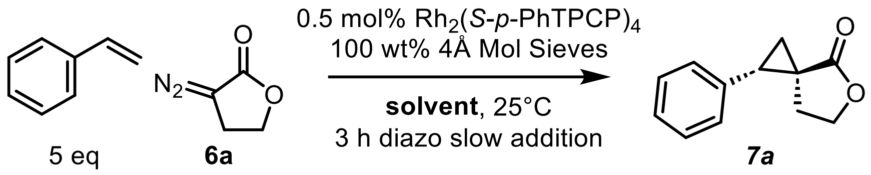

Entry	Solvent	Yield (%)	d.r. (*E*:*Z*)	*ee* (%)[a]
**1**	DCE	52	2.1:1	50
**2**	EtOAc	69	1.9:1	36
**3**	TFT	38	2.3:1	46
**4**	DMC	61	2.7:1	49

SI Figure S1. Solvent screen of styrene cyclopropanation with **6a**. a: *ee* of *E*-diastereomer

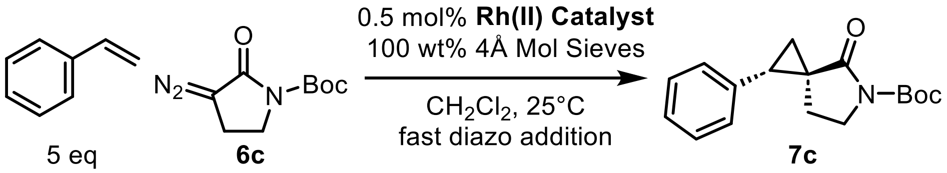

Entry	Rh(II) Catalyst	Yield (%)	d.r. (*E*:*Z*)	*ee* (%)
**1**	Rh_2(*S*-DOSP)$_4$	49	1.2:1	-8
**2**	Rh_2(*S*-TPPTTL)$_4$	48	0.9:1	-10
**3**	Rh_2(*S*-NTTL)$_4$	46	1.3:1	18
**4**	Rh_2(*S*-*p*-BrTPCP)$_4$	41	1.4:1	18
**5**	Rh_2(*S*-*p*-PhTPCP)$_4$	50	1.2:1	34

SI Figure S2. Full catalyst screen in styrene cyclopropanation with **6c**. a: *ee* of *E*-diastereomer

See SI Figure S7 for determination of diastereoselectivity by crude ^{1}H NMR

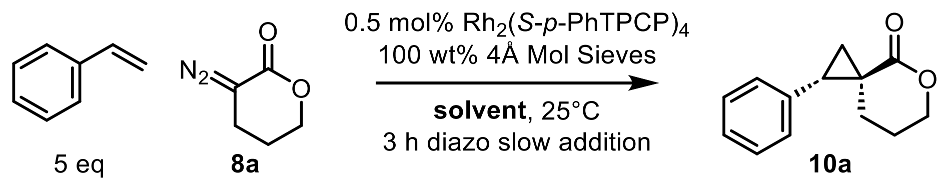

Entry	Solvent	Yield (%)	d.r. (*E*:*Z*)	*ee* (%)[a]
**1**	CH_2Cl_2	85	7:1	90
**2**	DCE	53	7:1	89
**3**	TFT	43	5:1	82
**4**	DMC	65	9:1	90

SI Figure S3. Solvent screen with diazo **8a**. a: *ee* of *E*-diastereomer

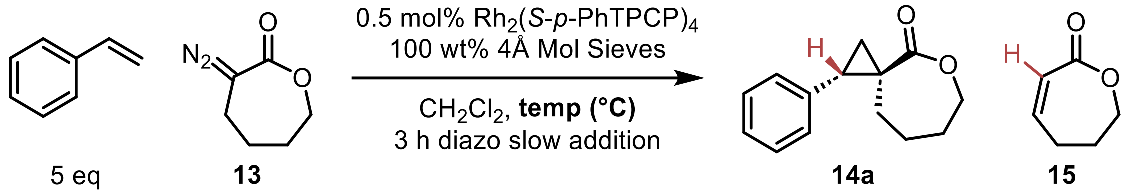

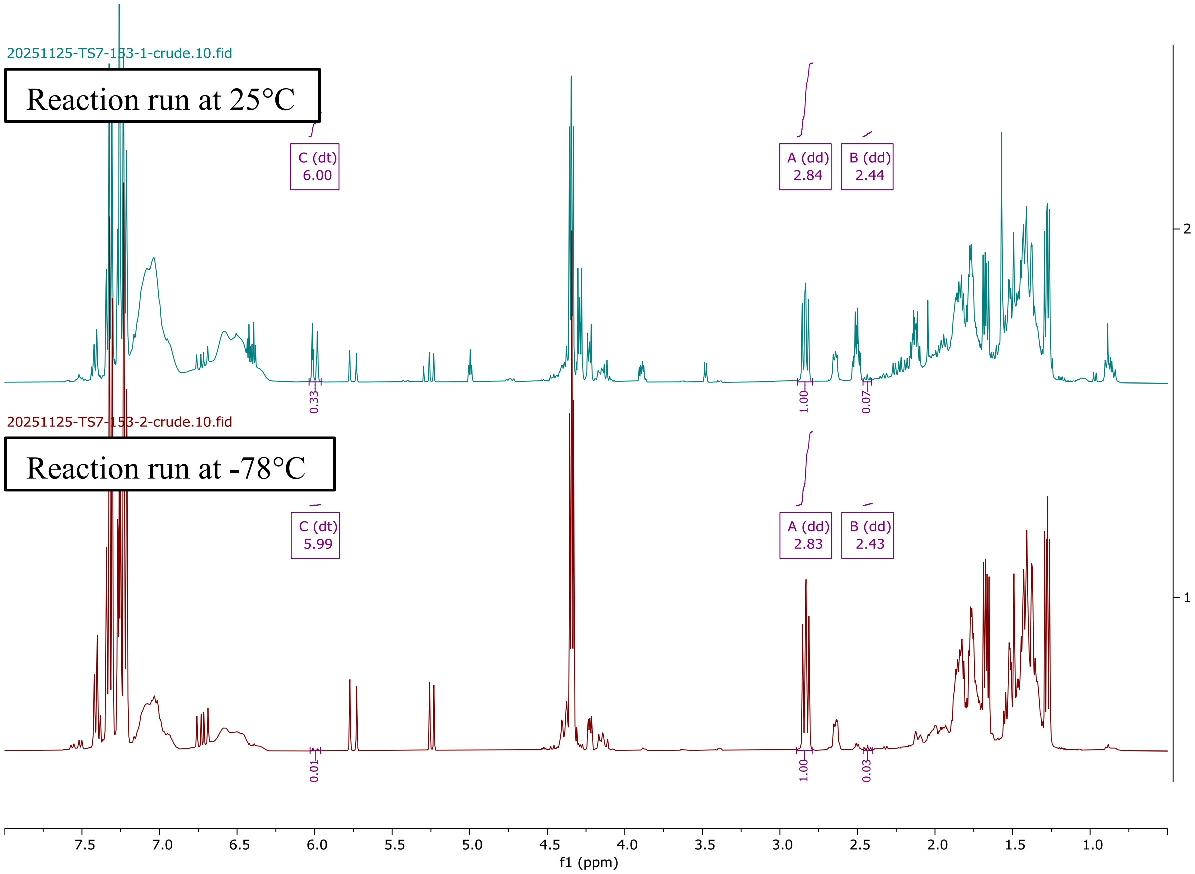

SI Figure S4. Crude ^{1}H NMR of reaction (w/ Rh_2(*S*-*p*-PhTPCP)$_4$) with diazo **13**, derived from ε-caprolactone.

Top spectrum is crude ^{1}H NMR of reaction run at 25°C.
Bottom spectrum is crude ^{1}H NMR of reaction run at -78°C.

Signal at 6.00 ppm (dt, J = 12.4, 2.0 Hz, 1H) was assigned as the α proton of the elimination product (**15**).
Signal at 2.83 (dd, *J* = 9.3, 7.1 Hz, 1H) was assigned as the benzylic proton of the *E*-diastereomer of the cyclopropanation product (**14a**).
Signal at 2.43 ppm (dd, J = 7.5, 7.5 Hz, 1H) was assigned as the benzylic proton of the *Z*-diastereomer of the cyclopropanation product.

SI Figure S5. Preparation of catalyst stock solution for low catalyst loading (TON studies)

All solution and reaction vials were prepared with new vials, septa, syringes, needles, and stir bars.

Diazo **8a** was synthesized according to the procedure outlined on page S53. After purification via Biotage, the collected fractions were combined and purified once more via flash column chromatography, eluting 100% CH_2Cl_2. The bright yellow fractions were combined and concentrated *in vacuo* to provide **8a** in high purity. Spectra are attached on the proceeding page.

Catalyst needed for 1.0 mmol of **8a** as limiting reagent. 2.5 mL of total solvent needed in styrene + catalyst + 4Å Mol Sieves solution.
Molecular weight of Rh_2(*S*-*p*-PhTPCP)$_4$ = 1763.71 mg/mmol

- 0.05 mol%, 881.9 μg
- 0.01 mol%, 176.4 μg
- 0.005 mol%, 88.19 μg
- 0.001 mol%, 17.64 μg
- 0.0005 mol%, 8.82 μg
- 0.0001 mol%, 1.76 μg
- 0.00005 mol%, 881.9 ng

Example procedure:
Vial 1: 3.2 mg of Rh_2(*S*-*p*-PhTPCP)$_4$ was weighed into a flame-dried 16 mL vial on an analytical balance. This was degassed and backfilled with argon, then left under an argon atmosphere via a balloon. The catalyst was diluted with 9.1 mL of CH_2Cl_2 to make a 351.65 μg/mL solution.

- 2.5 mL of Vial 1 solution dispensed for 0.05 mol% reaction.
- 0.5 mL of Vial 1 solution and 2.0 mL of fresh CH_2Cl_2 added for 0.01 mol% reaction.

Vial 2: 1.0 mL of the Vial 1 solution was taken out (351.65 μg/mL) into a flame-dried, degassed 16 mL vial, then diluted with 9.0 mL of fresh CH_2Cl_2 to make a 35.16 μg/mL total 10.0 mL solution.

- 2.5 mL of Vial 2 solution was taken out for 0.005 mol% reaction.
- 0.5 mL of Vial 2 solution and 2.0 mL of fresh CH_2Cl_2 added for 0.001 mol% reaction.

Vial 3: 1.0 mL of the Vial 2 solution was taken out (35.16 μg/mL) into a flame-dried, degassed 16 mL vial, then diluted with 9.0 mL of fresh CH_2Cl_2 to make a 3.52 μg/mL total 10.0 mL solution.

- 2.5 mL of Vial 3 solution was taken out for 0.0005 mol% reaction.
- 0.5 mL of Vial 3 solution and 2.0 mL of fresh CH_2Cl_2 added for 0.0001 mol% reaction.

Vial 4: 1.0 mL of the Vial 3 solution was taken out (3.52 μg/mL) into a flame-dried, degassed 16 mL vial, then diluted with 9.0 mL of fresh CH_2Cl_2 to make a 351.65 ng/mL total 10.0 mL solution.

- 2.5 mL of Vial 4 solution was taken out for 0.00005 mol% reaction.

This process was repeated with CH_2Cl_2 as solvent to confirm reproducibility for 0.0001 mol% and 0.00005 mol% catalyst loadings four times total (two runs in duplicates). 4.2 and 1.6 mg of catalyst weighed out initially. The yield reported in the main text for Table 6 entries 7 and 8 is an average of the four runs with standard deviation reported as error.

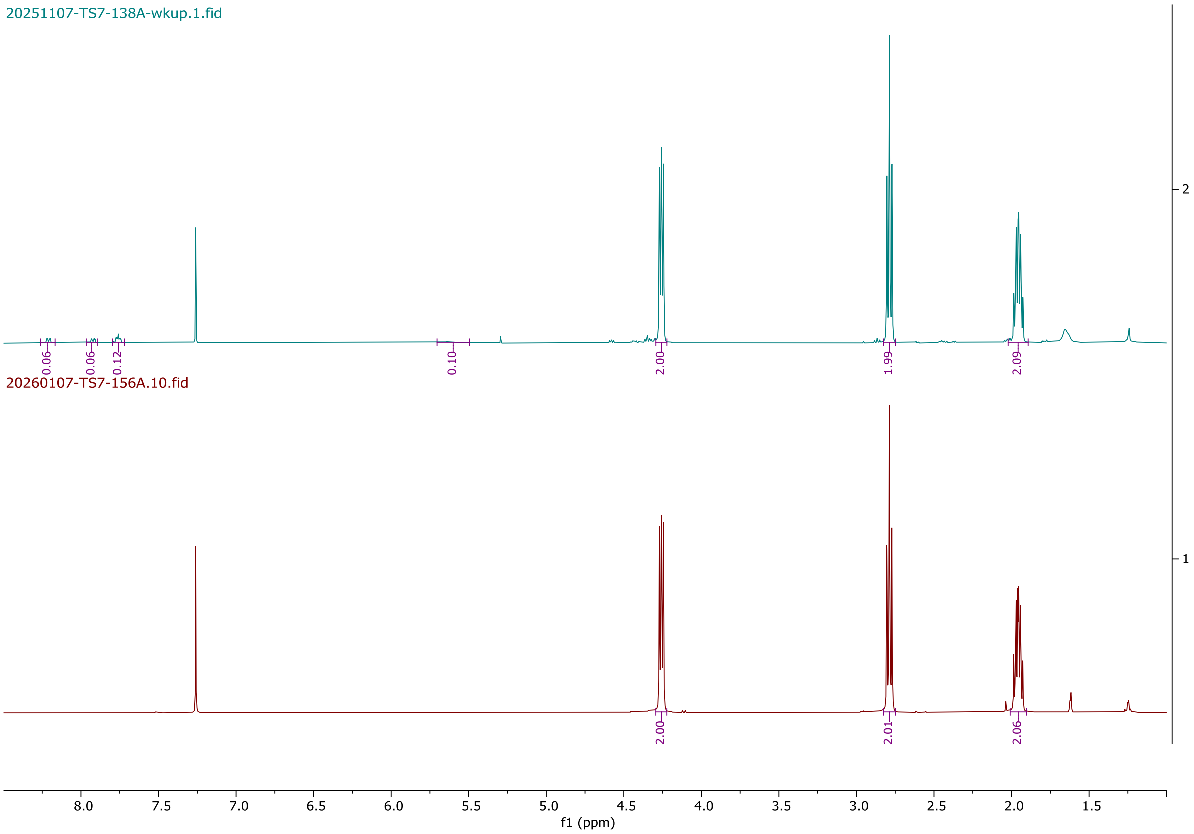

SI Figure S5.2. ^{1}H NMR spectra of high-purity diazo **8a**

Top spectrum is ^{1}H NMR of diazo **8a** used in substrate scope and part of TON studies. ~5 to 10% impurity is presumed to primarily be *o*-NBSA byproduct from diazo transfer.

Bottom spectrum is purified ^{1}H NMR of diazo **8a** used in entries 7 and 8 in Table 6 of main text

Supplementary Information: Determination of Diastereoselectivity

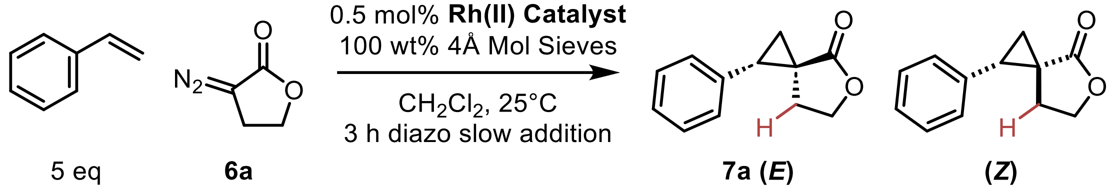

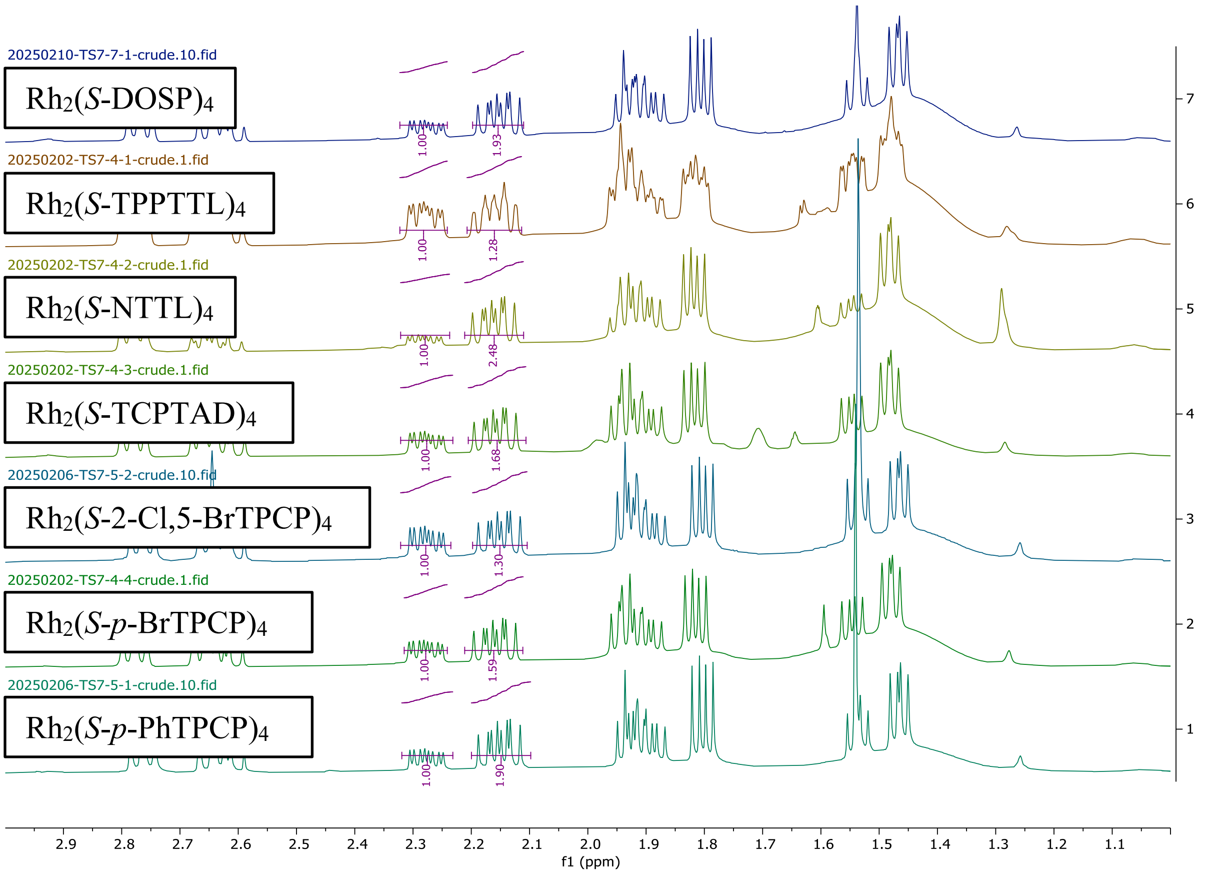

SI Figure 6. Diastereoselectivity from catalyst screen with diazo **6a**

Signal at 2.28 (ddd, *J* = 12.7, 7.2, 2.9 Hz, 1H) was assigned as one of the protons on C7 of the *Z*-diastereomer
Signal at 2.16 (ddd, *J* = 13.1, 8.9, 6.7 Hz, 1H) was assigned as one of the protons on C7 of the *E*-diastereomer

Catalysts and respective d.r. (*from top to bottom*)
Rh_2(*S*-DOSP)$_4$: 2.0:1
Rh_2(*S*-TPPTTL)$_4$: 1.3:1
Rh_2(*S*-NTTL)$_4$: 2.5:1
Rh_2(*S*-TCPTAD)$_4$: 1.7:1
Rh_2(*S*-2-Cl,5-BrTPCP)$_4$: 1.3:1
Rh_2(*S*-*p*-BrTPCP)$_4$: 1.6:1
Rh_2(*S*-*p*-PhTPCP)$_4$: 1.9:1

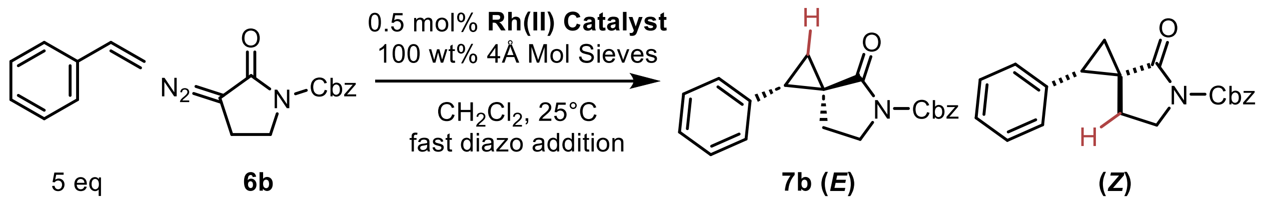

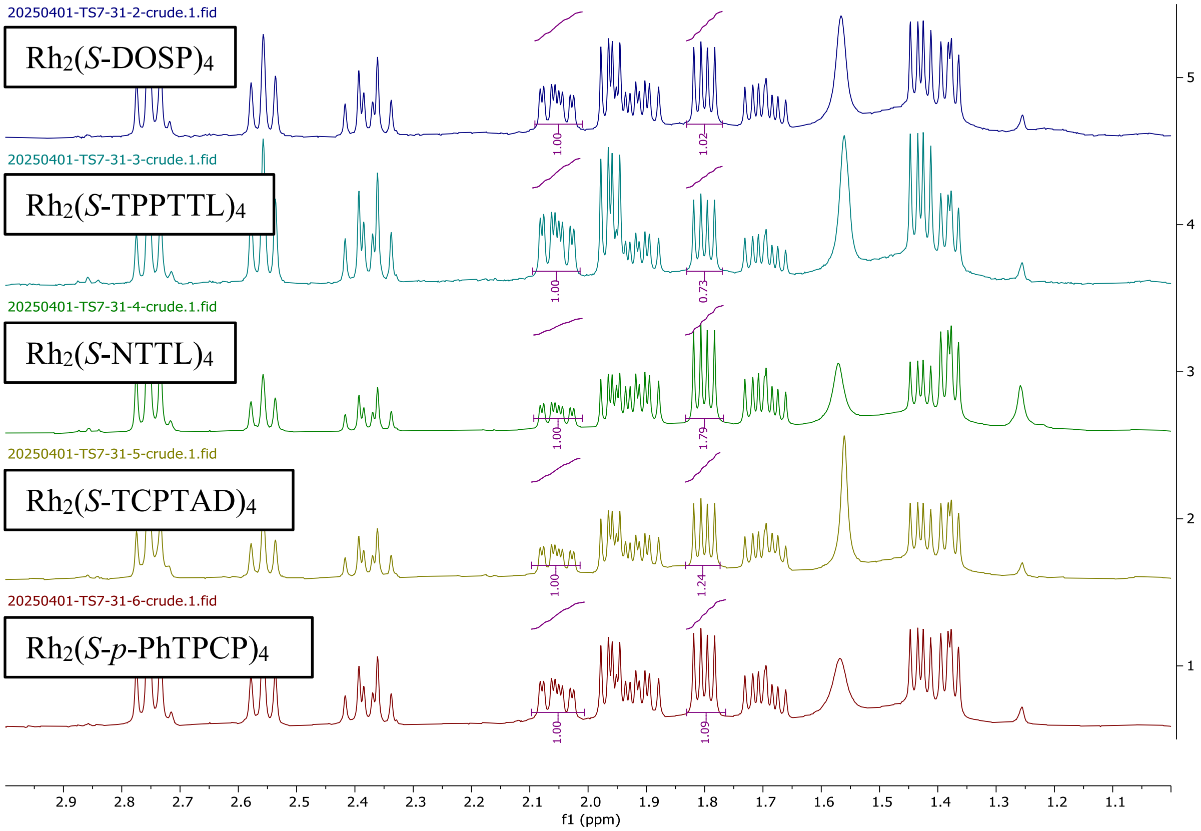

SI Figure S7. Diastereoselectivity from catalyst screen with diazo **6b**

Signal at 2.05 (ddd, J = 12.8, 7.8, 2.5 Hz, 1H) was assigned as one of the protons on C7 of the *Z*-diastereomer

Signal at 1.80 (dd, J = 9.4, 4.9 Hz, 1H) was assigned as one of the protons on C2 of the *E*-diastereomer

Catalysts and respective d.r. (*from top to bottom*)

Rh_2(*S*-DOSP)$_4$: 1.0:1

Rh_2(*S*-TPPTTL)$_4$: 0.7:1

Rh_2(*S*-NTTL)$_4$: 1.8:1

Rh_2(*S*-TCPTAD)$_4$: 1.2:1

Rh_2(*S*-*p*-PhTPCP)$_4$: 1.0:1

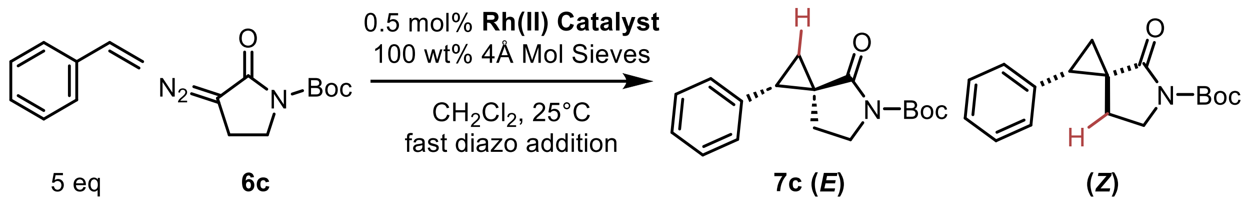

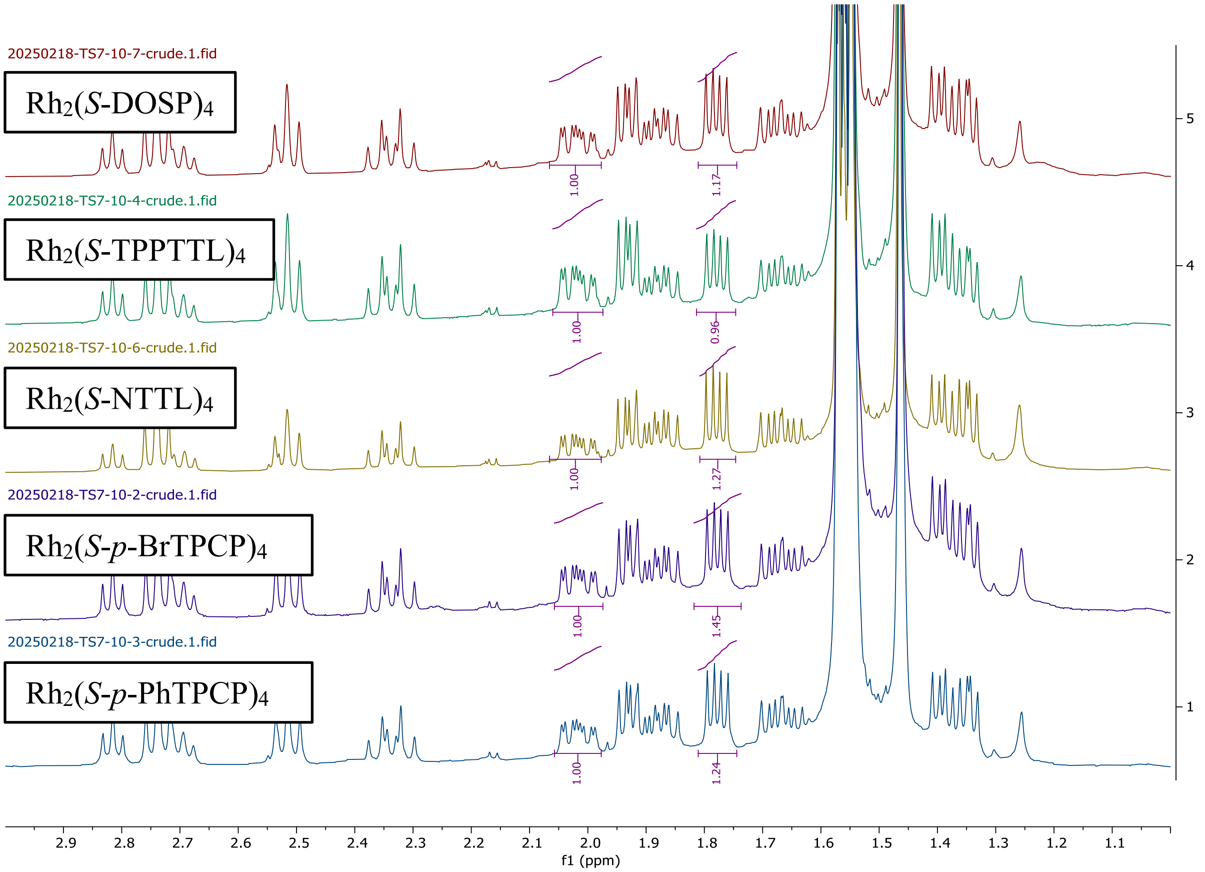

SI Figure S8. Diastereoselectivity from catalyst screen with diazo **6c**

Signal at 2.02 (ddd, J = 12.8, 7.7, 2.6 Hz, 1H) was assigned as one of the protons on C7 of the *Z*-diastereomer
Signal at 1.78 (dd, J = 9.3, 4.9 Hz, 1H) was assigned as one of the protons on C2 of the *E*-diastereomer

Catalysts and respective d.r. (*from top to bottom*)
Rh_2(*S*-DOSP)$_4$: 1.1:1
Rh_2(*S*-TPPTTL)$_4$: 1.0:1
Rh_2(*S*-NTTL)$_4$: 1.3:1
Rh_2(*S*-*p*-BrTPCP)$_4$: 1.5:1
Rh_2(*S*-*p*-PhTPCP)$_4$: 1.2:1

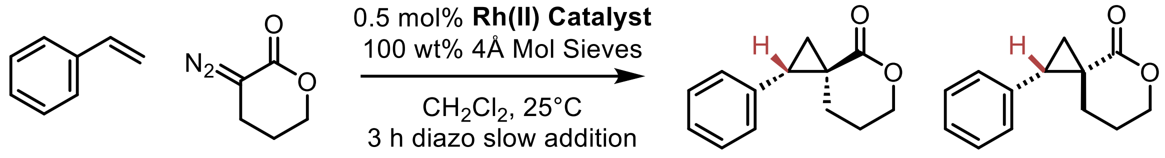

5 eq **8a** **10a (*E*)** **(*Z*)**

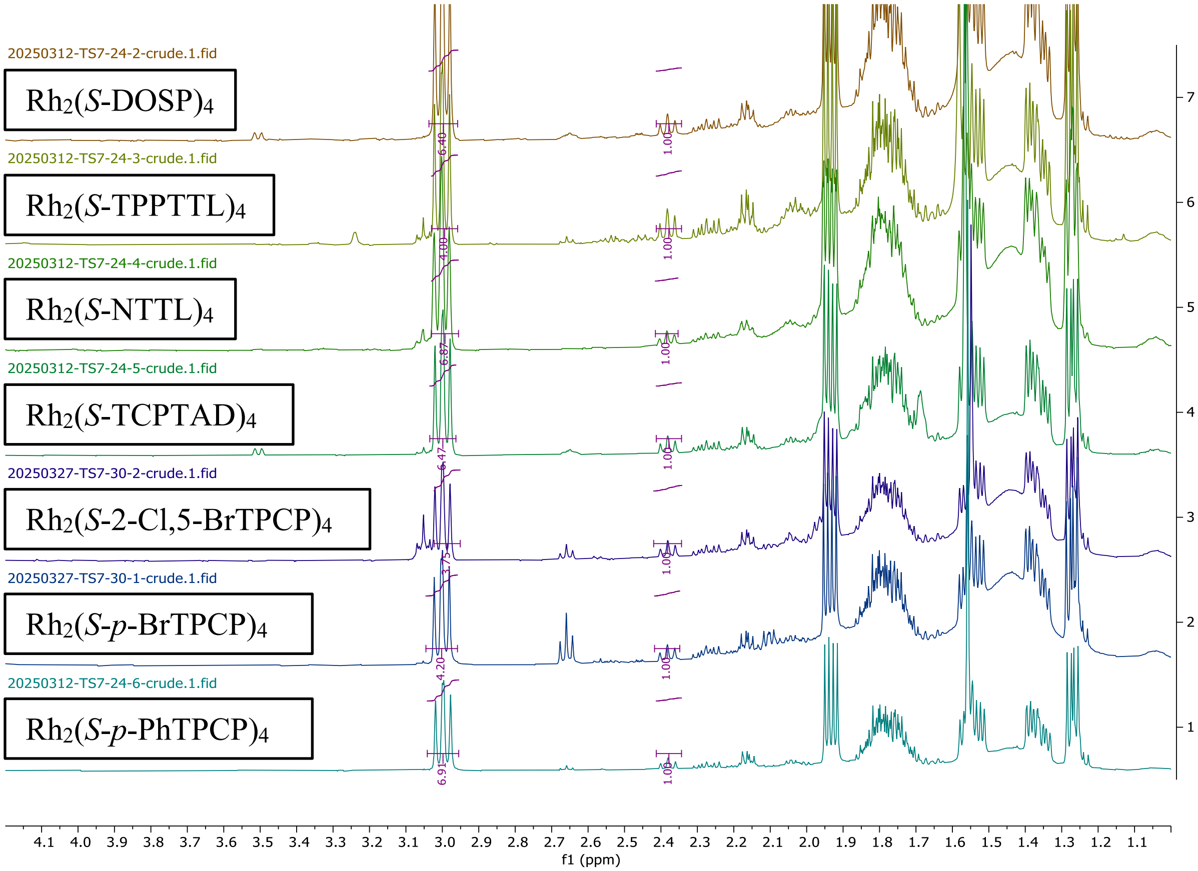

SI Figure S9. Diastereoselectivity from catalyst screen with diazo **8a**

Signal at 3.00 (dd, $J = 9.2$, 7.3 Hz, 1H) was assigned as the benzylic proton of the *E*-diastereomer
Signal at 2.38 (app t, $J = 8.1$ Hz, 1H) was assigned as the benzylic proton of the *Z*-diastereomer

Catalysts and respective d.r. (*from top to bottom*)
Rh_2(*S*-DOSP)$_4$: 6.4:1
Rh_2(*S*-TPPTTL)$_4$: 4.0:1
Rh_2(*S*-NTTL)$_4$: 6.9:1
Rh_2(*S*-TCPTAD)$_4$: 6.5:1
Rh_2(*S*-2-Cl,5-BrTPCP)$_4$: 3.8:1
Rh_2(*S*-*p*-BrTPCP)$_4$: 4.2:1
Rh_2(*S*-*p*-PhTPCP)$_4$: 7:1

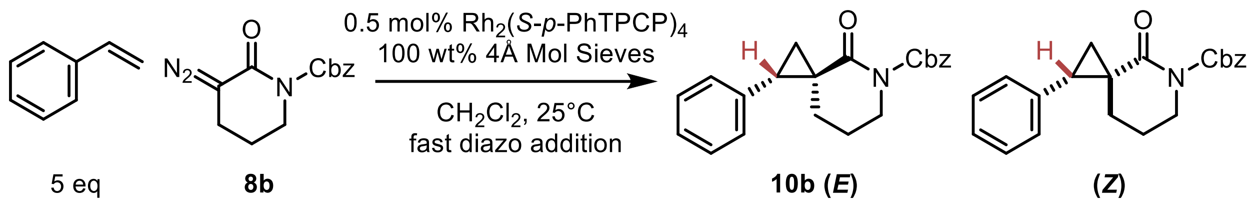

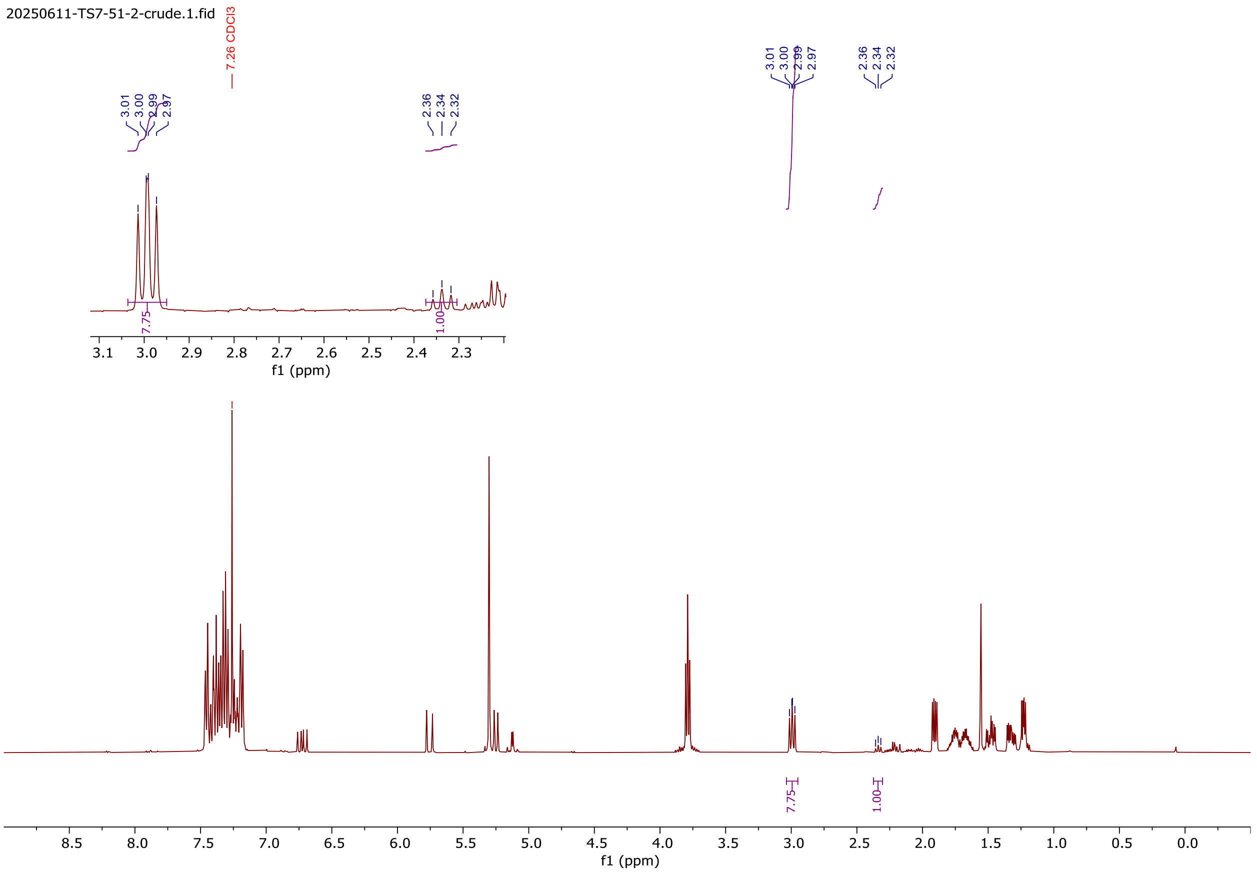

SI Figure S10. Diastereoselectivity from catalyst screen with diazo **8b**

Signal at 2.99 (dd, J = 9.2, 7.3 Hz, 1H) was assigned as the benzylic proton of the *E*-diastereomer
Signal at 2.34 (app t, J = 8.0 Hz, 1H) was assigned as the benzylic proton of the *Z*-diastereomer

Rh_2(*S*-*p*-PhTPCP)$_4$: 8:1

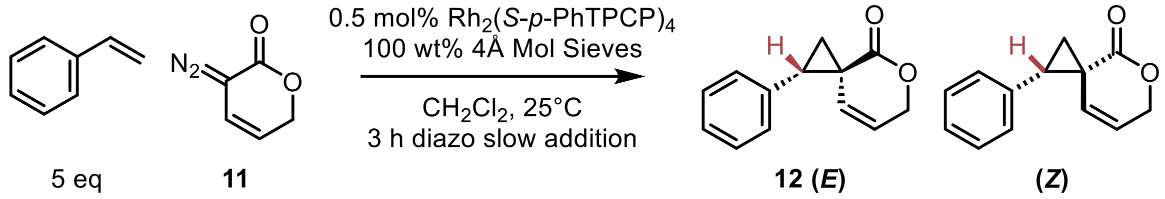

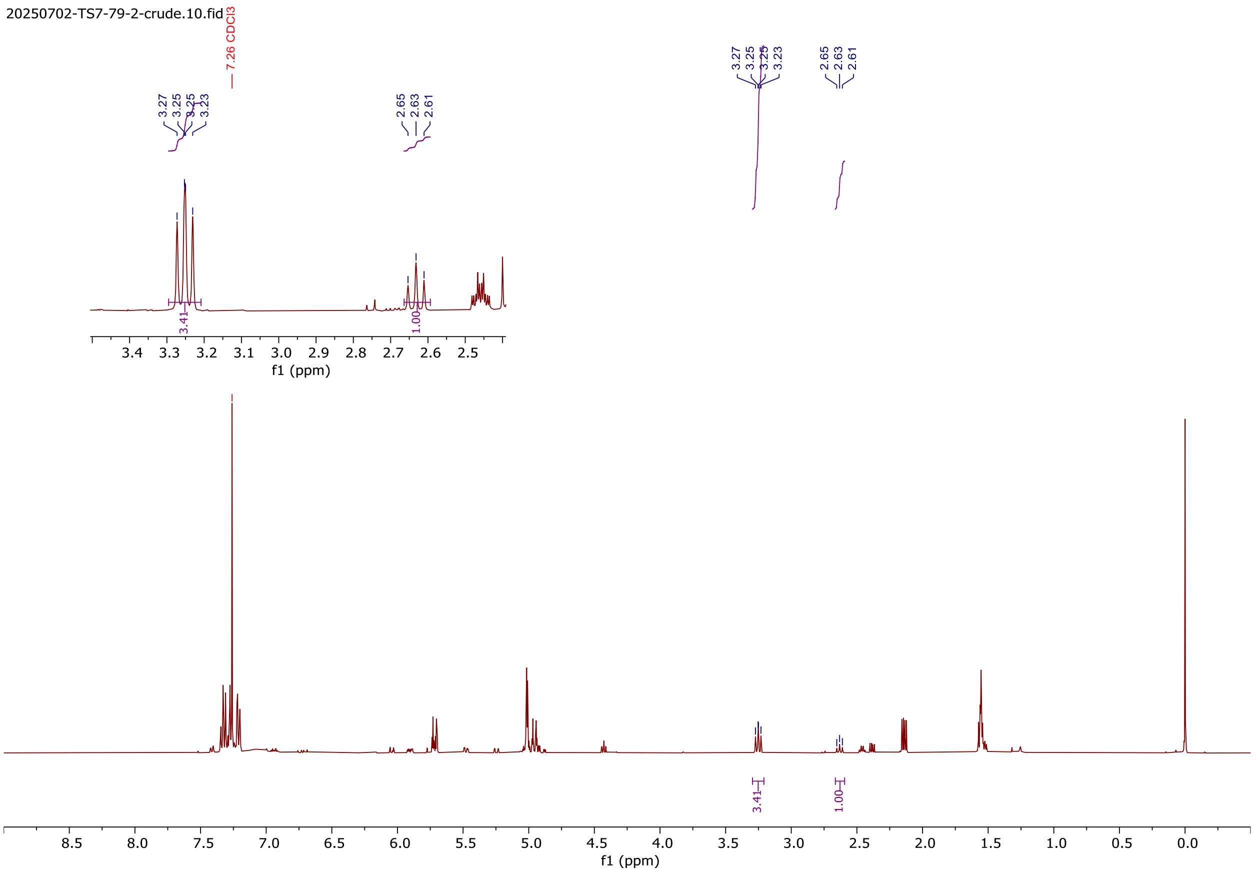

SI Figure S11. Diastereoselectivity from catalyst screen with diazo **11**

Signal at 3.25 (dd, J = 9.0, 7.8 Hz, 1H) was assigned as the benzylic proton of the E-diastereomer
Signal at 2.63 (app t, J = 8.6 Hz, 1H) was assigned as the benzylic proton of the Z-diastereomer

Rh_2(S-p-PhTPCP)$_4$: 3:1

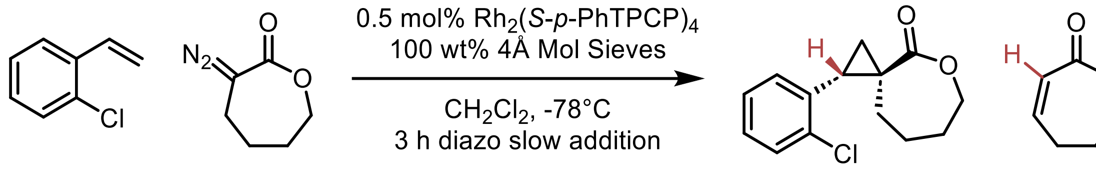

5 eq **13** **14b** **15**

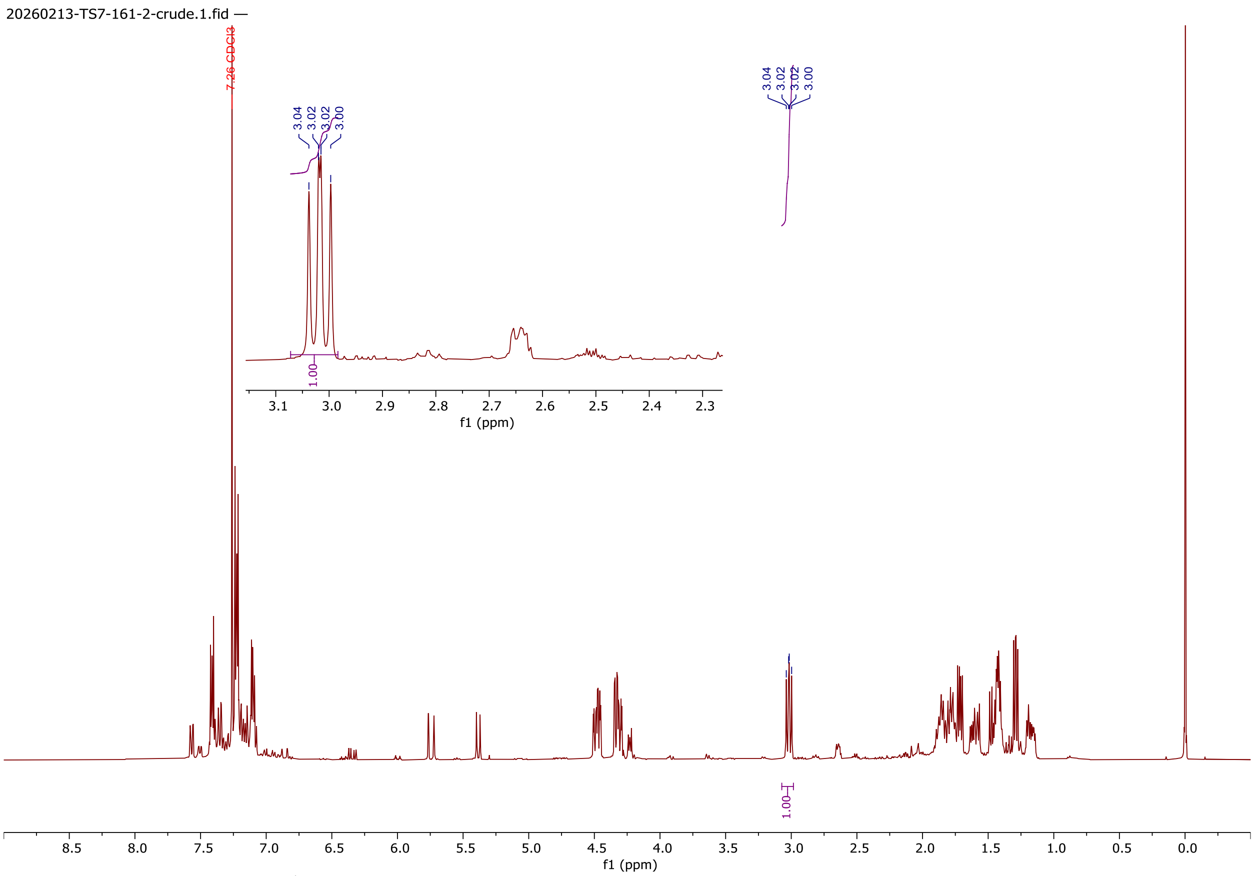

SI Figure S12. Crude ^{1}H NMR of reaction (w/ Rh_2(*S*-*p*-PhTPCP)$_4$) with diazo **13**, derived from ε-caprolactone.

Signal at 3.02 (dd, J = 9.1, 7.2 Hz, 1H) was assigned as the benzylic proton of the *E*-diastereomer of the cyclopropanation product (**14b**).
Unable to distinguish benzylic proton of *Z*-diastereomer. Presumed to not be present.

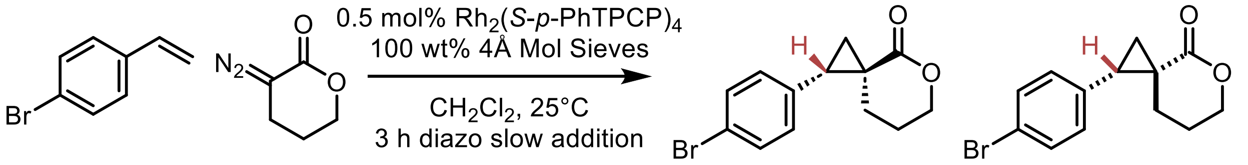

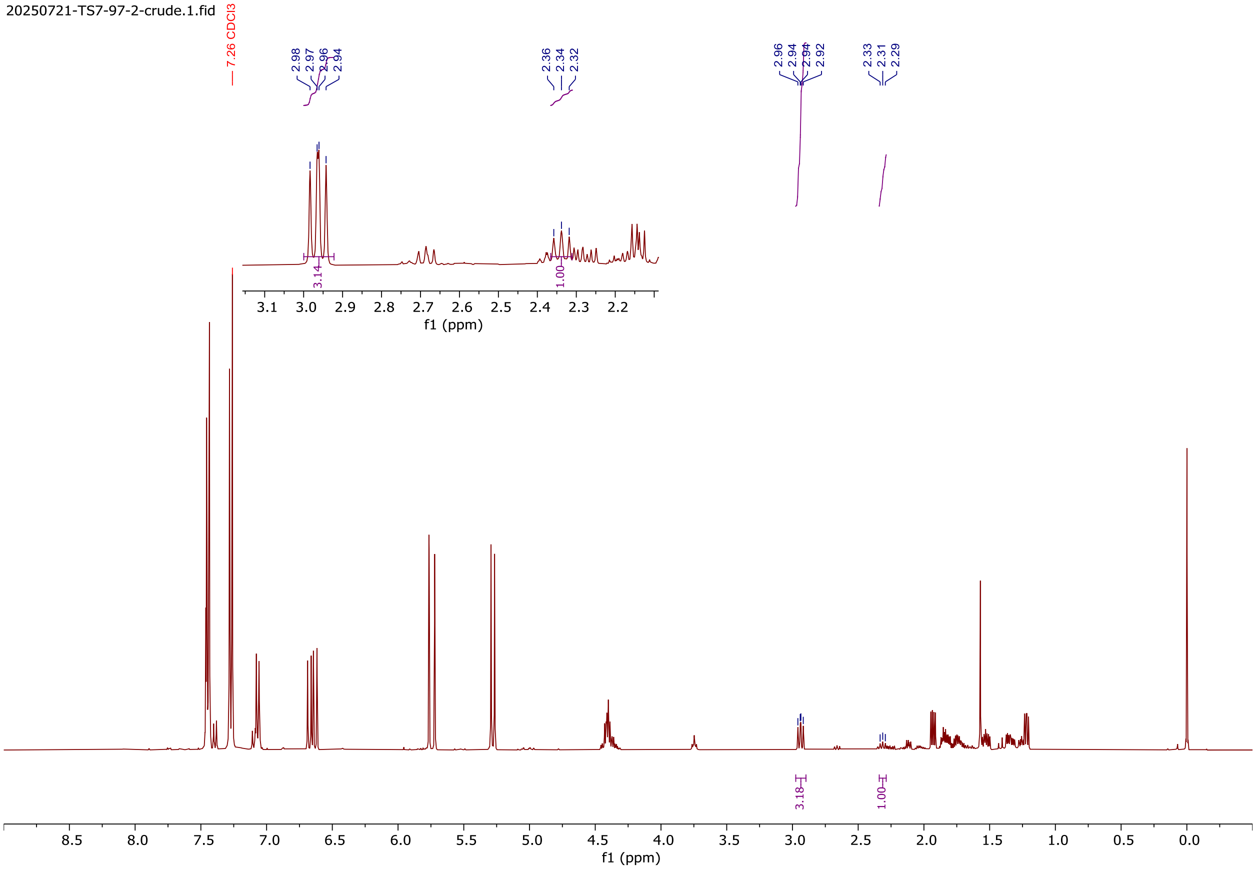

SI Figure S13. Diastereoselectivity of substrate **16a**

Signal at 2.94 (dd, J = 9.2, 7.3 Hz, 1H) was assigned as the benzylic proton of the *E*-diastereomer
Signal at 2.34-2.29 (m, 1H) was assigned as the benzylic proton of the *Z*-diastereomer

Rh_2(*S*-*p*-PhTPCP)$_4$: 3:1

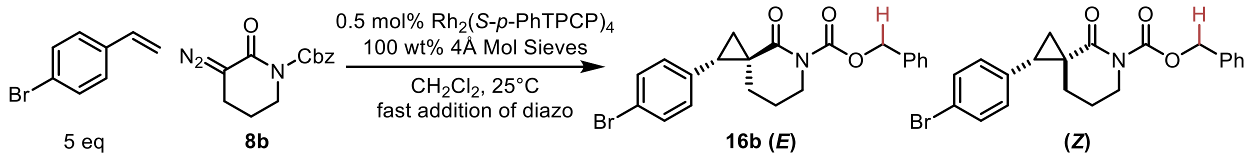

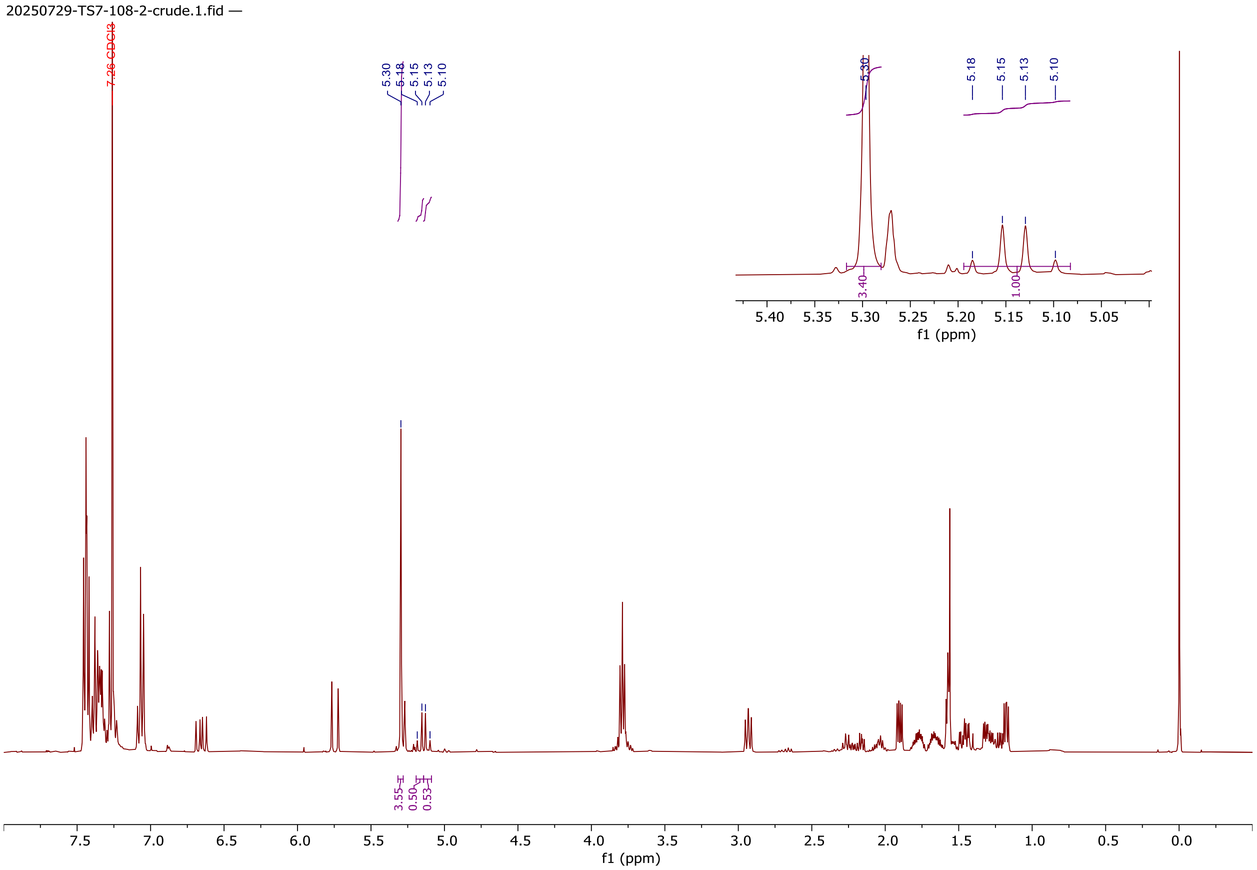

SI Figure S14. Diastereoselectivity of substrate **16b**

Signal at 5.30 (s, 2H) was assigned as the two benzylic protons of the Cbz group in the *E*-diastereomer

Signal at 5.17 (d, J = 12.5 Hz, 1H) was assigned as one of the benzylic protons of the Cbz group of the *Z*-diastereomer

Rh_2(*S*-*p*-PhTPCP)$_4$: 3:1

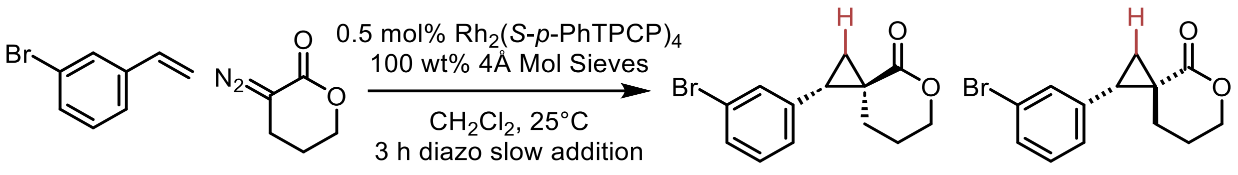

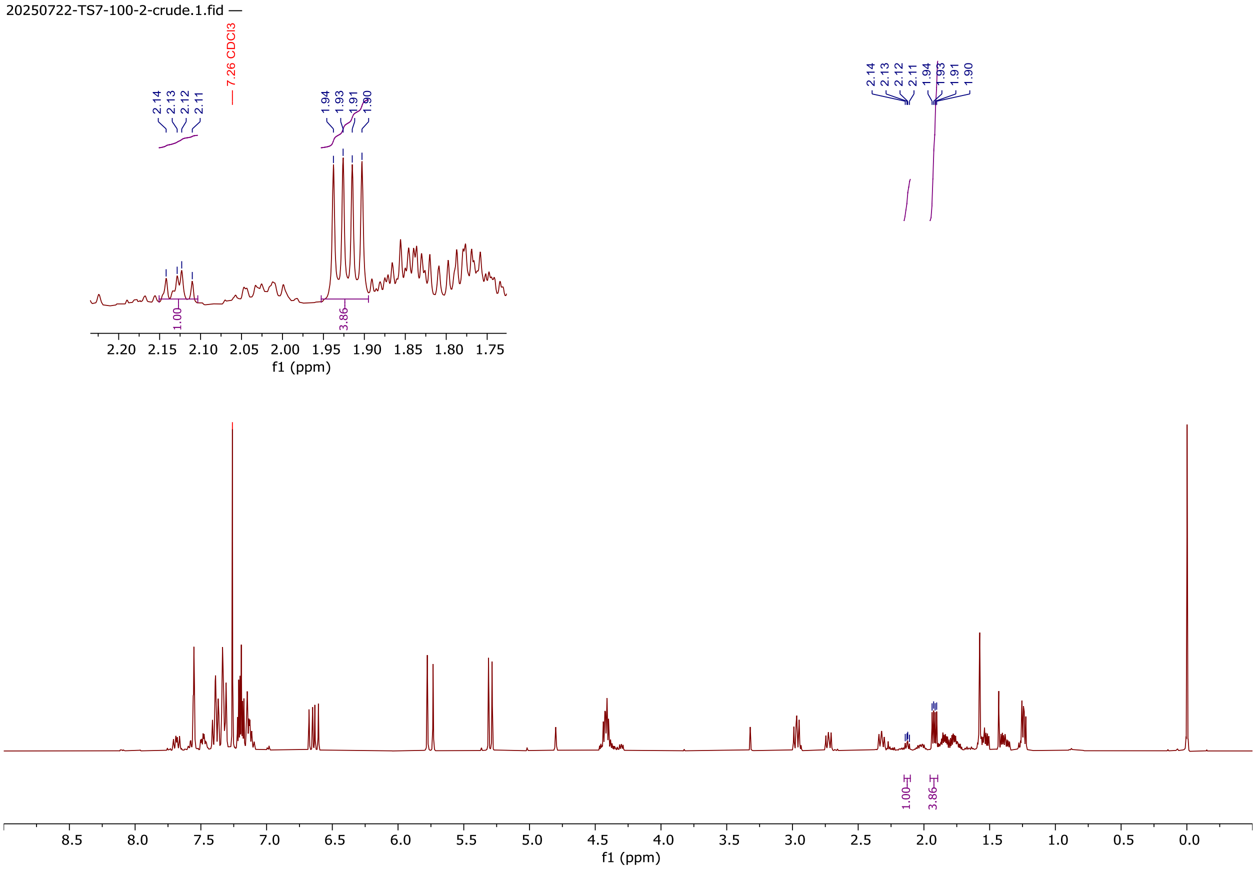

SI Figure S15. Diastereoselectivity of substrate **17a**

Signal at 1.92 (dd, J = 9.2, 4.7 Hz, 1H) was assigned as one of the cyclopropane protons of the E-diastereomer
Signal at 2.13 (dd, J = 7.5, 5.3 Hz, 1H) was assigned as one of the cyclopropane protons of the Z-diastereomer

Rh_2(S-p-PhTPCP)$_4$: 4:1

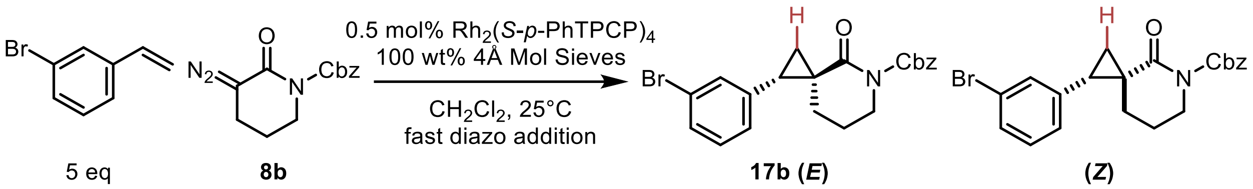

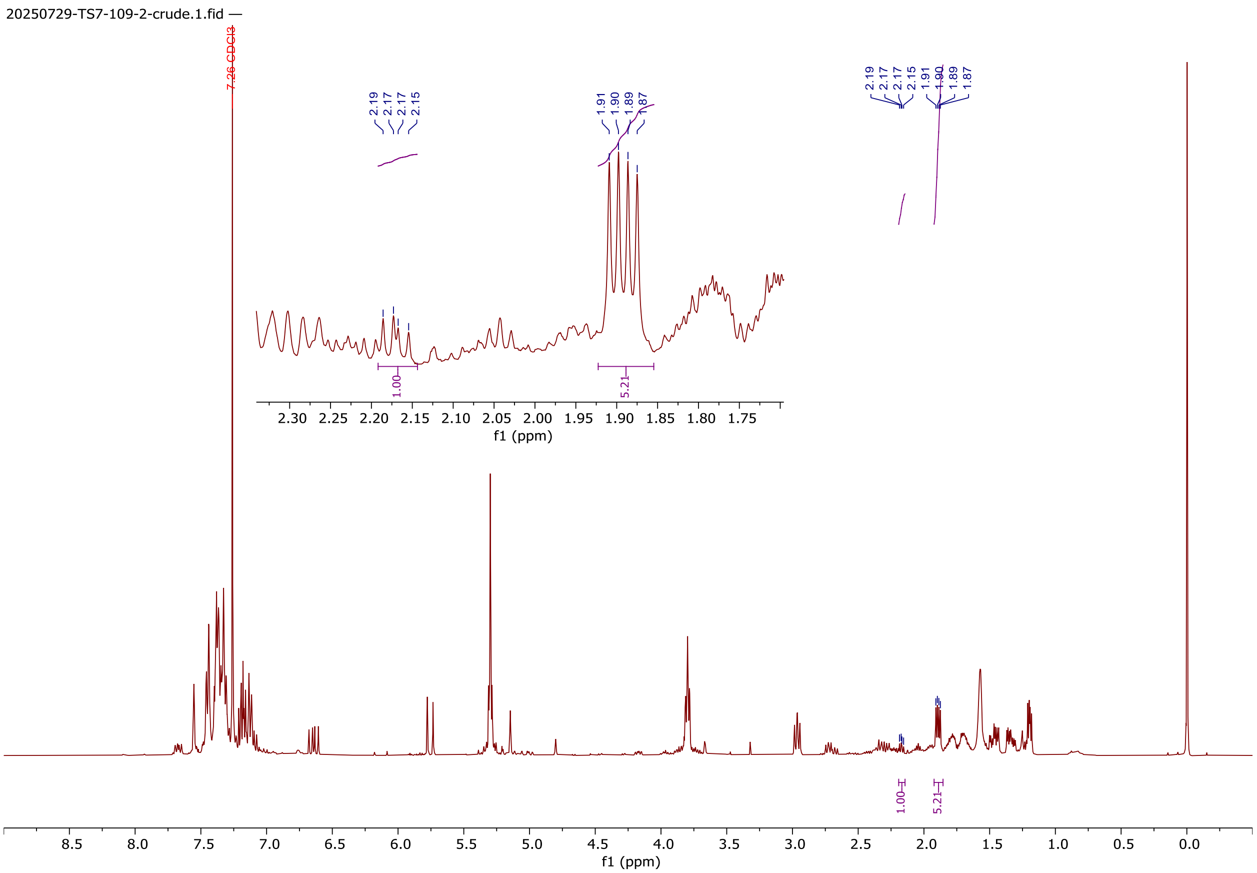

SI Figure S16. Diastereoselectivity of substrate **17b**

Signal at 1.89 (dd, J = 9.2, 4.5 Hz, 1H) was assigned as one of the cyclopropane protons of the *E*-diastereomer
Signal at 2.17 (dd, J = 7.4, 5.1 Hz, 1H) was assigned as one of the cyclopropane protons of the *Z*-diastereomer

Rh_2(*S*-*p*-PhTPCP)$_4$: 5:1

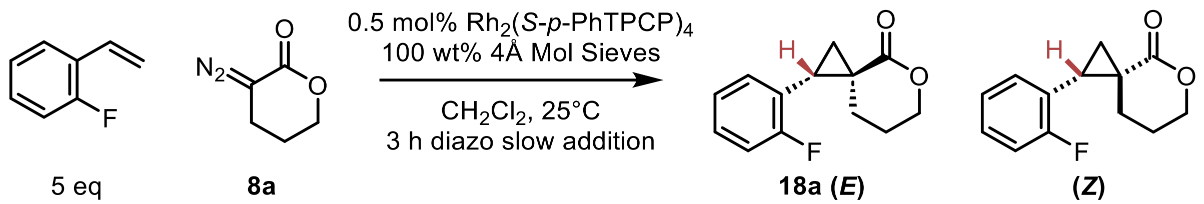

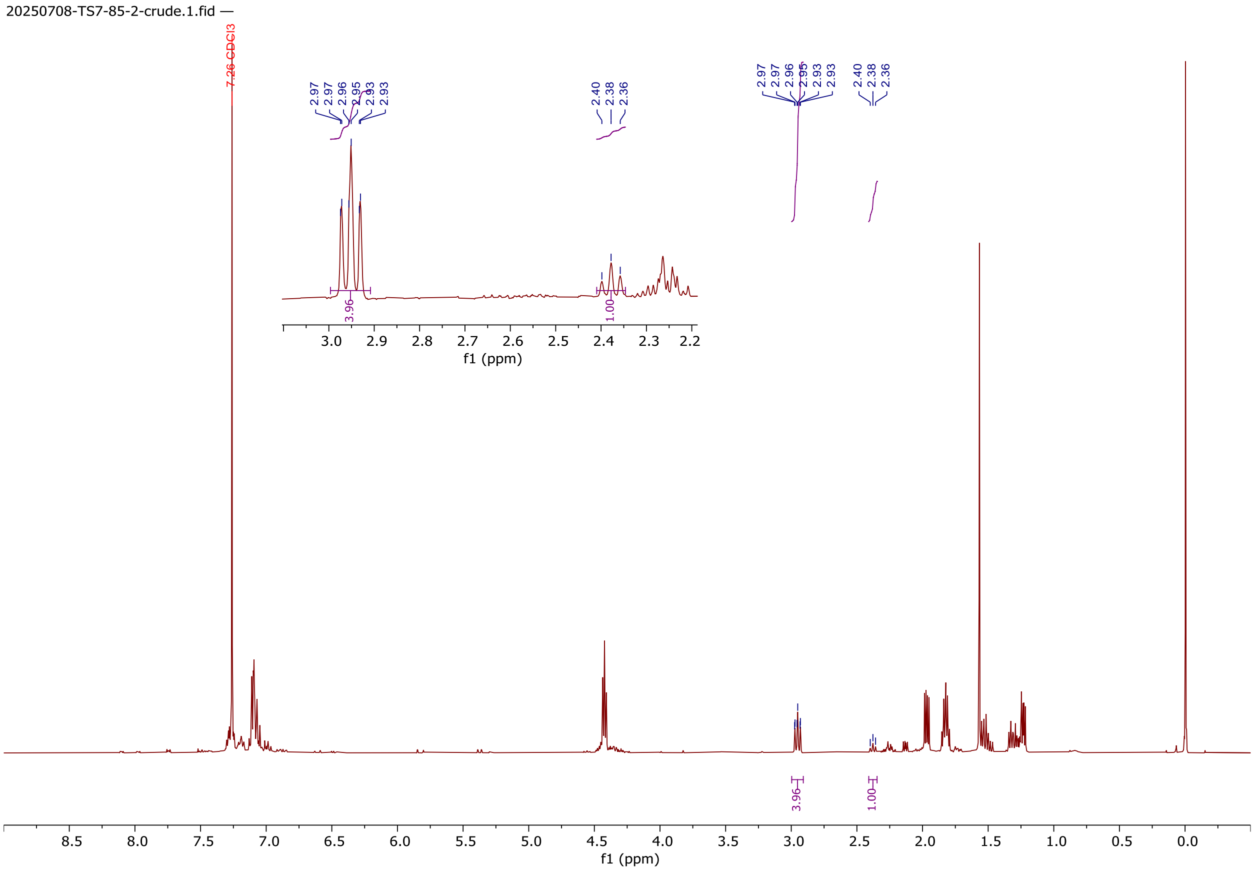

SI Figure S17. Diastereoselectivity of substrate **18a**

Signal at 2.95 (dd, J = 9.3, 7.2 Hz, 1H) was assigned as the benzylic proton of the *E*-diastereomer
Signal at 2.38 (app t, J = 8.1 Hz, 1H) was assigned as the benzylic proton of the *Z*-diastereomer

Rh_2(*S*-*p*-PhTPCP)$_4$: 4:1

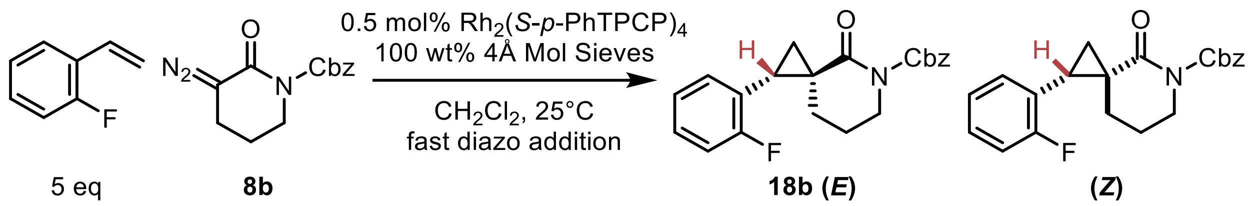

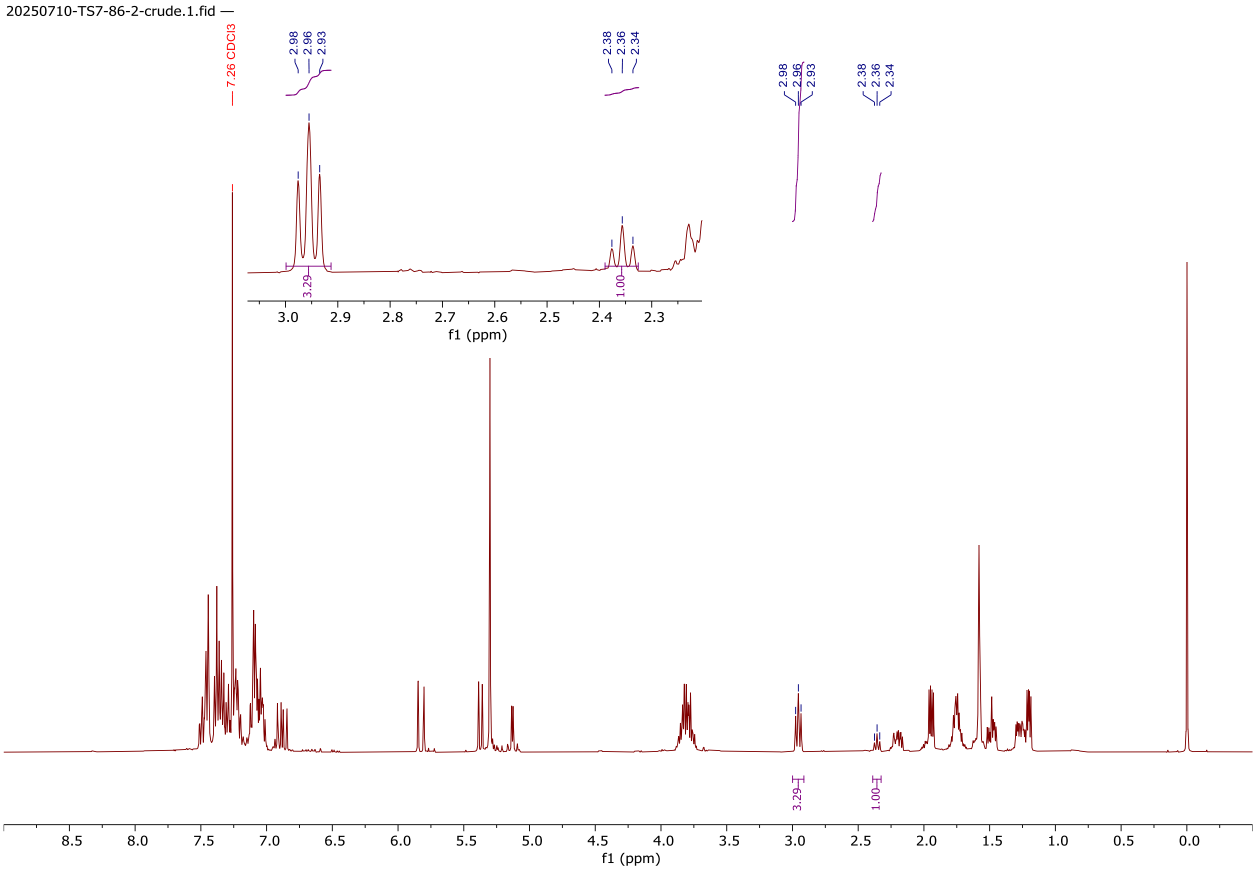

SI Figure S18. Diastereoselectivity of substrate **18b**

Signal at 2.96 (app t, J = 8.2 Hz, 1H) was assigned as the benzylic proton of the *E*-diastereomer
Signal at 2.36 (app t, J = 8.0 Hz, 1H) was assigned as the benzylic proton of the *Z*-diastereomer

Rh_2(*S*-*p*-PhTPCP)$_4$: 3:1

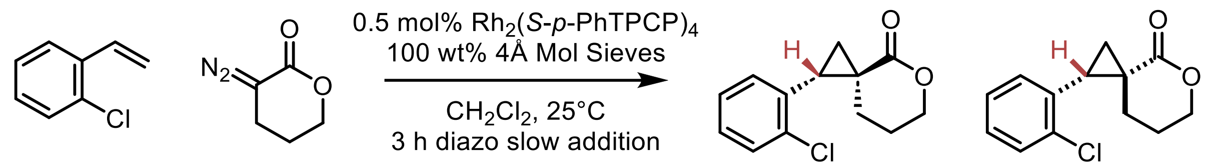

5 eq | **8a** | **19a (*E*)** | **(*Z*)**

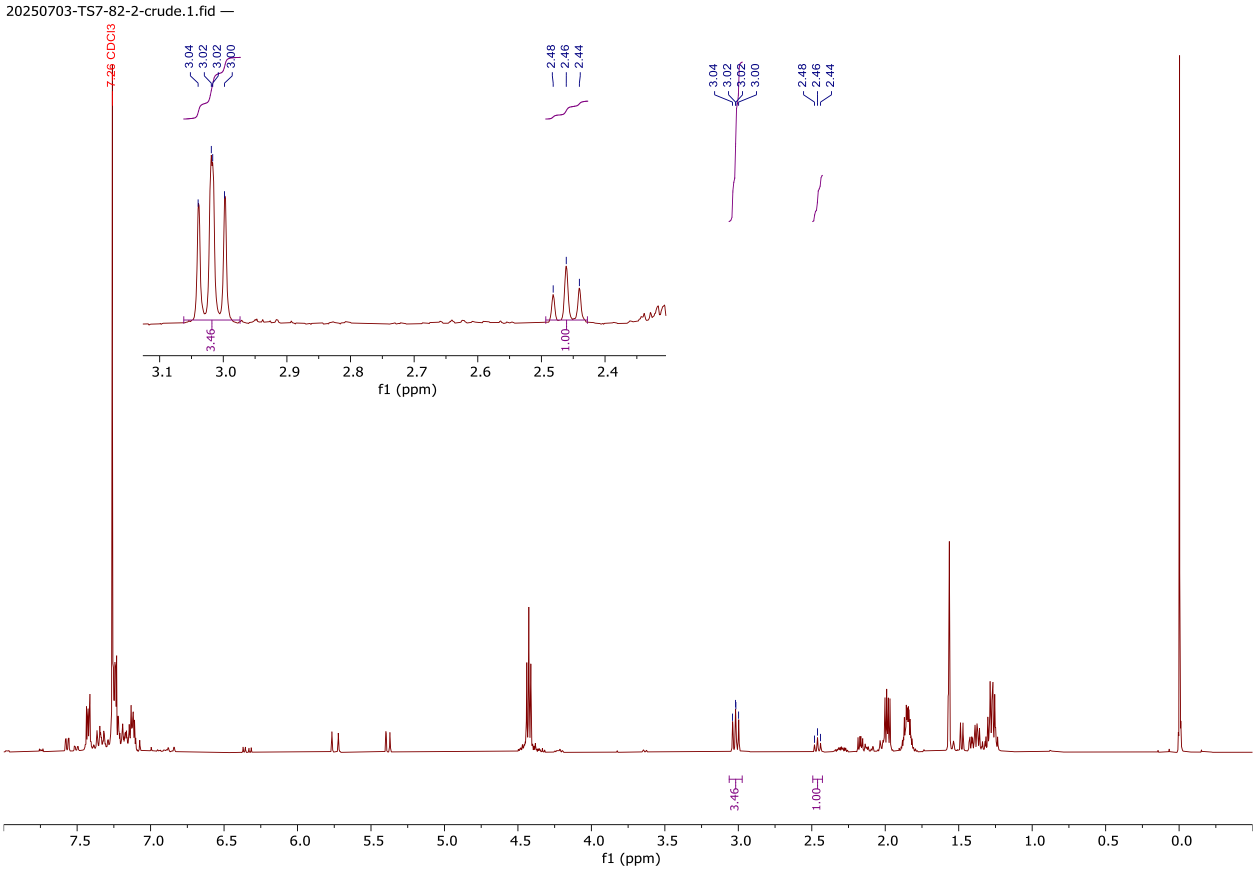

SI Figure S19. Diastereoselectivity of substrate **19a**

Signal at 3.02 (dd, J = 8.7, 7.9 Hz, 1H) was assigned as the benzylic proton of the E-diastereomer
Signal at 2.46 (app t, J = 8.2 Hz, 1H) was assigned as the benzylic proton of the Z-diastereomer

Rh_2(S-p-PhTPCP)$_4$: 3:1

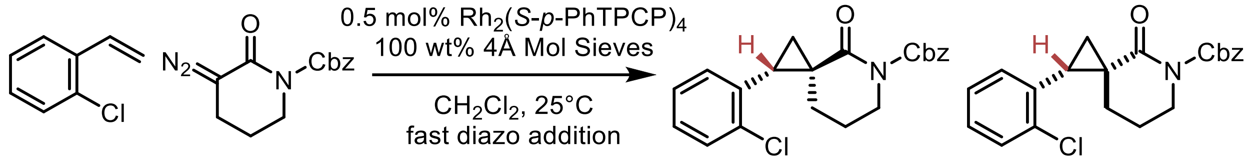

5 eq **8b** **19b (*E*)** **(*Z*)**

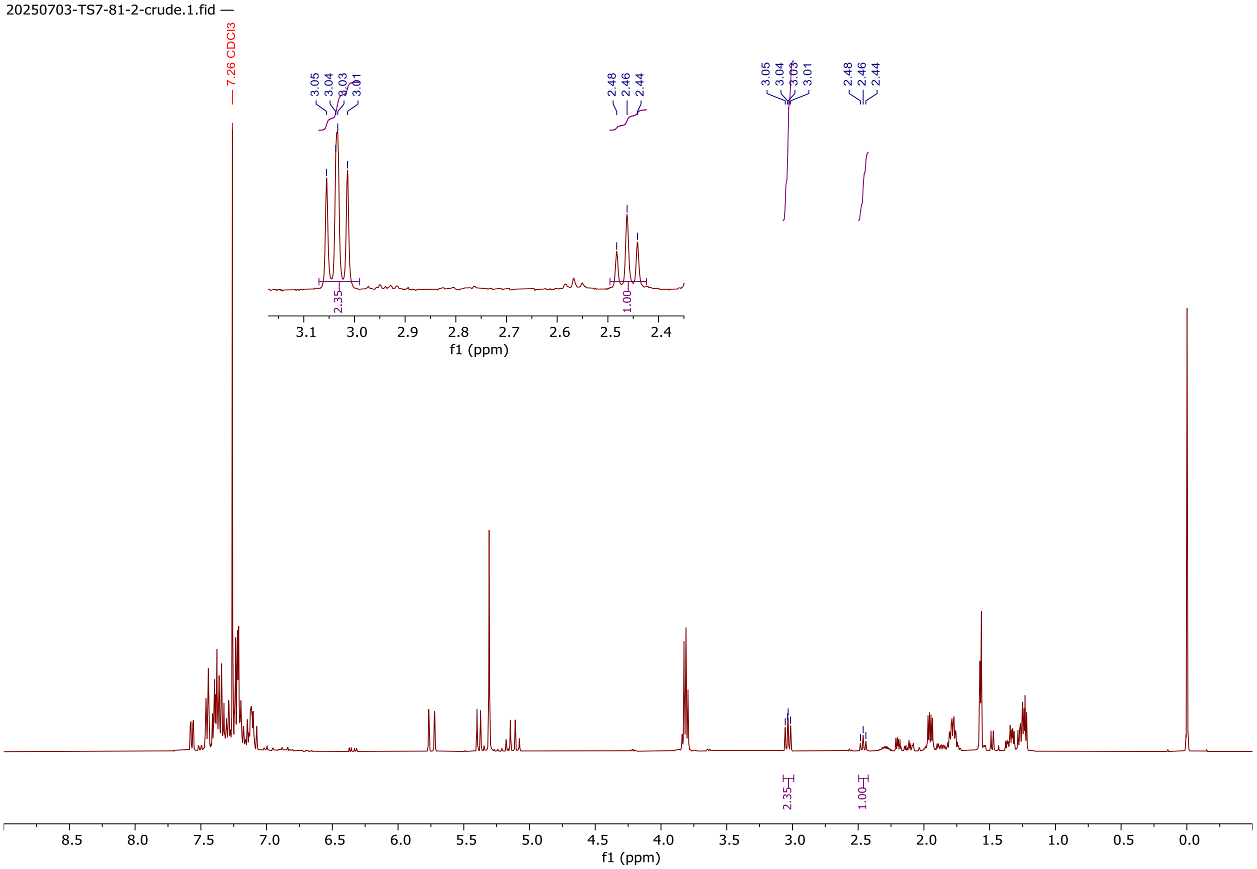

SI Figure S20. Diastereoselectivity of substrate **19b**

Signal at 3.03 (dd, $J = 9.0, 7.4$ Hz, 1H) was assigned as the benzylic proton of the *E*-diastereomer
Signal at 2.46 (app t, $J = 8.2$ Hz, 1H) was assigned as the benzylic proton of the *Z*-diastereomer

Rh_2(*S*-*p*-PhTPCP)$_4$: 3:1

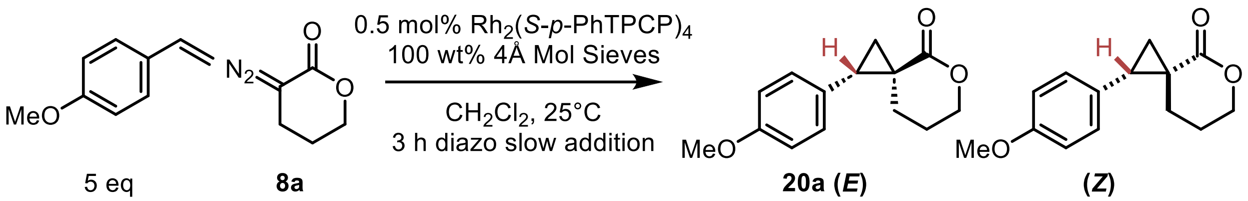

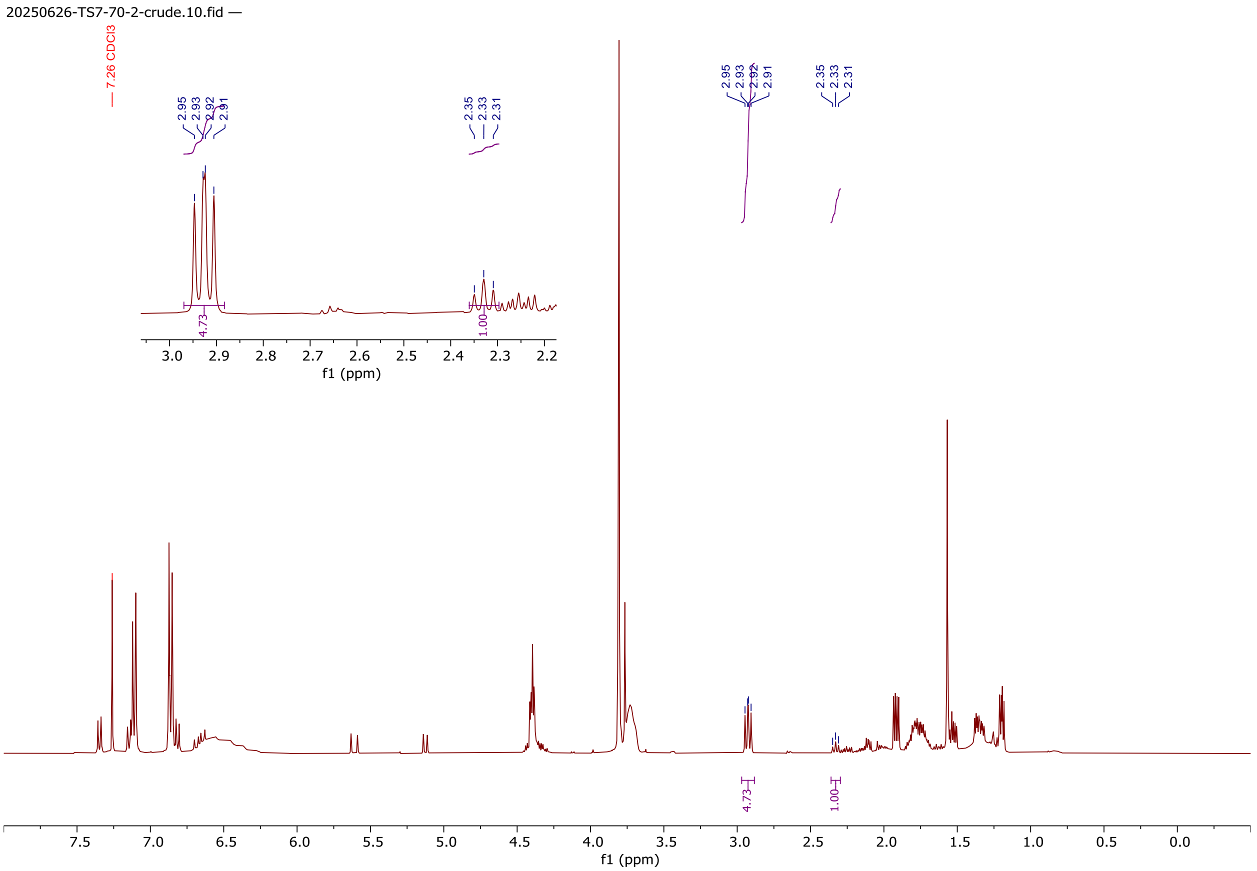

SI Figure S21. Diastereoselectivity of substrate **20a**

Signal at 2.93 (dd, $J = 9.3, 7.3$ Hz, 1H) was assigned as the benzylic proton of the *E*-diastereomer
Signal at 2.33 (app t, $J = 8.1$ Hz, 1H) was assigned as the benzylic proton of the *Z*-diastereomer

Rh_2(*S*-*p*-PhTPCP)$_4$: 5:1

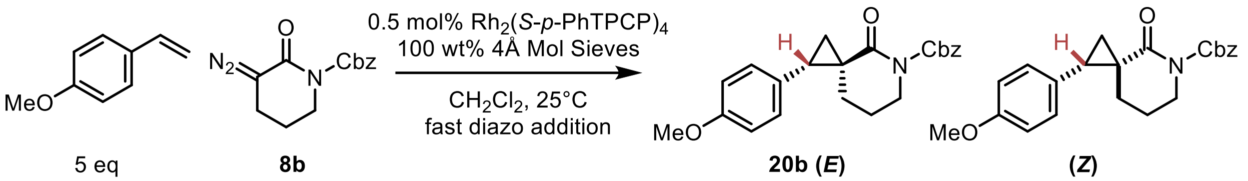

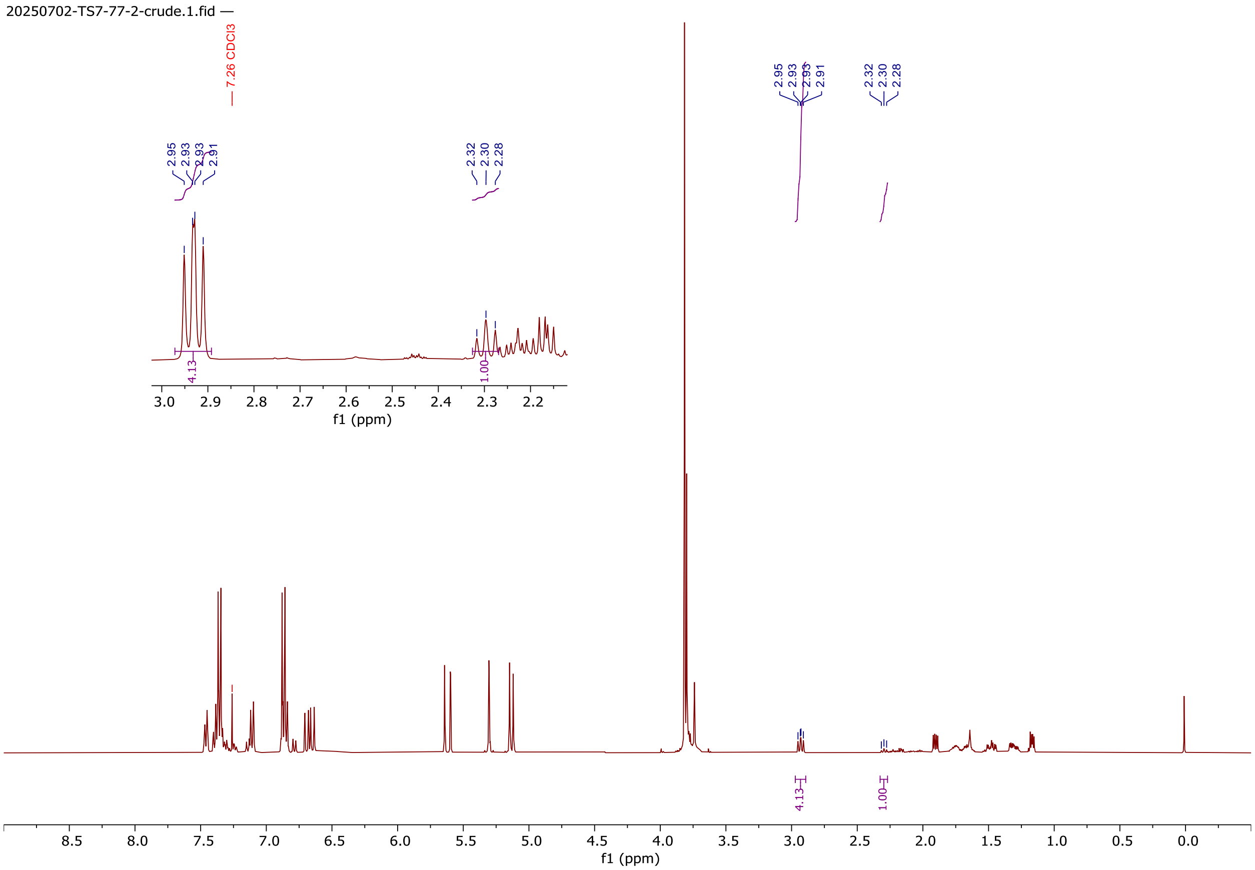

SI Figure S22. Diastereoselectivity of substrate **20b**

Signal at 2.93 (dd, $J = 9.2, 7.2$ Hz, 1H) was assigned as the benzylic proton of the *E*-diastereomer
Signal at 2.30 (app t, $J = 8.0$ Hz, 1H) was assigned as the benzylic proton of the *Z*-diastereomer

Rh_2(*S*-*p*-PhTPCP)$_4$: 4:1

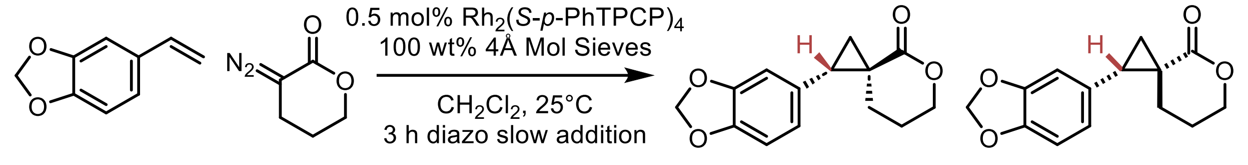

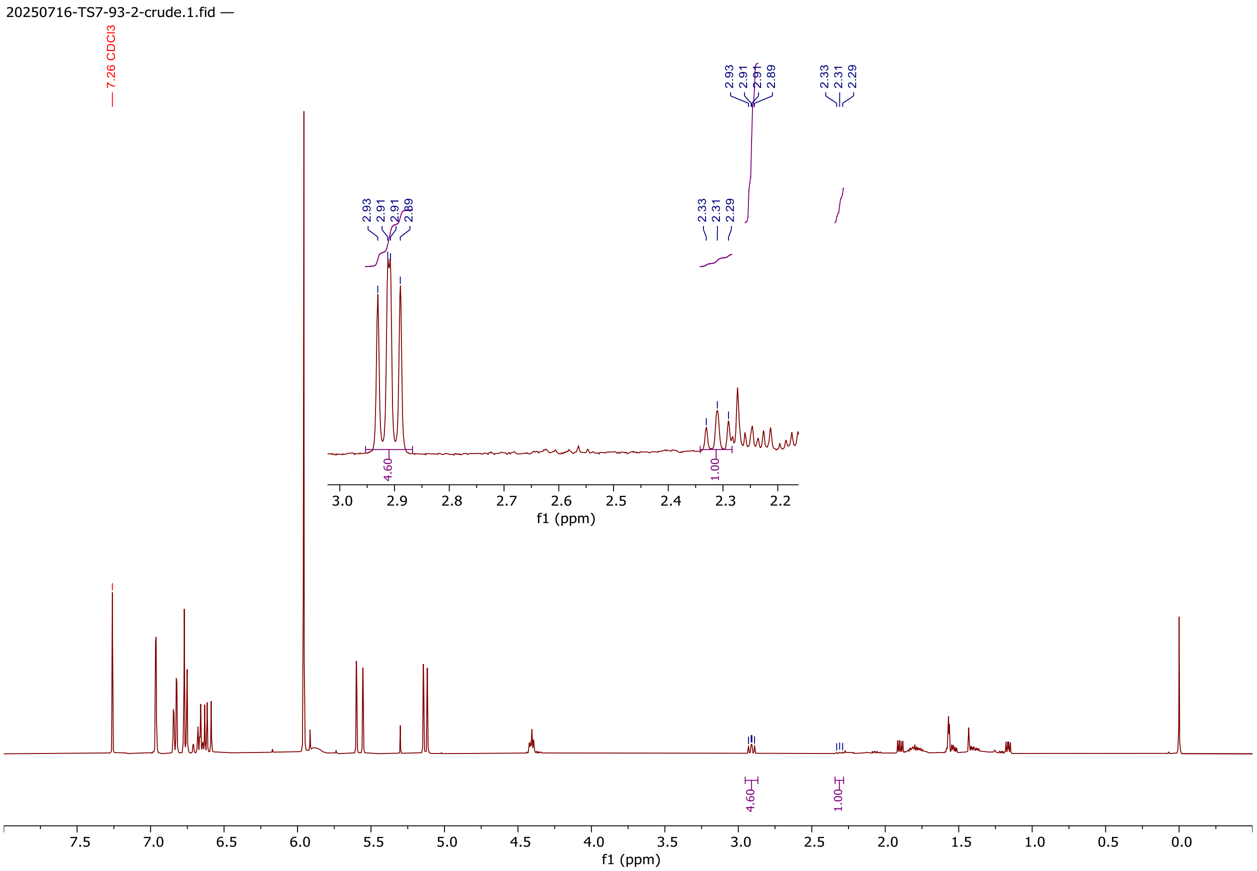

SI Figure S23. Diastereoselectivity of substrate **21a**

Signal at 2.91 (dd, J = 9.2, 7.2 Hz, 1H) was assigned as the benzylic proton of the E-diastereomer
Signal at 2.31 (app t, J = 8.1 Hz, 1H) was assigned as the benzylic proton of the Z-diastereomer

Rh_2(S-p-PhTPCP)$_4$: 5:1

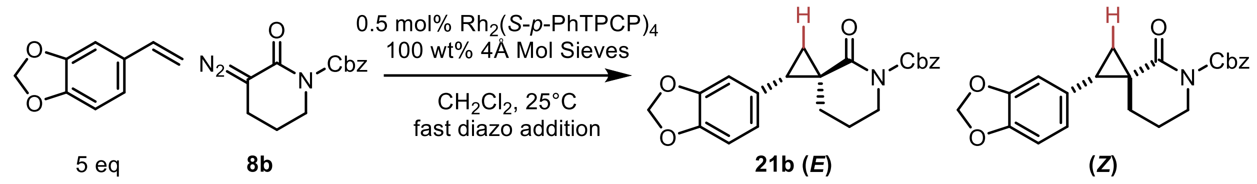

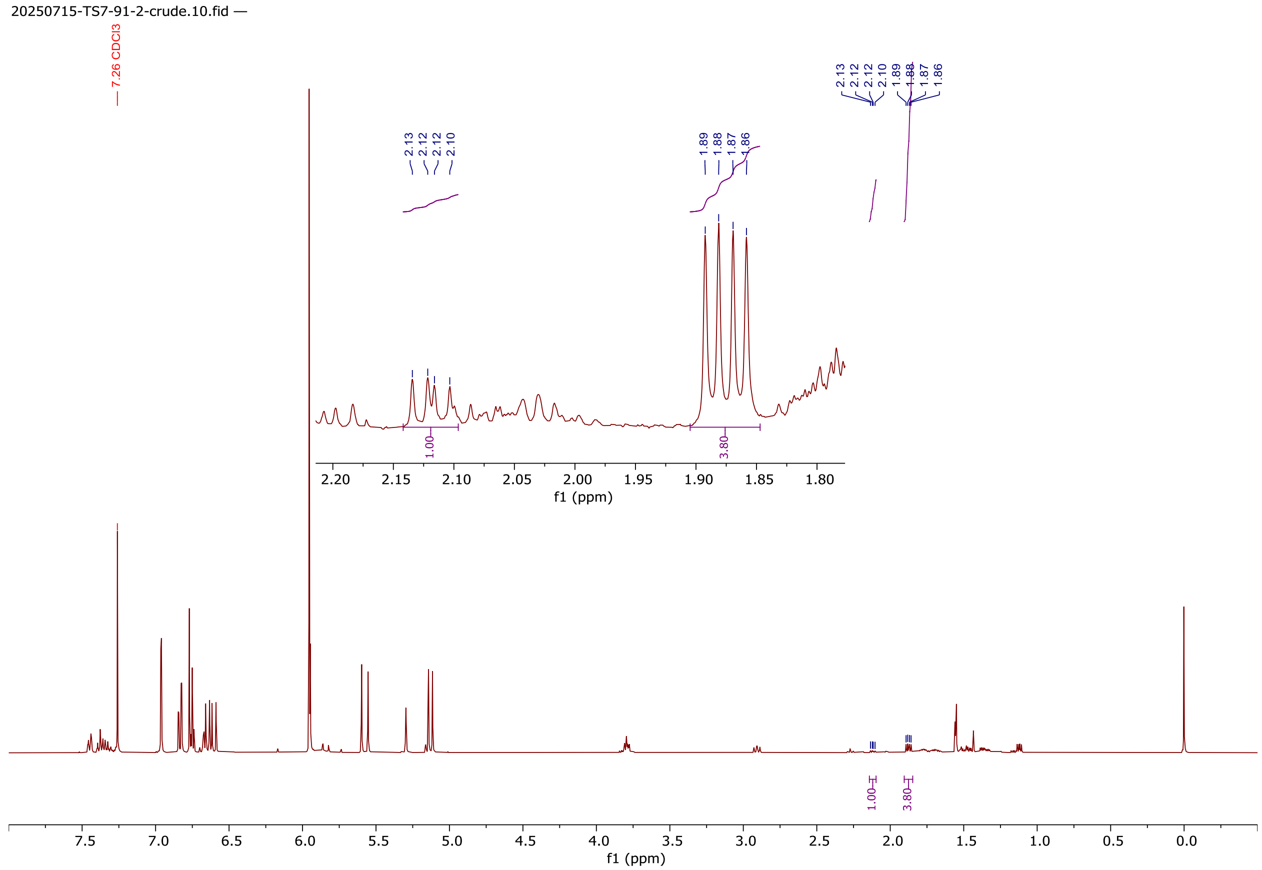

SI Figure S24. Diastereoselectivity of substrate **21b**

Signal at 1.88 (dd, $J = 9.2, 4.4$ Hz, 1H) was assigned as one of the cyclopropane protons of the *E*-diastereomer
Signal at 2.12 (dd, $J = 7.3, 5.1$ Hz, 1H) was assigned as one of the cyclopropane protons of the *Z*-diastereomer

Rh_2(*S*-*p*-PhTPCP)$_4$: 4:1

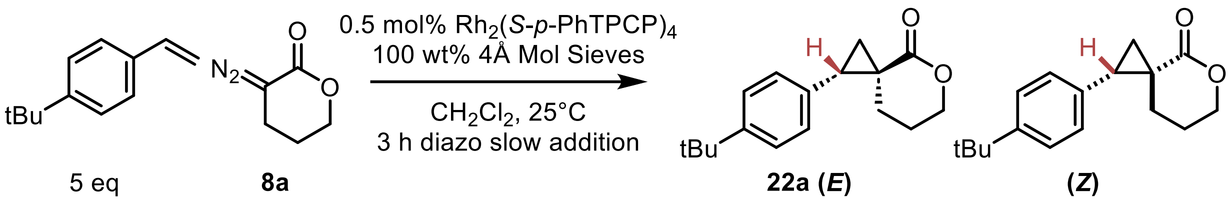

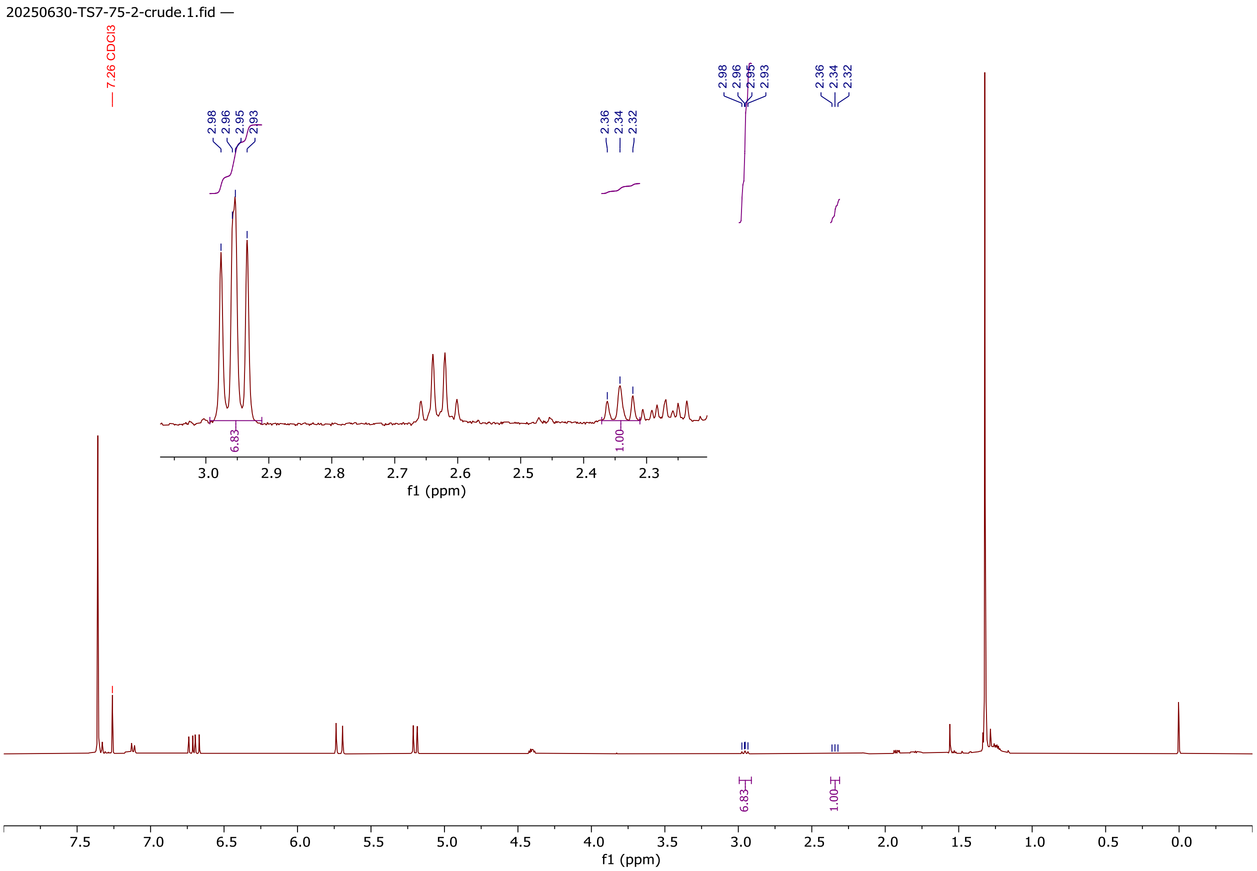

SI Figure S25. Diastereoselectivity of substrate **22a**

Signal at 2.96 (dd, $J = 9.2, 7.2$ Hz, 1H) was assigned as the benzylic proton of the *E*-diastereomer
Signal at 2.34 (app t, $J = 8.1$ Hz, 1H) was assigned as the benzylic proton of the *Z*-diastereomer

Rh_2(*S*-*p*-PhTPCP)$_4$: 7:1

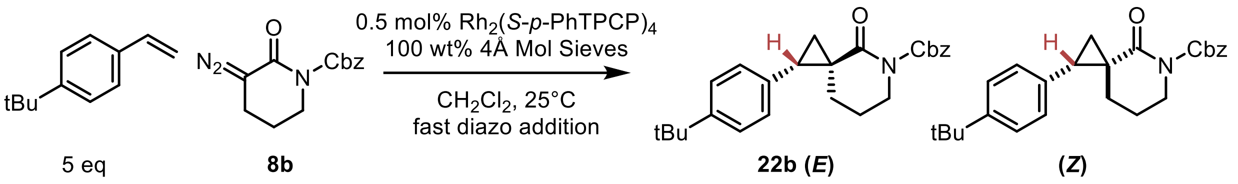

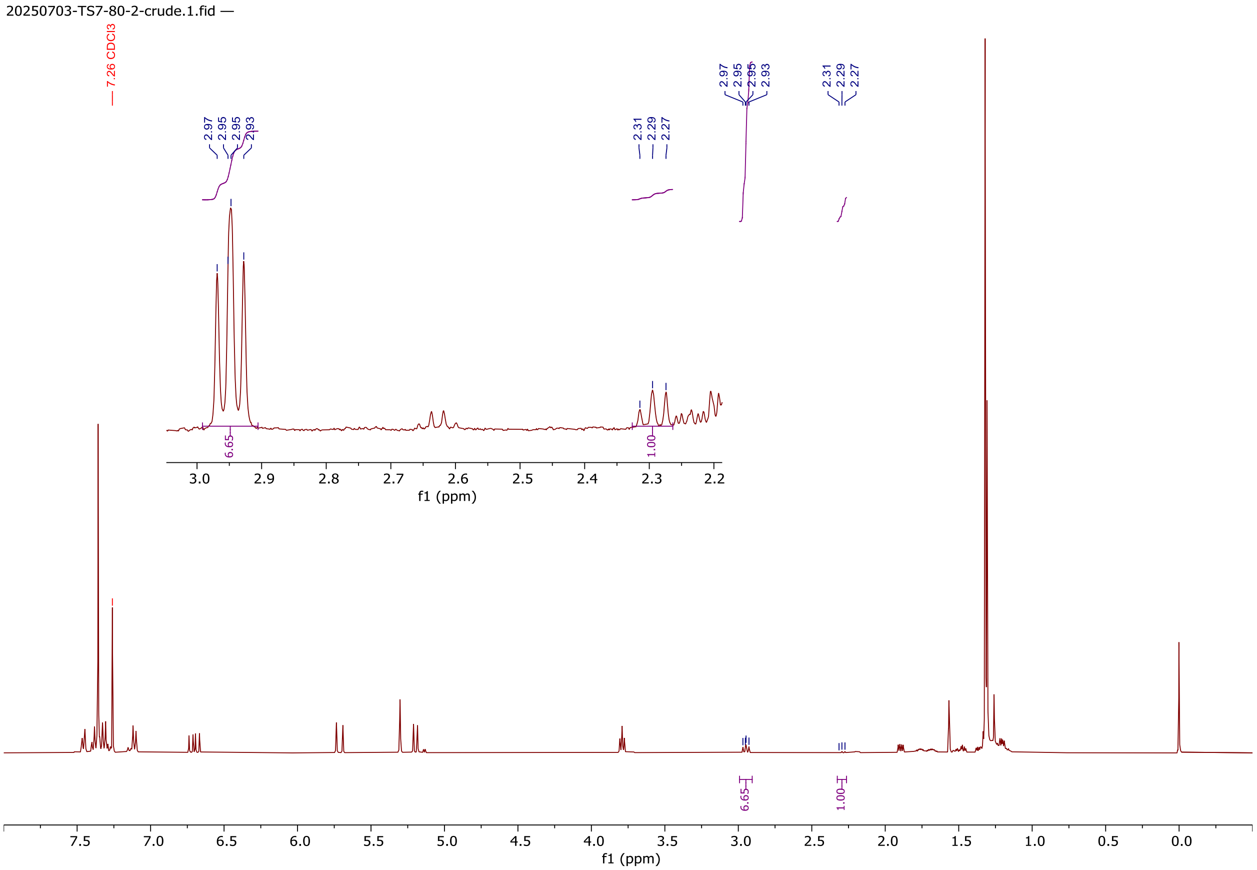

SI Figure S26. Diastereoselectivity of substrate **22b**

Signal at 2.95 (dd, $J = 9.2, 7.3$ Hz, 1H) was assigned as the benzylic proton of the *E*-diastereomer
Signal at 2.33-2.26 (m, 1H) was assigned as the benzylic proton of the *Z*-diastereomer

Rh_2(*S*-*p*-PhTPCP)$_4$: 7:1

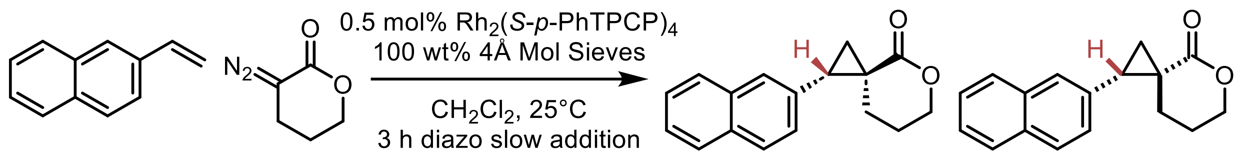

5 eq **8a** **23a (*E*)** **(*Z*)**

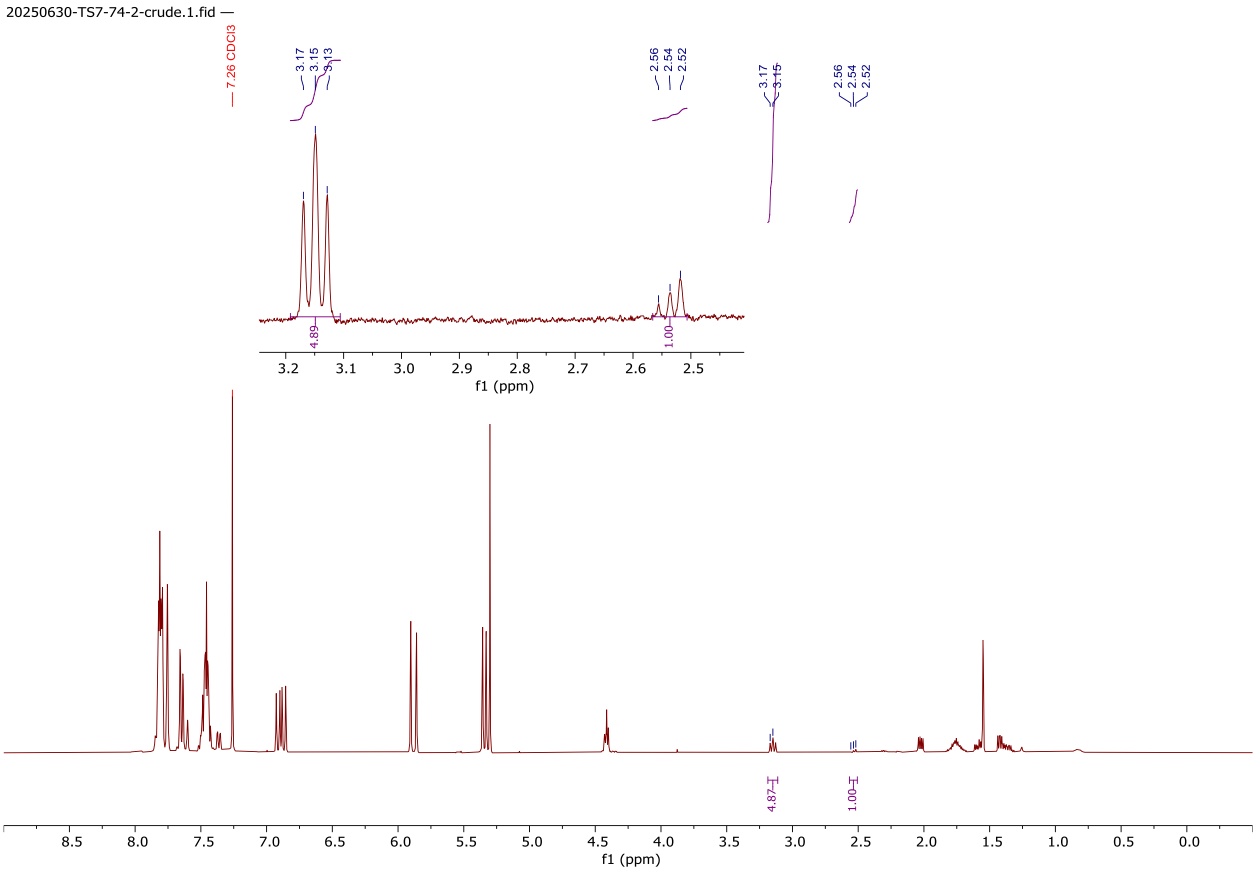

SI Figure S27. Diastereoselectivity of substrate **23a**

Signal at 3.16 (app t, *J* = 8.2 Hz, 1H) was assigned as the benzylic proton of the *E*-diastereomer
Signal at 2.57-2.51 (m, 1H) was assigned as the benzylic proton of the *Z*-diastereomer

Rh$_2$(*S*-*p*-PhTPCP)$_4$: 5:1

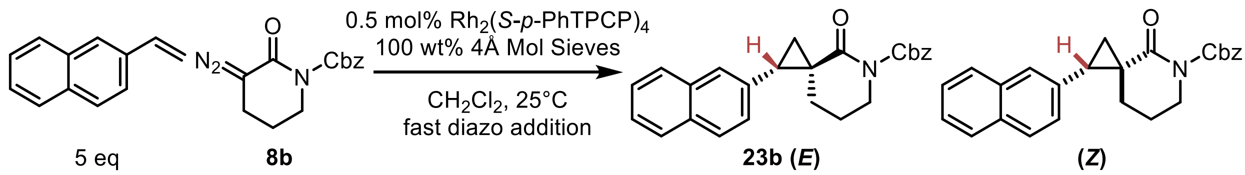

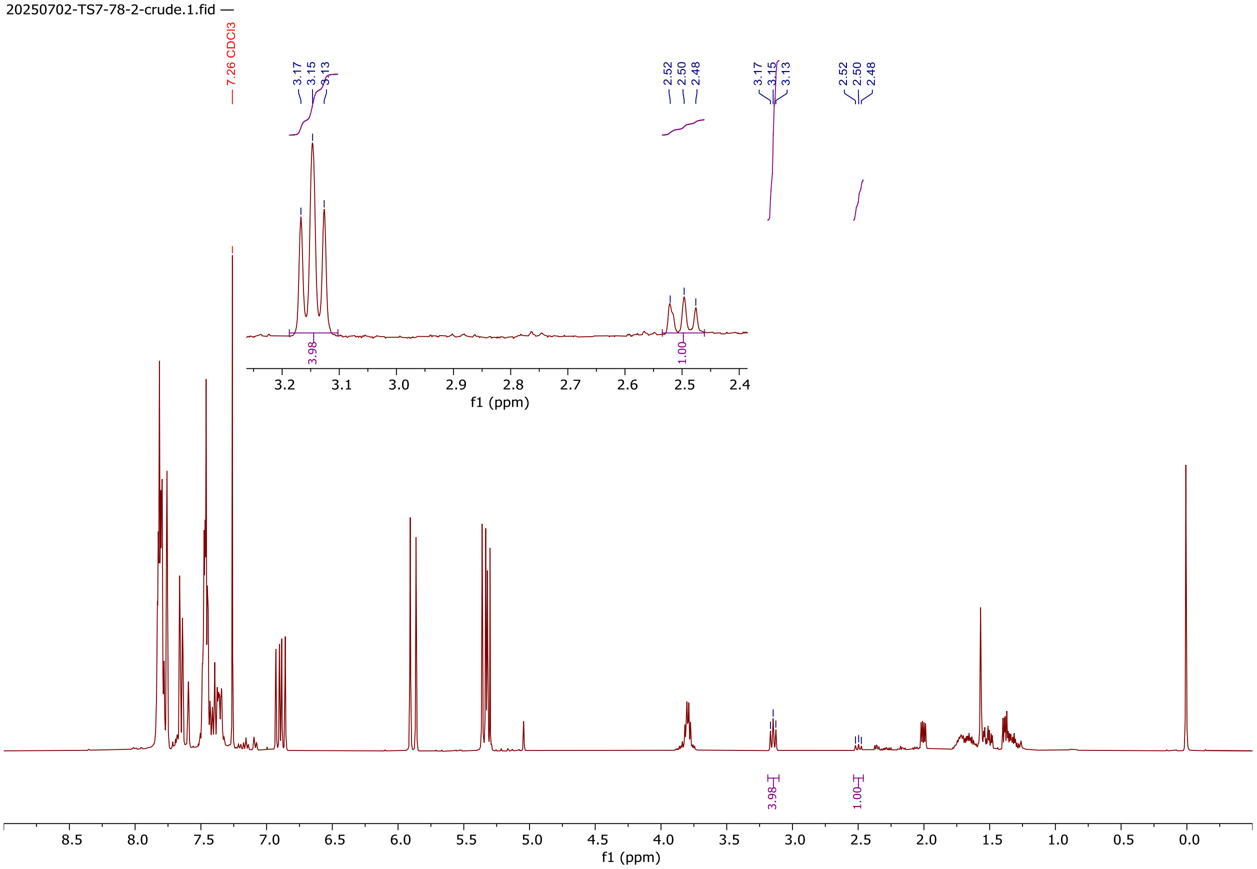

SI Figure S28. Diastereoselectivity of substrate **23b**

Signal at 3.15 (app t, $J = 8.2$ Hz, 1H) was assigned as the benzylic proton of the *E*-diastereomer
Signal at 2.53-2.46 (m, 1H) was assigned as the benzylic proton of the *Z*-diastereomer

Rh_2(*S*-*p*-PhTPCP)$_4$: 4:1

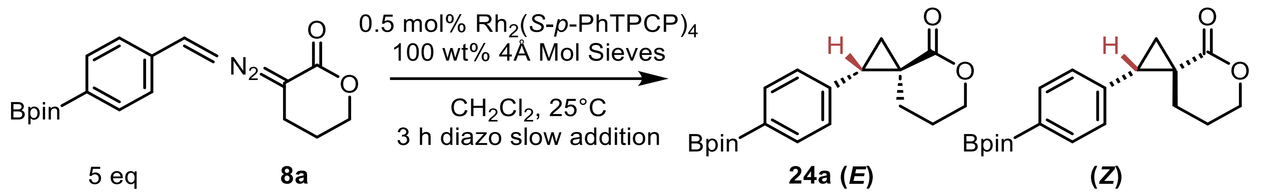

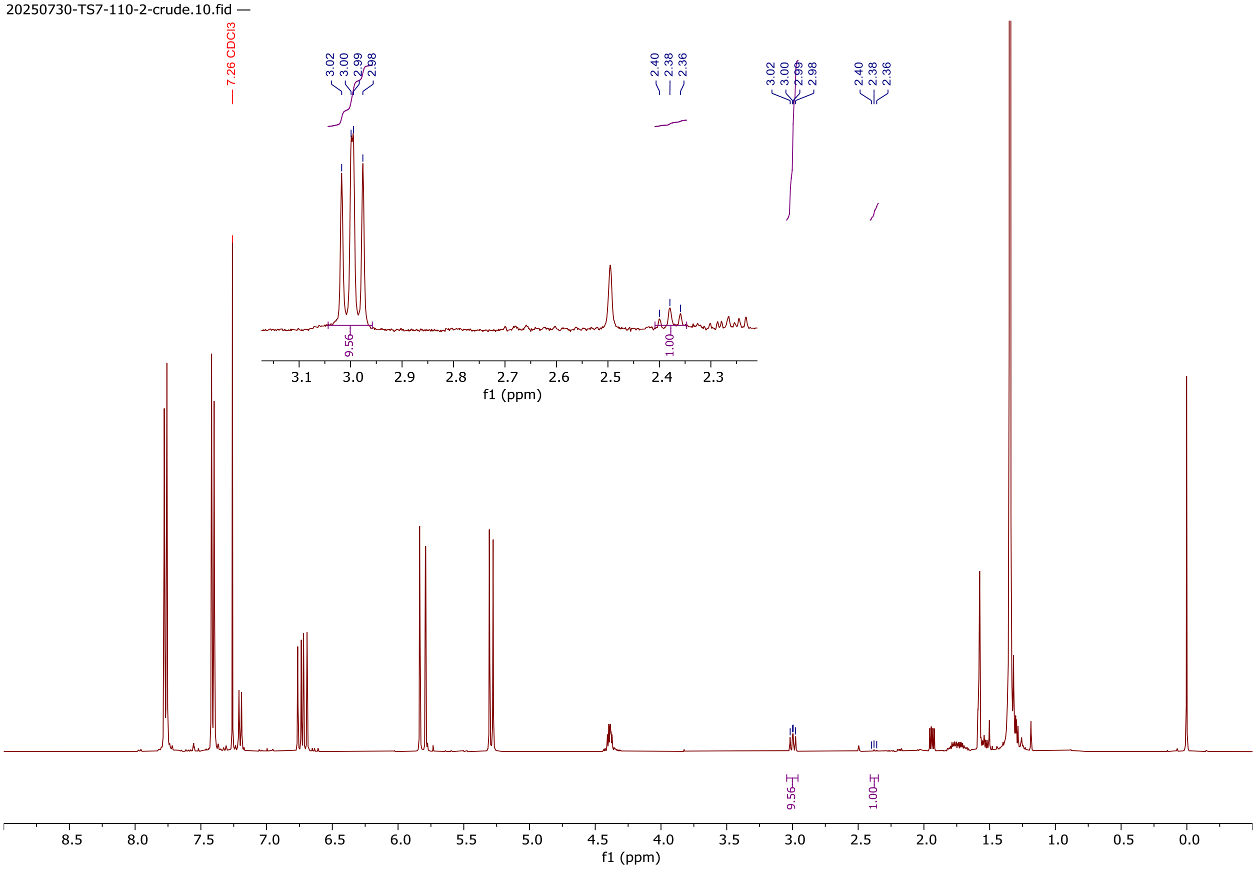

SI Figure S29. Diastereoselectivity of substrate **24a**

Signal at 3.00 (dd, $J = 9.1, 7.3$ Hz, 1H) was assigned as the benzylic proton of the *E*-diastereomer
Signal at 2.38 (app t, $J = 8.1$ Hz, 1H) was assigned as the benzylic proton of the *Z*-diastereomer

Rh_2(*S*-*p*-PhTPCP)$_4$: 10:1

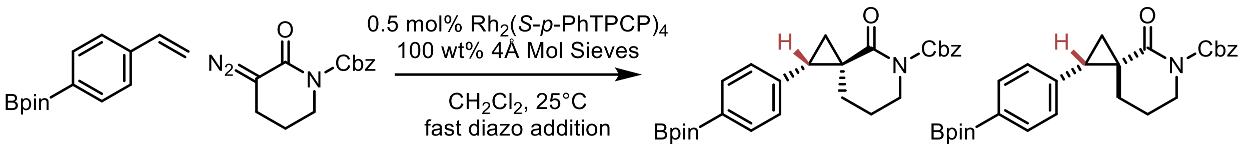

5 eq 8b **24b (*E*)** **(*Z*)**

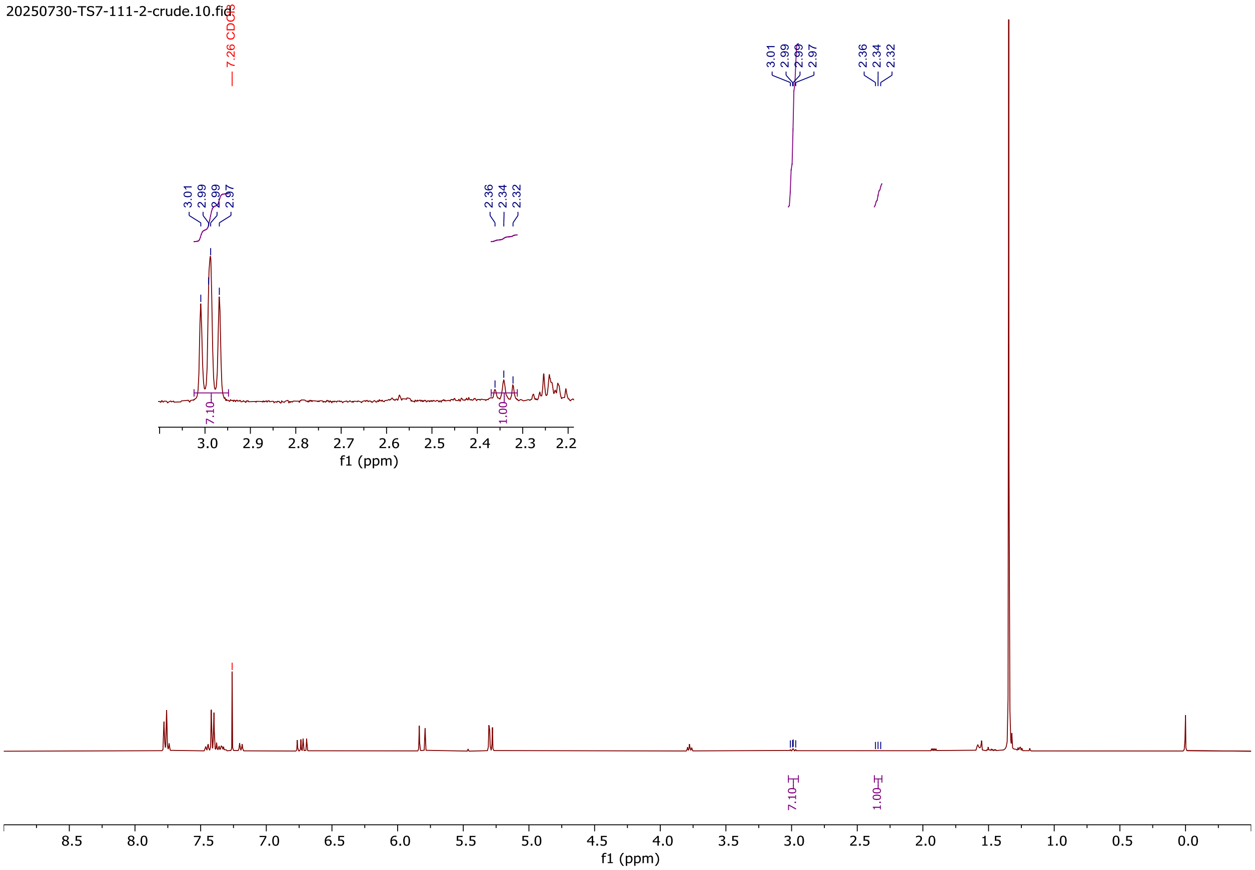

SI Figure S30. Diastereoselectivity of substrate **24b**

Signal at 2.99 (dd, J = 9.1, 7.2 Hz, 1H) was assigned as the benzylic proton of the *E*-diastereomer
Signal at 2.34 (app t, J = 7.9 Hz, 1H) was assigned as the benzylic proton of the *Z*-diastereomer

Rh_2(*S*-*p*-PhTPCP)$_4$: 7:1

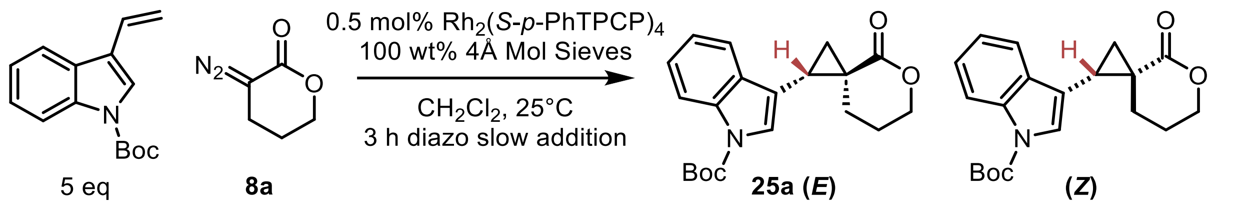

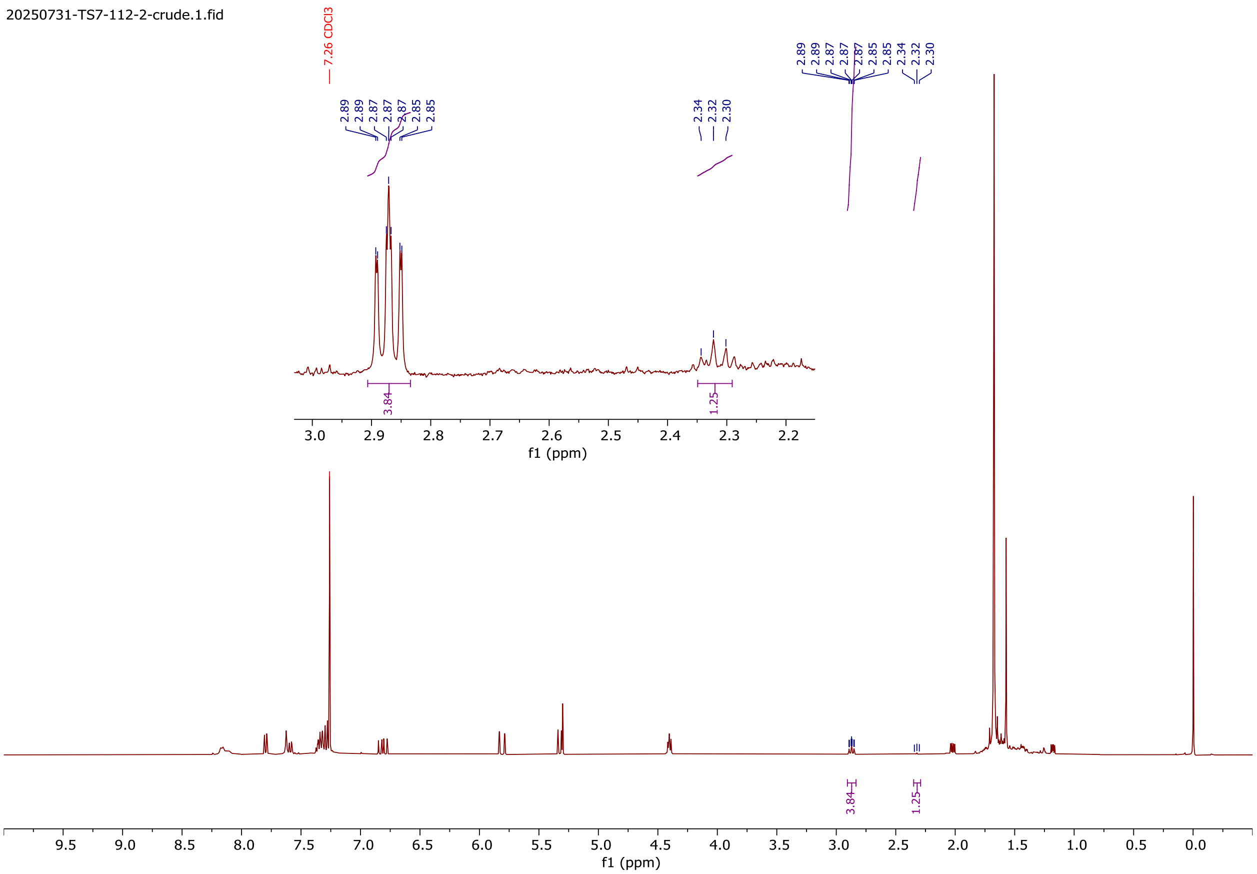

SI Figure S31. Diastereoselectivity of substrate **25a**

Signal at 2.87 (ddd, J = 8.9, 7.2, 1.3 Hz, 1H) was assigned as the benzylic proton of the *E*-diastereomer
Signal at 2.32 (app t, J = 8.4 Hz, 1H) was assigned as the benzylic proton of the *Z*-diastereomer

Rh_2(*S*-*p*-PhTPCP)$_4$: 4:1

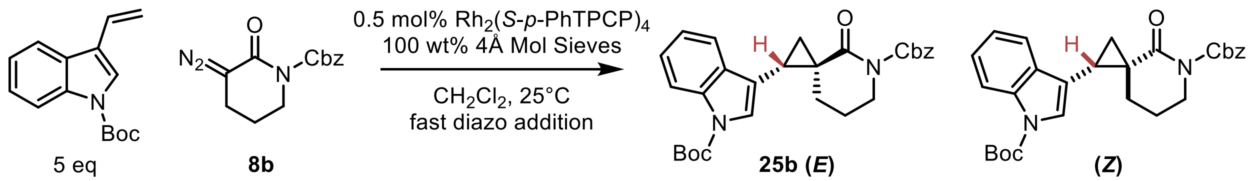

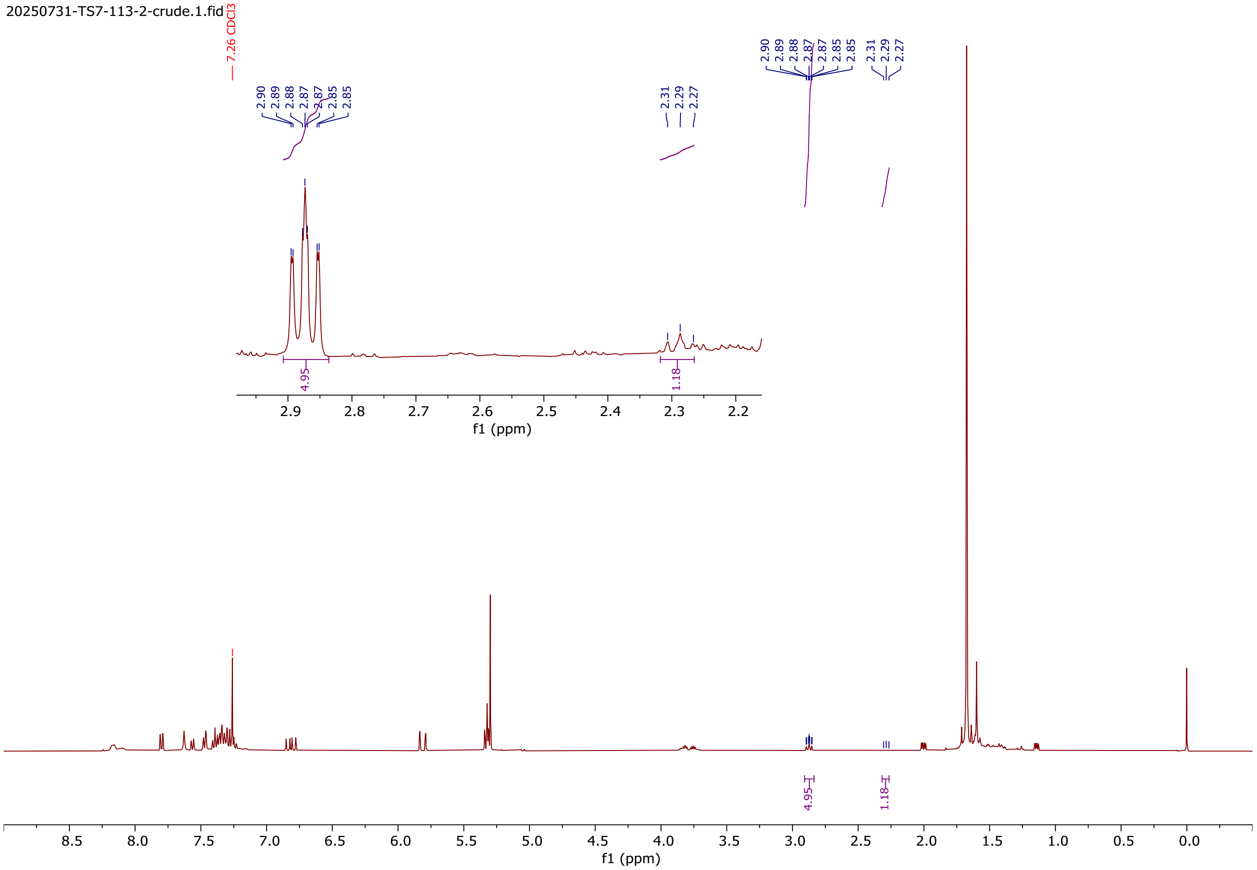

SI Figure S32. Diastereoselectivity of substrate **25b**

Signal at 2.87 (ddd, J = 8.9, 7.2, 1.2 Hz, 1H) was assigned as the benzylic proton of the *E*-diastereomer
Signal at 2.29 (app t, J = 8.1 Hz, 1H) was assigned as the benzylic proton of the *Z*-diastereomer

Rh_2(*S*-*p*-PhTPCP)$_4$: 4:1

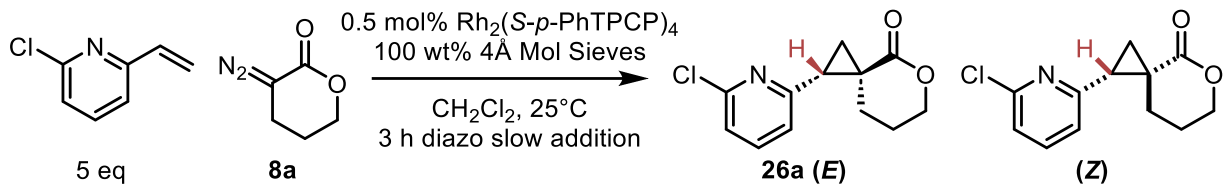

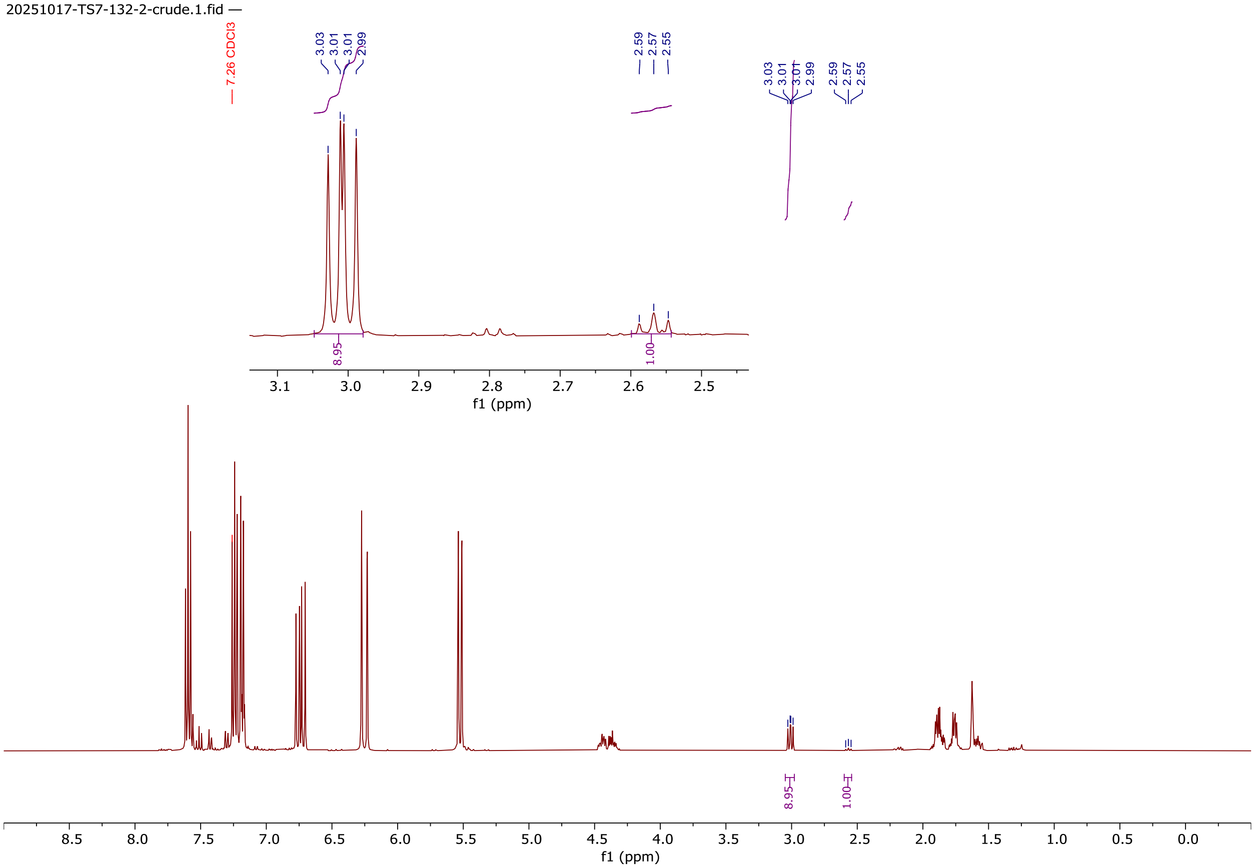

SI Figure S33. Diastereoselectivity of substrate **26a**

Signal at 3.01 (dd, $J = 9.0, 6.9$ Hz, 1H) was assigned as the benzylic proton of the *E*-diastereomer
Signal at 2.57 (app t, $J = 8.2$ Hz, 1H) was assigned as the benzylic proton of the *Z*-diastereomer

Rh_2(*S*-*p*-PhTPCP)$_4$: 9:1

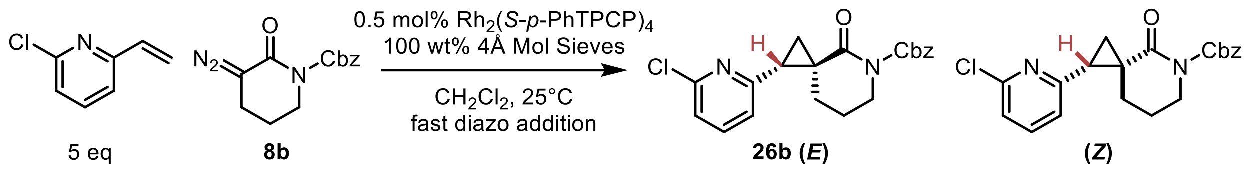

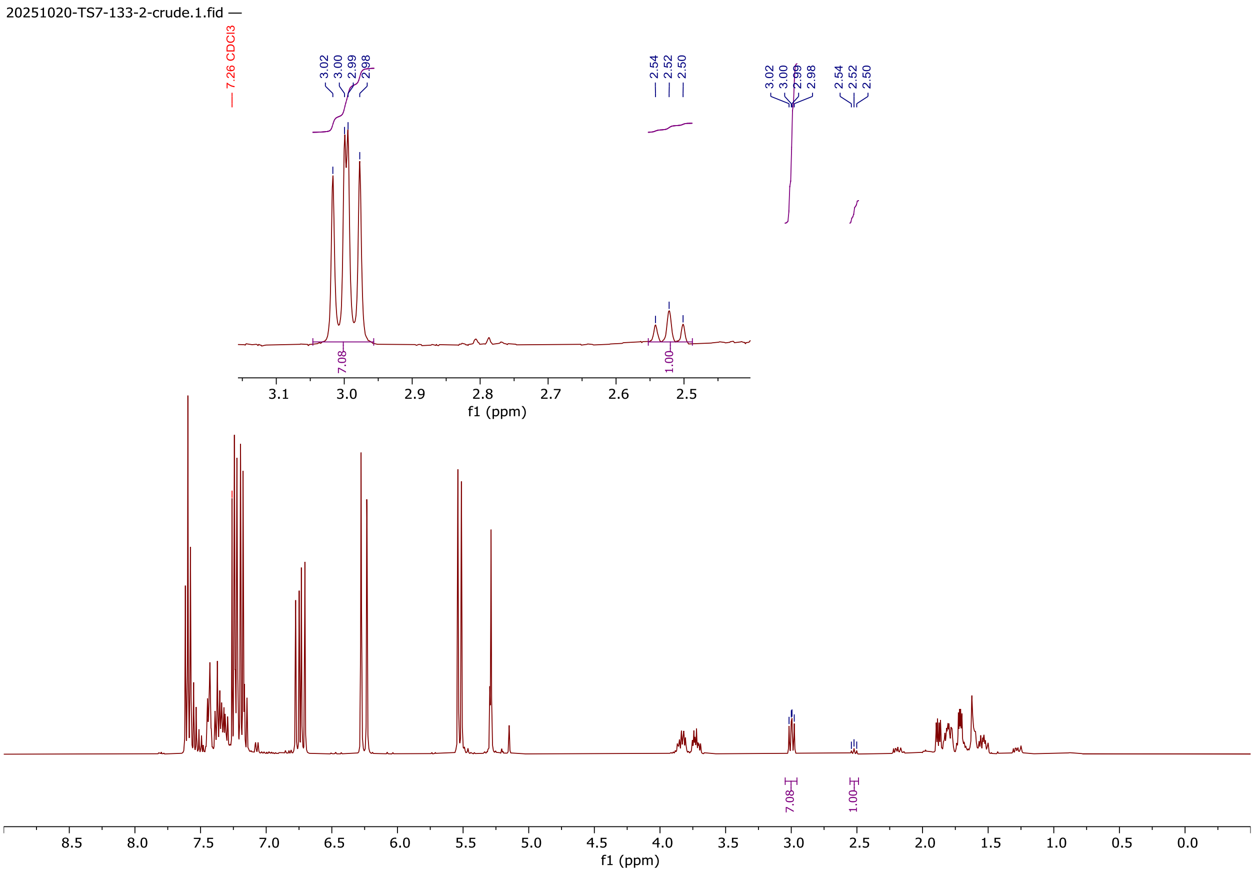

SI Figure S34. Diastereoselectivity of substrate **26b**

Signal at 3.00 (dd, J = 8.9, 6.9 Hz, 1H) was assigned as the benzylic proton of the *E*-diastereomer
Signal at 2.52 (app t, J = 8.1 Hz, 1H) was assigned as the benzylic proton of the *Z*-diastereomer

Rh_2(*S*-*p*-PhTPCP)$_4$: 7:1

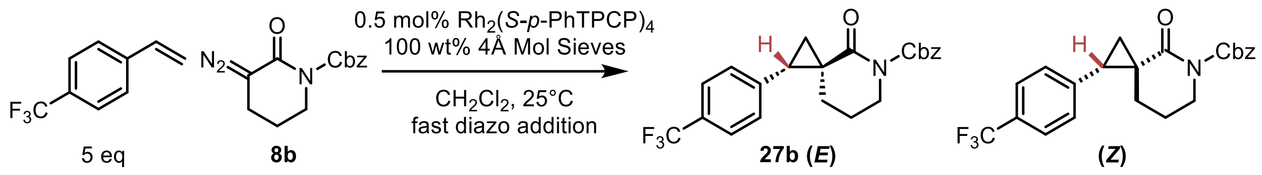

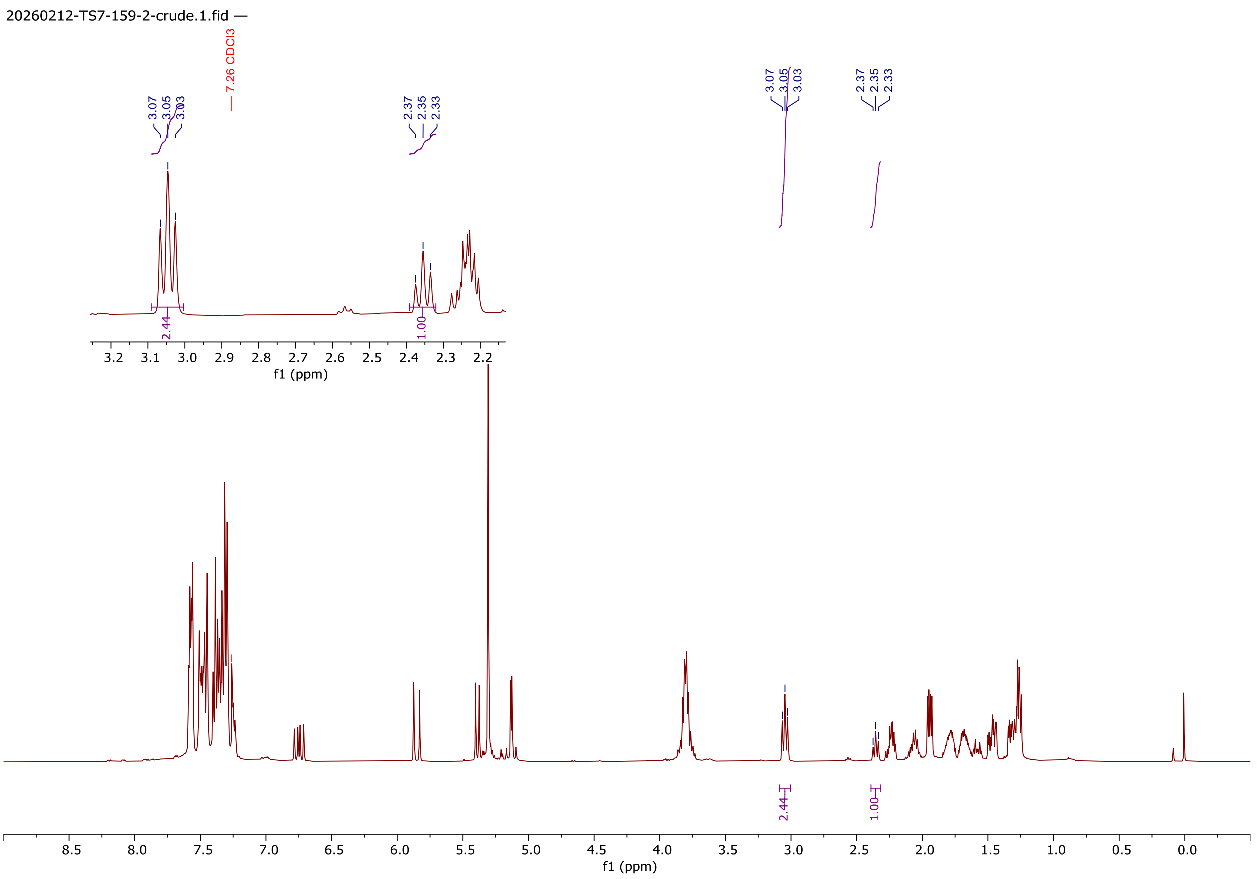

SI Figure S35. Diastereoselectivity of substrate **27b**

Signal at 3.05 (app t, J = 8.1 Hz, 2H) was assigned as the benzylic proton of the *E*-diastereomer
Signal at 2.35 (app t, *J* = 8.0 Hz, 1H) was assigned as the benzylic proton of the *Z*-diastereomer

Rh_2(*S*-*p*-PhTPCP)$_4$: 2:1

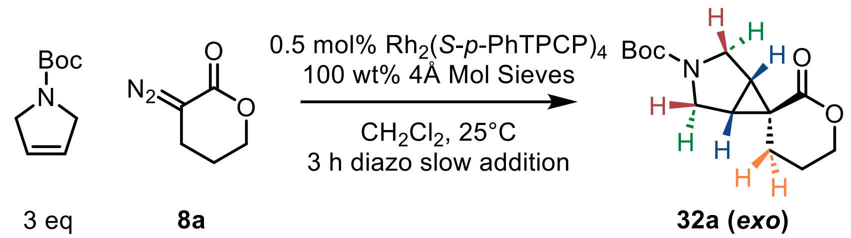

SI Figure S36. Diastereoselectivity of substrate **32a**

Rh_2(*S*-*p*-PhTPCP)$_4$: >20:1

Stereochemistry of **32b** was assigned by analogy

Notable signals utilized in determination of diastereoselectivity
Signal A at 3.64 – 3.56 (m, 2H) was assigned as the two α-to nitrogen protons on the top face (in red)
Signals B at 3.43 (d, J = 12.2 Hz, 1H), 3.35 (d, J = 12.1 Hz, 1H) were assigned as the two α-to nitrogen protons on the bottom face (in green)
Signal C at 2.35 – 2.27 (m, 2H) was assigned as the two cyclopropane bridgehead protons on the top face (in blue)
Signal D at 1.62 – 1.55 (m, 2H) was assigned as the two protons on the cyclohexanone (in orange)

^{1}H COSY NMR:

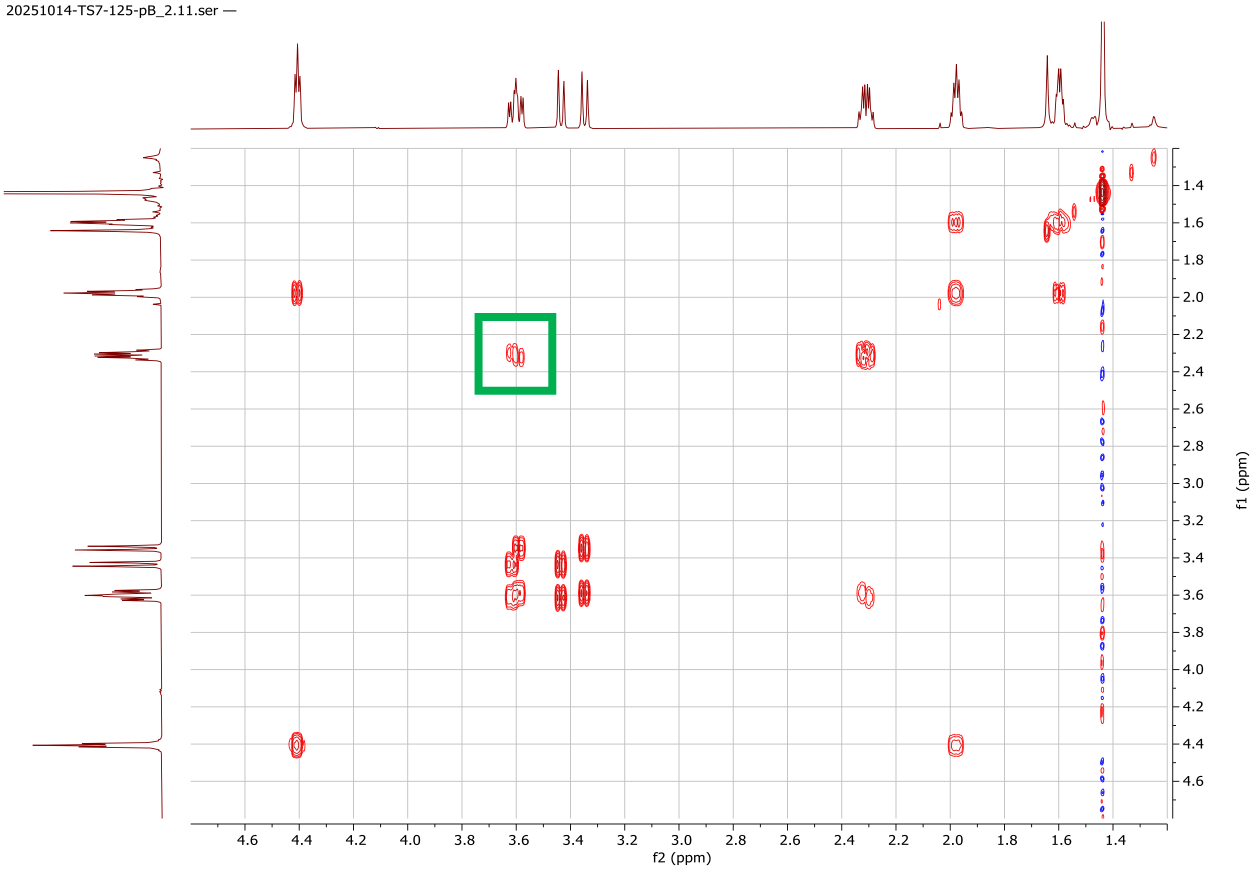

Signal A couples to signal C, indicating those protons are on the same face

Dihedral angle of signal A to C = 32.5° (calculated from Chem3D)
Dihedral angle of signal A to B = 88.1° (calculated from Chem3D)

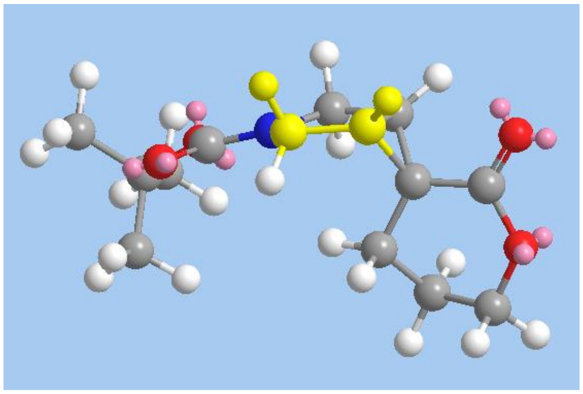

^{1}H NOESY NMR:

20251014-TS7-125-pB_2.12.ser —

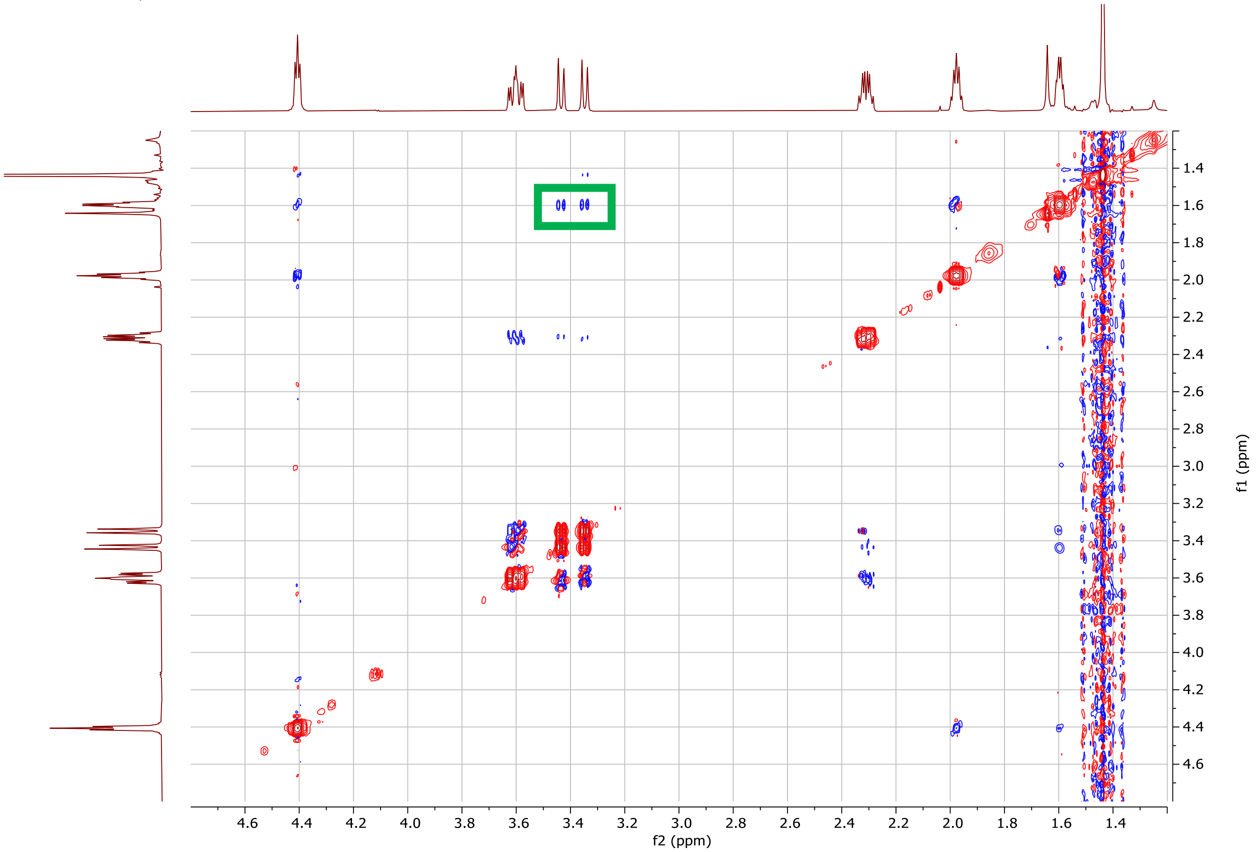

NOE correlation observed between Signal B and signal D through-space, indicating that the α-to-nitrogen protons on the bottom face are syn to the cyclohexanone methylene protons, confirming the relative stereochemistry.

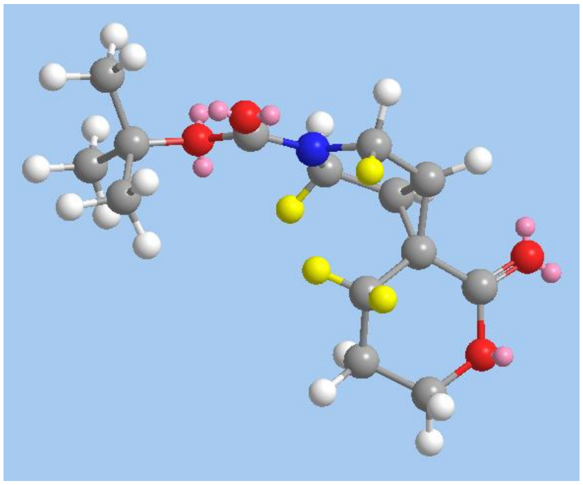

References

(1) Espino, C. G.; Fiori, K. W.; Kim, M.; Du Bois, J. Expanding the scope of C-H amination through catalyst design. *J. Am. Chem. Soc.* **2004**, *126*, 15378–15379.

(2) Davies, H. M. L.; Bruzinski, P. R.; Lake, D. H.; Kong, N.; Fall, M. J. Asymmetric Cyclopropanations by Rhodium(II) N-(Arylsulfonyl)prolinate Catalyzed Decomposition of Vinyldiazomethanes in the Presence of Alkenes. Practical Enantioselective Synthesis of the Four Stereoisomers of 2-Phenylcyclopropan-1-amino Acid. *J. Am. Chem. Soc.* **1996**, *118*, 6897–6907.

(3) Liao, K.; Liu, W.; Niemeyer, Z. L.; Ren, Z.; Bacsa, J.; Musaev, D. G.; Sigman, M. S.; Davies, H. M. L. Site-Selective Carbene-Induced C–H Functionalization Catalyzed by Dirhodium Tetrakis(triarylcyclopropanecarboxylate) Complexes. *ACS Catal.* **2017**, *8*, 678–682.

(4) Qin, C.; Davies, H. M. Role of sterically demanding chiral dirhodium catalysts in site-selective C-H functionalization of activated primary C-H bonds. *J. Am. Chem. Soc.* **2014**, *136*, 9792–9796.

(5) Liu, W.; Ren, Z.; Bosse, A. T.; Liao, K.; Goldstein, E. L.; Bacsa, J.; Musaev, D. G.; Stoltz, B. M.; Davies, H. M. L. Catalyst-Controlled Selective Functionalization of Unactivated C-H Bonds in the Presence of Electronically Activated C-H Bonds. *J. Am. Chem. Soc.* **2018**, *140*, 12247–12255.

(6) Ghanem, A.; Gardiner, M. G.; Williamson, R. M.; Muller, P. First X-ray structure of a N-naphthaloyl-tethered chiral dirhodium(II) complex: structural basis for tether substitution improving asymmetric control in olefin cyclopropanation. *Chem. Eur. J.* **2010**, *16*, 3291–3295.

(7) Reddy, R. P.; Davies, H. M. Dirhodium tetracarboxylates derived from adamantylglycine as chiral catalysts for enantioselective C-h aminations. *Org. Lett.* **2006**, *8*, 5013–5016.

(8) Fu, J.; Ren, Z.; Bacsa, J.; Musaev, D. G.; Davies, H. M. L. Desymmetrization of cyclohexanes by site- and stereoselective C-H functionalization. *Nature* **2018**, *564*, 395–399.

(9) Chen, K.; Zhang, S. Q.; Brandenberg, O. F.; Hong, X.; Arnold, F. H. Alternate Heme Ligation Steers Activity and Selectivity in Engineered Cytochrome P450-Catalyzed Carbene-Transfer Reactions. *J. Am. Chem. Soc.* **2018**, *140*, 16402–16407.

(10) Piperno, A.; Carnovale, C.; Giofrè, S. V.; Iannazzo, D. Hydrozirconation of four-, five-, six- and seven-membered N-alkoxycarbonyl lactams to lactamols. *Tetrahedron Lett.* **2011**, *52*, 6880–6882.

(11) DeAngelis, A.; Dmitrenko, O.; Fox, J. M. Rh-catalyzed intermolecular reactions of cyclic alpha-diazocarbonyl compounds with selectivity over tertiary C-H bond migration. *J. Am. Chem. Soc.* **2012**, *134*, 11035–11043.

(12) Lydon, K. M.; McKee, V.; McKervey, M. A. 3-Diazo-1-benzyloxycarbonylpyrrolidin-2-one. *Acta Crystallographica Section C Crystal Structure Communications* **1997**, *53*, 1675–1676.

(13) Zhukovsky, D.; Dar'in, D.; Krasavin, M. tert-Butyl 3-Diazo-2-oxopyrrolidine-1-carboxylate – A New Reagent for Introduction of the 2-Oxopyrrolidin-3-yl Motif via RhII-Catalyzed X–H Insertion Reactions. *Eur. J. Org. Chem.* **2020**, *2020*, 3013–3018.

(14) Chen, K.; Huang, X.; Zhang, S. Q.; Zhou, A. Z.; Kan, S. B. J.; Hong, X.; Arnold, F. H. Engineered Cytochrome c-Catalyzed Lactone-Carbene B-H Insertion. *Synlett* **2019**, *30*, 378–382.

(15) De Simone, F.; Gertsch, J.; Waser, J. Catalytic selective cyclizations of aminocyclopropanes: formal synthesis of aspidospermidine and total synthesis of goniomitine. *Angew. Chem. Int. Ed.* **2010**, *49*, 5767–5770.

(16) Bykowski, D.; Wu, K. H.; Doyle, M. P. Vinyldiazolactone as a vinylcarbene precursor: Highly selective C-H insertion and cyclopropanation reactions. *J. Am. Chem. Soc.* **2006**, *128*, 16038–16039.

(17) Denmark, S. E.; Gould, N. D.; Wolf, L. M. A systematic investigation of quaternary ammonium ions as asymmetric phase-transfer catalysts. Synthesis of catalyst libraries and evaluation of catalyst activity. *J. Org. Chem.* **2011**, *76*, 4260–4336.
(18) Janson, P. G.; Ghoneim, I.; Ilchenko, N. O.; Szabo, K. J. Electrophilic trifluoromethylation by copper-catalyzed addition of CF3-transfer reagents to alkenes and alkynes. *Org. Lett.* **2012**, *14*, 2882–2885.
(19) Browne, D. L.; Baumann, M.; Harji, B. H.; Baxendale, I. R.; Ley, S. V. A new enabling technology for convenient laboratory scale continuous flow processing at low temperatures. *Org. Lett.* **2011**, *13*, 3312–3315.
(20) Luongo, O. A.; Lemmerer, M.; Albers, S. L.; Streuff, J. Methoxide-Enabled Zirconium-Catalyzed Migratory Alkene Hydrosilylation. *Angew. Chem. Int. Ed. Engl.* **2024**, *63*, e202413182.
(21) Huffman, J.; Thompson, A.; Kabalka, G.; Akula, M. The Conversion of Phenols to the Corresponding Aryl Halides Under Mild Conditions. *Synthesis* **2005**, *2005*, 547–550.
(22) Banala, A. K.; Zhang, P.; Plenge, P.; Cyriac, G.; Kopajtic, T.; Katz, J. L.; Loland, C. J.; Newman, A. H. Design and synthesis of 1-(3-(dimethylamino)propyl)-1-(4-fluorophenyl)-1,3-dihydroisobenzofuran-5-carbonitrile (citalopram) analogues as novel probes for the serotonin transporter S1 and S2 binding sites. *J. Med. Chem.* **2013**, *56*, 9709–9724.
(23) Hilton, T. A.; Richardson, T. M.; McKay, A. P.; Watson, A. J. B. Copper-Catalyzed One-Pot Functionalization of Styrenes: Application toward Pharmaceutically Relevant Phenylethylamine Triazole Scaffolds. *J. Org. Chem.* **2025**, *90*, 13774–13778.
(24) Sharland, J. C.; Wei, B.; Hardee, D. J.; Hodges, T. R.; Gong, W.; Voight, E. A.; Davies, H. M. L. Asymmetric synthesis of pharmaceutically relevant 1-aryl-2-heteroaryl- and 1,2-diheteroarylcyclopropane-1-carboxylates. *Chem. Sci.* **2021**, *12*, 11181–11190.
(25) Sailer, J. K.; Ly, D.; Wang, A.; Musaev, D. G.; Davies, H. M. L. Enantioselective and Diastereoselective Synthesis of Azaspiro[n.2]alkanes by Rhodium-Catalyzed Cyclopropanations. *ACS Catal.* **2025**, *15*, 15253–15260.
(26) Nguyen, T. H.; Navarro, A.; Ruble, J. C.; Davies, H. M. L. Stereoselective Synthesis of Either Exo- or Endo-3-Azabicyclo[3.1.0]hexane-6-carboxylates by Dirhodium(II)-Catalyzed Cyclopropanation with Ethyl Diazoacetate under Low Catalyst Loadings. *Org. Lett.* **2024**, *26*, 2832–2836.

Experimental Procedures
General Procedures

General Procedure 1 (*GP-1*): Substrate scope with diazo **8a**

To a flame-dried 4 mL vial equipped with a stir bar and septa-lined plastic cap, was added activated 4Å Mol Sieves (100 wt%, 200 mg), Rh_2(*S*-*p*-PhTPCP)$_4$ (0.5 mol%, 1.0 µmol, 1.8 mg), and solid alkene substrate (2 eq, 0.4 mmol) or synthesized liquid alkene substrate (2 eq, 0.4 mmol, weighed by analytical balance). This was then degassed and backfilled with argon three times and left under an argon atmosphere via balloon. Commercially available liquid alkene substrates were added after degassing and backfilling with argon. To this, CH_2Cl_2 (0.4 M to diazo, 500 µL, distilled) was added and the resulting solution was allowed to stir at 25°C for 10 min to ensure homogeneity. To this, a previously-degassed solution of diazo **8a** (1.0 eq, 0.2 mmol, 25.4 mg) in CH_2Cl_2 (0.4 M to diazo, 500 µL, distilled) was added via dual syringe pump over 3 h [settings: 1 mL syringe, diameter 4.71 mm; Air-Tite/SilverPoint 22 G x 4" long hypodermic needle] and the resulting solution was left to stir for the indicated time, tracking the reaction via TLC. After complete diazo consumption, the reaction was filtered through celite, washing with CH_2Cl_2 and concentrated *in vacuo*. The crude residue was then purified as described. The fractions containing product were combined and concentrated *in vacuo*.

General Procedure 2 (*GP-2*): Substrate scope with diazo **8b**

To a flame-dried 4 mL vial equipped with a stir bar and septa-lined plastic cap, was added activated 4Å Mol Sieves (100 wt%, 200 mg), Rh_2(*S*-*p*-PhTPCP)$_4$ (0.5 mol%, 1.0 µmol, 1.8 mg), and solid alkene substrate (2 eq, 0.4 mmol) or synthesized liquid alkene substrate (2 eq, 0.4 mmol, weighed by analytical balance). This was then degassed and backfilled with argon three times and left under an argon atmosphere via balloon. Commercially available liquid alkene substrates were added after degassing and backfilling with argon. To this, CH_2Cl_2 (0.4 M to diazo, 500 µL, distilled) was added and the resulting solution was allowed to stir at 25°C for 10 min to ensure homogeneity. To this, a previously-degassed solution of diazo **8b** (1.0 eq, 0.2 mmol, 51.9 mg) in CH_2Cl_2 (0.4 M to diazo, 500 µL, distilled) was added dropwise over 30 sec and the resulting solution was left to stir for the indicated time, tracking the reaction via TLC. After complete diazo consumption, the reaction filtered through celite, washing with CH_2Cl_2 and concentrated *in vacuo*. The crude residue was then purified as described. The fractions containing product were combined and concentrated *in vacuo*.

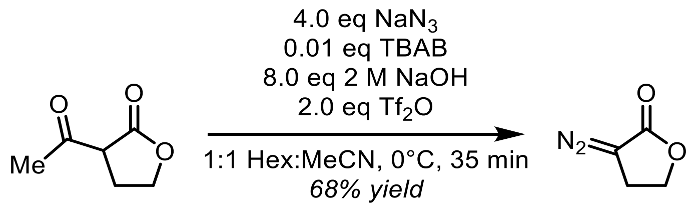

3-Diazodihydrofuran-2(3H)-one (6a)
Compound **6a** was prepared following a literature procedure.[9] Care was taken when handling NaN_3; plastic spatulas and a blast shield were used.

To an open-air (un-capped) 500 mL RBF equipped with a stir bar at 0°C, NaN_3 (4.0 eq, 80.0 mmol, 5.201 g), TBAB (0.01 eq, 0.2 mmol, 64.5 mg), and 2 M aq. NaOH (8.0 eq, 160.0 mmol, 6.4 g, 80.0 mL) were added and diluted with Hex (0.25 M, 80.0 mL, reagent grade). With vigorous stirring, Tf_2O (2.0 eq, 40.0 mmol, 11.285 g, 6.76 mL) was added dropwise and the resulting solution was stirred for 15 min. After this time, a previously prepared solution of α-acetyl butyrolactone (1.0 eq, 20.0 mmol, 2.5626 g, 2.16 mL) in MeCN (0.25 M, 80.0 mL, SPS grade) was added in one portion. After stirring at 0°C for an additional 20 min, the mixture was diluted with 50 mL of ice-cold water and 50 mL of chilled EtOAc and transferred to a 500 mL separatory funnel. The aqueous phase was washed with 50 mL of chilled EtOAc three times. The combined organic phases were washed with 50 mL of brine, dried over $MgSO_4$, filtered, and concentrated *in vacuo*. The crude residue was then dry-loaded onto silica gel and purified via automated flash column chromatography, eluting on a gradient. The collected fractions were concentrated *in vacuo* to afford 1522.8 mg of the product as a yellow-orange solid in 68% yield. ^{1}H NMR matched the reported spectra.[9]

Purification gradient:
20% EtOAc in Hex [5 CV] → 20 to 50% EtOAc in Hex [20 CV] → 50% EtOAc in Hex [5 CV]

^{1}H NMR (400 MHz, $CDCl_3$) δ 4.39 (dd, J = 8.3, 7.3 Hz, 2H), 3.40 – 3.33 (m, 2H).

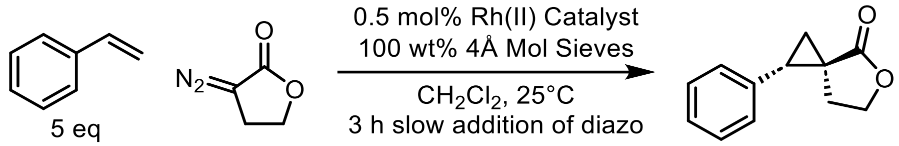

(1*S*,3*S*)-1-phenyl-5-oxaspiro[2.4]heptan-4-one (7a)

Styrene was filtered through a silica plug to remove inhibitor into a flame-dried 4 mL vial and bubbled with argon for 5 min before use. Absolute stereochemistry assigned by analogy to **24a**. A racemic sample was made following the same procedure but using 1 mol% $Rh_2(OAc)_4$. This procedure is for the catalyst screen – see **Table 1** in the main text for yields and catalysts used.

To a flame-dried 4 mL vial equipped with a stir bar and septa-lined plastic cap, was added activated 4Å Mol Sieves (100 wt%, 200 mg) and rhodium catalyst (0.5 mol%, 1.0 µmol). This was then degassed and backfilled with argon three times and left under an argon-filled balloon. To this, CH_2Cl_2 (0.5 M to diazo, 400 µL, distilled) was added, followed by styrene (5 eq, 1.0 mmol, 104 mg, 115 µL), and the resulting solution was allowed to stir at 25°C for 10 min to ensure homogeneity. To this, a previously-degassed solution of **6a** (1.0 eq, 0.2 mmol, 22.4 mg) in CH_2Cl_2 (0.5 M to diazo, 400 µL, distilled) was added via dual syringe pump over 3 h [settings: 1 mL syringe, diameter 4.71 mm; Air-Tite/SilverPoint 22 G x 4" long hypodermic needle] and the resulting solution was left to stir for the indicated time, tracking the reaction via TLC against diazo **6a**. After complete diazo consumption, the reaction was then filtered through celite with CH_2Cl_2 and concentrated *in vacuo*. The crude residue was then purified via pipette column, eluting 15% EtOAc in Hex, collecting each solvent replenishment as separate fractions. The *E* diastereomer elutes before the *Z* (collected separately) and these fractions were concentrated *in vacuo*. ^{1}H NMR matched the reported spectra.[9]

^{1}H NMR (400 MHz, CDCl$_3$) δ 7.37 – 7.30 (m, 2H), 7.29 – 7.23 (m, 1H), 7.16 – 7.08 (m, 2H), 4.40 (ddd, *J* = 9.0, 9.0, 5.6 Hz, 1H), 4.27 (ddd, *J* = 8.9, 8.9, 6.7 Hz, 1H), 2.76 (dd, *J* = 9.4, 7.1 Hz, 1H), 2.15 (ddd, *J* = 13.1, 8.9, 6.7 Hz, 1H), 1.90 (ddd, *J* = 13.0, 8.8, 5.6 Hz, 1H), 1.80 (dd, *J* = 9.4, 5.1 Hz, 1H), 1.46 (dd, *J* = 7.2, 5.1 Hz, 1H).

Chiral SFC: Sample dissolved in 80:20 Heptane:IPA
OZ-3 column, 1.0 mL/min, 2% 1:1 MeOH:IPA, 10 min
Retention times: major 5.02 min, minor 5.52 min.

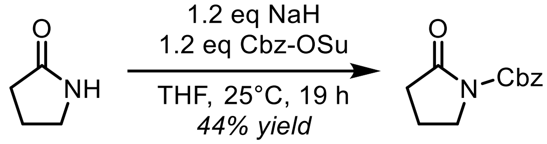

Benzyl 2-oxopyrrolidine-1-carboxylate (SI-1)

To a flame-dried 100 mL RBF equipped with a stir bar was added NaH (1.2 eq, 6.0 mmol, 240.0 mg, 60 wt% in mineral oil). This was then degassed and backfilled with argon three times and left under an argon-filled balloon, then diluted with THF (0.25 M to amide, 20.0 mL, SPS grade). To this, 2-pyrrolidone (1.0 eq, 5.0 mmol, 425.6 mg, 381.0 µL) was added dropwise and stirred at 25°C for 1 h, after which time, Cbz-OSu (1.2 eq, 6.0 mmol, 1.495 g) was added in one portion. The resulting solution was stirred at 25°C for 19 h. This was then quenched with 20 mL of brine and diluted with 20 mL of EtOAc. This was then extracted with 20 mL of EtOAc three times, washed with 20 mL of brine, dried over $MgSO_4$, filtered, then concentrated *in vacuo*. The crude residue was then purified via silica gel flash column chromatography, eluting 33% EtOAc in Hex. The collected fractions were concentrated *in vacuo* to afford 480.6 mg of the product in 44% yield. ^1H NMR matched the reported spectra.[10]

^{1}H NMR (400 MHz, $CDCl_3$) δ 7.45 – 7.40 (m, 2H), 7.40 – 7.31 (m, 3H), 5.28 (s, 2H), 3.81 (t, *J* = 7.2 Hz, 2H), 2.53 (t, *J* = 8.1 Hz, 2H), 2.03 (ddd, *J* = 15.3, 8.1, 7.1 Hz, 2H)

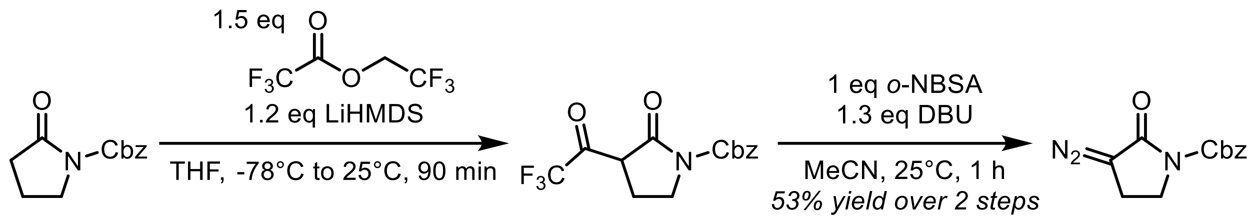

Benzyl 3-diazo-2-oxopyrrolidine-1-carboxylate (6b)
Compound **6b** was prepared adapting a literature procedure.[11]

To a flame-dried 100 mL RBF equipped with a stir bar and left under an argon-filled balloon, was added THF (0.38 M to carbamate, 20.0 mL, SPS grade) and LiHMDS (1.2 eq, 9.0 mmol, 9.0 mL, 1 M in THF), which was subsequently cooled to -78°C. To this, a previously degassed solution of **SI-1** (1.0 eq, 7.5 mmol, 1.6443 g) in THF (0.75 M to carbamate, 10.0 mL, SPS grade) was added dropwise and maintained at -78°C for 15 min. To this, 2,2,2-Trifluoroethyl 2,2,2-trifluoroacetate (1.5 eq, 11.25 mmol, 2.2056 g, 1.506 mL) was added dropwise and stirred at -78°C for 30 min, then warmed to 25°C over 1 h. The reaction was then quenched with 30 mL of a 10% aq. HCl solution and extracted with 30 mL of EtOAc three times. The combined organic phases were washed with 30 mL of brine, dried over $MgSO_4$, filtered, and concentrated *in vacuo*. The crude mixture was taken forward without further purification.

To the crude residue and *o*-NBSA (1.0 eq, 7.5 mmol, 1.7114 g) in a degassed 250 mL RBF equipped with a stir bar under an argon-filled balloon, MeCN (0.38 M to carbamate, 20.0 mL, SPS grade) was added. This was then cooled to 0°C, to which DBU (1.3 eq, 9.75 mmol, 1.4843 g, 1.47 mL) was added dropwise and stirred at 25°C for 1 h. The reaction was then quenched with 30 mL of brine and extracted with 30 mL of EtOAc three times. The combined organic phases were washed with 30 mL of brine, dried over $MgSO_4$, filtered, and concentrated *in vacuo*. The crude residue was then purified via silica gel flash column chromatography, eluting with 40% EtOAc in Hex, neutralizing the silica gel with a 5% TEA in Hex solution beforehand. The collected fractions were concentrated *in vacuo* to afford 975.8 mg of the product as a yellow-orange solid in 53% yield. ^{1}H NMR matched the reported spectra.[12]

R_f = 0.29, 50% EtOAc in Hex, 254 nm UV visualization

^{1}H NMR (400 MHz, $CDCl_3$) δ 7.46 – 7.39 (m, 2H), 7.39 – 7.28 (m, 3H), 5.28 (s, 2H), 3.88 – 3.79 (m, 2H), 3.14 – 3.04 (m, 2H).

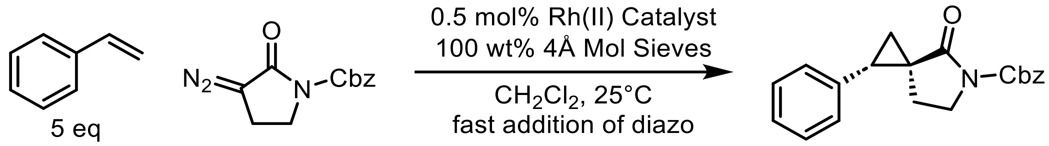

Benzyl (1*S*,3*S*)-4-oxo-1-phenyl-5-azaspiro[2.4]heptane-5-carboxylate (7b)
Styrene was filtered through a silica plug to remove inhibitor into a flame-dried 4 mL vial and bubbled with argon for 5 min before use. Absolute stereochemistry assigned by analogy to **24a**. A racemic sample was made following the same procedure but using 1 mol% $Rh_2(OAc)_4$. This procedure is for the catalyst screen – see **Table 1** in the main text for yields and catalysts used.

To a flame-dried 4 mL vial equipped with a stir bar and septa-lined plastic cap, was added activated 4Å Mol Sieves (100 wt%, 200 mg) and rhodium catalyst (0.5 mol% to diazo, 1.0 µmol). This was then degassed and backfilled with argon three times and left under an argon-filled balloon. To this, CH_2Cl_2 (0.4 M to diazo, 500 µL, distilled) was added, followed by styrene (5 eq, 1.0 mmol, 104 mg, 115 µL), and the resulting solution was allowed to stir at 25°C for 10 min to ensure homogeneity. To this, a previously-degassed solution of **5** (1.0 eq, 0.2 mmol, 49.0 mg) in CH_2Cl_2 (0.4 M to diazo, 500 µL, distilled) was added dropwise over 30 sec and the resulting solution was left to stir for 22 h. The reaction was then filtered through celite with CH_2Cl_2 and concentrated *in vacuo*. The crude residue was then purified via pipette column, eluting 25% EtOAc in Hex, collecting each solvent replenishment as separate fractions. The *E* and *Z* diastereomers were collected together and concentrated *in vacuo*.

R_f (product) = 0.33, 25% EtOAc in Hex, 254 nm UV visualization

^{1}H NMR (400 MHz, $CDCl_3$) δ 7.48 – 7.41 (m, 2H), 7.41 – 7.14 (m, 6H), 7.13 – 7.07 (m, 2H), 5.30 (s, 2H), 3.84 (dddd, *J* = 10.7, 9.3, 6.6, 5.5 Hz, 1H), 3.70 (ddd, *J* = 10.8, 9.3, 6.3 Hz, 1H), 2.75 (dd, *J* = 9.4, 7.2 Hz, 1H), 1.93 – 1.85 (m, 1H), 1.80 (dd, *J* = 9.3, 4.9 Hz, 1H), 1.69 (ddd, *J* = 13.3, 9.3, 5.4 Hz, 1H), 1.37 (dd, *J* = 7.2, 5.0 Hz, 1H).
Significant overlap with minor diastereomer in aromatic region.

^{13}C NMR (101 MHz, $CDCl_3$) δ 175.2, 151.6, 136.4, 135.5, 128.7, 128.6, 128.6, 128.3, 128.2, 127.1, 68.1, 43.8, 35.8, 31.6, 21.2, 19.1.
Significant overlap with minor diastereomer in aromatic region.

HRMS (+p APCI) calculated for $C_{20}H_{20}NO_3$ (M+H) 322.1445, found 322.1429

Chiral HPLC: Sample dissolved in 80:20 Hex:IPA
AS-H column, 1.0 mL/min, 4% IPA in Hex, 60 min
Retention times: major 40.52 min, minor 34.18 min.

1.2:1 mixture of diastereomers (E:Z) from reaction with Rh_2(S-p-PhTPCP)$_4$
$[\alpha]^{21.4}_D$ = -40.3° (c = 1.1, $CHCl_3$, 52% *ee of E-diastereomer*)

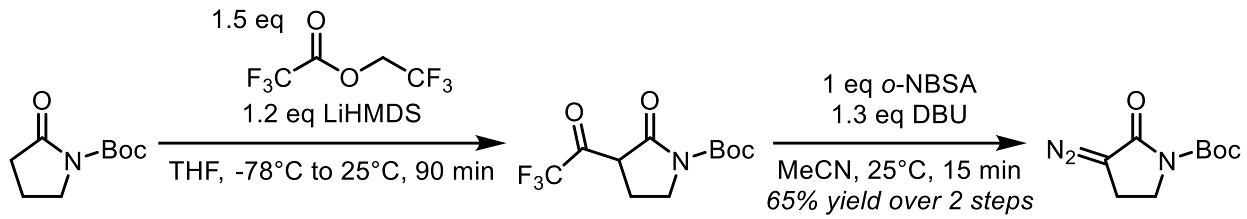

***tert*-Butyl 3-diazo-2-oxopyrrolidine-1-carboxylate (6c)**
Compound **6c** was prepared adapting a literature procedure.[11]

A flame-dried 50 mL RBF equipped with a stir bar was degassed and backfilled with argon three times, then left under an argon-filled balloon. THF (0.4 M to carbamate, 5.0 mL, SPS grade) and LiHMDS (1.2 eq, 2.4 mmol, 2.4 mL, 1.0 M in THF) were added, which was subsequently cooled to -78°C. To this, a previously degassed solution of N-Boc 2-pyrrolidinone (1.0 eq, 2.0 mmol, 370.4 mg, 341.1 µL) in THF (0.4 M to carbamate, 5.0 mL, SPS grade) was added dropwise and maintained at -78°C for 15 min. 2,2,2-trifluoroethyl 2,2,2-trifluoroacetate (1.5 eq, 3.0 mmol, 588.2 mg, 401.5 µL) was then added dropwise and stirred at -78°C for 30 min, then warmed to 25°C over 1 h. The reaction was then quenched with 30 mL of a 10% aq. HCl solution and extracted with 30 mL of EtOAc three times. The combined organic phases were washed with 30 mL of brine, dried over $MgSO_4$, filtered, and concentrated *in vacuo*. The crude mixture was taken forward without further purification.

The crude residue and *o*-NBSA (1.0 eq, 2.0 mmol, 456.4 mg) in a 20 mL vial equipped with a stir bar was degassed and backfilled with argon three times and left under an argon-filled balloon, to which MeCN (0.2 M to carbamate, 10.0 mL, SPS grade) was added. This was then cooled to 0°C, to which DBU (1.3 eq, 2.6 mmol, 395.8 mg, 391.9 µL) was added dropwise and stirred at 25°C for 15 min. The reaction was then quenched with 10 mL of brine and extracted with 20 mL of EtOAc three times. The combined organic phases were washed with 20 mL of brine, dried over $MgSO_4$, filtered, and concentrated *in vacuo*. The crude residue was then purified via silica gel flash column chromatography, eluting with 25% EtOAc in Hex, neutralizing the silica gel with a 3% TEA in Hex solution beforehand. The collected fractions were concentrated *in vacuo* to afford 287.7 mg of the product as a yellow-orange solid in 68% yield. ^1H NMR matched the reported spectra.[13]

R_f = 0.13, 25% EtOAc in Hex, 254 nm UV visualization

^1H NMR (400 MHz, $CDCl_3$) δ 3.81 – 3.73 (m, 2H), 3.12 – 3.03 (m, 2H), 1.52 (s, 9H).

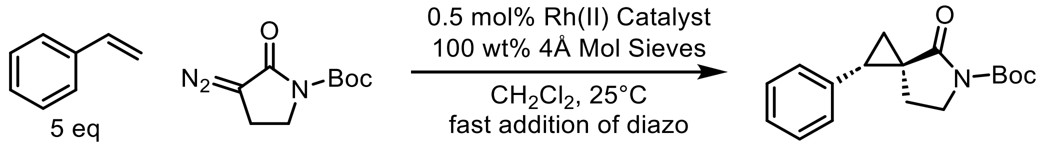

***tert*-Butyl (1*S*,3*S*)-4-oxo-1-phenyl-5-azaspiro[2.4]heptane-5-carboxylate (7c)**
Styrene was filtered through a silica plug to remove inhibitor into a flame-dried 4 mL vial and bubbled with argon for 5 min before use. Absolute stereochemistry assigned by analogy to **24a**. A racemic sample was made following the same procedure but using 1 mol% $Rh_2(OAc)_4$. This procedure is for the catalyst screen – see **Table 1** in the main text for yields and catalysts used.

To a flame-dried 4 mL vial equipped with a stir bar and septa-lined plastic cap, was added activated 4Å Mol Sieves (100 wt%, 194 mg) and rhodium catalyst (0.5 mol% to diazo, 0.97 µmol). This was then degassed and backfilled with argon three times and left under an argon-filled balloon. To this, CH_2Cl_2 (0.5 M to diazo, 400 µL, distilled) was added, followed by styrene (5 eq, 0.97 mmol, 101 mg, 111 µL), and the resulting solution was allowed to stir at 25°C for 10 min to ensure homogeneity. To this, a previously-degassed solution of **6c** (1.0 eq, 0.194 mmol, 41.0 mg) in CH_2Cl_2 (0.5 M to diazo, 400 µL, distilled) was added dropwise over 30 sec and the resulting solution was left to stir for 18 h. The reaction was then filtered through celite with CH_2Cl_2 and concentrated *in vacuo*. The crude residue was then purified via pipette column, eluting 15% EtOAc in Hex, collecting each solvent replenishment as separate fractions. The *E* and *Z* diastereomers were collected together and concentrated *in vacuo*.

^{1}H NMR (400 MHz, CDCl$_3$) δ 7.34 – 7.28 (m, 2H), 7.25 – 7.23 (m, 1H), 7.13 – 7.08 (m, 2H), 3.83 – 3.72 (m, 1H), 3.64 (ddd, J = 10.8, 9.3, 6.3 Hz, 1H), 2.73 (dd, J = 9.3, 7.1 Hz, 1H), 1.92 (dd, J = 7.8, 5.1 Hz, 1H), 1.77 (dd, J = 9.3, 4.9 Hz, 1H), 1.69 – 1.62 (m, 1H), 1.54 (s, 9H), 1.34 (dd, J = 7.1, 4.9 Hz, 1H).

^{13}C NMR (101 MHz, CDCl$_3$) δ 175.4, 150.5, 136.6, 128.6, 128.3, 127.0, 82.9, 43.8, 35.6, 31.4, 28.2, 21.1, 18.9, 17.6.

HRMS (+p APCI) calculated for $C_{17}H_{22}NO_3$ (M+H) 288.1601, found 288.1589

Chiral SFC: Sample dissolved in 80:20 Heptane:IPA
OJ-3 column, 1.0 mL/min, 1% 1:1 MeOH:IPA, 5 min
Retention times: major 4.34 min, minor 2.60 min.
Z diastereomer also separates on the same column conditions.

1.2:1 mixture of diastereomers (E:Z) from reaction with Rh_2(S-p-PhTPCP)$_4$
$[\alpha]^{21.2}_D$ = -33.7° (c = 0.68, $CHCl_3$, 34% *ee of E-diastereomer*)

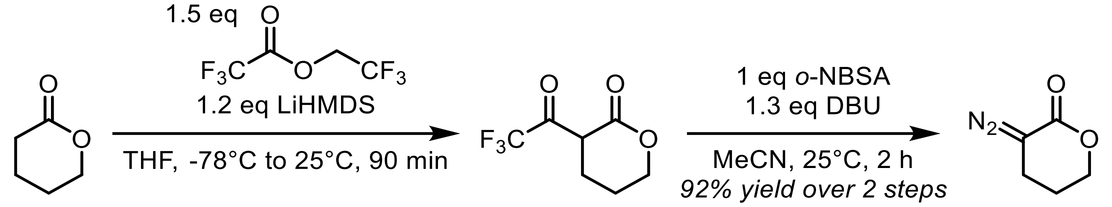

3-Diazotetrahydro-2H-pyran-2-one (8a)
Compound **8a** was prepared following a literature procedure.[11]

To a flame-dried 250 mL RBF equipped with a stir bar and left under an argon-filled balloon, was added THF (0.5 M to lactone, 40.0 mL, SPS grade) and LiHMDS (1.2 eq, 24.0 mmol, 24.0 mL, 1 M in THF), which was subsequently cooled to -78°C. To this, a previously degassed solution of α-valerolactone (1.0 eq, 20.0 mmol, 2.0024 g, 1.847 mL) was added dropwise and maintained at -78°C for 15 min. To this, 2,2,2-Trifluoroethyl 2,2,2-trifluoroacetate (1.5 eq, 30.0 mmol, 5.8815 g, 4.0 mL) was added dropwise and stirred at -78°C for 30 min, then warmed to 25°C over 1 h. The reaction was then quenched with 50 mL of a 10% aq. HCl solution and extracted with 50 mL of EtOAc three times. The combined organic phases were washed with 50 mL of brine, dried over $MgSO_4$, filtered, and concentrated *in vacuo*. The crude mixture was taken forward without further purification.

To the crude residue and *o*-NBSA (1.0 eq, 7.5 mmol, 1.7114 g) in a degassed 250 mL RBF equipped with a stir bar under an argon-filled balloon, MeCN (0.5 M to lactone, 40.0 mL, SPS grade) was added. This was then cooled to 0°C, to which DBU (1.3 eq, 26.0 mmol, 3.9682 g, 3.9 mL) was added dropwise and stirred at 25°C for 2 h. The reaction was then quenched with 30 mL of brine and extracted with 30 mL of EtOAc three times. The combined organic phases were washed with 30 mL of brine, dried over $MgSO_4$, filtered, and concentrated *in vacuo*. The crude residue was then dry-loaded onto silica gel and purified via automated flash column chromatography, eluting on a gradient. The collected fractions were concentrated *in vacuo* to afford 2441.3 mg of the yellow-orange product in 92% yield and 95% purity. ^{1}H NMR matched the reported spectra.[14]

Diazo **8a** is a solid at 0°C (fridge storage) but rapidly melts upon warming to 25°C. This compound was weighed as a frozen solid, taking the sample out of storage immediately before weighing for use in reactions.

R_f = 0.37, 50% EtOAc in Hex, 254 nm UV visualization

Purification gradient:
25% EtOAc in Hex [5 CV] → 25 to 65% EtOAc in Hex [20 CV] → 65% EtOAc in Hex [5 CV]

^{1}H NMR (400 MHz, $CDCl_3$) δ 4.30 – 4.22 (m, 2H), 2.79 (t, *J* = 6.5 Hz, 2H), 2.01 – 1.91 (m, 2H).

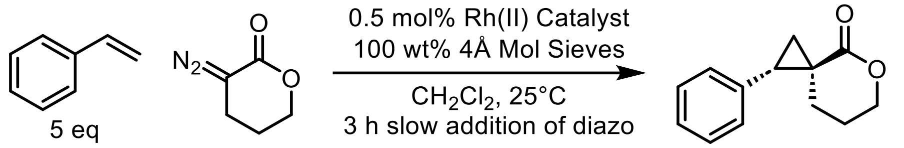

(1*S*,3*R*)-1-phenyl-5-oxaspiro[2.5]octan-4-one (10a)

Styrene was filtered through a silica plug to remove inhibitor into a flame-dried 4 mL vial and bubbled with argon for 5 min before use. Absolute stereochemistry assigned by analogy to **24a**. A racemic sample was made following the same procedure but using 1 mol% $Rh_2(esp)_2$. This procedure is for the catalyst screen – see **Table 2** in the main text for yields and catalysts used.

To a flame-dried 4 mL vial equipped with a stir bar and septa-lined plastic cap, was added activated 4Å Mol Sieves (100 wt%, 200 mg) and rhodium catalyst (0.5 mol% to diazo, 1.0 µmol). This was then degassed and backfilled with argon three times and left under an argon-filled balloon. To this, CH_2Cl_2 (0.4 M to diazo, 500 µL, distilled) was added, followed by styrene (5 eq, 1.0 mmol, 104 mg, 115 µL), and the resulting solution was allowed to stir at 25°C for 10 min to ensure homogeneity. To this, a previously-degassed solution of **8a** (1.0 eq, 0.2 mmol, 49.0 mg) in CH_2Cl_2 (0.4 M to diazo, 500 µL, distilled) was added via dual syringe pump over 3 h [settings: 1 mL syringe, diameter 4.71 mm; Air-Tite/SilverPoint 22 G x 4" long hypodermic needle] and the resulting solution was left to stir for 22 h. The reaction was then filtered through celite with CH_2Cl_2 and concentrated *in vacuo*. The crude residue was then purified via pipette column, eluting 25% EtOAc in Hex, collecting each solvent replenishment as separate fractions. The *E* and *Z* diastereomers were collected together and concentrated *in vacuo*.

R_f (product) = 0.33, 25% EtOAc in Hex, 254 nm UV visualization

^{1}H NMR (400 MHz, CDCl$_3$) δ 7.37 – 7.29 (m, 2H), 7.29 – 7.24 (m, 1H), 7.23 – 7.16 (m, 2H), 4.47 – 4.33 (m, 2H), 2.99 (dd, *J* = 9.2, 7.3 Hz, 1H), 1.93 (dd, *J* = 9.2, 4.6 Hz, 1H), 1.88 – 1.68 (m, 2H), 1.54 (ddd, *J* = 13.1, 8.5, 4.2 Hz, 1H), 1.36 (dddd, *J* = 14.0, 7.6, 4.2, 1.0 Hz, 1H), 1.27 (dd, *J* = 7.3, 4.6 Hz, 1H).

^{13}C NMR (101 MHz, CDCl$_3$) δ 174.4, 135.9, 129.3, 128.5, 127.2, 70.4, 34.6, 26.0, 24.9, 23.2, 21.4.

HRMS (+p APCI) calculated for $C_{13}H_{15}O_2$ (M+H) 203.1074, found 203.1064

Chiral SFC: Sample dissolved in 80:20 Heptane:IPA
OJ-3 column, 2.5 mL/min, 3% 1:1 MeOH:IPA, 5 min.
Retention times: major 1.94 min, minor 2.44 min.

$[\alpha]^{21.5}_D$ = -65.2° (c = 1.43, $CHCl_3$, 90% *ee of E-diastereomer*)

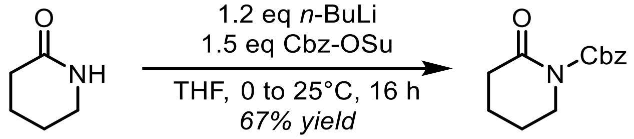

Benzyl 2-oxopiperidine-1-carboxylate (**SI-2**)
2-Piperidone is a low melting point solid. Material weighed as a frozen solid, taking out of fridge storage immediately before use.

To a flame-dried 250 mL RBF equipped with a stir bar and left under an argon-filled balloon, 2-piperidone (1.0 eq, 22.45 mmol, 2.2257 g) was added, which was then diluted with THF (0.22 M to amide, 100.0 mL, SPS grade). To this solution cooled to 0°C, *n*-BuLi (1.2 eq, 26.94 mmol, 10.78 mL, 2.5 M in Hex) was added dropwise. The resulting solution was stirred and allowed to naturally warm to 25°C for over 90 min. To this, Cbz-OSu (1.5 eq, 33.68 mmol, 8.393 g) was added in one portion and the resulting solution was refluxed via open-top findenser over 16 h. This was then quenched with 50 mL of sat. aq. NH_4Cl and extracted with 50 mL of EtOAc three times, washed with 50 mL of brine, dried over $MgSO_4$, filtered, then concentrated *in vacuo*. The crude residue was then dry-loaded onto silica gel and purified via automated flash column chromatography, eluting on a gradient. The collected fractions were concentrated *in vacuo* to afford 3488.4 mg of the product in 67% yield. ^{1}H NMR matched the reported spectra.[15]

R_f = 0.26, 25% EtOAc in Hex, 254 nm UV visualization

Purification gradient:
40% Et_2O in Hex [5 CV] → 40 to 70% Et_2O in Hex [20 CV] → 70% Et_2O in Hex [5 CV]

^{1}H NMR (400 MHz, $CDCl_3$) δ 7.46 – 7.41 (m, 2H), 7.40 – 7.31 (m, 3H), 5.28 (s, 2H), 3.74 (td, *J* = 5.0, 2.5 Hz, 2H), 2.54 (td, *J* = 6.0, 2.3 Hz, 2H), 1.84 (p, *J* = 3.4 Hz, 4H).

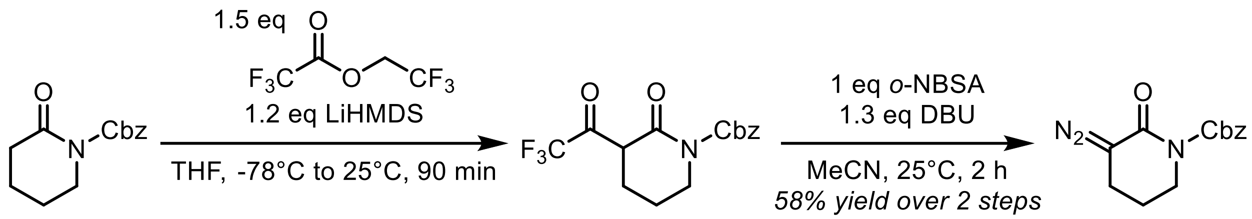

Benzyl 3-diazo-2-oxopiperidine-1-carboxylate (8b)
Compound **8b** was prepared adapting a literature procedure.[11]

To a flame-dried 250 mL RBF equipped with a stir bar and left under an argon-filled balloon, was added THF (0.4 M to carbamate, 40.0 mL, SPS grade) and LiHMDS (1.2 eq, 19.5 mmol, 19.5 mL, 1 M in THF), which was subsequently cooled to -78°C. To this, a previously degassed solution of **SI-2** (1.0 eq, 16.22 mmol, 4203.9 mg, 90% pure) in THF (0.8 M to carbamate, 20.0 mL, SPS grade) was added dropwise and maintained at -78°C for 30 min. To this, 2,2,2-Trifluoroethyl 2,2,2-trifluoroacetate (1.5 eq, 24.33 mmol, 4.7697 g, 3.3 mL) was added dropwise and stirred at -78°C for 30 min, then warmed to 25°C over 1 h. The reaction was then quenched with 30 mL of a 10% aq. HCl solution and extracted with 30 mL of EtOAc three times. The combined organic phases were washed with 30 mL of brine, dried over $MgSO_4$, filtered, and concentrated *in vacuo*. The crude mixture was taken forward without further purification.

To the crude residue and *o*-NBSA (1.0 eq, 16.22 mmol, 3.7010 g) in a degassed 250 mL RBF equipped with a stir bar under an argon-filled balloon, MeCN (0.4 M to carbamate, 40.0 mL, SPS grade) was added. This was then cooled to 0°C, to which DBU (1.3 eq, 21.1 mmol, 3.21 g, 3.2 mL) was added dropwise and stirred at 25°C for 1 h. The reaction was then quenched with 30 mL of brine and extracted with 30 mL of EtOAc three times. The combined organic phases were washed with 30 mL of brine, dried over $MgSO_4$, filtered, and concentrated *in vacuo*. The crude residue was then dry-loaded onto silica gel and purified via automated flash column chromatography, eluting on a gradient. The collected fractions were concentrated *in vacuo* to afford 2456.6 mg of the product as a yellow-orange solid in 58% yield. The *o*-NBSA byproduct elutes closely to product. Only clean fractions of the product were collected.

R_f = 0.38, 50% EtOAc in Hex, 254 nm UV visualization

Purification gradient:
15% EtOAc in Hex [5 CV] → 15 to 45% EtOAc in Hex [20 CV] → 45% EtOAc in Hex [5 CV]

^{1}H NMR (400 MHz, $CDCl_3$) δ 7.47 – 7.42 (m, 2H), 7.40 – 7.28 (m, 3H), 5.29 (s, 2H), 3.80 – 3.73 (m, 2H), 2.76 (t, *J* = 6.6 Hz, 2H), 1.93 (ddd, *J* = 13.2, 7.1, 4.5 Hz, 2H).

^{13}C NMR (101 MHz, $CDCl_3$) δ 164.7, 153.7, 135.6, 128.7, 128.3, 128.1, 68.7, 58.6, 45.4, 22.0, 21.5.

HRMS (+p APCI) calculated for $C_{13}H_{14}N_3O_3$ (M+H) 260.1037, found 260.1028

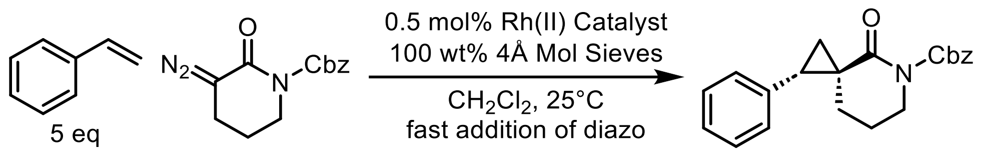

Benzyl (1*S*,3*R*)-4-oxo-1-phenyl-5-azaspiro[2.5]octane-5-carboxylate (10b)
Styrene was filtered through a silica plug to remove inhibitor into a flame-dried 4 mL vial and bubbled with argon for 5 min before use. Absolute stereochemistry assigned by analogy to **24a**. A racemic sample was made following the same procedure but using 1 mol% $Rh_2(esp)_2$.

To a flame-dried 4 mL vial equipped with a stir bar and septa-lined plastic cap, was added activated 4Å Mol Sieves (100 wt%, 200 mg) and Rh_2(*S*-*p*-PhTPCP)$_4$ (0.5 mol%, 1.0 µmol, 1.8 mg). This was then degassed and backfilled with argon three times and left under an argon-filled balloon. To this, CH_2Cl_2 (0.4 M to diazo, 500 µL, distilled) was added, followed by styrene (5 eq, 1.0 mmol, 104 mg, 115 µL), and the resulting solution was allowed to stir at 25°C for 10 min to ensure homogeneity. To this, a previously-degassed solution of diazo **8b** (1.0 eq, 0.2 mmol, 49.0 mg) in CH_2Cl_2 (0.4 M to diazo, 500 µL, distilled) was added dropwise over 30 sec and the resulting solution was left to stir for 22 h. The reaction was then filtered through celite with CH_2Cl_2 and concentrated *in vacuo*. The crude residue was then dry-loaded onto silica gel and purified via automated flash column chromatography, eluting on a gradient. The *E* and *Z* diastereomers were separable, only E diastereomer collected.

Purification gradient:
0% EtOAc in Hex [5 CV] → 0 to 25% EtOAc in Hex [20 CV] → 25% EtOAc in Hex [5 CV]

^{1}H NMR (400 MHz, CDCl$_3$) δ 7.46 (dt, *J* = 6.1, 1.6 Hz, 2H), 7.43 – 7.35 (m, 2H), 7.35 – 7.28 (m, 3H), 7.27 – 7.24 (m, 1H), 7.21 – 7.15 (m, 2H), 5.30 (s, 2H), 3.79 (t, *J* = 6.2 Hz, 2H), 3.00 (dd, *J* = 9.2, 7.2 Hz, 1H), 1.91 (dd, *J* = 9.2, 4.4 Hz, 1H), 1.84 – 1.62 (m, 2H), 1.48 (ddd, *J* = 13.8, 9.0, 3.6 Hz, 1H), 1.33 (ddd, *J* = 13.8, 7.8, 3.6 Hz, 1H), 1.23 (dd, *J* = 7.3, 4.4 Hz, 1H).

^{13}C NMR (101 MHz, CDCl$_3$) δ 173.5, 154.2, 136.5, 135.7, 129.3, 128.7, 128.4, 128.3, 127.1, 77.5, 76.8, 68.6, 47.8, 34.3, 30.2, 25.5, 22.1, 21.1.

HRMS (+p APCI) calculated for $C_{21}H_{22}NO_3$ (M+H) 336.1601, found 336.1591

Chiral HPLC: Sample dissolved in 80:20 Hex:IPA
AS-H column, 1.0 mL/min, 5.0% IPA in Hex, 30 min – 95% *ee*
Retention times: major 20.90 min, minor 18.59 min.

$[\alpha]^{21.5}_D$ = -124.6° (c = 1.60, $CHCl_3$, 96% *ee of E-diastereomer*)

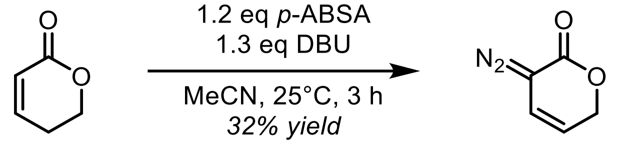

3-diazo-3,6-dihydro-2H-pyran-2-one (11)
Diazo **11** was prepared adapting the literature precedent.[16]

To a flame-dried 100 mL RBF equipped with a stir bar was added *p*-ABSA (1.2 eq, 6.0 mmol, 1.441 g). This was degassed and backfilled with argon three times, then left under an argon-filled balloon. MeCN (0.13 M, 40.0 mL, SPS grade) was added to the flask, followed by 5,6-dihydro-2H-pyran-2-one (1.0 eq, 5.0 mmol, 490.5 mg, 429.9 µL). To this solution at 0°C, DBU (1.3 eq, 6.5 mmol, 989.6 mg, 972.1 µL) was added dropwise. The resulting solution was stirred at 25°C for 3 h. After the elapsed time, the reaction was concentrated *in vacuo*, dry-loaded onto silica, and purified via automated flash column chromatography, eluting on a gradient. The yellow fractions containing product were combined and concentrated *in vacuo* to obtain 219.9 mg of a yellow solid in 90% purity and 32% yield. ^{1}H NMR matched the reported spectra.[16]

Purification gradient:
10% EtOAc in Hex [5 CV] → 10 to 40% EtOAc in Hex [20 CV] → 40% EtOAc in Hex [5 CV]

^{1}H NMR (400 MHz, $CDCl_3$) δ 6.18 (dt, *J* = 9.8, 1.8 Hz, 1H), 5.36 (dt, *J* = 9.9, 3.3 Hz, 1H), 5.00 (dd, *J* = 3.4, 1.8 Hz, 2H).

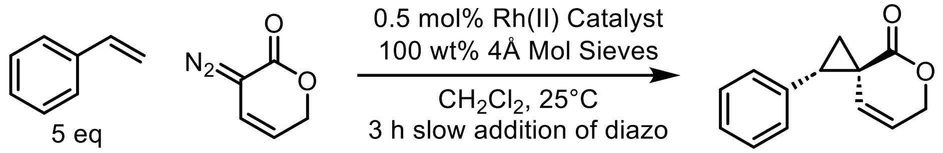

(1*S*,3*S*)-1-phenyl-5-oxaspiro[2.5]oct-7-en-4-one (12)

Styrene was filtered through a silica plug to remove inhibitor into a flame-dried 4 mL vial and bubbled with argon for 5 min before use. Absolute stereochemistry assigned by analogy to **24a**. A racemic sample was made following the same procedure but using 1 mol% $Rh_2(OAc)_4$.

To a flame-dried 4 mL vial equipped with a stir bar and septa-lined plastic cap, was added activated 4Å Mol Sieves (100 wt%, 200 mg) and Rh_2(*S*-*p*-PhTPCP)$_4$ (0.5 mol%, 1.0 µmol, 1.8 mg). This was then degassed and backfilled with argon three times and left under an argon-filled balloon. To this, CH_2Cl_2 (0.4 M to diazo, 500 µL, distilled) was added, followed by styrene (5 eq, 1.0 mmol, 104 mg, 115 µL), and the resulting solution was allowed to stir at 25°C for 10 min to ensure homogeneity. To this, a previously-degassed solution of **11** (1.0 eq, 0.2 mmol, 24.8 mg) in CH_2Cl_2 (0.4 M to diazo, 500 µL, distilled) was added via dual syringe pump over 3 h [settings: 1 mL syringe, diameter 4.71 mm; Air-Tite/SilverPoint 22 G x 4" long hypodermic needle] and the resulting solution was left to stir for 10 min. The reaction was then filtered through celite with CH_2Cl_2 and concentrated *in vacuo*. The crude residue was then dry-loaded onto silica gel and purified via automated flash column chromatography, eluting on a gradient. ^{1}H NMR matched the reported spectra.[16]

Purification gradient:
0% EtOAc in Hex [5 CV] → 0 to 25% EtOAc in Hex [20 CV] → 25% EtOAc in Hex [5 CV]

^{1}H NMR (400 MHz, $CDCl_3$) δ 7.36 – 7.29 (m, 2H), 7.29 – 7.23 (m, 1H), 7.23 – 7.18 (m, 2H), 5.72 (dt, J = 10.2, 2.9 Hz, 1H), 5.01 (dd, J = 2.9, 2.0 Hz, 2H), 4.96 (dt, J = 10.2, 2.0 Hz, 1H), 3.25 (dd, J = 9.1, 7.7 Hz, 1H), 2.14 (dd, J = 9.1, 4.8 Hz, 1H), 1.55 (dd, J = 7.6, 4.8 Hz, 1H).

Chiral HPLC: Sample dissolved in 80:20 Hex:IPA
AS-H column, 1.0 mL/min, 10% IPA in Hex, 30 min – 30% *ee*
Retention times: major 24.33 min, minor 17.74 min.

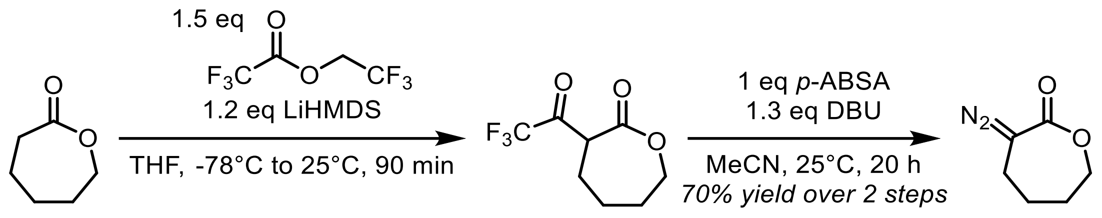

3-Diazooxepan-2-one (13)

Compound **13** was prepared adapting a literature procedure.[11]

To a flame-dried 100 mL RBF equipped with a stir bar and left under an argon-filled balloon, was added THF (0.5 M to lactone, 20.0 mL, SPS grade) and LiHMDS (1.2 eq, 12.0 mmol, 12.0 mL, 1 M in THF), which was subsequently cooled to -78°C. To this, a previously degassed solution of ε-caprolactone (1.0 eq, 10.0 mmol, 1.1414 g, 1.082 mL) was added dropwise and maintained at -78°C for 15 min. To this, 2,2,2-Trifluoroethyl 2,2,2-trifluoroacetate (1.3 eq, 13.0 mmol, 2.5487 g, 1.7 mL) was added dropwise and stirred at -78°C for 30 min, then warmed to 25°C over 1 h. The reaction was then quenched with 30 mL of a 10% aq. HCl solution and extracted with 30 mL of EtOAc three times. The combined organic phases were washed with 30 mL of brine, dried over $MgSO_4$, filtered, and concentrated *in vacuo*. The crude mixture was taken forward without further purification.

To the crude residue and *p*-ABSA (1.0 eq, 10.0 mmol, 2.4024 g) in a degassed 100 mL RBF equipped with a stir bar under an argon-filled balloon, MeCN (0.5 M to lactone, 20.0 mL, SPS grade) was added. This was then cooled to 0°C, to which DBU (1.3 eq, 13.0 mmol, 1.9791 g, 2.0 mL) was added dropwise and stirred at 25°C for 20 h. The reaction was then quenched with 30 mL of brine and extracted with 30 mL of EtOAc three times. The combined organic phases were washed with 30 mL of brine, dried over $MgSO_4$, filtered, and concentrated *in vacuo*. The crude residue was then purified via silica gel flash column chromatography, eluting 20% EtOAc in Hex, thoroughly neutralizing the silica gel with a 5% TEA in Hex solution beforehand. The collected fractions were concentrated *in vacuo* to afford 984.7 mg of a yellow oil in 70% yield.

^{1}H NMR (400 MHz, $CDCl_3$) δ 4.24 (dd, *J* = 5.9, 4.6 Hz, 2H), 2.59 – 2.52 (m, 2H), 1.90 (ddt, *J* = 10.4, 8.7, 4.0 Hz, 4H).

^{13}C NMR (101 MHz, $CDCl_3$) δ 171.9, 68.3, 58.6, 28.1, 24.7, 23.6.

HRMS (+p APCI) calculated for $C_6H_9N_2O_2$ (M+H) 141.0666, found 141.0659

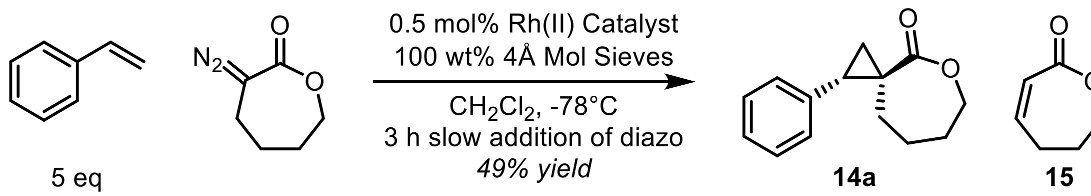

(1*S*,3*R*)-1-phenyl-5-oxaspiro[2.6]nonan-4-one (14a)

Styrene was filtered through a silica plug to remove inhibitor into a flame-dried 4 mL vial and bubbled with argon for 5 min before use. Absolute stereochemistry assigned by analogy to **24a**. A racemic sample was made following the same procedure but using 1 mol% $Rh_2(OAc)_4$. The same procedure was utilized when running the reaction at 25°C, resulting in a 29% yield of **14a**. **15** was isolated as a side-product from the above reaction, the ^{1}H NMR for which matched the reported spectra.[17]

To a flame-dried 4 mL vial equipped with a stir bar and septa-lined plastic cap, was added activated 4Å Mol Sieves (100 wt%, 200 mg) and Rh_2(*S*-*p*-PhTPCP)$_4$ (0.5 mol%, 1.0 µmol, 1.8 mg). This was then degassed and backfilled with argon three times and left under an argon-filled balloon. To this, CH_2Cl_2 (0.4 M to diazo, 500 µL, distilled) was added, followed by styrene (5 eq, 1.0 mmol, 104 mg, 115 µL), and the resulting solution was cooled to and allowed to stir at -78°C for 10 min to ensure homogeneity. To this, a previously-degassed solution of **13** (1.0 eq, 0.2 mmol, 28.0 mg) in CH_2Cl_2 (0.4 M to diazo, 500 µL, distilled) was added via dual syringe pump over 3 h [settings: 1 mL syringe, diameter 4.71 mm; Air-Tite/SilverPoint 22 G x 4" long hypodermic needle] and the resulting solution was left to stir for 30 min naturally allowing the reaction to warm to 25°C without replenishing the cooling bath. The reaction was then filtered through celite with CH_2Cl_2 and concentrated *in vacuo*. The crude residue was then purified via flash column chromatography, eluting 15% EtOAc in Hex. The collected fractions were concentrated *in vacuo* to afford 21.2 mg of a white solid in 49% yield.

^{1}H NMR (400 MHz, $CDCl_3$) δ 7.36 – 7.28 (m, 2H), 7.28 – 7.24 (m, 1H), 7.24 – 7.18 (m, 2H), 4.34 (dd, *J* = 5.5, 4.2 Hz, 2H), 2.83 (dd, *J* = 9.3, 7.0 Hz, 1H), 1.90 – 1.71 (m, 2H), 1.67 (dd, *J* = 9.3, 5.2 Hz, 1H), 1.56 – 1.31 (m, 4H), 1.28 (dd, *J* = 7.1, 5.3 Hz, 1H).

^{13}C NMR (101 MHz, $CDCl_3$) δ 176.7, 136.5, 129.0, 128.5, 127.0, 68.9, 30.4, 29.9, 28.9, 27.2, 24.9, 17.4.

HRMS (+p APCI) calculated for $C_{14}H_{17}O_2$ (M+H) 217.1230, found 217.1221

Chiral HPLC: Sample dissolved in 80:20 Hex:IPA
AD-H column, 1.0 mL/min, 2% IPA in Hex, 30 min – 99% *ee*
Retention times: major 13.46 min, minor 15.88 min.

$[\alpha]^{22.0}_D$ = -37.9° (c = 0.46, $CHCl_3$, 99% *ee of E-diastereomer*)

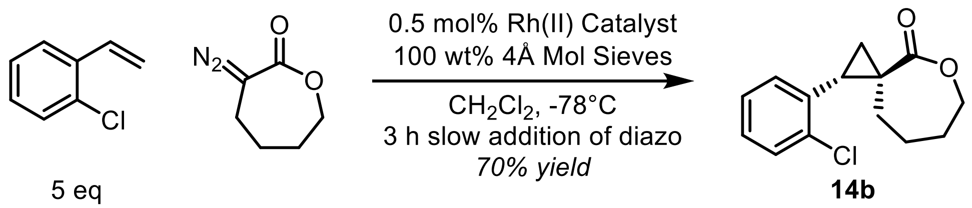

(1*R*,3*R*)-1-(2-chlorophenyl)-5-oxaspiro[2.6]nonan-4-one (14b)
2-Chlorostyrene was filtered through a silica plug to remove inhibitor into a flame-dried 4 mL vial and bubbled with argon for 5 min before use. Absolute stereochemistry assigned by analogy to **24a**. A racemic sample was made following the same procedure but using 1 mol% $Rh_2(OAc)_4$.

To a flame-dried 4 mL vial equipped with a stir bar and septa-lined plastic cap, was added activated 4Å Mol Sieves (100 wt%, 200 mg) and Rh_2(*S*-*p*-PhTPCP)$_4$ (0.5 mol%, 1.0 µmol, 1.8 mg). This was then degassed and backfilled with argon three times and left under an argon-filled balloon. To this, CH_2Cl_2 (0.4 M to diazo, 500 µL, distilled) was added, followed by 2-chlorostyrene (5 eq, 1.0 mmol, 139 mg, 128 µL), and the resulting solution was cooled to and allowed to stir at -78°C for 10 min to ensure homogeneity. To this, a previously-degassed solution of **13** (1.0 eq, 0.2 mmol, 28.0 mg) in CH_2Cl_2 (0.4 M to diazo, 500 µL, distilled) was added via dual syringe pump over 3 h [settings: 1 mL syringe, diameter 4.71 mm; Air-Tite/SilverPoint 22 G x 4" long hypodermic needle] and the resulting solution was left to stir for an additional 15 h naturally allowing the reaction to warm to 25°C without replenishing the cooling bath. The reaction was then filtered through celite with CH_2Cl_2 and concentrated *in vacuo*. The crude residue was then purified via flash column chromatography, eluting 15% EtOAc in Hex. The collected fractions were concentrated *in vacuo* to afford 35.2 mg of a white solid in 70% yield.

^{1}H NMR (400 MHz, $CDCl_3$) δ 7.44 – 7.36 (m, 1H), 7.25 – 7.18 (m, 2H), 7.13 – 7.06 (m, 1H), 4.48 (ddd, *J* = 12.4, 7.4, 2.3 Hz, 1H), 4.32 (ddd, *J* = 12.4, 7.9, 2.1 Hz, 1H), 3.01 (dd, *J* = 9.1, 7.2 Hz, 1H), 1.92 – 1.74 (m, 2H), 1.71 (ddd, *J* = 9.2, 5.1, 0.9 Hz, 1H), 1.64 – 1.55 (m, 2H), 1.50 – 1.35 (m, 2H), 1.29 (dd, *J* = 7.2, 5.1 Hz, 1H), 1.22 – 1.12 (m, 1H).

^{13}C NMR (101 MHz, $CDCl_3$) δ 176.41, 136.58, 134.95, 129.70, 129.62, 128.40, 126.76, 69.11, 30.23, 29.07, 28.90, 27.05, 25.34, 18.21.

HRMS (+p APCI) calculated for $C_{14}H_{16}ClO_2$ (M+H) 251.0841, found 251.0833

Chiral HPLC: Sample dissolved in 80:20 Hex:IPA
AS-H column, 1.0 mL/min, 10% IPA in Hex, 60 min – 99% *ee*
Retention times: major 26.61 min, minor 34.41 min.

$[\alpha]^{21.7}_D$ = -15.7° (c = 0.84, $CHCl_3$, 99% *ee of E-diastereomer*)

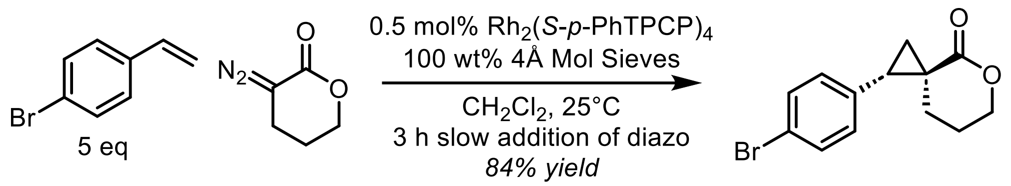

(1*S*,3*R*)-1-(4-bromophenyl)-5-oxaspiro[2.5]octan-4-one (16a)
Synthesized following **GP-1**. 4-Bromostyrene was filtered through a silica plug to remove inhibitor into a flame-dried 4 mL vial and bubbled with argon for 5 min before use. Absolute stereochemistry assigned by analogy to **24a**. A racemic sample was made following the same procedure but using 1 mol% $Rh_2(OAc)_4$.

4-Bromostyrene: 5 eq, 1.0 mmol, 183 mg
Reaction time: 22 h

The crude residue was dry-loaded onto silica gel and purified via automated flash column chromatography, eluting on a gradient. Fractions containing product were combined and concentrated *in vacuo*, to obtain 47 mg of a clear oil in 84% yield. *E* and *Z* diastereomers collected together.

Purification gradient:
0% EtOAc in Hex [5 CV] → 0 to 30% EtOAc in Hex [20 CV] → 30% EtOAc in Hex [5 CV]

^{1}H NMR (400 MHz, $CDCl_3$) δ 7.49 – 7.41 (m, 2H), 7.11 – 7.02 (m, 2H), 4.48 – 4.33 (m, 2H), 2.93 (dd, *J* = 9.2, 7.3 Hz, 1H), 1.92 (dd, *J* = 9.2, 4.7 Hz, 1H), 1.89 – 1.66 (m, 2H), 1.57 – 1.49 (m, 1H), 1.34 (dddd, *J* = 14.0, 7.7, 4.1, 1.0 Hz, 1H), 1.22 (dd, *J* = 7.2, 4.7 Hz, 1H).

^{13}C NMR (101 MHz, $CDCl_3$) δ 174.0, 135.0, 131.7, 130.9, 121.2, 70.4, 33.7, 26.1, 24.9, 23.1, 21.5.

HRMS (+p APCI) calculated for $C_{13}H_{14}BrO_2$ (M+H) 281.0179, found 281.0170

Chiral HPLC: Sample dissolved in 80:20 Hex:IPA
AD-H column, 1.0 mL/min, 0.5% IPA in Hex, 90 min – 84% *ee*
Retention times: major 63.89 min, minor 80.49 min.

$[\alpha]^{21.9}_{D}$ = -72.8° (c = 1.55, $CHCl_3$, 84% *ee of E-diastereomer*)

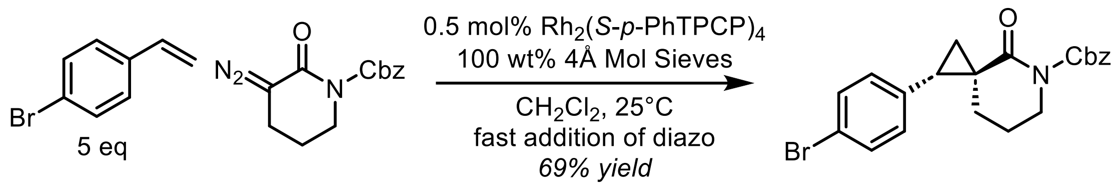

Benzyl (1*S*,3*R*)-1-(4-bromophenyl)-4-oxo-5-azaspiro[2.5]octane-5-carboxylate (16b)

Synthesized following **GP-2**. 4-Bromostyrene was filtered through a silica plug to remove inhibitor into a flame-dried 4 mL vial and bubbled with argon for 5 min before use. Absolute stereochemistry assigned by analogy to **24a**. A racemic sample was made following the same procedure but using 1 mol% $Rh_2(OAc)_4$.

4-Bromostyrene: 5 eq, 1.0 mmol, 183 mg
Reaction time: 4 h

The crude residue was dry-loaded onto silica gel and purified via automated flash column chromatography, eluting on a gradient. Fractions containing product were combined and concentrated *in vacuo*, to obtain 57.2 mg of a clear oil in 69% yield. *E* and *Z* diastereomers collected together.

Purification gradient:
0% EtOAc in Hex [5 CV] → 0 to 30% EtOAc in Hex [20 CV] → 30% EtOAc in Hex [5 CV]

^{1}H NMR (400 MHz, $CDCl_3$) δ 7.44 (td, *J* = 7.5, 1.9 Hz, 4H), 7.41 – 7.35 (m, 2H), 7.35 (s, 1H), 7.08 – 7.03 (m, 2H), 5.30 (s, 2H), 3.79 (t, *J* = 6.1 Hz, 2H), 2.93 (dd, *J* = 9.1, 7.2 Hz, 1H), 1.90 (dd, *J* = 9.2, 4.5 Hz, 1H), 1.83 – 1.61 (m, 2H), 1.51 – 1.44 (m, 1H), 1.30 (ddd, *J* = 13.9, 7.9, 3.6 Hz, 1H), 1.18 (dd, *J* = 7.2, 4.5 Hz, 1H).

^{13}C NMR (101 MHz, $CDCl_3$) δ 173.1, 154.1, 135.7, 135.6, 131.6, 130.9, 128.7, 128.5, 128.3, 121.0, 68.7, 47.7, 33.5, 30.1, 25.5, 22.1, 21.2.

HRMS (+p APCI) calculated for $C_{21}H_{21}BrNO_3$ (M+H) 414.0707, found 414.0697

Chiral HPLC: Sample dissolved in 80:20 Hex:IPA
AS-H column, 1.0 mL/min, 7.5% IPA in Hex, 30 min – 97% *ee*
Retention times: major 17.15 min, minor 20.08 min.

$[\alpha]^{22.2}_D$ = -124.4° (c = 1.52, $CHCl_3$, 97% *ee of E-diastereomer*)

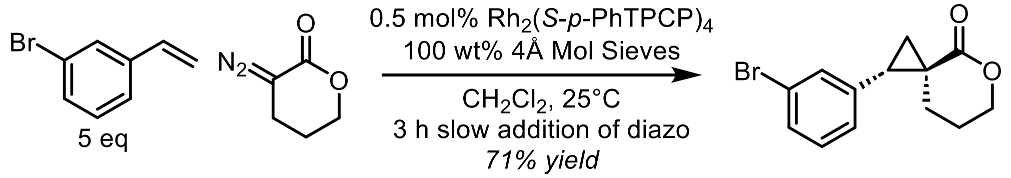

(1*S*,3*R*)-1-(3-Bromophenyl)-5-oxaspiro[2.5]octan-4-one (17a)
Synthesized following **GP-1**. 3-Bromostyrene was filtered through a silica plug to remove inhibitor into a flame-dried 4 mL vial and bubbled with argon for 5 min before use. Absolute stereochemistry assigned by analogy to **24a**. A racemic sample was made following the same procedure but using 1 mol% $Rh_2(OAc)_4$.

3-Bromostyrene: 5 eq, 1.0 mmol, 183 mg
Reaction time: 1 h

The crude residue was dry-loaded onto silica gel and purified via automated flash column chromatography, eluting on a gradient. Fractions containing product were combined and concentrated *in vacuo*, to obtain 40.1 mg of a clear oil in 71% yield. *E* and *Z* diastereomers collected together.

Purification gradient:
20% Et_2O in Hex [5 CV] → 20 to 70% Et_2O in Hex [20 CV] → 70% Et_2O in Hex [5 CV]

^{1}H NMR (400 MHz, $CDCl_3$) δ 7.40 (dt, *J* = 7.9, 1.6 Hz, 1H), 7.33 (t, *J* = 1.9 Hz, 1H), 7.20 (t, *J* = 7.8 Hz, 1H), 7.16 – 7.10 (m, 1H), 4.49 – 4.34 (m, 2H), 2.96 (dd, *J* = 9.2, 7.2 Hz, 1H), 1.91 (dd, *J* = 9.2, 4.7 Hz, 1H), 1.89 – 1.70 (m, 2H), 1.54 (dddd, *J* = 13.4, 8.4, 4.4, 0.8 Hz, 1H), 1.38 (dddd, *J* = 14.0, 7.9, 4.1, 1.0 Hz, 1H), 1.24 (dd, *J* = 7.2, 4.7 Hz, 1H).

^{13}C NMR (101 MHz, $CDCl_3$) δ 174.0, 138.5, 132.2, 130.4, 130.1, 128.1, 122.7, 70.4, 33.7, 26.2, 25.0, 23.1, 21.6.

HRMS (+p APCI) calculated for $C_{13}H_{14}BrO_2$ (M+H) 281.0179, found 281.0170

Chiral HPLC: Sample dissolved in 80:20 Hex:IPA
OD-H column, 1.0 mL/min, 3.0% IPA in Hex, 30 min – 94% *ee*
Retention times: major 21.99 min, minor 25.18 min.

$[\alpha]^{22.2}_D$ = -62.8° (c = 1.40, $CHCl_3$, 94% *ee of E-diastereomer*)

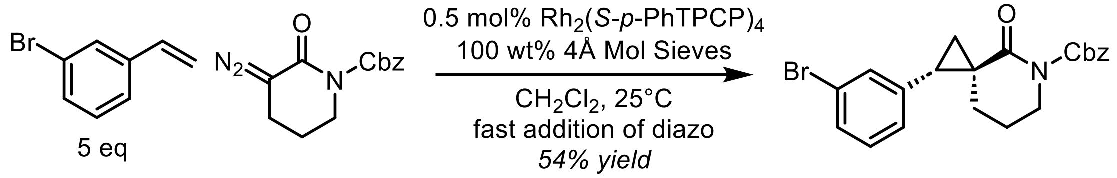

Benzyl (1*S*,3*R*)-1-(3-bromophenyl)-4-oxo-5-azaspiro[2.5]octane-5-carboxylate (17b)
Synthesized following **GP-2**. 3-Bromostyrene was filtered through a silica plug to remove inhibitor into a flame-dried 4 mL vial and bubbled with argon for 5 min before use. Absolute stereochemistry assigned by analogy to **24a**. A racemic sample was made following the same procedure but using 1 mol% $Rh_2(OAc)_4$.

3-Bromostyrene: 5 eq, 1.0 mmol, 183 mg
Reaction time: 4 h

The crude residue was dry-loaded onto silica gel and purified via automated flash column chromatography, eluting on a gradient. Fractions containing product were combined and concentrated *in vacuo*, to obtain 44.6 mg of a clear oil in 54% yield. *E* and *Z* diastereomers collected together.

Purification gradient:
10% Et_2O in Hex [5 CV] → 10 to 40% Et_2O in Hex [20 CV] → 40% Et_2O in Hex [5 CV]

^{1}H NMR (400 MHz, $CDCl_3$) δ 7.47 – 7.43 (m, 2H), 7.41 – 7.30 (m, 5H), 7.18 (t, *J* = 7.7 Hz, 1H), 7.15 – 7.10 (m, 1H), 5.30 (s, 2H), 3.87 – 3.73 (m, 2H), 2.96 (dd, *J* = 9.1, 7.2 Hz, 1H), 1.89 (dd, *J* = 9.1, 4.5 Hz, 1H), 1.85 – 1.63 (m, 2H), 1.47 (ddd, *J* = 13.9, 8.7, 3.6 Hz, 1H), 1.34 (ddd, *J* = 13.9, 8.1, 3.6 Hz, 1H), 1.19 (dd, *J* = 7.2, 4.6 Hz, 1H).

^{13}C NMR (101 MHz, $CDCl_3$) δ 173.0, 154.1, 139.1, 135.6, 132.2, 130.2, 130.0, 128.7, 128.5, 128.3, 128.1, 122.6, 68.7, 47.8, 33.4, 30.2, 25.7, 22.1, 21.3.

HRMS (+p APCI) calculated for $C_{21}H_{21}BrNO_3$ (M+H) 414.0707, found 414.0697

Chiral HPLC: Sample dissolved in 80:20 Hex:IPA
AS-H column, 1.0 mL/min, 5.0% IPA in Hex, 30 min – 98% *ee*
Retention times: major 24.17 min, minor 20.56 min.

$[\alpha]^{21.6}_D$ = -123.0° (c = 1.63, $CHCl_3$, 98% *ee of E-diastereomer*)

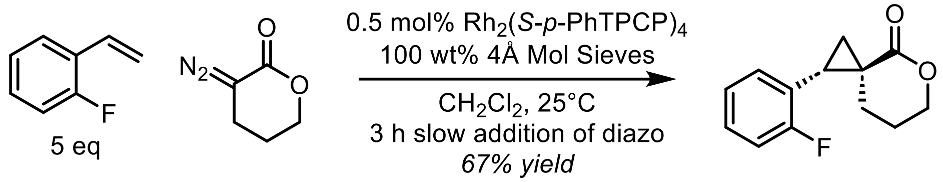

(1*S*,3*R*)-1-(2-Fluorophenyl)-5-oxaspiro[2.5]octan-4-one (18a)

Synthesized following **GP-1**. 2-Fluorostyrene was filtered through a silica plug to remove inhibitor into a flame-dried 4 mL vial and bubbled with argon for 5 min before use. Absolute stereochemistry assigned by analogy to **24a**. A racemic sample was made following the same procedure but using 1 mol% $Rh_2(OAc)_4$.

2-Fluorostyrene: 5.0 eq, 1.0 mmol, 122 mg, 119 µL
Reaction time: 1 h

The crude residue was dry-loaded onto silica gel and purified via automated flash column chromatography, eluting on a gradient. Fractions containing product were combined and concentrated *in vacuo*, to obtain 29.3 mg of a clear oil in 67% yield. *E* and *Z* diastereomers collected together.

Purification gradient:
0% Et_2O in Hex [5 CV] → 0 to 50% Et_2O in Hex [20 CV] → 50% Et_2O in Hex [5 CV]

^{1}H NMR (400 MHz, $CDCl_3$) δ 7.31 – 7.22 (m, 1H), 7.13 – 7.03 (m, 3H), 4.44 – 4.39 (m, 2H), 2.99 – 2.90 (m, 1H), 1.96 (ddd, *J* = 9.1, 4.6, 0.8 Hz, 1H), 1.87 – 1.77 (m, 2H), 1.53 (dt, *J* = 13.1, 6.4 Hz, 1H), 1.31 (dt, *J* = 12.2, 5.0 Hz, 1H), 1.23 (dd, *J* = 7.4, 4.7 Hz, 1H).

^{19}F NMR (376 MHz, $CDCl_3$) δ -114.95 (dt, *J* = 11.2, 5.8 Hz, 1F).

^{13}C NMR (101 MHz, $CDCl_3$) δ 174.1, 162.8 (d, *J* = 247.4 Hz), 130.3 (d, *J* = 3.7 Hz), 129.1 (d, *J* = 8.1 Hz), 124.1 (d, *J* = 3.7 Hz), 123.7 (d, *J* = 15.0 Hz), 115.5 (d, *J* = 21.5 Hz), 70.4, 31.3, 28.9 (d, *J* = 3.8 Hz), 25.2, 23.1, 20.9.

HRMS (+p APCI) calculated for $C_{13}H_{14}FO_2$ (M+H) 221.0980, found 221.0970

Chiral HPLC: Sample dissolved in 80:20 Hex:IPA
AD-H column, 1.0 mL/min, 0.5% IPA in Hex, 90 min – 93% *ee*
Retention times: major 62.17 min, minor 54.86 min.

4.2:1 mixture of diastereomers (E:Z)
$[\alpha]^{22.4}_D$ = -39.5° (c = 0.93, $CHCl_3$, 93% *ee of E-diastereomer*)

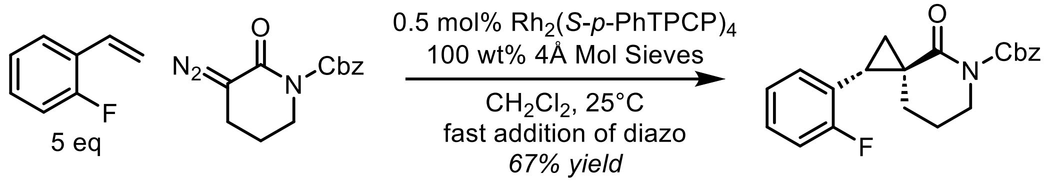

Benzyl (1*S*,3*R*)-1-(2-fluorophenyl)-4-oxo-5-azaspiro[2.5]octane-5-carboxylate (18b)
Synthesized following **GP-2**. 2-Fluorostyrene was filtered through a silica plug to remove inhibitor into a flame-dried 4 mL vial and bubbled with argon for 5 min before use. Absolute stereochemistry assigned by analogy to **24a**. A racemic sample was made following the same procedure but using 1 mol% $Rh_2(OAc)_4$.

2-Fluorostyrene: 5.0 eq, 1.0 mmol, 122 mg, 119 µL
Reaction time: 5 h

The crude residue was dry-loaded onto silica gel and purified via automated flash column chromatography, eluting on a gradient. Fractions containing product were combined and concentrated *in vacuo*, to obtain 58.8 mg of a clear oil in 67% yield. *E* and *Z* diastereomers collected together.

Purification gradient:
0% EtOAc in Hex [5 CV] → 0 to 30% EtOAc in Hex [20 CV] → 30% EtOAc in Hex [5 CV]

^{1}H NMR (400 MHz, $CDCl_3$) δ 7.51 – 7.45 (m, 2H), 7.40 (ddt, *J* = 7.9, 6.3, 1.1 Hz, 2H), 7.38 – 7.34 (m, 1H), 7.34 – 7.30 (m, 1H), 7.13 – 7.08 (m, 2H), 7.08 – 7.04 (m, 1H), 5.33 (s, 2H), 3.88 – 3.80 (m, 2H), 2.98 (dd, *J* = 9.1, 7.3 Hz, 1H), 1.97 (dd, *J* = 9.2, 4.4 Hz, 1H), 1.78 (dddd, *J* = 12.6, 8.1, 6.2, 3.0 Hz, 2H), 1.51 (ddd, *J* = 13.1, 8.4, 4.4 Hz, 1H), 1.34 – 1.26 (m, 1H), 1.26 – 1.17 (m, 1H).

^{19}F NMR (376 MHz, $CDCl_3$) δ -114.77 – -114.92 (m, 1F).

^{13}C NMR (101 MHz, $CDCl_3$) δ 173.1, 154.2, 135.7, 130.2 (d, *J* = 3.8 Hz), 128.9 (d, *J* = 8.1 Hz), 128.7, 128.5, 128.4, 128.3, 127.9, 124.0 (d, *J* = 3.6 Hz), 115.4 (d, *J* = 21.5 Hz), 68.6, 47.7, 29.5, 28.6 (d, *J* = 3.8 Hz), 25.8, 22.0, 20.6.

HRMS (+p APCI) calculated for $C_{21}H_{21}FNO_3$ (M+H) 354.1507, found 354.1496

Chiral HPLC: Sample dissolved in 80:20 Hex:IPA
AD-H column, 1.0 mL/min, 0.5% IPA in Hex, 90 min – 94% *ee*
Retention times: major 69.53 min, minor 50.55 min.

3.7:1 mixture of diastereomers (E:Z)
$[\alpha]^{22.4}_D$ = -60.8° (c = 0.87, $CHCl_3$, 94% *ee of E-diastereomer*)

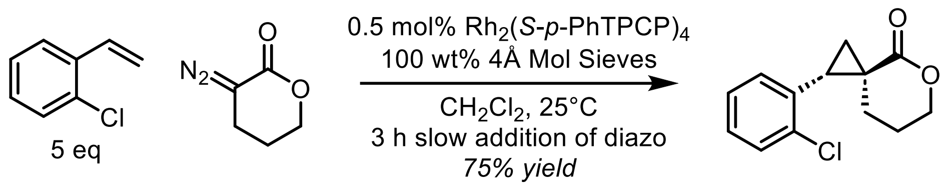

(1*S*,3*R*)-1-(2-Chlorophenyl)-5-oxaspiro[2.5]octan-4-one (19a)

Synthesized following **GP-1**. 2-Chlorostyrene was filtered through a silica plug to remove inhibitor into a flame-dried 4 mL vial and bubbled with argon for 5 min before use. Absolute stereochemistry assigned by analogy to **24a**. A racemic sample was made following the same procedure but using 1 mol% $Rh_2(OAc)_4$.

2-Chlorostyrene: 5.0 eq, 1.0 mmol, 139 mg, 128 μL
Reaction time: 30 min

The crude residue was dry-loaded onto silica gel and purified via automated flash column chromatography, eluting on a gradient. Fractions containing product were combined and concentrated *in vacuo*, to obtain 35.5 mg of a clear oil in 75% yield. *E* and *Z* diastereomers collected together.

Purification gradient:
0% EtOAc in Hex [5 CV] → 0 to 30% EtOAc in Hex [20 CV] → 30% EtOAc in Hex [5 CV]

^{1}H NMR (400 MHz, $CDCl_3$) δ 7.45 – 7.39 (m, 1H), 7.26 – 7.22 (m, 2H), 7.14 – 7.10 (m, 1H), 4.43 (t, *J* = 5.5 Hz, 2H), 3.06 – 2.97 (m, 1H), 1.98 (dd, *J* = 9.1, 4.5 Hz, 1H), 1.91 – 1.79 (m, 2H), 1.39 (ddd, *J* = 13.8, 7.0, 4.9 Hz, 1H), 1.29 – 1.25 (m, 2H).

^{13}C NMR (101 MHz, $CDCl_3$) δ 174.1, 136.8, 134.6, 129.8, 129.6, 128.7, 126.8, 70.4, 33.4, 25.4, 25.3, 23.3, 22.3.

HRMS (+p APCI) calculated for $C_{13}H_{14}ClO_2$ (M+H) 237.0684, found 237.0676

Chiral HPLC: Sample dissolved in 80:20 Hex:IPA
OD-H column, 1.0 mL/min, 1.0% IPA in Hex, 60 min – 88% *ee*
Retention times: major 38.70 min, minor 44.40 min.

2.8:1 mixture of diastereomers (E:Z)
$[\alpha]^{22.4}_D$ = 6.6° (c = 0.80, $CHCl_3$, 88% *ee of E-diastereomer*)

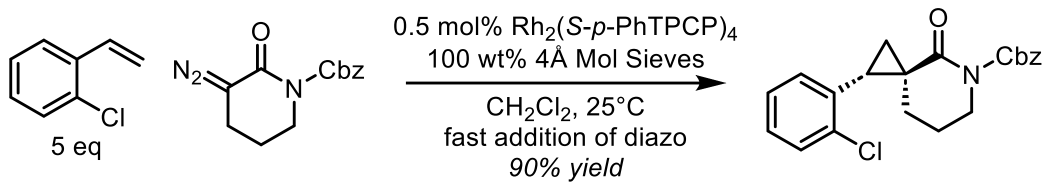

Benzyl (1*S*,3*R*)-1-(2-chlorophenyl)-4-oxo-5-azaspiro[2.5]octane-5-carboxylate (19b)
Synthesized following **GP-2**. 2-Chlorostyrene was filtered through a silica plug to remove inhibitor into a flame-dried 4 mL vial and bubbled with argon for 5 min before use. Absolute stereochemistry assigned by analogy to **24a**. A racemic sample was made following the same procedure but using 1 mol% $Rh_2(OAc)_4$.

2-Chlorostyrene: 5.0 eq, 1.0 mmol, 139 mg, 128 μL
Reaction time: 4 h

The crude residue was dry-loaded onto silica gel and purified via automated flash column chromatography, eluting on a gradient. Fractions containing product were combined and concentrated *in vacuo*, to obtain 66.7 mg of a clear oil in 90% yield. *E* and *Z* diastereomers collected together.

Purification gradient:
0% EtOAc in Hex [5 CV] → 0 to 30% EtOAc in Hex [20 CV] → 30% EtOAc in Hex [5 CV]

^{1}H NMR (400 MHz, CDCl$_3$) δ 7.48 – 7.43 (m, 2H), 7.42 – 7.35 (m, 3H), 7.35 – 7.31 (m, 1H), 7.22 (dd, *J* = 5.7, 3.6 Hz, 2H), 7.14 – 7.08 (m, 1H), 5.31 (s, 2H), 3.81 (t, *J* = 6.1 Hz, 2H), 3.04 (dd, *J* = 9.0, 7.4 Hz, 1H), 1.95 (dd, *J* = 9.0, 4.4 Hz, 1H), 1.79 (dddd, *J* = 12.3, 10.6, 4.6, 3.2 Hz, 2H), 1.35 (ddd, *J* = 13.8, 7.6, 4.4 Hz, 1H), 1.25 (ddd, *J* = 16.4, 7.4, 4.6 Hz, 2H).

^{13}C NMR (101 MHz, CDCl$_3$) δ 173.0, 154.3, 136.7, 135.7, 135.1, 129.7, 129.5, 128.7, 128.5, 128.4, 128.2, 126.7, 68.6, 47.7, 33.0, 29.5, 26.1, 22.2, 21.8.

HRMS (+p APCI) calculated for $C_{21}H_{21}ClNO_3$ (M+H) 370.1212, found 370.1202

Chiral HPLC: Sample dissolved in 80:20 Hex:IPA
AD-H column, 1.0 mL/min, 5.0% IPA in Hex, 30 min – 96% ee
Retention times: major 20.15 min, minor 15.76 min.

2.3:1 mixture of diastereomers (E:Z)
$[\alpha]^{22.3}_D$ = -0.5° (c = 1.54, $CHCl_3$, 96% *ee of E-diastereomer*)

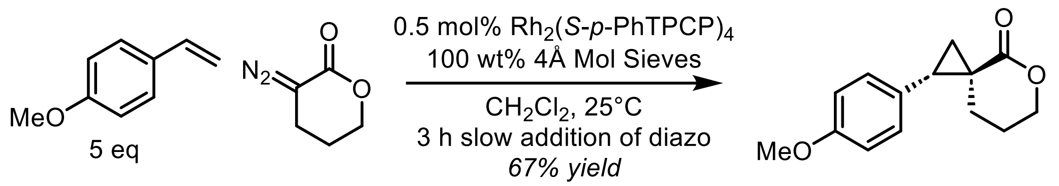

(1*S*,3*R*)-1-(4-Methoxyphenyl)-5-oxaspiro[2.5]octan-4-one (20a)
Synthesized following **GP-1**. 4-Methoxystyrene was filtered through a silica plug to remove inhibitor into a flame-dried 4 mL vial and bubbled with argon for 5 min before use. Absolute stereochemistry assigned by analogy to **24a**. A racemic sample was made following the same procedure but using 1 mol% $Rh_2(OAc)_4$.

4-Methoxystyrene: 5.0 eq, 1.0 mmol, 134 mg, 134 µL
Reaction time: 10 min

The crude residue was dry-loaded onto silica gel and purified via automated flash column chromatography, eluting on a gradient. Fractions containing product were combined and concentrated *in vacuo*, to obtain 31.3 mg of a clear oil in 67% yield. *E* and *Z* diastereomers collected together.

Purification gradient:
0% EtOAc in Hex [5 CV] → 0 to 30% EtOAc in Hex [20 CV] → 30% EtOAc in Hex [5 CV]

^{1}H NMR (400 MHz, $CDCl_3$) δ 7.15 – 7.07 (m, 2H), 6.90 – 6.82 (m, 2H), 4.39 (tt, *J* = 5.3, 1.5 Hz, 2H), 3.80 (s, 3H), 2.92 (dd, *J* = 9.3, 7.3 Hz, 1H), 1.91 (dd, *J* = 9.3, 4.6 Hz, 1H), 1.77 (dddt, *J* = 17.7, 9.0, 7.3, 4.3 Hz, 2H), 1.54 (ddd, *J* = 13.2, 8.7, 4.3 Hz, 1H), 1.40 – 1.29 (m, 1H), 1.20 (dd, *J* = 7.3, 4.6 Hz, 1H).

^{13}C NMR (101 MHz, $CDCl_3$) δ 174.6, 158.8, 130.3, 127.8, 113.9, 70.4, 55.4, 34.2, 25.9, 24.8, 23.2, 21.5.

HRMS (+p APCI) calculated for $C_{14}H_{17}O_3$ (M+H) 233.1179, found 233.1166

Chiral HPLC: Sample dissolved in 80:20 Hex:IPA
OD-H column, 1.0 mL/min, 7.5% IPA in Hex, 30 min – 94% *ee*
Retention times: major 14.19 min, minor 16.23 min.

$[\alpha]^{22.4}_D$ = -73.4° (c = 1.04, $CHCl_3$, 94% *ee of E-diastereomer*)

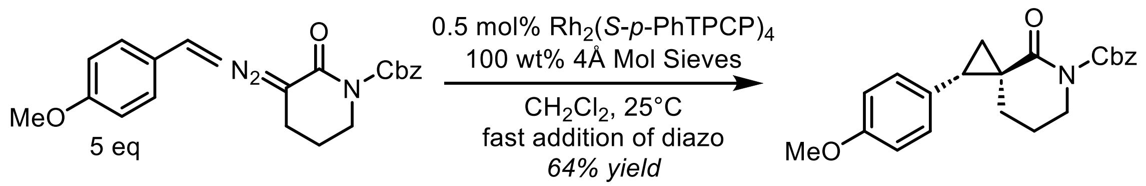

Benzyl (1*S*,3*R*)-1-(4-methoxyphenyl)-4-oxo-5-azaspiro[2.5]octane-5-carboxylate (20b)
Synthesized following **GP-2**. 4-Methoxystyrene was filtered through a silica plug to remove inhibitor into a flame-dried 4 mL vial and bubbled with argon for 5 min before use. Absolute stereochemistry assigned by analogy to **24a**. A racemic sample was made following the same procedure but using 1 mol% $Rh_2(OAc)_4$.

4-Methoxystyrene: 5.0 eq, 1.0 mmol, 134 mg, 134 μL
Reaction time: 1.5 h

The crude residue was dry-loaded onto silica gel and purified via automated flash column chromatography, eluting on a gradient. Fractions containing product were combined and concentrated *in vacuo*, to obtain 46.6 mg of a clear oil in 64% yield. *E* and *Z* diastereomers collected together.

Purification gradient:
0% EtOAc in Hex [5 CV] → 0 to 30% EtOAc in Hex [20 CV] → 30% EtOAc in Hex [5 CV]

^{1}H NMR (400 MHz, $CDCl_3$) δ 7.48 – 7.43 (m, 2H), 7.41 – 7.30 (m, 3H), 7.13 – 7.07 (m, 2H), 6.88 – 6.80 (m, 2H), 5.30 (s, 2H), 3.80 (s, 3H), 2.92 (dd, *J* = 9.2, 7.2 Hz, 1H), 1.89 (dd, *J* = 9.3, 4.4 Hz, 1H), 1.81 – 1.62 (m, 2H), 1.47 (ddd, *J* = 13.0, 9.2, 3.6 Hz, 1H), 1.30 (ddd, *J* = 13.9, 7.6, 3.6 Hz, 1H), 1.16 (dd, *J* = 7.2, 4.4 Hz, 1H).

^{13}C NMR (101 MHz, $CDCl_3$) δ 173.6, 158.7, 154.3, 135.7, 130.3, 128.7, 128.4, 128.3, 113.8, 68.6, 55.4, 47.8, 33.9, 30.0, 25.4, 22.1, 21.2.

HRMS (+p APCI) calculated for $C_{22}H_{24}NO_4$ (M+H) 366.1707, found 366.1695

Chiral HPLC: Sample dissolved in 80:20 Hex:IPA
AD-H column, 1.0 mL/min, 5.0% IPA in Hex, 45 min – 93% ee
Retention times: major 37.61 min, minor 32.62 min.

$[\alpha]^{21.7}_D$ = -151.9° (c = 0.90, $CHCl_3$, 93% *ee of E-diastereomer*)

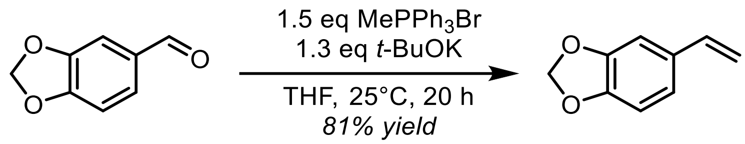

5-vinylbenzo[d][1,3]dioxole (SI-3)

To a flame-dried 100 mL RBF equipped with a stir bar was added methyltriphenylphosphonium bromide (1.5 eq, 15.0 mmol, 5.3585 g) and *t*-BuOK (1.3 eq, 13.0 mmol, 1.4587 g). This was degassed and backfilled with argon three times, left under an argon-filled balloon, then diluted with THF (0.25 M to aldehyde, 40.0 mL, SPS grade). The resulting solution was stirred at 25°C for 1 h. To this, a previously degassed solution of piperonal (1.0 eq, 10.0 mmol, 1.5013 g) in THF (1.0 M, to aldehyde, 10.00 mL, SPS grade) was added dropwise. The resulting solution was stirred at 25°C for 20 h. The reactions were then concentrated *in vacuo*, directly dry-loaded onto silica gel, then purified via automated flash column chromatography, eluting 100% Hex for 20 CV. Fractions containing product were combined and concentrated *in vacuo* to afford 1193.3 mg of a yellow oil in 81% yield. ^{1}H NMR matched the reported spectra.[18]

^{1}H NMR (400 MHz, $CDCl_3$) δ 6.97 (d, *J* = 1.7 Hz, 1H), 6.84 (dd, *J* = 8.0, 1.7 Hz, 1H), 6.76 (d, *J* = 8.0 Hz, 1H), 6.62 (dd, *J* = 17.5, 10.9 Hz, 1H), 5.96 (s, 2H), 5.58 (dd, *J* = 17.5, 0.8 Hz, 1H), 5.13 (dd, *J* = 10.9, 0.8 Hz, 1H).

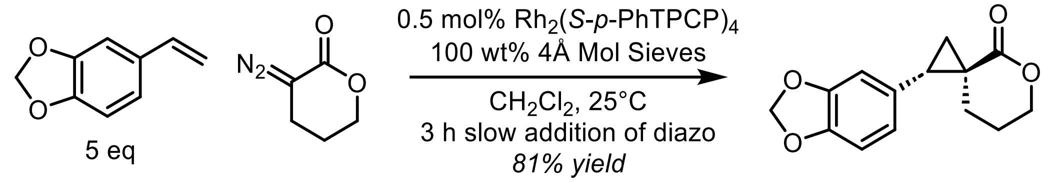

(1*S*,3*R*)-1-(Benzo[d][1,3]dioxol-5-yl)-5-oxaspiro[2.5]octan-4-one (21a)
Synthesized following **GP-1**. Absolute stereochemistry assigned by analogy to **24a**. A racemic sample was made following the same procedure but using 1 mol% $Rh_2(OAc)_4$.

SI-3: 5.0 eq, 1.0 mmol, 148 mg
Reaction time: 1 h

The crude residue was dry-loaded onto silica gel and purified via automated flash column chromatography, eluting on a gradient. Fractions containing product were combined and concentrated *in vacuo*, to obtain 39.9 mg of a clear oil in 81% yield. *E* and *Z* diastereomers collected together.

Purification gradient:
20% Et_2O in Hex [5 CV] → 20 to 70% Et_2O in Hex [20 CV] → 70% Et_2O in Hex [5 CV]

^{1}H NMR (400 MHz, $CDCl_3$) δ 6.76 (d, *J* = 7.9 Hz, 1H), 6.70 – 6.61 (m, 2H), 5.96 (s, 2H), 4.47 – 4.34 (m, 2H), 2.91 (dd, *J* = 9.2, 7.3 Hz, 1H), 1.89 (dd, *J* = 9.3, 4.6 Hz, 1H), 1.87 – 1.70 (m, 2H), 1.55 (ddd, *J* = 13.2, 8.6, 4.3 Hz, 1H), 1.44 – 1.34 (m, 1H), 1.16 (dd, *J* = 7.3, 4.7 Hz, 1H).

^{13}C NMR (101 MHz, $CDCl_3$) δ 174.4, 147.8, 146.9, 129.7, 122.5, 109.7, 108.3, 101.3, 70.4, 34.5, 26.0, 24.8, 23.2, 21.7.

HRMS (+p APCI) calculated for $C_{14}H_{15}O_4$ (M+H) 247.0972, found 247.0959

Chiral HPLC: Sample dissolved in 80:20 Hex:IPA
AD-H column, 1.0 mL/min, 1.0% IPA in Hex, 90 min – 91% *ee*
Retention times: major 78.30 min, minor 63.61 min.

$[\alpha]^{21.9}_D$ = -84.1° (c = 1.06, $CHCl_3$, 91% *ee of E-diastereomer*)

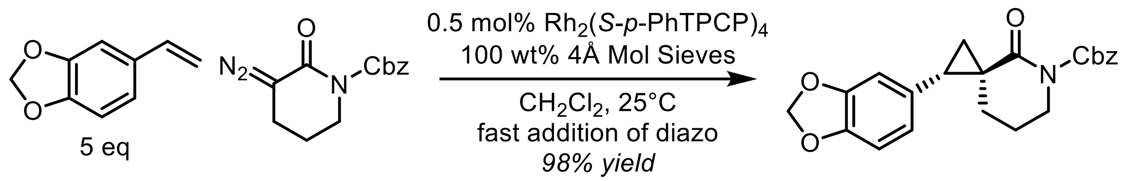

Benzyl (1*S*,3*R*)-1-(benzo[d][1,3]dioxol-5-yl)-4-oxo-5-azaspiro[2.5]octane-5-carboxylate (21b)
Synthesized following **GP-2**. Absolute stereochemistry assigned by analogy to **24a**. A racemic sample was made following the same procedure but using 1 mol% $Rh_2(OAc)_4$.

SI-3: 5.0 eq, 1.0 mmol, 148 mg
Reaction time: 3.5 h

The crude residue was dry-loaded onto silica gel and purified via automated flash column chromatography, eluting on a gradient. Fractions containing product were combined and concentrated *in vacuo*, to obtain 74.2 mg of a clear oil in 98% yield. *E* and *Z* diastereomers collected together.

Purification gradient:
0% EtOAc in Hex [5 CV] → 0 to 30% EtOAc in Hex [20 CV] → 30% EtOAc in Hex [5 CV]

^{1}H NMR (400 MHz, $CDCl_3$) δ 7.48 – 7.42 (m, 2H), 7.41 – 7.31 (m, 3H), 6.75 (d, *J* = 7.9 Hz, 1H), 6.69 – 6.64 (m, 2H), 5.94 (s, 2H), 5.29 (s, 2H), 3.79 (td, *J* = 5.5, 2.5 Hz, 2H), 2.90 (dd, *J* = 9.2, 7.2 Hz, 1H), 1.87 (dd, *J* = 9.2, 4.4 Hz, 1H), 1.83 – 1.64 (m, 2H), 1.48 (ddd, *J* = 13.0, 9.0, 3.6 Hz, 1H), 1.35 (ddd, *J* = 13.8, 7.7, 3.7 Hz, 1H), 1.12 (dd, *J* = 7.2, 4.5 Hz, 1H).

^{13}C NMR (101 MHz, $CDCl_3$) δ 173.5, 154.2, 147.8, 146.7, 135.6, 130.3, 128.7, 128.4, 128.3, 122.5, 109.7, 108.2, 101.2, 68.6, 47.8, 34.3, 30.1, 25.4, 22.2, 21.5.

HRMS (+p APCI) calculated for $C_{22}H_{22}NO_5$ (M+H) 380.1500, found 380.1488

Chiral HPLC: Sample dissolved in 80:20 Hex:IPA
AD-H column, 1.0 mL/min, 3.0% IPA in Hex, 60 min – 97% *ee*
Retention times: major 43.80 min, minor 38.76 min.

$[\alpha]^{22.5}_D$ = -83.6° (c = 1.93, $CHCl_3$, 97% *ee of E-diastereomer*)

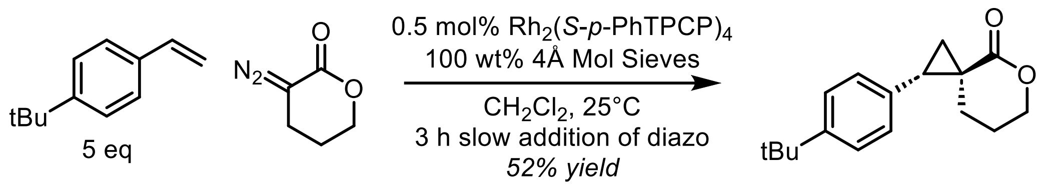

(1*S*,3*R*)-1-(4-(*tert*-Butyl)phenyl)-5-oxaspiro[2.5]octan-4-one (22a)

Synthesized following **GP-1**. 4-*tert*-Butylstyrene was filtered through a silica plug to remove inhibitor into a flame-dried 4 mL vial and bubbled with argon for 5 min before use. Absolute stereochemistry assigned by analogy to **24a**. A racemic sample was made following the same procedure but using 1 mol% $Rh_2(OAc)_4$.

4-*tert*-Butylstyrene: 5.0 eq, 1.0 mmol, 160 mg, 182 µL
Reaction time: 1 h

The crude residue was dry-loaded onto silica gel and purified via automated flash column chromatography, eluting on a gradient. Fractions containing product were combined and concentrated *in vacuo*, to obtain 26.7 mg of a clear oil in 52% yield. *E* and *Z* diastereomers collected together.

Purification gradient:
0% EtOAc in Hex [5 CV] → 0 to 25% EtOAc in Hex [20 CV] → 25% EtOAc in Hex [5 CV]

^{1}H NMR (400 MHz, $CDCl_3$) δ 7.37 – 7.29 (m, 2H), 7.15 – 7.07 (m, 2H), 4.47 – 4.33 (m, 2H), 2.95 (dd, *J* = 9.3, 7.3 Hz, 1H), 1.92 (dd, *J* = 9.3, 4.5 Hz, 1H), 1.88 – 1.70 (m, 2H), 1.54 (td, *J* = 8.9, 4.2 Hz, 1H), 1.44 – 1.35 (m, 1H), 1.31 (s, 9H), 1.24 (dd, *J* = 7.3, 4.6 Hz, 1H).

^{13}C NMR (101 MHz, $CDCl_3$) δ 174.6, 150.2, 132.8, 129.0, 125.4, 70.4, 34.6, 34.4, 31.5, 26.0, 24.9, 23.2, 21.6.

HRMS (+p APCI) calculated for $C_{17}H_{23}O_2$ (M+H) 259.1700, found 259.1693

Chiral HPLC: Sample dissolved in 80:20 Hex:IPA
OD column, 1.0 mL/min, 3.0% IPA in Hex, 30 min – 94% *ee*
Retention times: major 13.28 min, minor 17.82 min.

$[\alpha]^{22.1}_D$ = -76.5° (c = 0.58, $CHCl_3$, 94% *ee of E-diastereomer*)

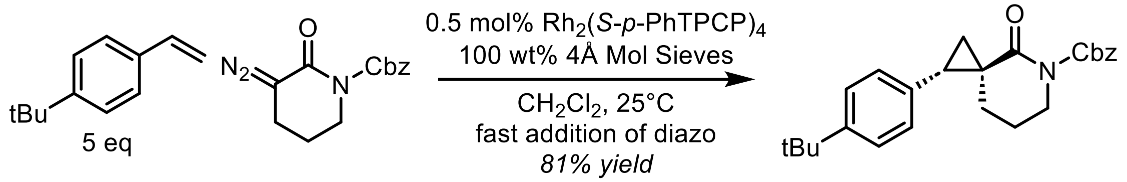

Benzyl (1*S*,3*R*)-1-(4-(*tert*-butyl)phenyl)-4-oxo-5-azaspiro[2.5]octane-5-carboxylate (22b)
Synthesized following **GP-2**. 4-*tert*-Butylstyrene was filtered through a silica plug to remove inhibitor into a flame-dried 4 mL vial and bubbled with argon for 5 min before use. Absolute stereochemistry assigned by analogy to **24a**. A racemic sample was made following the same procedure but using 1 mol% $Rh_2(OAc)_4$.

4-*tert*-Butylstyrene: 5.0 eq, 1.0 mmol, 160 mg, 182 µL
Reaction time: 4 h

The crude residue was dry-loaded onto silica gel and purified via automated flash column chromatography, eluting on a gradient. Fractions containing product were combined and concentrated *in vacuo*, to obtain 63.4 mg of a clear oil in 81% yield. *E* and *Z* diastereomers collected together.

Purification gradient:
0% EtOAc in Hex [5 CV] → 0 to 30% EtOAc in Hex [20 CV] → 30% EtOAc in Hex [5 CV]

^{1}H NMR (400 MHz, $CDCl_3$) δ 7.48 – 7.42 (m, 2H), 7.38 (ddt, *J* = 7.9, 6.3, 1.2 Hz, 2H), 7.35 – 7.29 (m, 3H), 7.15 – 7.07 (m, 2H), 5.30 (s, 2H), 3.79 (t, *J* = 6.1 Hz, 2H), 2.95 (dd, *J* = 9.2, 7.3 Hz, 1H), 1.89 (dd, *J* = 9.2, 4.3 Hz, 1H), 1.82 – 1.62 (m, 2H), 1.49 (ddd, *J* = 13.9, 8.9, 3.8 Hz, 1H), 1.40 – 1.32 (m, 1H), 1.31 (s, 9H), 1.20 (dd, *J* = 7.3, 4.4 Hz, 1H).

^{13}C NMR (101 MHz, $CDCl_3$) δ 173.7, 154.3, 149.9, 135.7, 133.3, 129.0, 128.7, 128.4, 128.3, 125.3, 68.6, 47.8, 34.6, 34.1, 31.5, 30.1, 25.5, 22.2, 21.3.

HRMS (+p APCI) calculated for $C_{25}H_{30}NO_3$ (M+H) 392.2227, found 392.2214

Chiral HPLC: Sample dissolved in 80:20 Hex:IPA
AS-H column, 1.0 mL/min, 3.0% IPA in Hex, 30 min – 80% *ee*
Retention times: major 19.97 min, minor 14.86 min.

$[\alpha]^{22.3}_D$ = -160.9° (c = 1.10, $CHCl_3$, 80% *ee of E-diastereomer*)

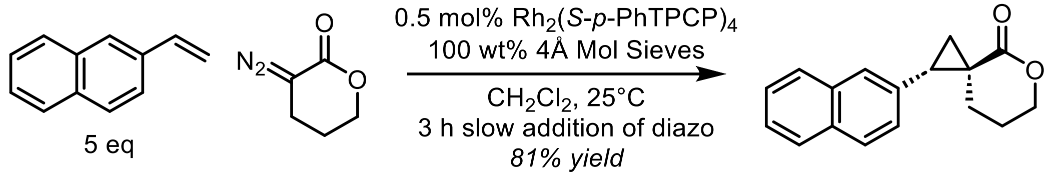

(1*S*,3*R*)-1-(Naphthalen-2-yl)-5-oxaspiro[2.5]octan-4-one (23a)

Synthesized following **GP-1**. Absolute stereochemistry assigned by analogy to **24a**. A racemic sample was made following the same procedure but using 1 mol% $Rh_2(OAc)_4$.

2-vinylnapthalene: 5.0 eq, 1.0 mmol, 154 mg
Reaction time: 30 min

The crude residue was dry-loaded onto silica gel and purified via automated flash column chromatography, eluting on a gradient. Fractions containing product were combined and concentrated *in vacuo*, to obtain 40.8 mg of a clear oil in 81% yield. *E* and *Z* diastereomers collected together.

Purification gradient:
0% EtOAc in Hex [5 CV] → 0 to 30% EtOAc in Hex [20 CV] → 30% EtOAc in Hex [5 CV]

^{1}H NMR (400 MHz, $CDCl_3$) δ 7.88 – 7.76 (m, 3H), 7.60 (d, *J* = 1.7 Hz, 1H), 7.54 – 7.43 (m, 2H), 7.36 (dd, *J* = 8.4, 1.8 Hz, 1H), 4.48 – 4.36 (m, 2H), 3.19 – 3.10 (m, 1H), 2.02 (dd, *J* = 9.2, 4.6 Hz, 1H), 1.85 – 1.67 (m, 2H), 1.57 (td, *J* = 8.9, 4.2 Hz, 1H), 1.42 (dd, *J* = 7.3, 4.7 Hz, 1H), 1.37 (ddd, *J* = 13.9, 7.3, 4.3 Hz, 1H).

^{13}C NMR (101 MHz, $CDCl_3$) δ 174.4, 133.6, 133.3, 132.7, 128.2, 127.8, 127.8, 127.5, 126.5, 126.1, 70.4, 34.9, 26.3, 24.8, 23.2, 21.4.

HRMS (+p APCI) calculated for $C_{17}H_{17}O_2$ (M+H) 253.1230, found 253.1220

Chiral HPLC: Sample dissolved in 80:20 Hex:IPA
OD-H column, 1.0 mL/min, 4.0% IPA in Hex, 30 min – 96% *ee*
Retention times: major 22.26 min, minor 25.84 min.

$[\alpha]^{22.4}_D$ = -145.9° (c = 0.80, $CHCl_3$, 96% *ee of E-diastereomer*)

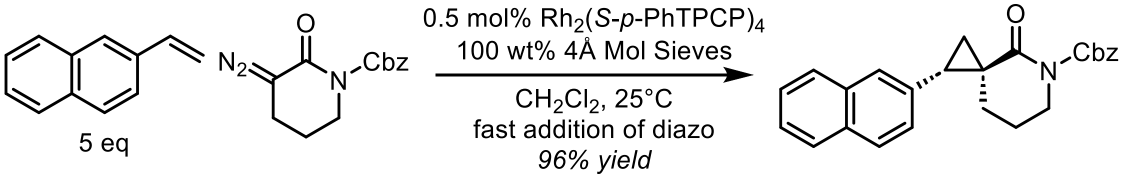

Benzyl (1*S*,3*R*)-1-(naphthalen-2-yl)-4-oxo-5-azaspiro[2.5]octane-5-carboxylate (23b)
Synthesized following **GP-2**. Absolute stereochemistry assigned by analogy to **24a**. A racemic sample was made following the same procedure but using 1 mol% $Rh_2(OAc)_4$.

2-vinylnapthalene: 5.0 eq, 1.0 mmol, 154 mg
Reaction time: 1.5 h

The crude residue was dry-loaded onto silica gel and purified via automated flash column chromatography, eluting on a gradient. Fractions containing product were combined and concentrated *in vacuo*, to obtain 82.4 mg of a clear oil in 96% yield. *E* and *Z* diastereomers collected together.

Purification gradient:
0% EtOAc in Hex [5 CV] → 0 to 30% EtOAc in Hex [20 CV] → 30% EtOAc in Hex [5 CV]

^{1}H NMR (400 MHz, $CDCl_3$) δ 7.85 – 7.75 (m, 3H), 7.59 (d, *J* = 1.7 Hz, 1H), 7.52 – 7.43 (m, 4H), 7.43 – 7.31 (m, 4H), 5.32 (s, 2H), 3.86 – 3.72 (m, 2H), 3.14 (dd, *J* = 8.9, 7.5 Hz, 1H), 2.00 (dd, *J* = 9.1, 4.4 Hz, 1H), 1.79 – 1.62 (m, 2H), 1.51 (ddd, *J* = 13.0, 9.0, 3.6 Hz, 1H), 1.38 (dd, *J* = 7.3, 4.6 Hz, 1H), 1.33 (ddd, *J* = 11.2, 6.0, 2.3 Hz, 1H).

^{13}C NMR (101 MHz, $CDCl_3$) δ 173.5, 154.2, 135.7, 134.2, 133.3, 132.6, 128.7, 128.4, 128.3, 128.1, 127.9, 127.8, 127.7, 127.5, 126.4, 125.9, 68.6, 47.8, 34.6, 30.5, 25.4, 22.2, 21.14.

HRMS (+p APCI) calculated for $C_{25}H_{24}NO_3$ (M+H) 386.1758, found 386.1742

Chiral HPLC: Sample dissolved in 80:20 Hex:IPA
AD-H column, 1.0 mL/min, 5.0% IPA in Hex, 30 min – 96% *ee*
Retention times: major 21.70 min, minor 17.08 min.

$[\alpha]^{22.4}_D$ = -240.7° (c = 0.90, $CHCl_3$, 96% *ee of E-diastereomer*)

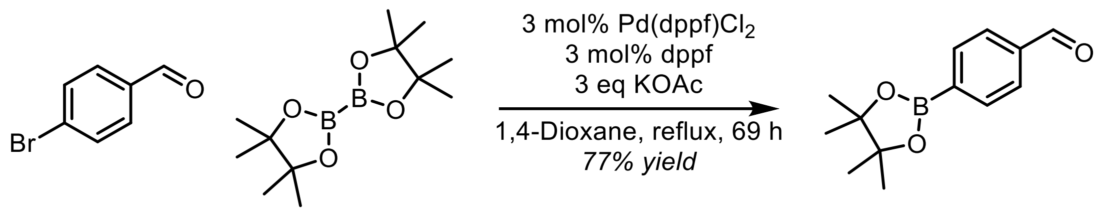

4-(4,4,5,5-tetramethyl-1,3,2-dioxaborolan-2-yl)benzaldehyde (SI-4)

To a flame-dried 100 mL RBF equipped with a stir bar was added 4-bromobenzaldehyde (1.0 eq, 10.0 mmol, 1.8502 g), Bis(pinacolato)diborane (1.2 eq, 12.0 mmol, 3.0473 g), Pd(dppf)Cl_2 (3 mol%, 0.3 mmol, 219.51 mg), dppf (3 mol%, 0.3 mmol, 166.32 mg), and KOAc (3.0 eq, 30.0 mmol, 2.944 g). This was degassed and backfilled with argon three times, left under an argon-filled balloon, then diluted with 1,4-dioxane (0.3 M to aldehyde, 33.3 mL, reagent grade). The resulting solution was refluxed via open-top findenser for 69 h. The reaction was then transferred to a 250 mL separatory funnel and diluted with 30 mL of brine. The aqueous phase was extracted with 30 mL of EtOAc three times. The combined organic phases were washed with 30 mL of brine, dried over $MgSO_4$, filtered, and concentrated *in vacuo*. The crude residue was dry-loaded onto silica gel, then purified via automated flash column chromatography, eluting on a gradient. Fractions containing product were combined and concentrated *in vacuo* to afford 1797.8 mg of a clear oil in 77% yield. ^{1}H NMR matched the reported spectra.[19]

Purification gradient:
0% EtOAc in Hex [5 CV] → 0 to 25% EtOAc in Hex [20 CV] → 25% EtOAc in Hex [5 CV]

^{1}H NMR (400 MHz, $CDCl_3$) δ 10.05 (s, 1H), 7.96 (d, *J* = 8.1 Hz, 2H), 7.86 (d, *J* = 8.3 Hz, 2H), 1.36 (s, 12H).

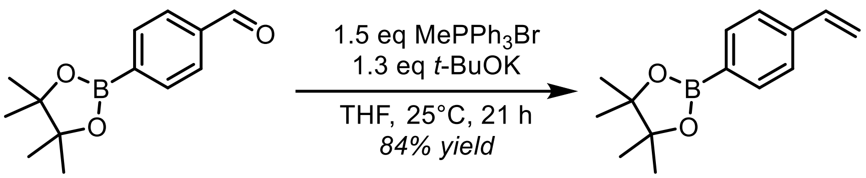

4,4,5,5-tetramethyl-2-(4-vinylphenyl)-1,3,2-dioxaborolane (SI-5)

To a flame-dried 100 mL RBF equipped with a stir bar was added methyltriphenylphosphonium bromide (1.5 eq, 11.6 mmol, 4.1507 g) and *t*-BuOK (1.3 eq, 10.1 mmol, 1.13 g). This was degassed and backfilled with argon three times, left under an argon-filled balloon, then diluted with THF (0.25 M to aldehyde, 30.0 mL, SPS grade). The resulting solution was stirred at 25°C for 1 h. To this, a previously degassed solution of **SI-4** (1.0 eq, 7.7 mmol, 1797.8 mg) in THF (1.0 M, to aldehyde, 10.0 mL, SPS grade) was added dropwise. The resulting solution was stirred at 25°C for 21 h. The reactions were then concentrated *in vacuo*, directly dry-loaded onto silica gel, then purified via automated flash column chromatography, eluting on a gradient. Fractions containing product were combined and concentrated *in vacuo* to afford 1504.2 mg of a white solid in 84% yield. ^{1}H NMR matched the reported spectra.[20]

Purification gradient:
0% Et_2O in Hex [5 CV] → 0 to 5% Et_2O in Hex [20 CV] → 5% Et_2O in Hex [5 CV]

^{1}H NMR (400 MHz, $CDCl_3$) δ 7.82 – 7.75 (m, 2H), 7.45 – 7.39 (m, 2H), 6.73 (dd, *J* = 17.6, 10.9 Hz, 1H), 5.82 (dd, *J* = 17.6, 1.0 Hz, 1H), 5.30 (dd, *J* = 10.9, 1.0 Hz, 1H), 1.35 (s, 12H).

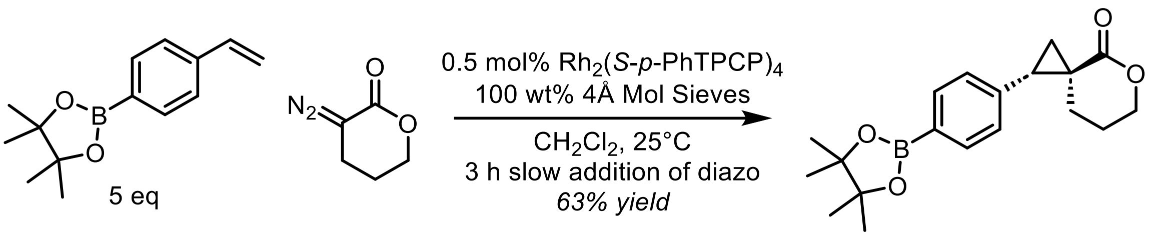

(1*S*,3*R*)-1-(4-(4,4,5,5-tetramethyl-1,3,2-dioxaborolan-2-yl)phenyl)-5-oxaspiro[2.5]octan-4-one (24a)
Synthesized following **GP-1**. Absolute stereochemistry was determined by X-ray crystallography. A racemic sample was made following the same procedure but using 1 mol% $Rh_2(OAc)_4$.

SI-5: 5.0 eq, 1.0 mmol, 230 mg
Reaction time: 2 h

The crude residue was dry-loaded onto silica gel and purified via automated flash column chromatography, eluting on a gradient. Fractions containing product were combined and concentrated *in vacuo*, to obtain 41.1 mg of a clear oil in 63% yield. *E* and *Z* diastereomers collected together.

Purification gradient:
20% Et_2O in Hex [5 CV] → 20 to 70% Et_2O in Hex [20 CV] → 70% Et_2O in Hex [5 CV]

^{1}H NMR (400 MHz, $CDCl_3$) δ 7.80 – 7.73 (m, 2H), 7.22 – 7.17 (m, 2H), 4.46 – 4.32 (m, 2H), 2.99 (dd, *J* = 9.2, 7.3 Hz, 1H), 1.93 (dd, *J* = 9.2, 4.6 Hz, 1H), 1.84 – 1.65 (m, 2H), 1.54 (ddt, *J* = 12.5, 7.9, 4.0 Hz, 1H), 1.34 (s, 12H), 1.30 (dd, *J* = 7.3, 4.6 Hz, 1H).

^{13}C NMR (101 MHz, $CDCl_3$) δ 174.3, 139.2, 134.9, 128.7, 84.0, 70.4, 34.7, 26.3, 25.0, 25.0, 24.8, 23.1, 21.2.

HRMS (+p APCI) calculated for $C_{19}H_{26}BO_4$ (M+H) 329.1926, found 329.1913

Chiral HPLC: Sample dissolved in 80:20 Hex:IPA
AS-H column, 1.0 mL/min, 7.5% IPA in Hex, 30 min – 94% *ee*
Retention times: major 20.75 min, minor 16.00 min.

$[\alpha]^{22.4}_D$ = -58.2° (c = 1.33, $CHCl_3$, 94% *ee of E-diastereomer*)

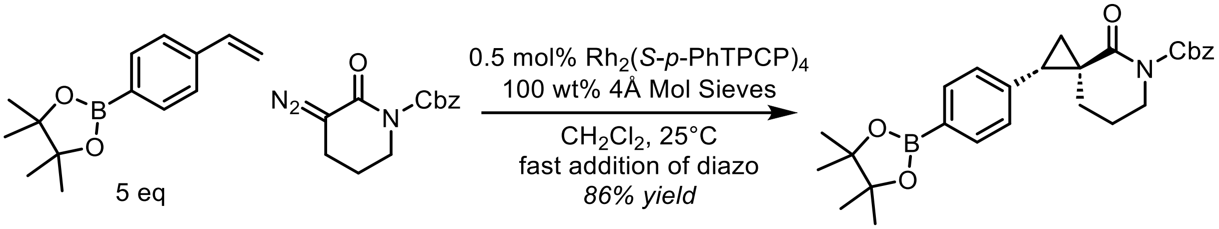

Benzyl (1*S*,3*R*)-4-oxo-1-(4-(4,4,5,5-tetramethyl-1,3,2-dioxaborolan-2-yl)phenyl)-5-azaspiro[2.5]octane-5-carboxylate (24b)

Synthesized following **GP-2**. Absolute stereochemistry assigned by analogy to **24a**. A racemic sample was made following the same procedure but using 1 mol% $Rh_2(OAc)_4$. See next page for details of determination of enantioselectivity.

SI-5: 5.0 eq, 1.0 mmol, 230 mg
Reaction time: 4 h

The crude residue was dry-loaded onto silica gel and purified via automated flash column chromatography, eluting on a gradient. Fractions containing product were combined and concentrated *in vacuo*, to obtain 79.7 mg of a clear oil in 86% yield. *E* and *Z* diastereomers collected together.

Purification gradient:
20% Et_2O in Hex [5 CV] → 20 to 70% Et_2O in Hex [20 CV] → 70% Et_2O in Hex [5 CV]

^{1}H NMR (400 MHz, $CDCl_3$) δ 7.77 – 7.72 (m, 2H), 7.48 – 7.42 (m, 2H), 7.41 – 7.30 (m, 3H), 7.21 – 7.16 (m, 2H), 5.30 (s, 2H), 3.83 – 3.72 (m, 2H), 2.99 (dd, *J* = 9.1, 7.3 Hz, 1H), 1.91 (dd, *J* = 9.1, 4.4 Hz, 1H), 1.79 – 1.59 (m, 2H), 1.48 (ddd, *J* = 12.9, 9.1, 3.6 Hz, 1H), 1.34 (s, 12H), 1.29 (dd, *J* = 6.2, 3.6 Hz, 1H), 1.26 (dd, *J* = 7.3, 4.5 Hz, 1H).

^{13}C NMR (101 MHz, $CDCl_3$) δ 173.4, 154.2, 139.8, 135.7, 134.9, 128.7, 128.7, 128.4, 128.3, 84.0, 68.6, 47.8, 34.5, 30.4, 25.4, 25.0, 25.0, 22.1, 21.0.

HRMS (+p APCI) calculated for $C_{27}H_{33}BNO_5$ (M+H) 462.2454, found 462.2437

Chiral HPLC: *Sample was not able to separate on chiral HPLC columns. Please see next page for procedure converting 24b to 16b.*

$[\alpha]^{22.3}_D$ = -124.8° (c = 0.58, $CHCl_3$, 97% *ee of E-diastereomer*)

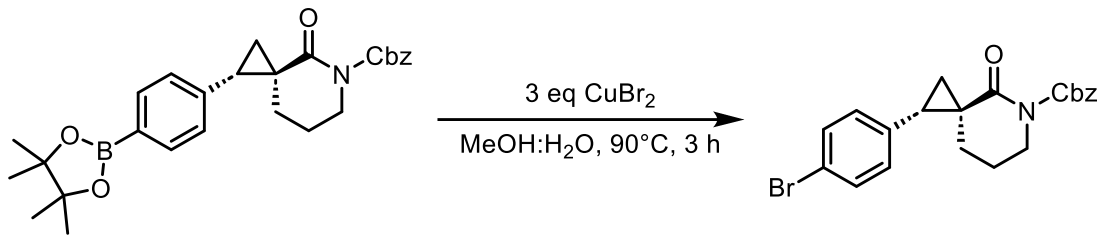

Conversion of **24b** to **16b** for enantioselectivity determination via chiral HPLC. The procedure was adapted from a literature procedure.[21]

To a solution of **24b** (1.0 eq, 80.2 μmol, 37.0 mg) in MeOH (0.1 M, 802 μL, reagent grade) in a flame-dried 8 mL vial, was added a previously prepared solution of copper(II) bromide (3.0 eq, 241 μmol, 53.7 mg) in water (0.3 M, 802 μL). The resulting solution was heated to 90°C for 3 h. The solution was then diluted with 3 mL of brine and extracted with 3 mL of EtOAc three times. The combined organic phases were washed with 3 mL brine, dried over $MgSO_4$, filtered, and concentrated *in vacuo*.

The crude residue was dissolved in 1 mL of CH_2Cl_2. Half of this was taken out and purified via prepTLC, eluting 25% EtOAc in Hex. The band corresponding to product was scraped off, washed with EtOAc, and concentrated *in vacuo*. 2.6 mg of **16b** was recovered and subjected to Chiral HPLC analysis.

Chiral HPLC: Sample dissolved in 80:20 Hex:IPA
AS-H column, 1.0 mL/min, 7.5% IPA in Hex, 30 min – 97% *ee*
Retention times: major 17.15 min, minor 20.08 min.

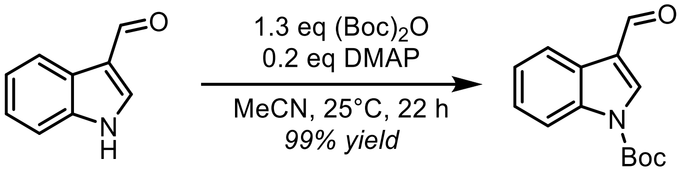

***tert*-Butyl 3-formyl-1H-indole-1-carboxylate (SI-6)**

To a flame-dried 100 mL RBF equipped with a stir bar was added 3-Indolylformaldehyde (1.0 eq, 10.0 mmol, 1.4516 g), DMAP (0.2 eq, 2.0 mmol, 244.34 mg), and $(Boc)_2O$ (1.3 eq, 13.0 mmol, 2.8373 g, 2.90 mL). This was degassed and backfilled with argon three times, left under an argon-filled balloon, then diluted with MeCN (0.25 M, 40.0 mL, SPS grade). The resulting solution was stirred at 25°C for 22 h. The reactions were then concentrated *in vacuo*, directly dry-loaded onto silica gel, then purified via automated flash column chromatography, eluting on a gradient. Fractions containing product were combined and concentrated *in vacuo* to afford 2434.4 mg of a white solid in 99% yield.[22]

Purification gradient:
0% EtOAc in Hex [5 CV] → 0 to 20% EtOAc in Hex [20 CV] → 20% EtOAc in Hex [5 CV]

^{1}H NMR (400 MHz, $CDCl_3$) δ 10.11 (s, 1H), 8.32 – 8.27 (m, 1H), 8.24 (s, 1H), 8.15 (d, *J* = 8.2 Hz, 1H), 7.47 – 7.33 (m, 2H), 1.71 (s, 9H).

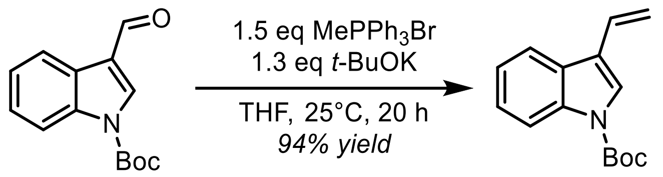

***tert*-Butyl 3-vinyl-1H-indole-1-carboxylate (SI-7)**

To a flame-dried 100 mL RBF equipped with a stir bar was added methyltriphenylphosphonium bromide (1.5 eq, 14.89 mmol, 5.3183 g) and *t*-BuOK (1.3 eq, 12.9 mmol, 1.4478 g). This was degassed and backfilled with argon three times, then diluted with THF (0.25 M to aldehyde, 40.0 mL, SPS grade). The resulting solution was stirred at 25°C for 1 h. To this, a previously degassed solution of **SI-6** (1 eq, 9.93 mmol, 2434.4 mg) in THF (1.0 M to aldehyde, 10.0 mL, SPS grade) dropwise. The resulting solution was stirred at 25°C for 20 h. The reaction was then concentrated *in vacuo* and directly dry-loaded onto silica gel, then purified via automated flash column chromatography, eluting on a gradient. Fractions containing product were combined and concentrated *in vacuo* to afford 2260.6 mg of a yellow oil in 94% yield.[23]

Purification gradient:
0% Et_2O in Hex [5 CV] → 0 to 5% Et_2O in Hex [20 CV] → 5% Et_2O in Hex [5 CV]

^{1}H NMR (400 MHz, $CDCl_3$) δ 8.19 (d, J = 8.2 Hz, 1H), 7.86 – 7.79 (m, 1H), 7.65 (s, 1H), 7.37 (ddd, J = 8.4, 7.2, 1.4 Hz, 1H), 7.31 (dd, J = 7.7, 1.2 Hz, 1H), 6.84 (ddd, J = 17.8, 11.3, 0.8 Hz, 1H), 5.84 (dd, J = 17.9, 1.3 Hz, 1H), 5.35 (dd, J = 11.3, 1.3 Hz, 1H), 1.70 (s, 9H).

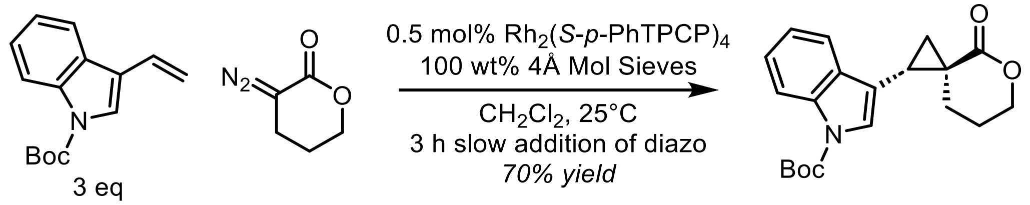

***tert*-Butyl 3-((1*S*,3*R*)-4-oxo-5-oxaspiro[2.5]octan-1-yl)-1H-indole-1-carboxylate (25a)**
Synthesized following **GP-1**. Absolute stereochemistry assigned by analogy to **24a**. A racemic sample was made following the same procedure but using 1 mol% $Rh_2(OAc)_4$.

SI-7: 3.0 eq, 0.6 mmol, 146 mg
Reaction time: 1 h

The crude residue was dry-loaded onto silica gel and purified via automated flash column chromatography, eluting on a gradient. Fractions containing product were combined and concentrated *in vacuo*, to obtain 47.9 mg of a clear oil in 70% yield. *E* and *Z* diastereomers collected together.

Purification gradient:
0% EtOAc in Hex [5 CV] → 0 to 20% EtOAc in Hex [20 CV] → 20% EtOAc in Hex [5 CV]

^{1}H NMR (400 MHz, $CDCl_3$) δ 8.11 (d, *J* = 8.0 Hz, 1H), 7.59 (ddd, *J* = 7.8, 1.3, 0.7 Hz, 1H), 7.35 (ddd, *J* = 8.4, 7.2, 1.3 Hz, 1H), 7.33 – 7.25 (m, 2H), 4.44 – 4.37 (m, 2H), 2.87 (ddd, *J* = 8.9, 7.2, 1.3 Hz, 1H), 2.02 (dd, *J* = 9.1, 4.3 Hz, 1H), 1.81 – 1.69 (m, 2H), 1.68 (s, 9H), 1.62 (dt, *J* = 8.9, 4.4 Hz, 1H), 1.46 – 1.38 (m, 1H), 1.18 (dd, *J* = 7.2, 4.4 Hz, 1H).

^{13}C NMR (101 MHz, $CDCl_3$) δ 174.5, 149.7, 135.7, 131.1, 125.0, 124.2, 123.0, 119.2, 117.1, 115.5, 84.1, 70.4, 28.3, 25.7, 25.4, 24.7, 23.6, 21.5.

HRMS (+p APCI) calculated for $C_{20}H_{24}NO_4$ (M+H) 342.1707, found 342.1703

Chiral HPLC: Sample dissolved in 80:20 Hex:IPA
OD-H column, 1.0 mL/min, 10.0% IPA in Hex, 15 min – 93% *ee*
Retention times: major 8.27 min, minor 9.95 min.

$[\alpha]^{21.5}_D$ = -27.0° (c = 0.52, $CHCl_3$, 93% *ee of E-diastereomer*)

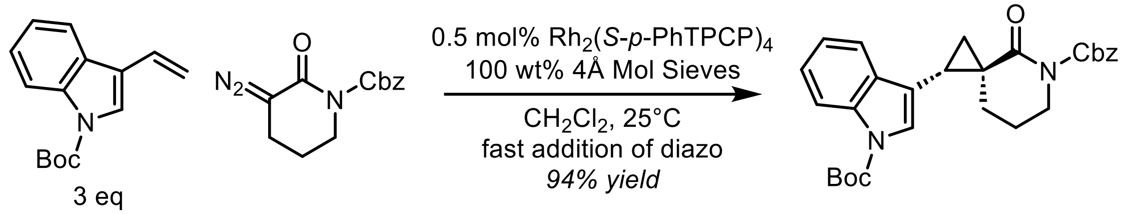

***tert*-Butyl 3-((1*S*,3*R*)-5-((benzyloxy)carbonyl)-4-oxo-5-azaspiro[2.5]octan-1-yl)-1H-indole-1-carboxylate (25b)**

Synthesized following **GP-2**. Absolute stereochemistry assigned by analogy to **24a**. A racemic sample was made following the same procedure but using 1 mol% $Rh_2(OAc)_4$.

SI-7: 3.0 eq, 0.6 mmol, 146 mg
Reaction time: 4 h

The crude residue was dry-loaded onto silica gel and purified via automated flash column chromatography, eluting on a gradient. Fractions containing product were combined and concentrated *in vacuo*, to obtain 88.8 mg of a clear oil in 94% yield. *E* and *Z* diastereomers collected together.

Purification gradient:
20% Et_2O in Hex [5 CV] → 20 to 70% Et_2O in Hex [20 CV] → 70% Et_2O in Hex [5 CV]

^{1}H NMR (400 MHz, $CDCl_3$) δ 8.10 (s, 1H), 7.56 (dt, *J* = 7.7, 1.0 Hz, 1H), 7.47 (dt, *J* = 6.1, 1.5 Hz, 2H), 7.39 (tt, *J* = 8.0, 1.9 Hz, 2H), 7.36 – 7.33 (m, 2H), 7.33 – 7.28 (m, 1H), 7.24 (dd, *J* = 7.5, 1.1 Hz, 1H), 5.32 (s, 2H), 3.88 – 3.68 (m, 2H), 2.87 (ddd, *J* = 8.9, 7.2, 1.3 Hz, 1H), 2.00 (dd, *J* = 9.1, 4.1 Hz, 1H), 1.78 – 1.70 (m, 1H), 1.67 (s, 9H), 1.62 – 1.55 (m, 2H), 1.45 – 1.36 (m, 1H), 1.14 (dd, *J* = 7.1, 4.2 Hz, 1H).

^{13}C NMR (101 MHz, $CDCl_3$) δ 173.5, 154.3, 149.8, 135.7, 131.1, 128.7, 128.4, 128.3, 124.9, 124.1, 122.9, 119.4, 117.7, 115.4, 84.0, 68.6, 47.8, 29.6, 28.4, 25.4, 25.4, 22.6, 21.4.
One missing signal overlaps with another.

HRMS (+p APCI) calculated for $C_{28}H_{31}N_2O_5$ (M+H) 475.2235, found 475.2235

Chiral HPLC: Sample dissolved in 80:20 Hex:IPA
OD-H column, 1.0 mL/min, 7.5% IPA in Hex, 30 min – 94% *ee*
Retention times: major 11.41 min, minor 13.45 min.

$[\alpha]^{21.6}_D$ = -91.5° (c = 1.05, $CHCl_3$, 94% *ee of E-diastereomer*)

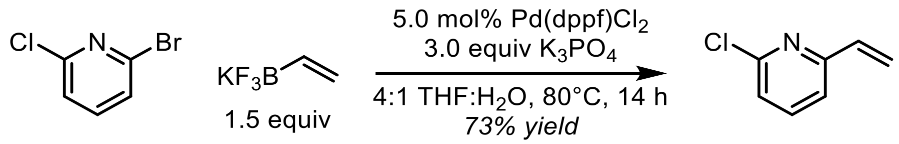

2-Chloro-6-vinylpyridine (SI-8)

To a flame-dried 250 mL RBF equipped with a stir bar was added potassium trifluoro(vinyl)borate (1.5 eq, 30.0 mmol, 4.0185 g), 2-chloro-6-bromopyridine (1.0 eq, 20.0 mmol, 3.8488 g), K_3PO_4 (3.0 eq, 60.0 mmol, 12.736 g), Pd(dppf)Cl_2 (5.0 mol%, 1.0 mmol, 731.71 mg). This was then degassed and backfilled with argon three times and left under an argon atmosphere via balloon. The flask was then diluted with THF (0.25 M to pyridine, 80.0 mL) and water (1 M to pyridine, 20.0 mL) and heated to 80°C via open-top findenser for 14 h. The reaction was then cooled to 25°C and poured into a 250 mL separatory funnel, which was then diluted with 50 mL of brine and extracted with 50 mL of EtOAc three times. The combined organic phases were washed with 50 mL of brine, dried over $MgSO_4$, filtered, and concentrated *in vacuo*. The reaction was then dry-loaded onto silica gel and purified via automated flash column chromatography, eluting on a gradient. Fractions containing product were combined and concentrated *in vacuo* to afford 2269.4 mg of a yellow oil in 73% yield and 90% purity. ^{1}H NMR matched the reported spectra.[24]

Purification gradient:
0% Et_2O in Hex [5 CV] → 0 to 10% Et_2O in Hex [20 CV] → 10% Et_2O in Hex [5 CV]

^{1}H NMR (400 MHz, $CDCl_3$) δ 7.60 (t, *J* = 7.8 Hz, 1H), 7.23 (dd, *J* = 7.6, 0.8 Hz, 1H), 7.18 (dd, *J* = 7.9, 0.8 Hz, 1H), 6.74 (dd, *J* = 17.4, 10.8 Hz, 1H), 6.25 (dd, *J* = 17.4, 1.1 Hz, 1H), 5.52 (dd, *J* = 10.8, 1.2 Hz, 1H).

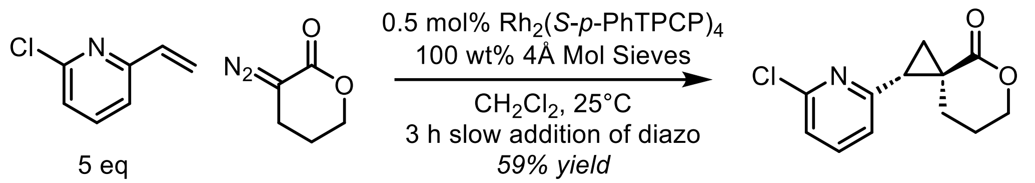

(1*R*,3*R*)-1-(6-chloropyridin-2-yl)-5-oxaspiro[2.5]octan-4-one (26a)

Synthesized following **GP-1**. Absolute stereochemistry assigned by analogy to **24a**. A racemic sample was made following the same procedure but using 1 mol% $Rh_2(OAc)_4$.

SI-8: 5.0 eq, 1.0 mmol, 140 mg
Reaction time: 30 min

The crude residue was dry-loaded onto silica gel and purified via automated flash column chromatography, eluting on a gradient. Fractions containing product were combined and concentrated *in vacuo*, to obtain 27.8 mg of a clear oil in 59% yield. *E* and *Z* diastereomers collected together.

Purification gradient:
20% Et_2O in Hex [5 CV] → 20 to 70% Et_2O in Hex [20 CV] → 70% Et_2O in Hex [5 CV]

^{1}H NMR (400 MHz, $CDCl_3$) δ 7.58 (t, *J* = 7.7 Hz, 1H), 7.26 (dd, *J* = 7.7, 0.8 Hz, 1H), 7.18 (dd, *J* = 7.9, 0.8 Hz, 1H), 4.45 (dddd, *J* = 10.8, 6.6, 4.0, 1.3 Hz, 1H), 4.41 – 4.33 (m, 1H), 3.01 (dd, *J* = 9.0, 6.9 Hz, 1H), 1.92 – 1.83 (m, 3H), 1.77 (dt, *J* = 6.9, 4.3 Hz, 2H), 1.63 – 1.54 (m, 1H).

^{13}C NMR (101 MHz, $CDCl_3$) δ 173.5, 157.5, 150.8, 138.8, 123.6, 122.3, 70.5, 33.9, 28.4, 23.9, 23.3, 22.5.

HRMS (+p APCI) calculated for $C_{12}H_{13}ClNO_2$ (M+H) 238.0637, found 238.0626

Chiral HPLC: Sample dissolved in 80:20 Hex:IPA
OD-H column, 1.0 mL/min, 10.0% IPA in Hex, 30 min – 83% *ee*
Retention times: major 12.29 min, minor 17.17 min.

$[\alpha]^{21.8}_D$ = -249.0° (c = 1.32, $CHCl_3$, 83% *ee of E-diastereomer*)

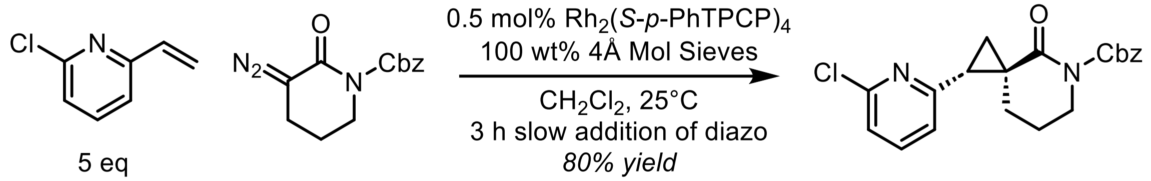

Benzyl (1*R*,3*R*)-1-(6-chloropyridin-2-yl)-4-oxo-5-azaspiro[2.5]octane-5-carboxylate (26b)
Synthesized following **GP-2**. Absolute stereochemistry assigned by analogy to **24a**. A racemic sample was made following the same procedure but using 1 mol% $Rh_2(OAc)_4$.

SI-8: 5.0 eq, 1.0 mmol, 140 mg
Reaction time: 8 h

The crude residue was dry-loaded onto silica gel and purified via automated flash column chromatography, eluting on a gradient. Fractions containing product were combined and concentrated *in vacuo*, to obtain 59.3 mg of a clear oil in 80% yield. *E* and *Z* diastereomers collected together.

Purification gradient:
20% Et_2O in Hex [5 CV] → 20 to 70% Et_2O in Hex [20 CV] → 70% Et_2O in Hex [5 CV]

^{1}H NMR (400 MHz, $CDCl_3$) δ 7.55 (t, *J* = 7.7 Hz, 1H), 7.47 – 7.41 (m, 2H), 7.40 – 7.35 (m, 2H), 7.35 – 7.29 (m, 1H), 7.23 (dd, *J* = 7.6, 0.8 Hz, 1H), 7.16 (dd, *J* = 7.9, 0.8 Hz, 1H), 5.29 (s, 2H), 3.89 – 3.79 (m, 1H), 3.77 – 3.68 (m, 1H), 3.00 (dd, *J* = 9.0, 6.9 Hz, 1H), 1.88 (dd, *J* = 9.0, 3.9 Hz, 1H), 1.85 – 1.76 (m, 2H), 1.75 – 1.65 (m, 2H), 1.58 – 1.49 (m, 1H).

^{13}C NMR (101 MHz, $CDCl_3$) δ 172.7, 158.1, 154.2, 150.8, 138.7, 135.6, 128.7, 128.4, 128.3, 123.5, 122.1, 68.7, 47.8, 33.9, 32.4, 24.3, 22.23, 21.99.

HRMS (+p APCI) calculated for $C_{20}H_{20}ClN_3O_3$ (M+H) 371.1164, found 371.1148

Chiral HPLC: Sample dissolved in 80:20 Hex:IPA
OD-H column, 1.0 mL/min, 10.0% IPA in Hex, 30 min – 92% *ee*
Retention times: major 14.91 min, minor 19.33 min.

$[\alpha]^{22.1}_D$ = -286.3° (c = 0.92, $CHCl_3$, 92% *ee of E-diastereomer*)

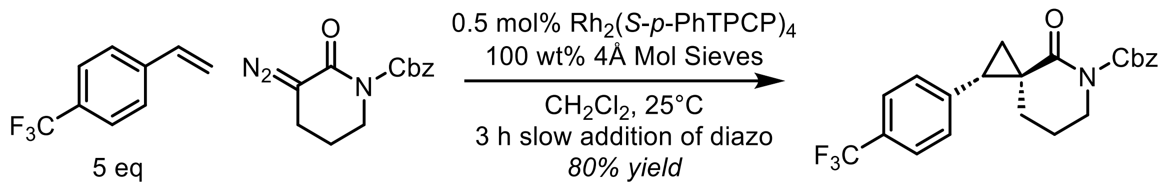

Benzyl (1*S*,3*R*)-4-oxo-1-(4-(trifluoromethyl)phenyl)-5-azaspiro[2.5]octane-5-carboxylate (27b)

Synthesized following **GP-2**. Absolute stereochemistry assigned by analogy to **24a**. A racemic sample was made following the same procedure but using 1 mol% $Rh_2(OAc)_4$.

4-(Trifluoromethyl)styrene: 5.0 eq, 1.0 mmol, 172 mg, 148 µL
Reaction time: 17 h

The crude residue was dry-loaded onto silica gel and purified via automated flash column chromatography, eluting on a gradient. Fractions containing product were combined and concentrated *in vacuo*, to obtain 64.6 mg of a clear oil in 80% yield. *E* and *Z* diastereomers collected together.

Purification gradient:
15% Et_2O in Hex [5 CV] → 15 to 65% Et_2O in Hex [20 CV] → 65% Et_2O in Hex [5 CV]

^{1}H NMR (400 MHz, $CDCl_3$) δ 7.57 (d, *J* = 8.1 Hz, 2H), 7.48 – 7.42 (m, 2H), 7.41 – 7.36 (m, 2H), 7.36 – 7.32 (m, 1H), 7.30 (d, *J* = 8.1 Hz, 2H), 5.30 (s, 2H), 3.87 – 3.73 (m, 2H), 3.04 (dd, *J* = 9.1, 7.3 Hz, 1H), 1.94 (dd, *J* = 9.1, 4.5 Hz, 1H), 1.80 (ddddd, *J* = 13.4, 8.5, 7.0, 5.1, 3.5 Hz, 1H), 1.68 (ddtd, *J* = 13.7, 7.2, 5.2, 3.7 Hz, 1H), 1.51 – 1.42 (m, 1H), 1.31 (ddd, *J* = 14.1, 8.3, 3.7 Hz, 1H), 1.26 (dd, *J* = 7.2, 4.6 Hz, 1H).

^{19}F NMR (376 MHz, $CDCl_3$) δ -62.43 (s, 1F).

^{13}C NMR (101 MHz, $CDCl_3$) δ 172.94, 154.11, 140.85, 135.55, 129.56, 129.29 (d, *J* = 32.5 Hz), 128.75, 128.50, 128.34, 125.39 (q, *J* = 3.7 Hz), 124.26 (d, *J* = 271.9 Hz), 68.72, 47.74, 33.43, 30.38, 25.65, 22.09, 21.40.
CF_3 multiplicity only observed as doublets (not quartets) for $^1J_{C\text{-}F}$ and $^2J_{C\text{-}F}$ coupling

HRMS (+p APCI) calculated for $C_{22}H_{21}F_3NO_3$ (M+H) 404.1475, found 404.1468

Chiral HPLC: Sample dissolved in 80:20 Hex:IPA
AS-H column, 1.0 mL/min, 10.0% IPA in Hex, 15 min – 96% *ee*
Retention times: major 11.32 min, minor 9.87 min.

$[\alpha]^{21.5}_D$ = -136.3° (c = 0.66, $CHCl_3$, 96% *ee of E-diastereomer*)

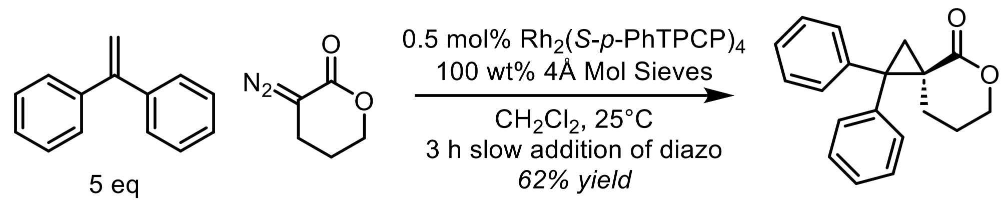

(*R*)-1,1-diphenyl-5-oxaspiro[2.5]octan-4-one (28a)
Synthesized following **GP-1**. Absolute stereochemistry assigned by analogy to **24a**. A racemic sample was made following the same procedure but using 1 mol% $Rh_2(OAc)_4$.

1,1-Diphenylethylene: 5.0 eq, 1.0 mmol, 180 mg, 176 µL
Reaction time: 2 h

The crude residue was dry-loaded onto silica gel and purified via automated flash column chromatography, eluting on a gradient. Fractions containing product were combined and concentrated *in vacuo*, to obtain 34.4 mg of a clear oil in 62% yield.

Purification gradient:
10% Et_2O in Hex [5 CV] → 10 to 50% Et_2O in Hex [20 CV] → 50% Et_2O in Hex [5 CV]

^{1}H NMR (400 MHz, $CDCl_3$) δ 7.45 – 7.40 (m, 2H), 7.37 – 7.28 (m, 4H), 7.26 (dd, *J* = 9.2, 6.2 Hz, 3H), 7.23 – 7.18 (m, 1H), 7.18 – 7.12 (m, 1H), 4.70 – 4.60 (m, 1H), 4.52 – 4.41 (m, 1H), 2.42 (d, *J* = 4.9 Hz, 1H), 2.14 – 2.01 (m, 1H), 1.94 – 1.77 (m, 2H), 1.67 (d, *J* = 4.9 Hz, 1H), 1.50 – 1.40 (m, 1H).

^{13}C NMR (101 MHz, $CDCl_3$) δ 171.7, 142.0, 140.5, 129.4, 129.2, 128.9, 128.7, 127.2, 126.9, 69.8, 48.1, 31.8, 26.1, 24.9, 23.0.

HRMS (+p APCI) calculated for $C_{19}H_{19}O_2$ (M+H) 279.1387, found 279.1373

Chiral HPLC: Sample dissolved in 80:20 Hex:IPA
AS-H column, 1.0 mL/min, 10.0% IPA in Hex, 45 min – 90% *ee*
Retention times: major 29.43 min, minor 21.46 min.

$[\alpha]^{22.2}_D$ = -131.2° (c = 1.16, $CHCl_3$, 90% *ee of E-diastereomer*)

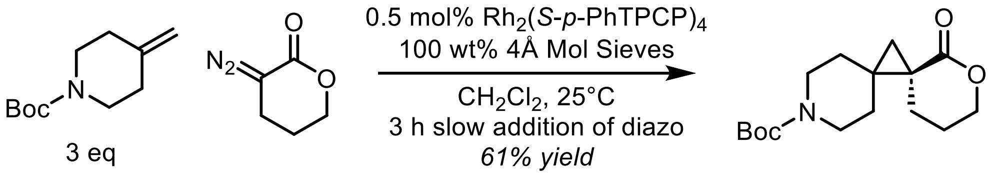

***tert*-Butyl (*R*)-1-oxo-2-oxa-10-azadispiro[5.0.5^7.1^6]tridecane-10-carboxylate (29a)**

Synthesized following **GP-1**. Absolute stereochemistry assigned by analogy to **24a**. A racemic sample was made following the same procedure but using 1 mol% $Rh_2(OAc)_4$. *tert*-Butyl 4-methylene-1-piperidinecarboxylate was prepared following the literature procedure.[25]

tert-Butyl 4-methylene-1-piperidinecarboxylate: 3.0 eq, 0.6 mmol, 118 mg
Reaction time: 2 h

The crude residue was dry-loaded onto silica gel and purified via automated flash column chromatography, eluting on a gradient. Fractions containing product were combined and concentrated *in vacuo*, to obtain 36.0 mg of a clear oil in 61% yield.

Purification gradient:
50% Et_2O in Hex [5 CV] → 50 to 100% Et_2O in Hex [20 CV] → 100% Et_2O in Hex [5 CV]

^{1}H NMR (400 MHz, $CDCl_3$) δ 4.43 – 4.33 (m, 1H), 4.29 – 4.17 (m, 1H), 3.88 (d, *J* = 58.3 Hz, 2H), 3.01 (dh, *J* = 13.2, 3.6 Hz, 2H), 2.05 – 1.80 (m, 4H), 1.73 (ddd, *J* = 14.2, 10.6, 4.2 Hz, 1H), 1.63 – 1.56 (m, 3H), 1.45 (s, 9H), 1.36 (dt, *J* = 13.3, 3.9 Hz, 1H), 0.59 (d, *J* = 4.7 Hz, 1H).

^{13}C NMR (101 MHz, $CDCl_3$) δ 173.0, 154.9, 79.7, 68.8, 32.6, 31.4, 29.8, 29.4, 28.6, 25.1, 24.5, 23.3.

HRMS (+p APCI) calculated for $C_{16}H_{26}NO_4$ (M+H) 296.1864, found 296.1849

Chiral HPLC: Sample dissolved in 80:20 Hex:IPA
AS-H column, 1.0 mL/min, 10.0% IPA in Hex, 30 min – 77% *ee*
Retention times: major 16.95 min, minor 12.17 min.

$[\alpha]^{22.2}_D$ = -3.6° (c = 1.60, $CHCl_3$, 77% *ee of E-diastereomer*)

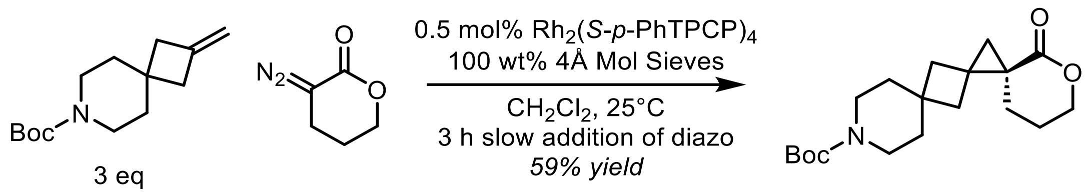

***tert*-Butyl (*R*)-1-oxo-2-oxa-12-azatrispiro[5.0.1.5^{9}.1^{7}.1^{6}]hexadecane-12-carboxylate (30a)**

Synthesized following **GP-1**. Absolute stereochemistry assigned by analogy to **24a**. A racemic sample was made following the same procedure but using 1 mol% $Rh_2(OAc)_4$. *tert*-Butyl 2-methylene-7-azaspiro[3.5]nonane-7-carboxylate was prepared following the literature procedure.[25]

tert-Butyl 2-methylene-7-azaspiro[3.5]nonane-7-carboxylate: 3.0 eq, 0.6 mmol, 142 mg
Reaction time: 30 min

The crude residue was dry-loaded onto silica gel and purified via automated flash column chromatography, eluting on a gradient. Fractions containing product were combined and concentrated *in vacuo*, to obtain 39.9 mg of a clear oil in 59% yield.

Purification gradient:
50% Et_2O in Hex [5 CV] → 50 to 100% Et_2O in Hex [20 CV] → 100% Et_2O in Hex [5 CV]

^{1}H NMR (400 MHz, $CDCl_3$) δ 4.46 – 4.34 (m, 2H), 3.51 – 3.36 (m, 2H), 3.33 – 3.13 (m, 2H), 2.02 – 1.84 (m, 5H), 1.78 (ddd, *J* = 13.7, 8.5, 4.9 Hz, 1H), 1.71 (d, *J* = 11.7 Hz, 1H), 1.68 – 1.61 (m, 3H), 1.58 – 1.47 (m, 3H), 1.44 (s, 9H), 0.74 (d, *J* = 4.5 Hz, 1H).

^{13}C NMR (101 MHz, $CDCl_3$) δ 172.0, 155.0, 79.5, 70.3, 38.9, 38.3, 37.4, 32.4, 32.2, 29.0, 28.6, 26.8, 26.6, 23.5.

HRMS (+p APCI) calculated for $C_{19}H_{30}NO_4$ (M+H) 336.2177, found 336.2162

Chiral HPLC: Sample dissolved in 80:20 Hex:IPA
AS-H column, 1.0 mL/min, 10.0% IPA in Hex, 30 min – 77% *ee*
Retention times: major 14.11 min, minor 10.55 min.

$[\alpha]^{22.2}_{D}$ = -12.1° (c = 1.24, $CHCl_3$, 77% *ee of E-diastereomer*)

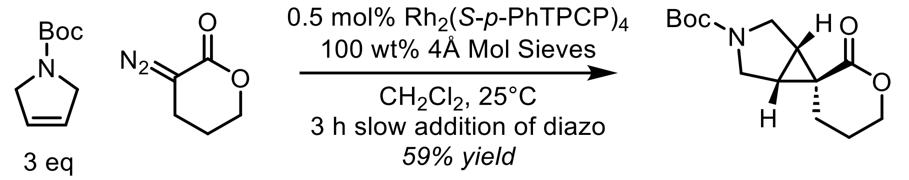

***tert*-Butyl (1*R*,5*S*,6*r*)-2'-oxodihydro-2'*H*,4'*H*-3-azaspiro[bicyclo[3.1.0]hexane-6,3'-pyran]-3-carboxylate (32a)**

Synthesized following **GP-1**. Absolute stereochemistry determined by 2D NMR experiments. 1-*tert*-Butyloxycarbonyl-3-pyrroline (**31**) was purchased from a commercial source and purified according to the procedure outlined in the literature.[26]

1-*tert*-butyloxycarbonyl-3-pyrroline: 3.0 eq, 0.6 mmol, 102 mg
Reaction time: 30 min

The crude residue was purified via flash column chromatography, eluting 50% EtOAc in Hex. Fractions containing product were combined and concentrated *in vacuo*, to obtain a 31.4 mg of a clear oil in 59% yield.

^{1}H NMR (400 MHz, $CDCl_3$) δ 4.41 (t, J = 5.5 Hz, 2H), 3.60 (ddd, J = 12.1, 10.1, 4.5 Hz, 2H), 3.43 (d, J = 12.2 Hz, 1H), 3.35 (d, J = 12.1 Hz, 1H), 2.37 – 2.26 (m, 2H), 2.03 – 1.93 (m, 2H), 1.65 – 1.61 (m, 1H), 1.59 (dd, J = 6.2, 2.1 Hz, 2H), 1.44 (s, 9H).

^{13}C NMR (101 MHz, $CDCl_3$) δ 172.8, 153.7, 80.0, 69.8, 45.6, 45.4, 31.9, 31.3, 28.6, 26.1, 23.3, 17.3.

HRMS (+p APCI) calculated for $C_{14}H_{22}NO_4$ (M+H) 268.1551, found 268.1539

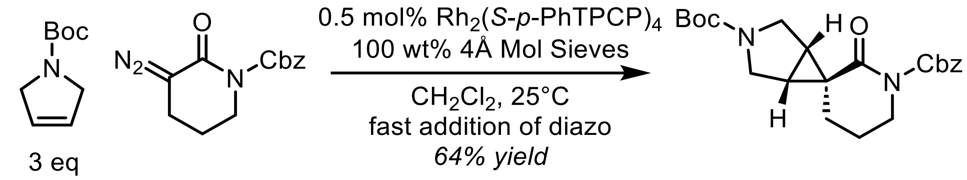

1'-Benzyl 3-(*tert*-butyl) (1*R*,5*S*,6*r*)-2'-oxo-3-azaspiro[bicyclo[3.1.0]hexane-6,3'-piperidine]-1',3-dicarboxylate (32b)

Synthesized following **GP-2**. Absolute stereochemistry assigned by analogy to **32a**. 1-*tert*-Butyloxycarbonyl-3-pyrroline (**31**) was purchased from a commercial source and purified according to the procedure outlined in the literature.[26]

1-*tert*-butyloxycarbonyl-3-pyrroline: 3.0 eq, 0.6 mmol, 102 mg
Reaction time: 6 h

The crude residue was dry-loaded onto silica gel and purified via automated flash column chromatography, eluting on a gradient. Fractions containing product were combined and concentrated *in vacuo*, to obtain 54.2 mg of a clear oil in 64% yield.

Purification gradient:
30% Et_2O in Hex [5 CV] → 30 to 70% Et_2O in Hex [20 CV] → 70% Et_2O in Hex [5 CV]

^{1}H NMR (400 MHz, $CDCl_3$) δ 7.43 – 7.39 (m, 2H), 7.36 (ddd, J = 8.0, 5.5, 3.6 Hz, 2H), 7.33 – 7.28 (m, 1H), 5.26 (s, 2H), 3.87 – 3.73 (m, 2H), 3.61 (td, J = 11.9, 4.9 Hz, 2H), 3.38 (d, J = 12.1 Hz, 1H), 3.29 (d, J = 12.0 Hz, 1H), 2.30 (qd, J = 8.1, 4.8 Hz, 2H), 1.92 (p, J = 6.1 Hz, 2H), 1.60 – 1.52 (m, 2H), 1.43 (s, 9H).

^{13}C NMR (101 MHz, $CDCl_3$) δ 172.1, 154.0, 153.8, 135.5, 128.7, 128.4, 128.3, 79.9, 68.6, 47.3, 45.9, 45.7, 31.9, 31.4, 30.3, 28.6, 22.4, 18.1.

HRMS (+p APCI) calculated for $C_{22}H_{29}N_2O_5$ (M+H) 401.2078, found 401.2063

Spectra and Chromatograms

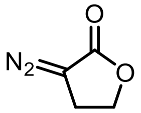

3-Diazodihydrofuran-2(3H)-one (6a)

^{1}H NMR (400 MHz, $CDCl_3$)

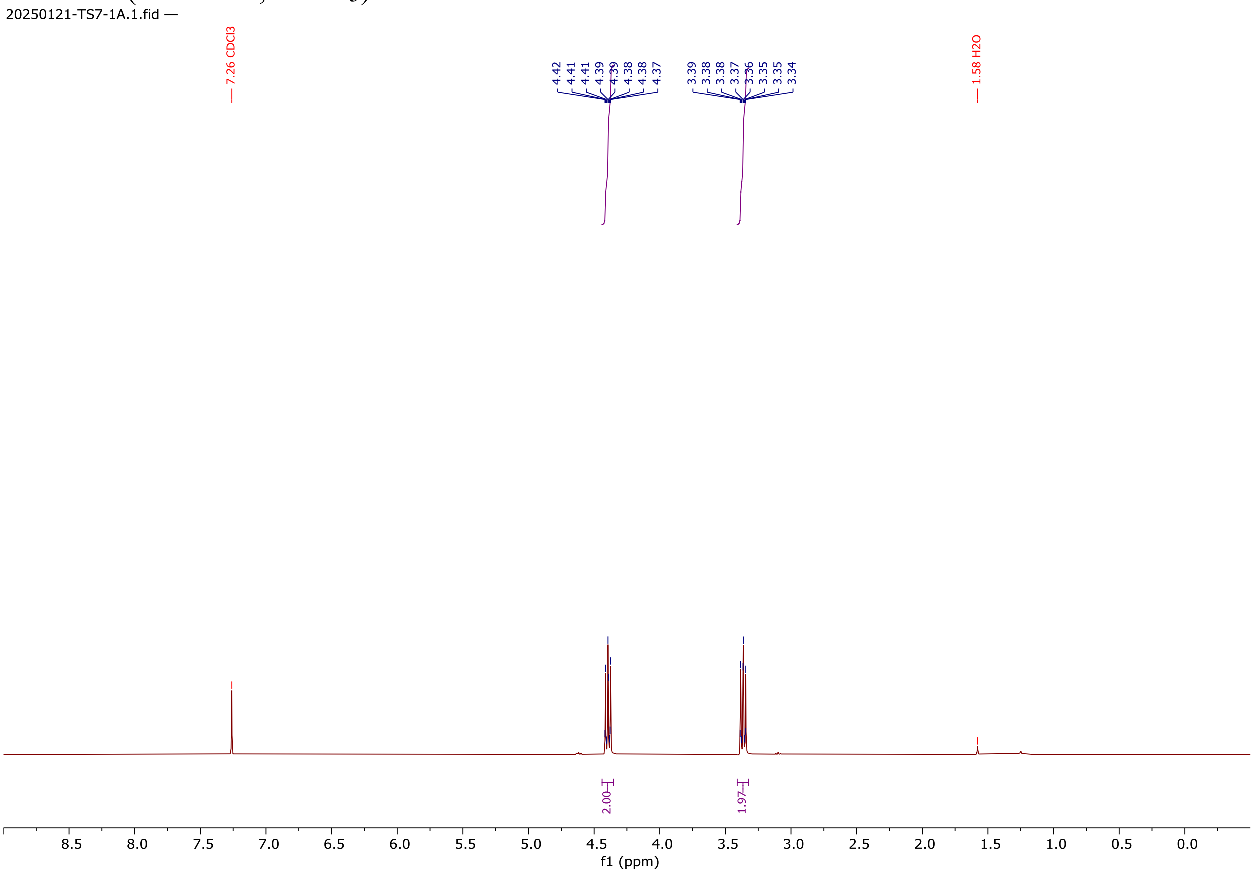

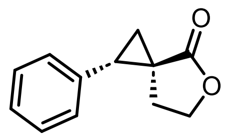

(1*S*,3*S*)-1-phenyl-5-oxaspiro[2.4]heptan-4-one (7a)

^{1}H NMR (400 MHz, $CDCl_3$)

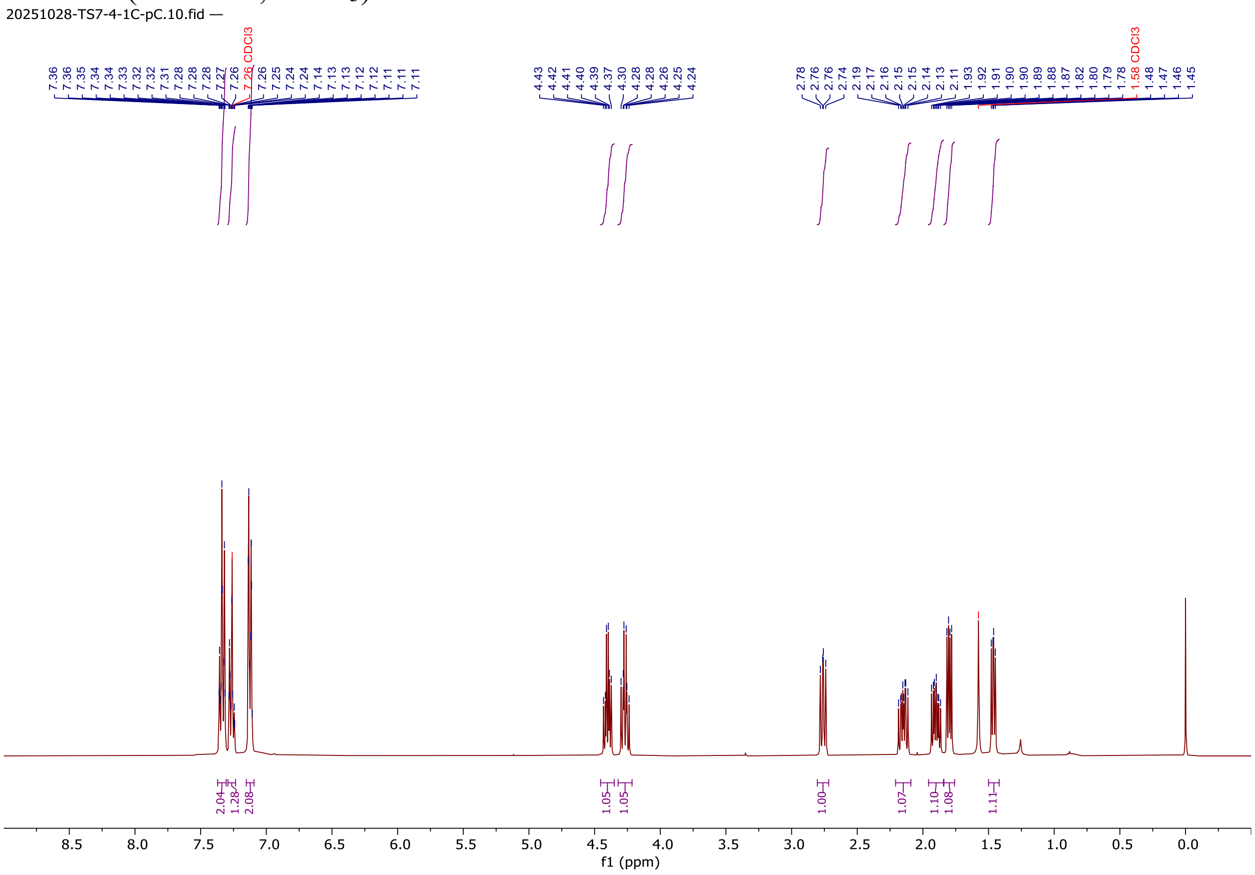

SFC (210 nm trace):
Racemate, synthesized from $Rh_2(OAc)_4$

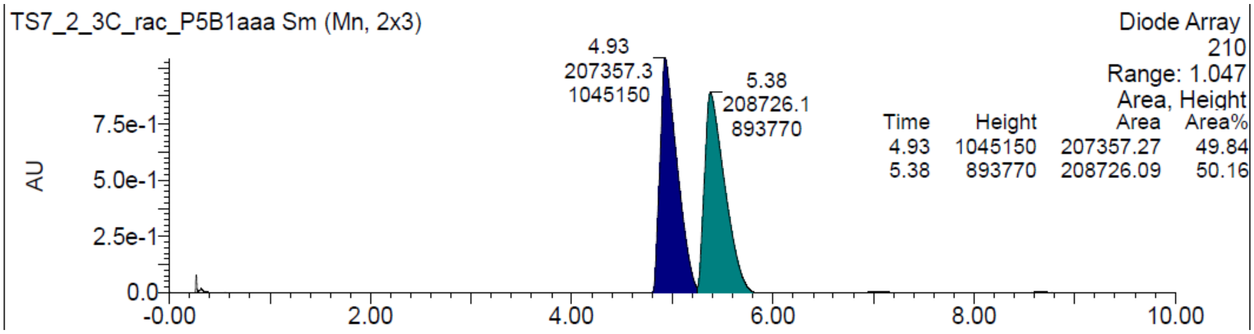

Chiral, synthesized from Rh_2(*S*-*p*-PhTPCP)$_4$, 43% *ee*

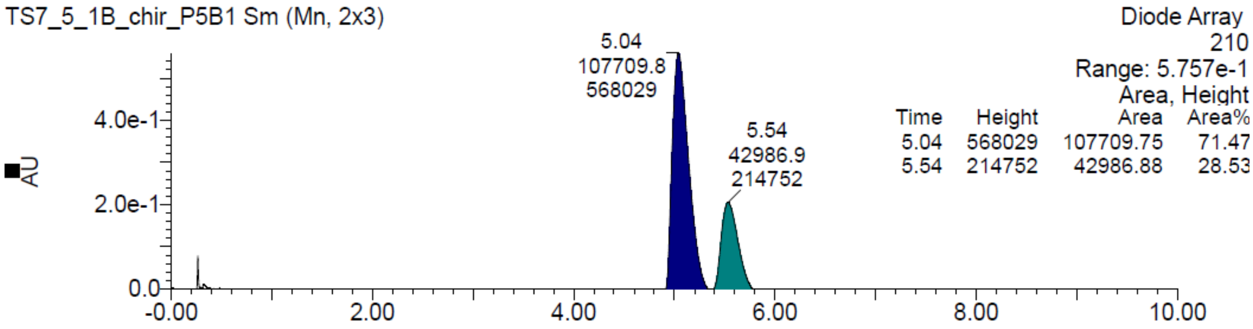

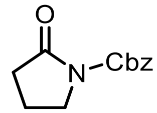

Benzyl 2-oxopyrrolidine-1-carboxylate (SI-1)

^{1}H NMR (400 MHz, $CDCl_3$)

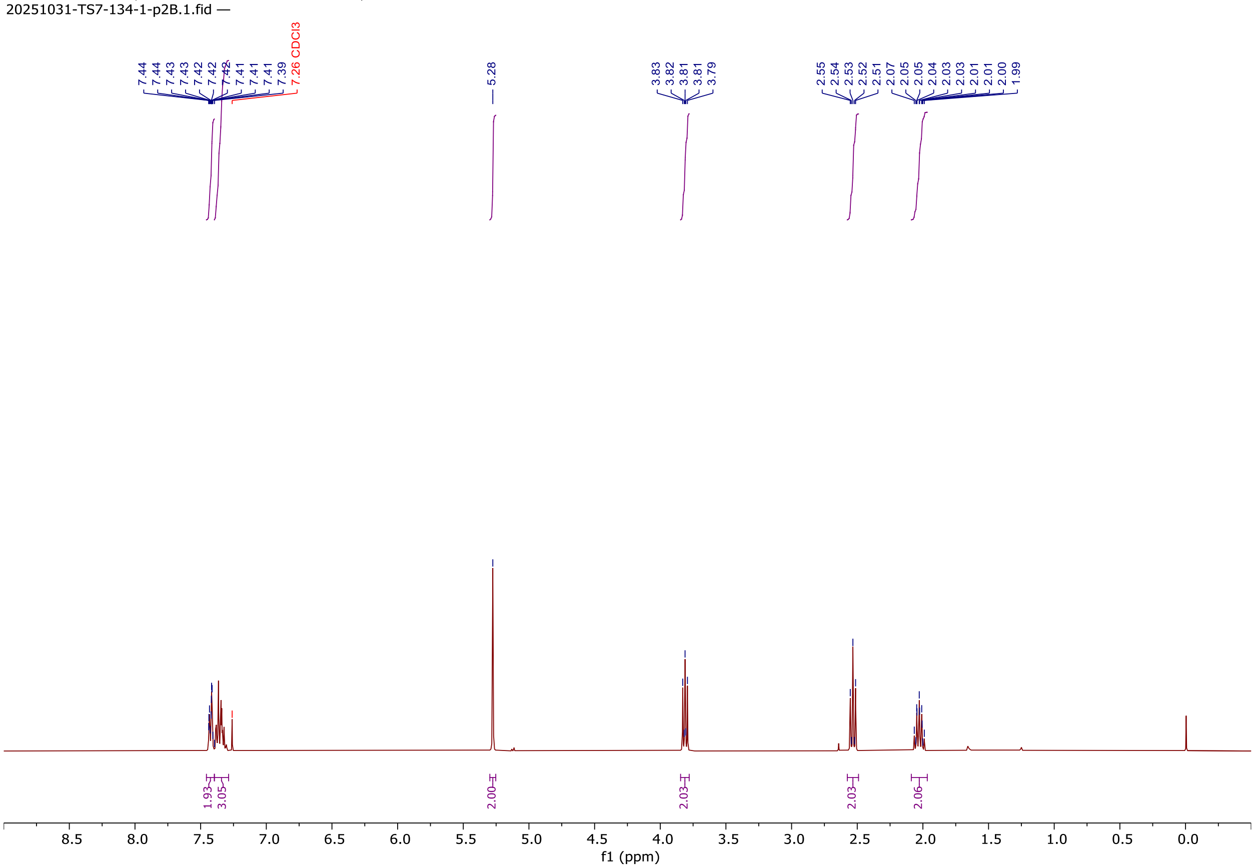

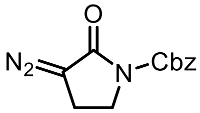

Benzyl 3-diazo-2-oxopyrrolidine-1-carboxylate (6b)

^{1}H NMR (400 MHz, $CDCl_3$)

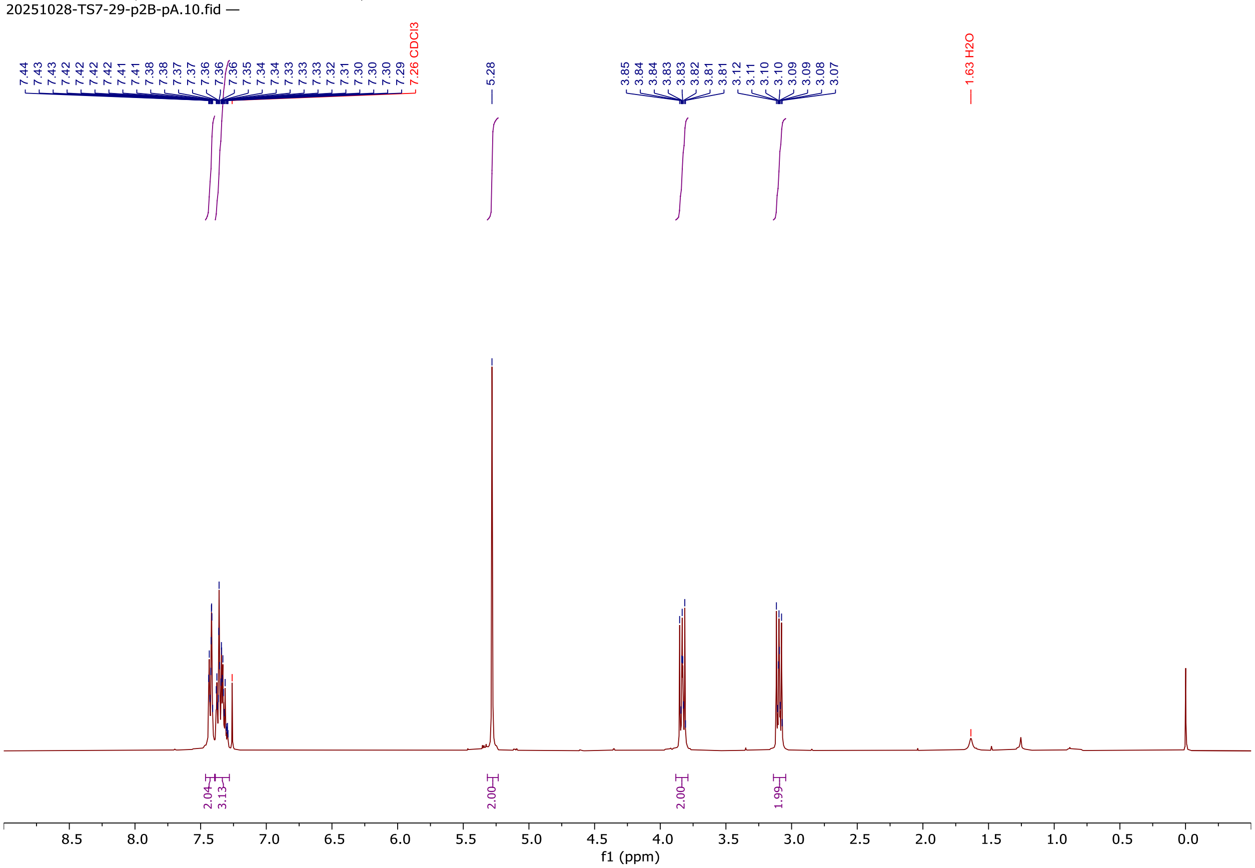

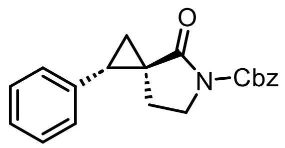

Benzyl (1*S*,3*S*)-4-oxo-1-phenyl-5-azaspiro[2.4]heptane-5-carboxylate (7b)

^{1}H NMR (400 MHz, $CDCl_3$)

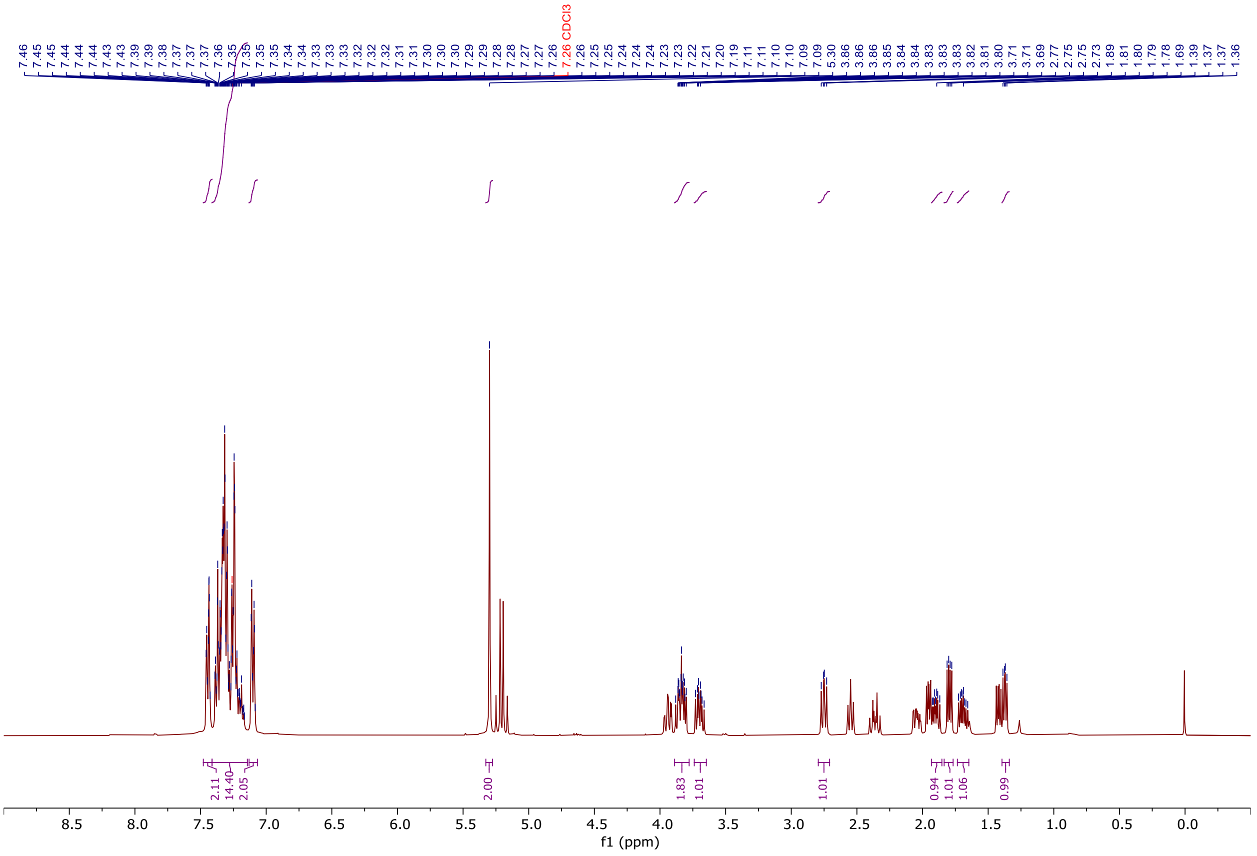

^{13}C NMR (101 MHz, $CDCl_3$)

20251110-TS7-31-1-p2AB.11.fid —

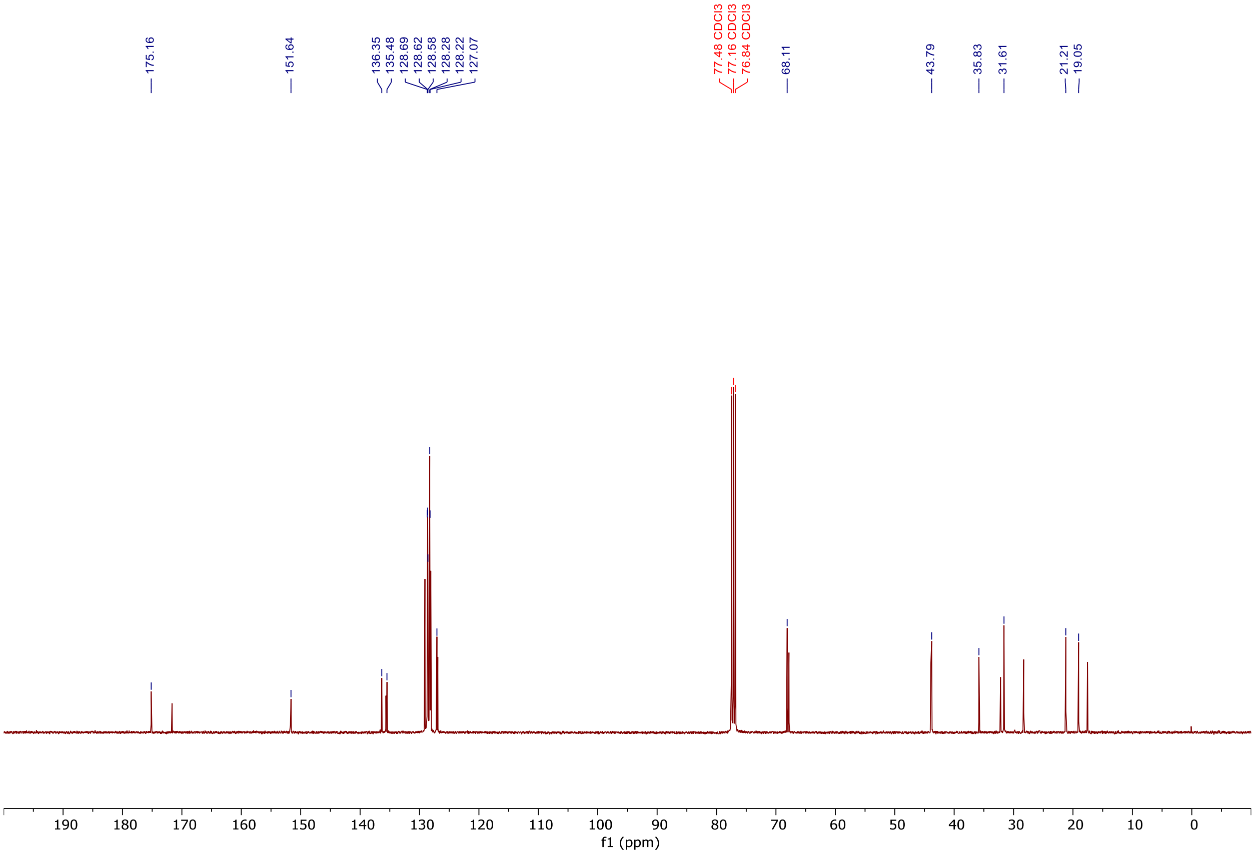

Chiral HPLC (210 nm trace):
Racemate, synthesized from $Rh_2(OAc)_4$

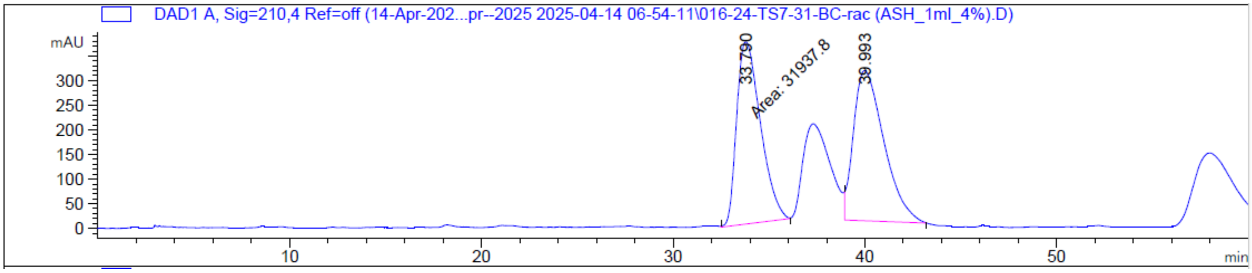


```
Signal 1: DAD1 A, Sig=210,4 Ref=off

Peak RetTime Type  Width     Area       Height      Area
  #   [min]        [min]   [mAU*s]      [mAU]        %
----|-------|----|-------|----------|----------|--------|
   1  33.790 MM    1.4392 3.19378e4  369.86551  48.6410
   2  39.993 FM R  1.8418 3.37224e4  305.15826  51.3590

Totals :                  6.56602e4  675.02377
```

Chiral, synthesized from Rh_2(*S*-*p*-PhTPCP)$_4$, 43% *ee*

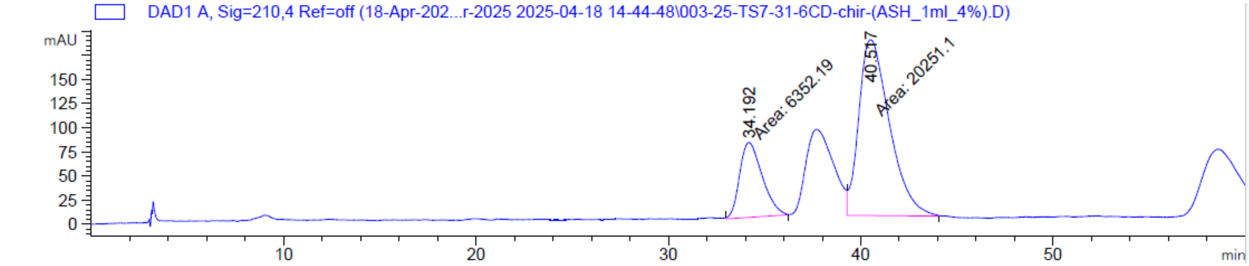


```
Signal 1: DAD1 A, Sig=210,4 Ref=off

Peak RetTime Type  Width     Area       Height      Area
  #   [min]        [min]   [mAU*s]      [mAU]        %
----|-------|----|-------|----------|----------|--------|
   1  34.192 MM    1.3650 6352.19189   77.56152  23.8774
   2  40.517 FM    1.8536 2.02511e4  182.08859  76.1226

Totals :                  2.66033e4  259.65012
```

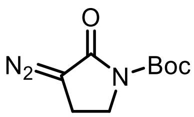

tert-Butyl 3-diazo-2-oxopyrrolidine-1-carboxylate (6c)

^{1}H NMR (400 MHz, $CDCl_3$)

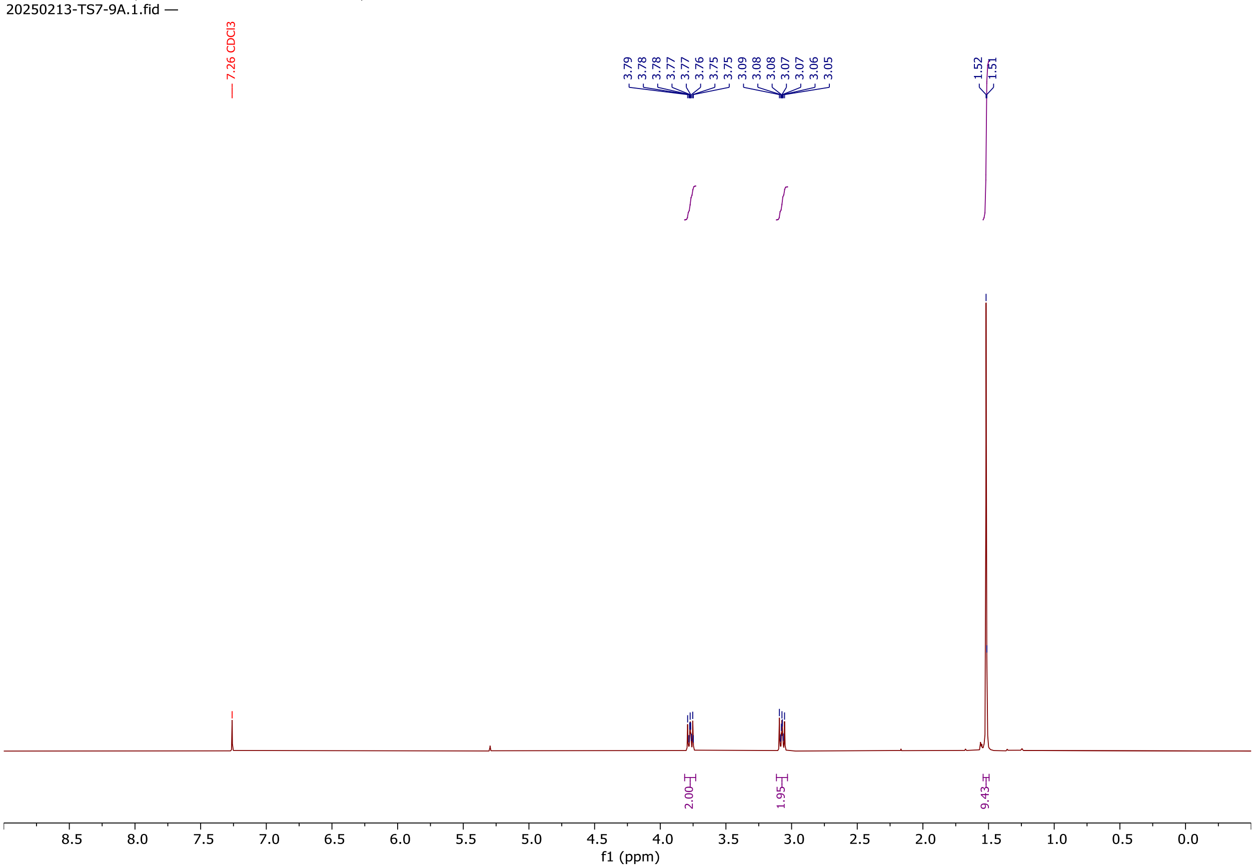

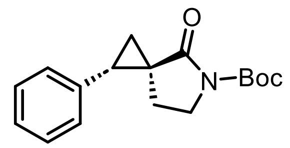

tert-Butyl (1_S_,3_S_)-4-oxo-1-phenyl-5-azaspiro[2.4]heptane-5-carboxylate (7c)

^{1}H NMR (400 MHz, $CDCl_3$)

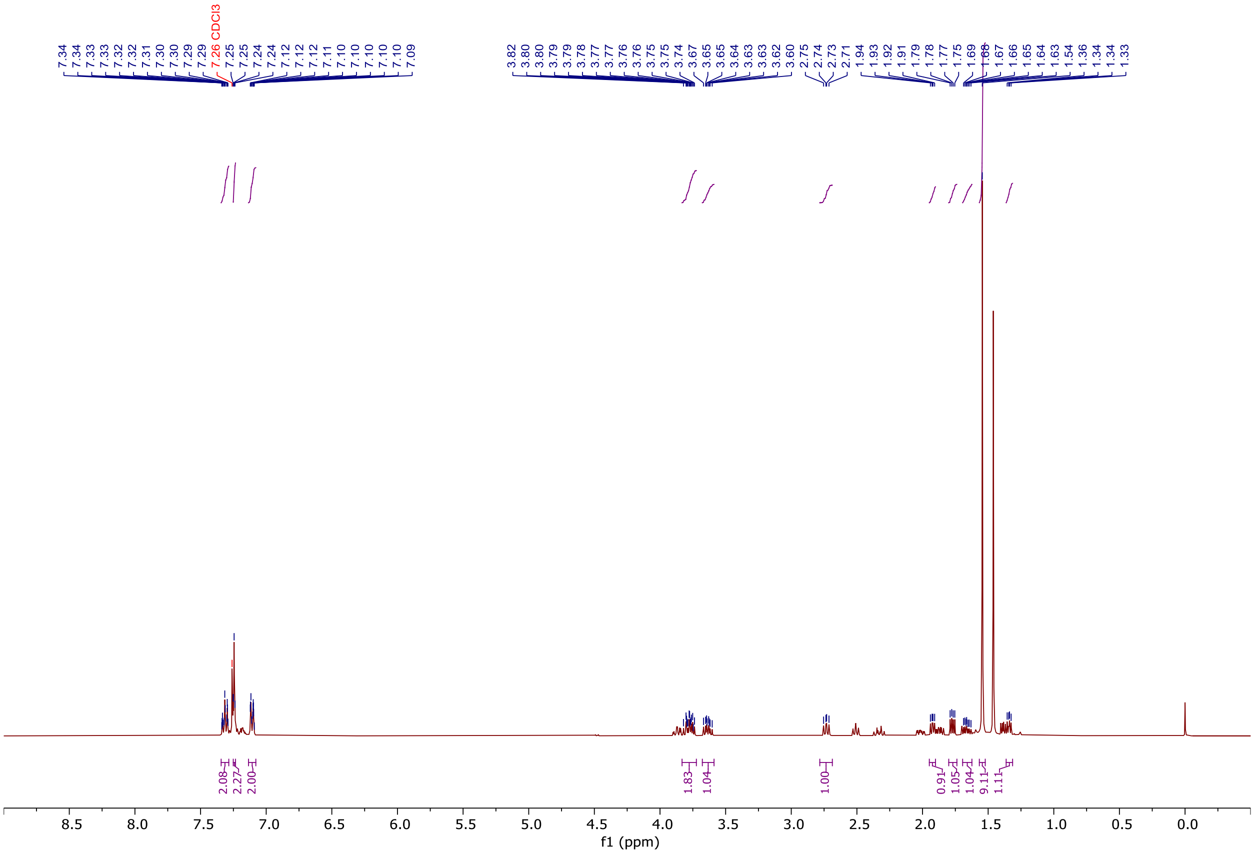

^{13}C NMR (101 MHz, $CDCl_3$)

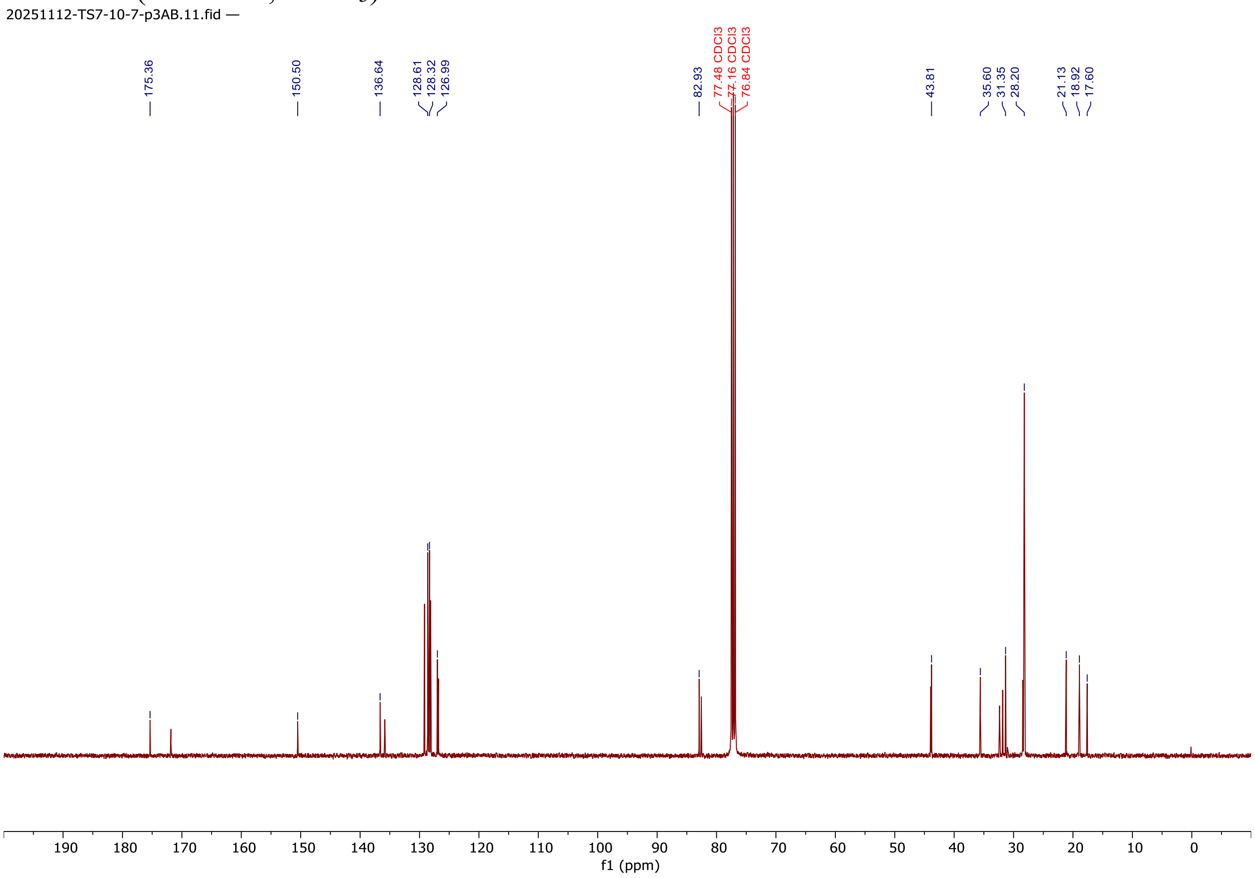

SFC (210 nm trace):
Racemate, synthesized from $Rh_2(OAc)_4$

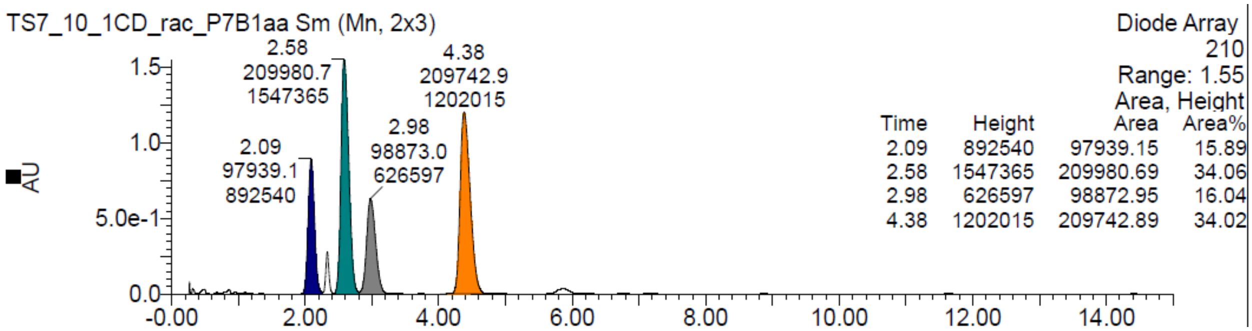

Time	Height	Area	Area%
2.09	892540	97939.15	15.89
2.58	1547365	209980.69	34.06
2.98	626597	98872.95	16.04
4.38	1202015	209742.89	34.02

Chiral, synthesized from Rh_2(*S*-*p*-PhTPCP)$_4$, 34% *ee*

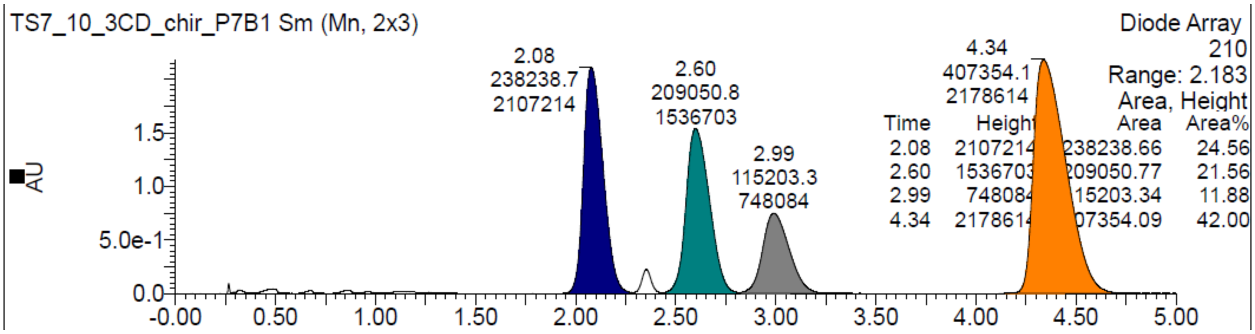

Time	Height	Area	Area%
2.08	2107214	238238.66	24.56
2.60	1536703	209050.77	21.56
2.99	748084	115203.34	11.88
4.34	2178614	407354.09	42.00

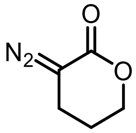

3-Diazotetrahydro-2H-pyran-2-one (8a)

^{1}H NMR (400 MHz, $CDCl_3$)

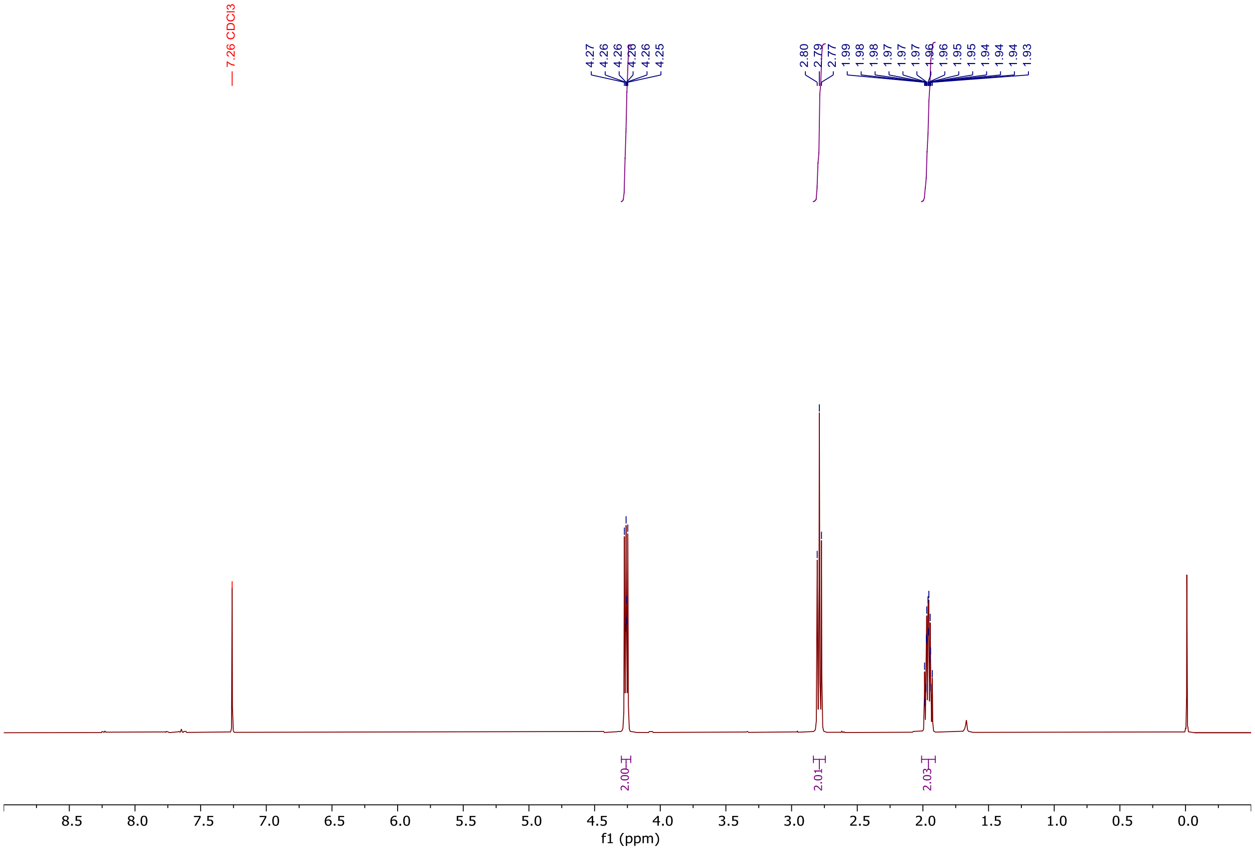

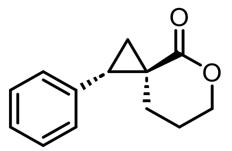

(1*S*,3*R*)-1-Phenyl-5-oxaspiro[2.5]octan-4-one (10a)

^{1}H NMR (400 MHz, $CDCl_3$)

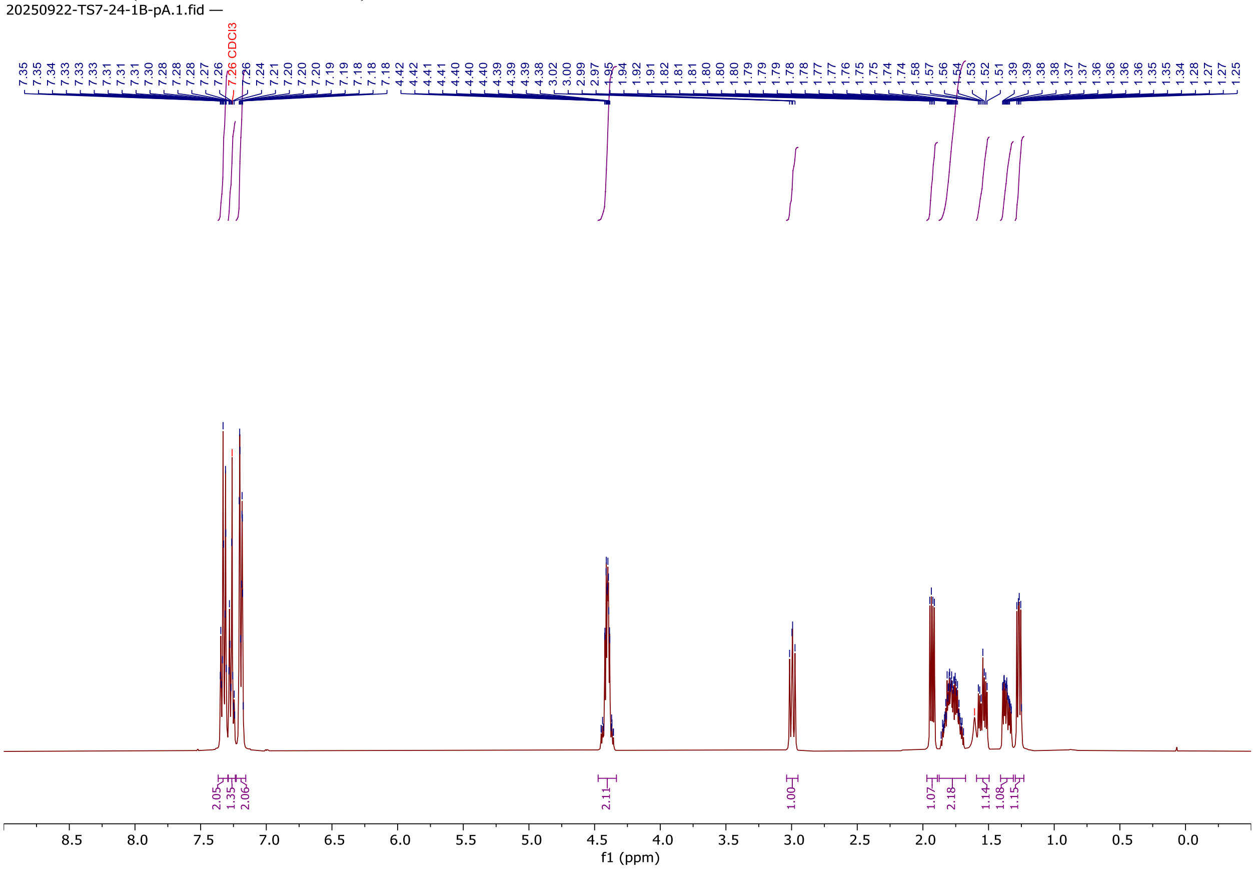

^{13}C NMR (101 MHz, $CDCl_3$)

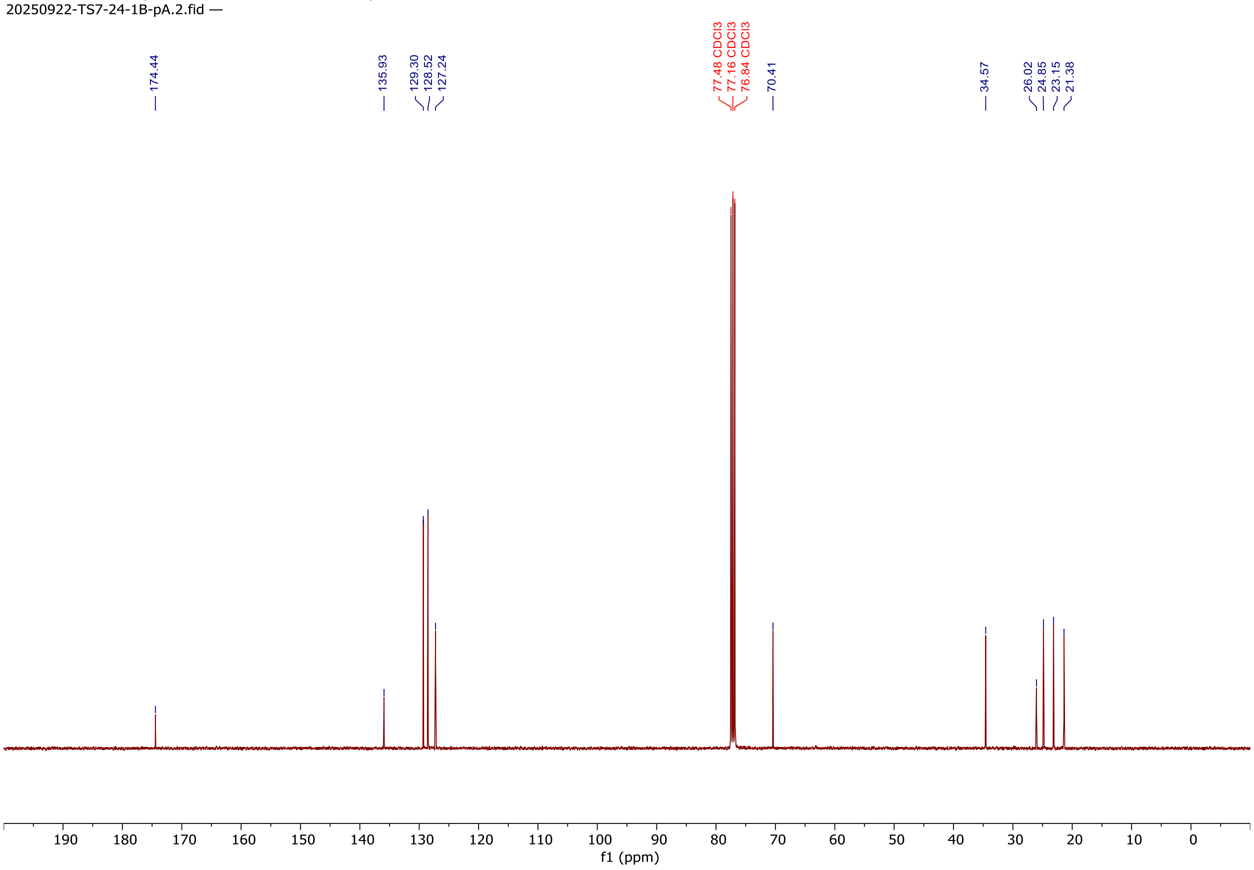

SFC (210 nm trace):
Racemate, synthesized from $Rh_2(OAc)_4$

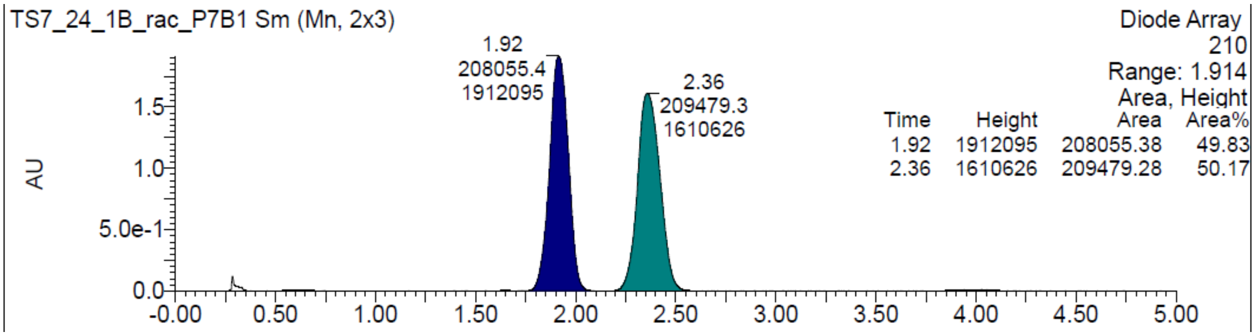

Time	Height	Area	Area%
1.92	1912095	208055.38	49.83
2.36	1610626	209479.28	50.17

Chiral, synthesized from Rh_2(*S*-*p*-PhTPCP)$_4$, 90% *ee*

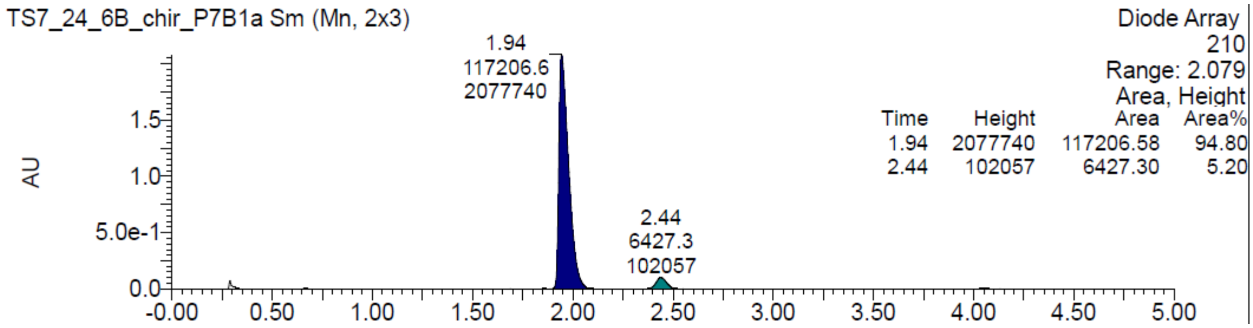

Time	Height	Area	Area%
1.94	2077740	117206.58	94.80
2.44	102057	6427.30	5.20

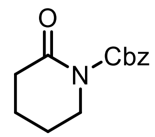

Benzyl 2-oxopiperidine-1-carboxylate (SI-2)

^{1}H NMR (400 MHz, $CDCl_3$)

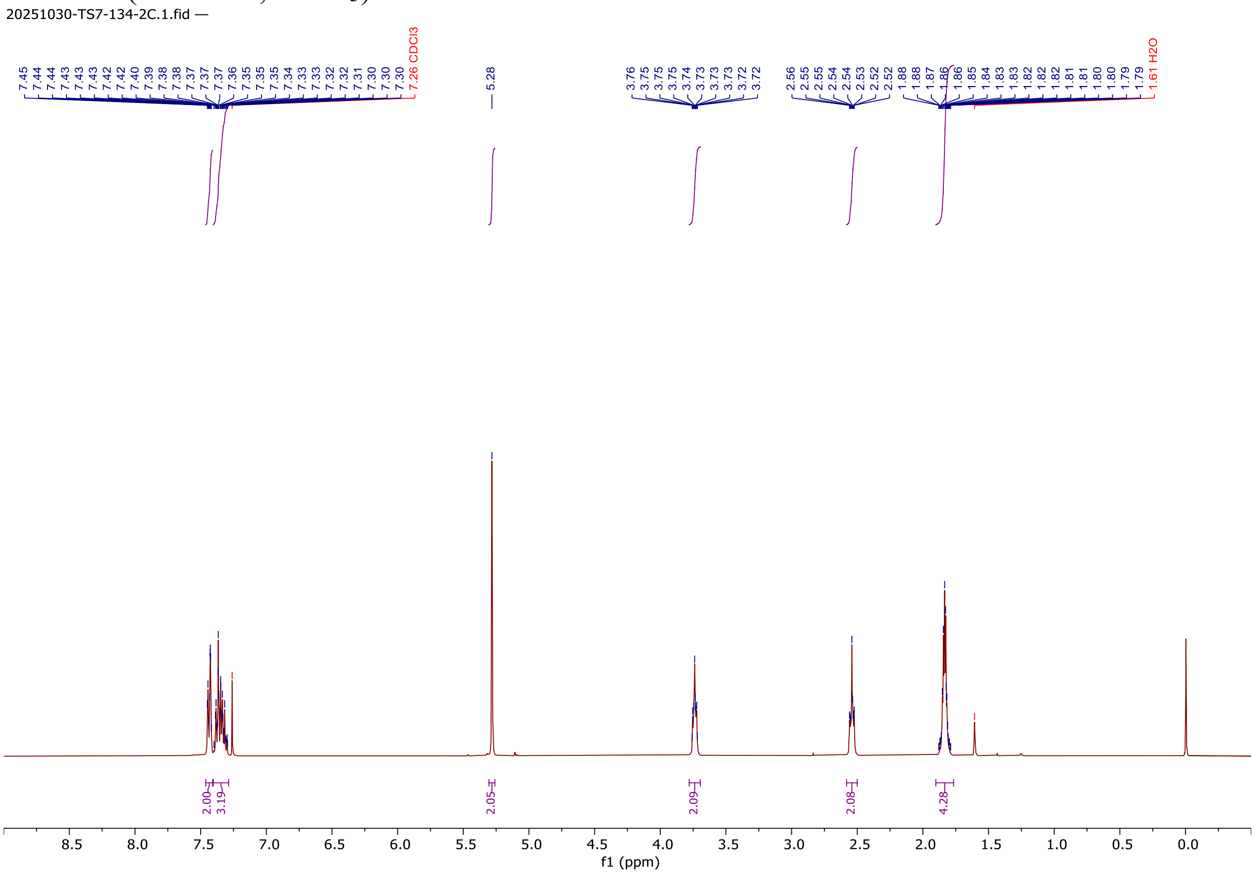

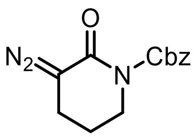

Benzyl 3-diazo-2-oxopiperidine-1-carboxylate (8b)

^{1}H NMR (400 MHz, $CDCl_3$)

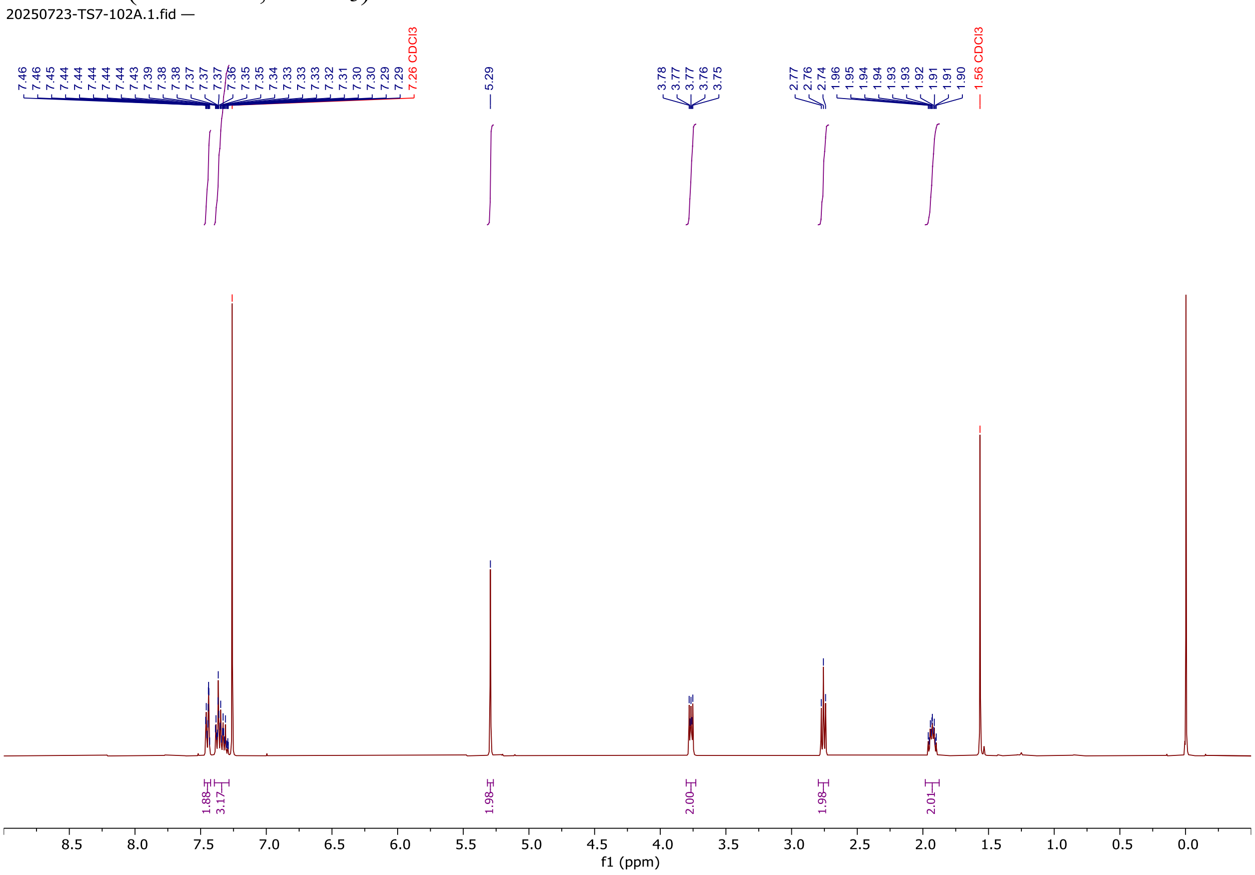

^{13}C NMR (101 MHz, $CDCl_3$)

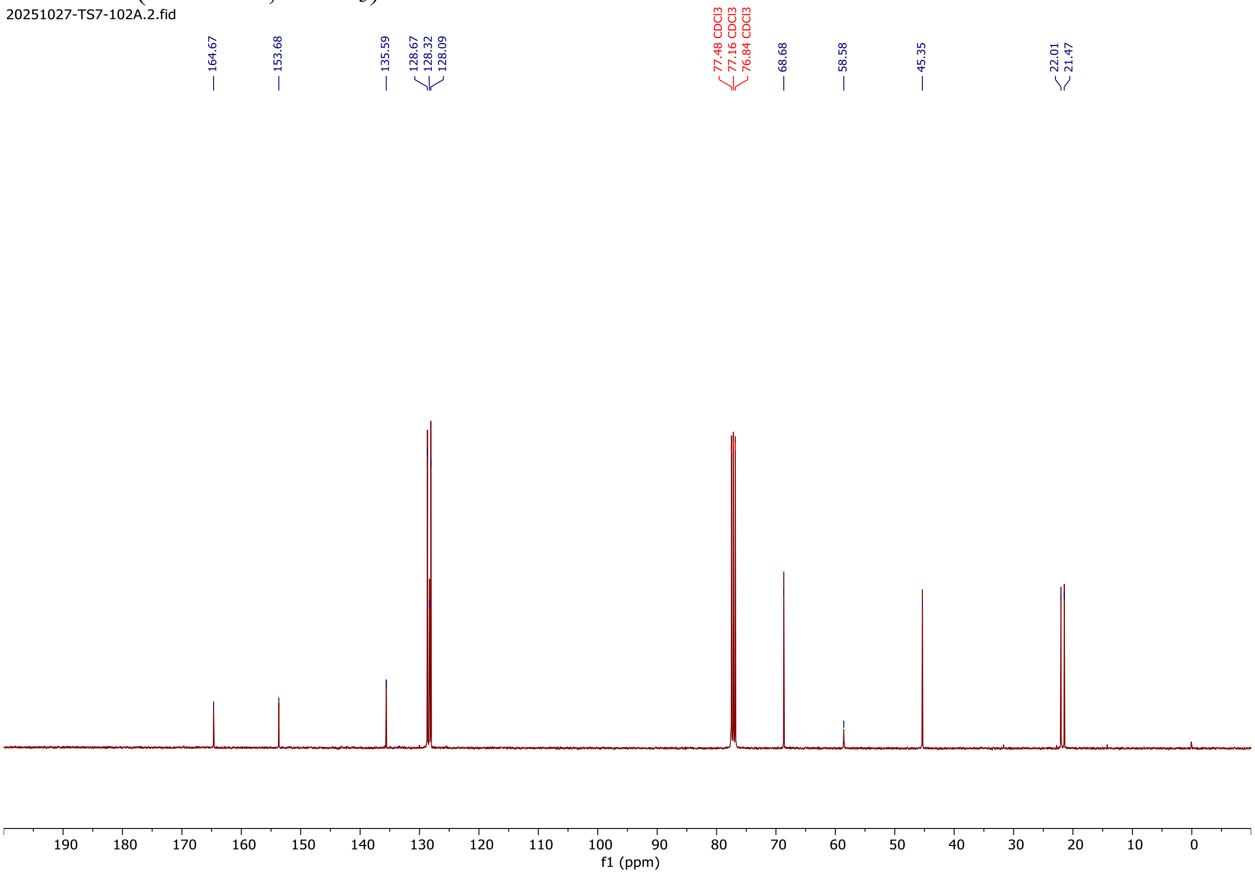

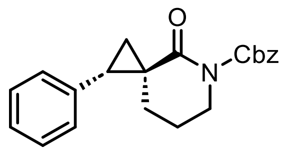

(1*S*,3*R*)-Benzyl 4-oxo-1-phenyl-5-azaspiro[2.5]octane-5-carboxylate (10b)

^{1}H NMR (400 MHz, $CDCl_3$)

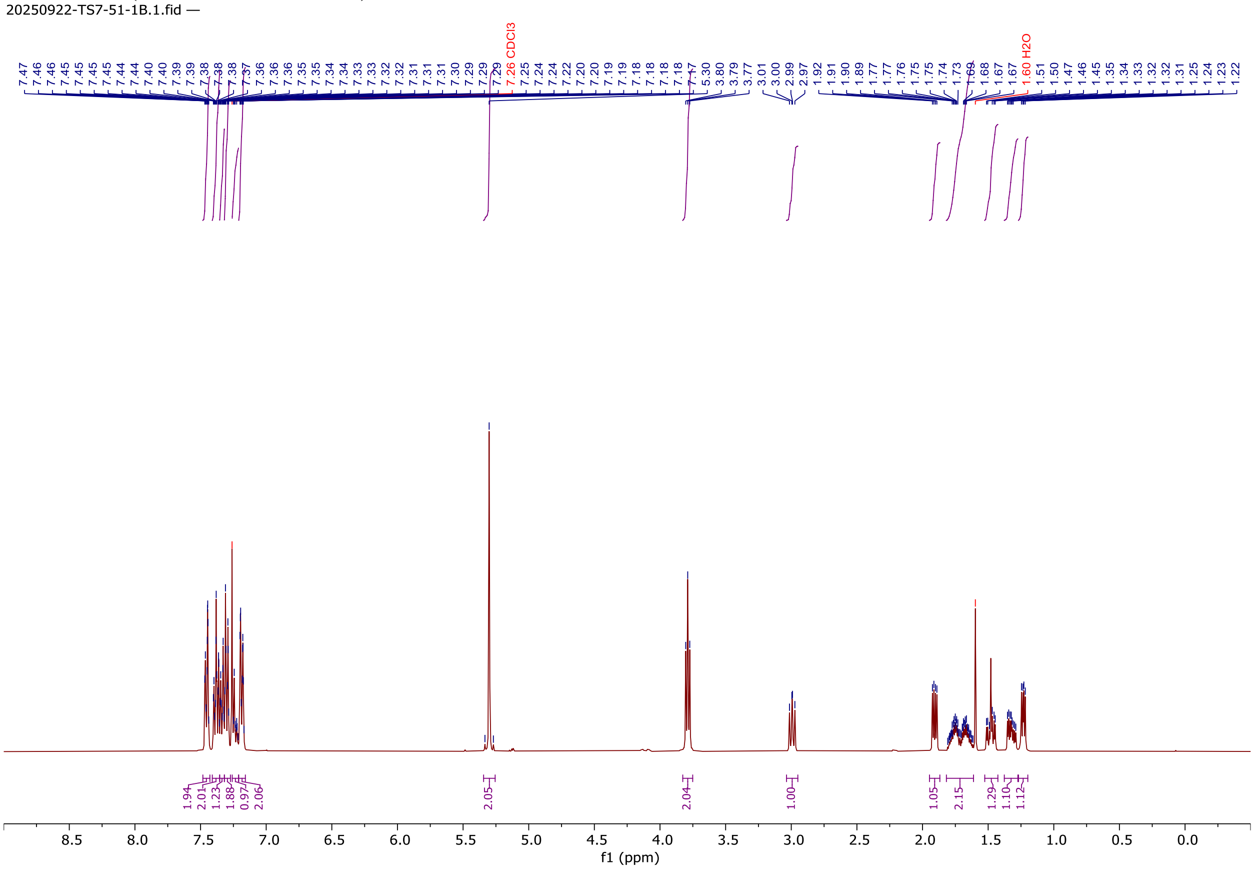

^{13}C NMR (101 MHz, $CDCl_3$)

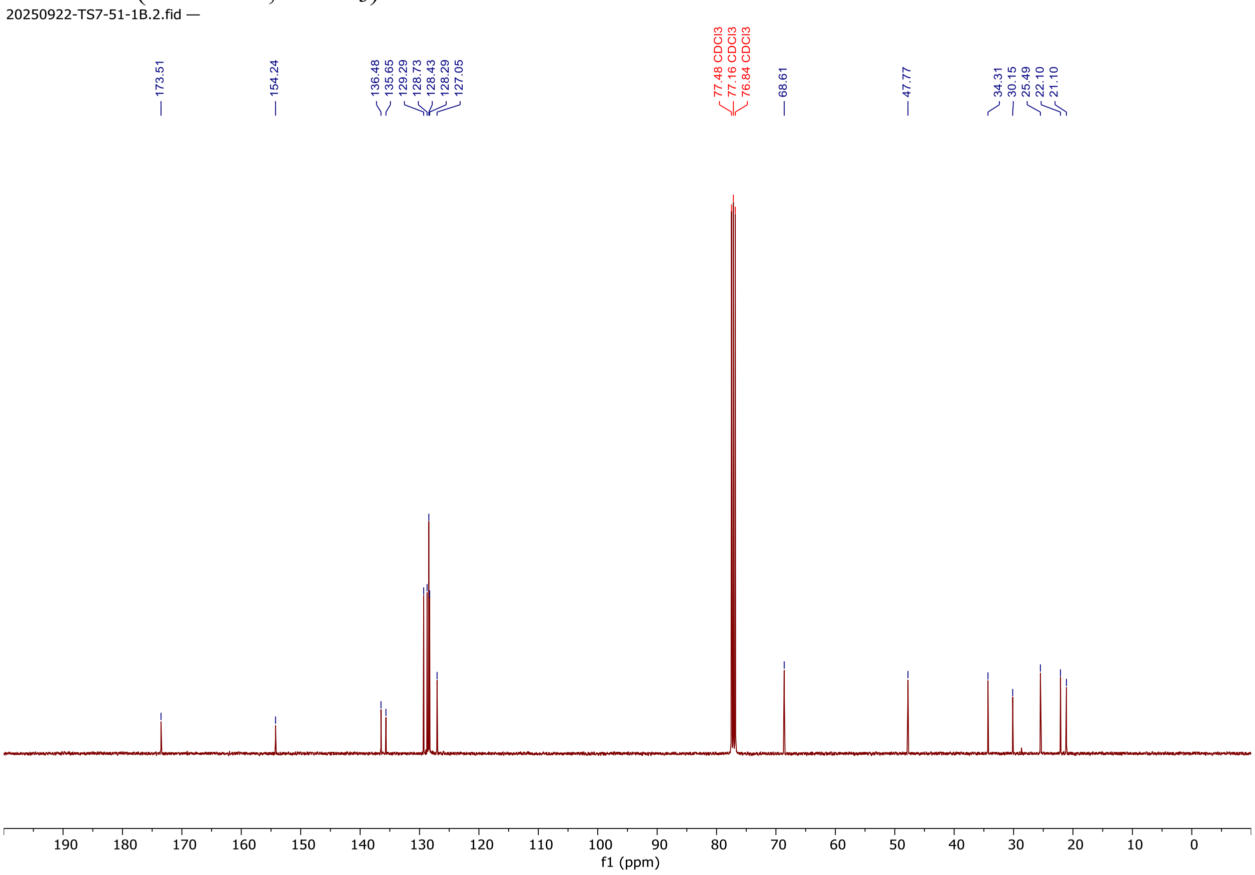

Chiral HPLC (210 nm trace):
Racemate, synthesized from $Rh_2(OAc)_4$

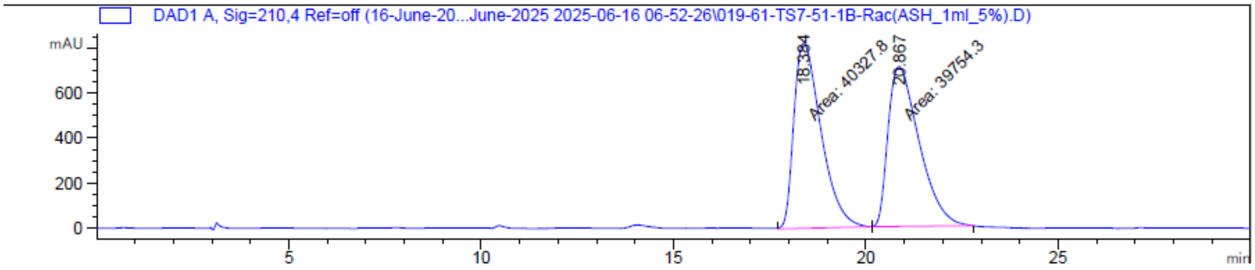

Signal 1: DAD1 A, Sig=210,4 Ref=off

Peak #	RetTime [min]	Type	Width [min]	Area [mAU*s]	Height [mAU]	Area %
1	18.384	MM	0.8188	4.03278e4	820.89966	50.3580
2	20.867	MM	0.9368	3.97543e4	707.27411	49.6420
Totals :				8.00821e4	1528.17377	

Chiral, synthesized from Rh_2(*S*-*p*-PhTPCP)$_4$, 95% *ee*

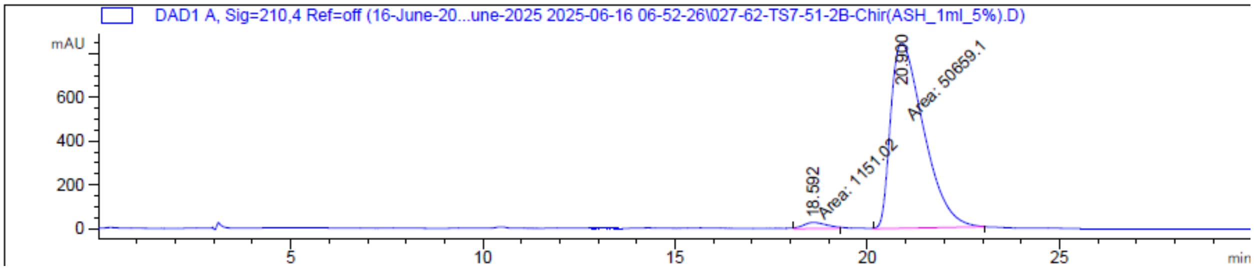

Signal 1: DAD1 A, Sig=210,4 Ref=off

Peak #	RetTime [min]	Type	Width [min]	Area [mAU*s]	Height [mAU]	Area %
1	18.592	MM	0.6962	1151.02405	27.55576	2.2216
2	20.900	MM	0.9971	5.06591e4	846.77551	97.7784
Totals :				5.18101e4	874.33127	

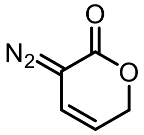

3-diazo-3,6-dihydro-2H-pyran-2-one (11)

^{1}H NMR (400 MHz, $CDCl_3$)

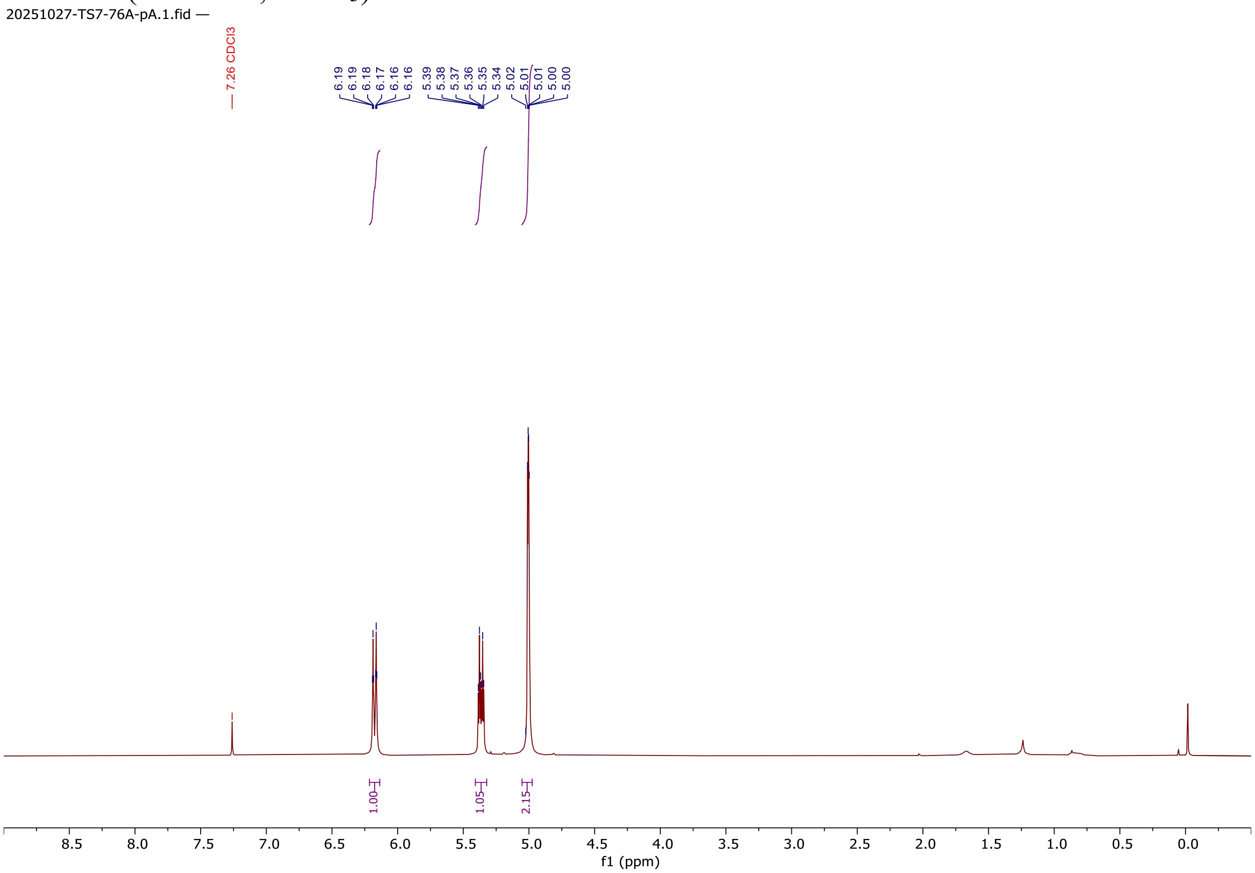

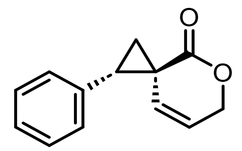

(1*S*,3*R*)-1-phenyl-5-oxaspiro[2.5]oct-7-en-4-one (12)

^{1}H NMR (400 MHz, $CDCl_3$)

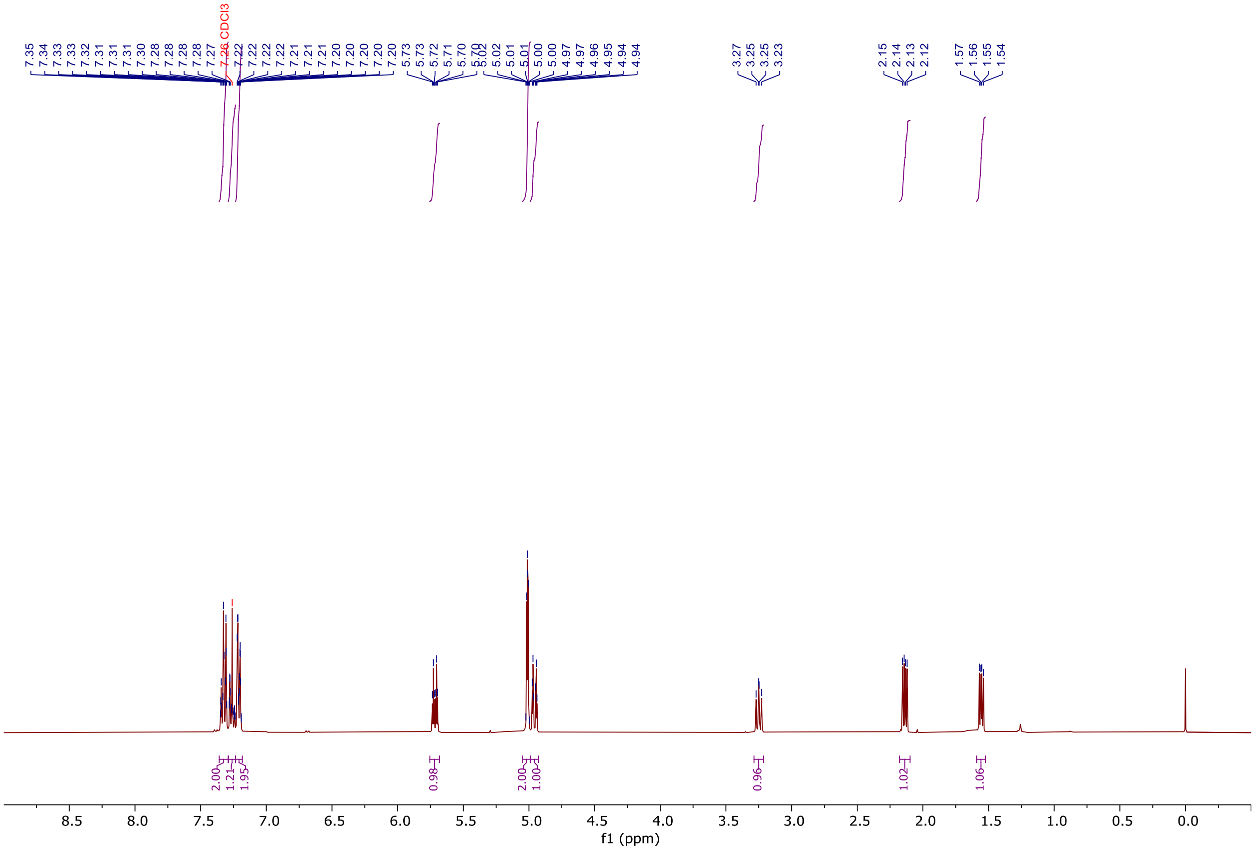

Chiral HPLC (210 nm trace):
Racemate, synthesized from $Rh_2(OAc)_4$

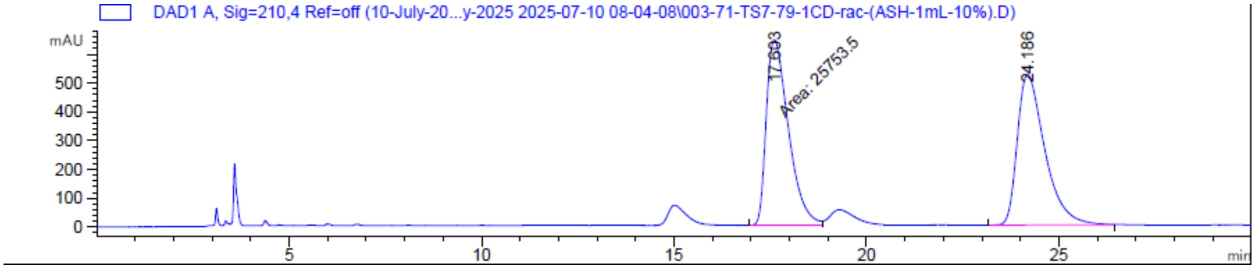

Signal 1: DAD1 A, Sig=210,4 Ref=off

Peak #	RetTime [min]	Type	Width [min]	Area [mAU*s]	Height [mAU]	Area %
1	17.603	MF	0.6670	2.57535e4	643.53113	49.8550
2	24.186	VV R	0.5818	2.59033e4	522.56952	50.1450

Totals : 5.16567e4 1166.10065

Chiral, synthesized from Rh_2(*S*-*p*-PhTPCP)$_4$, 30% *ee*

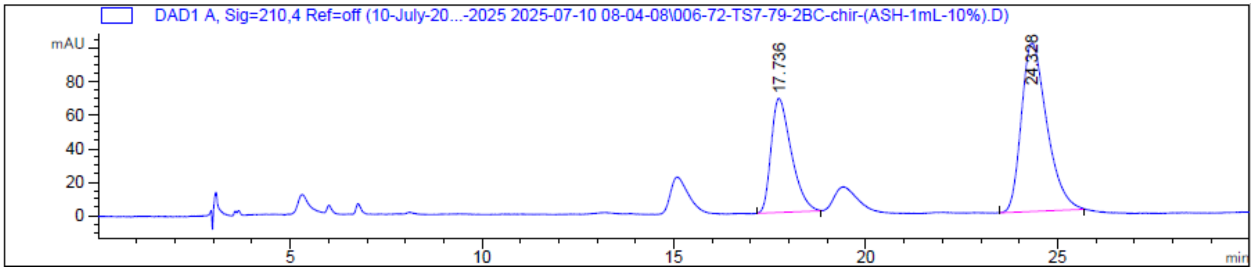

Signal 1: DAD1 A, Sig=210,4 Ref=off

Peak #	RetTime [min]	Type	Width [min]	Area [mAU*s]	Height [mAU]	Area %
1	17.736	VV R	0.4285	2500.82300	68.33012	34.9203
2	24.328	VV R	0.5425	4660.69287	100.88068	65.0797

Totals : 7161.51587 169.21081

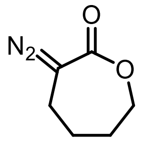

3-diazooxepan-2-one (13)

^{1}H NMR (400 MHz, $CDCl_3$)

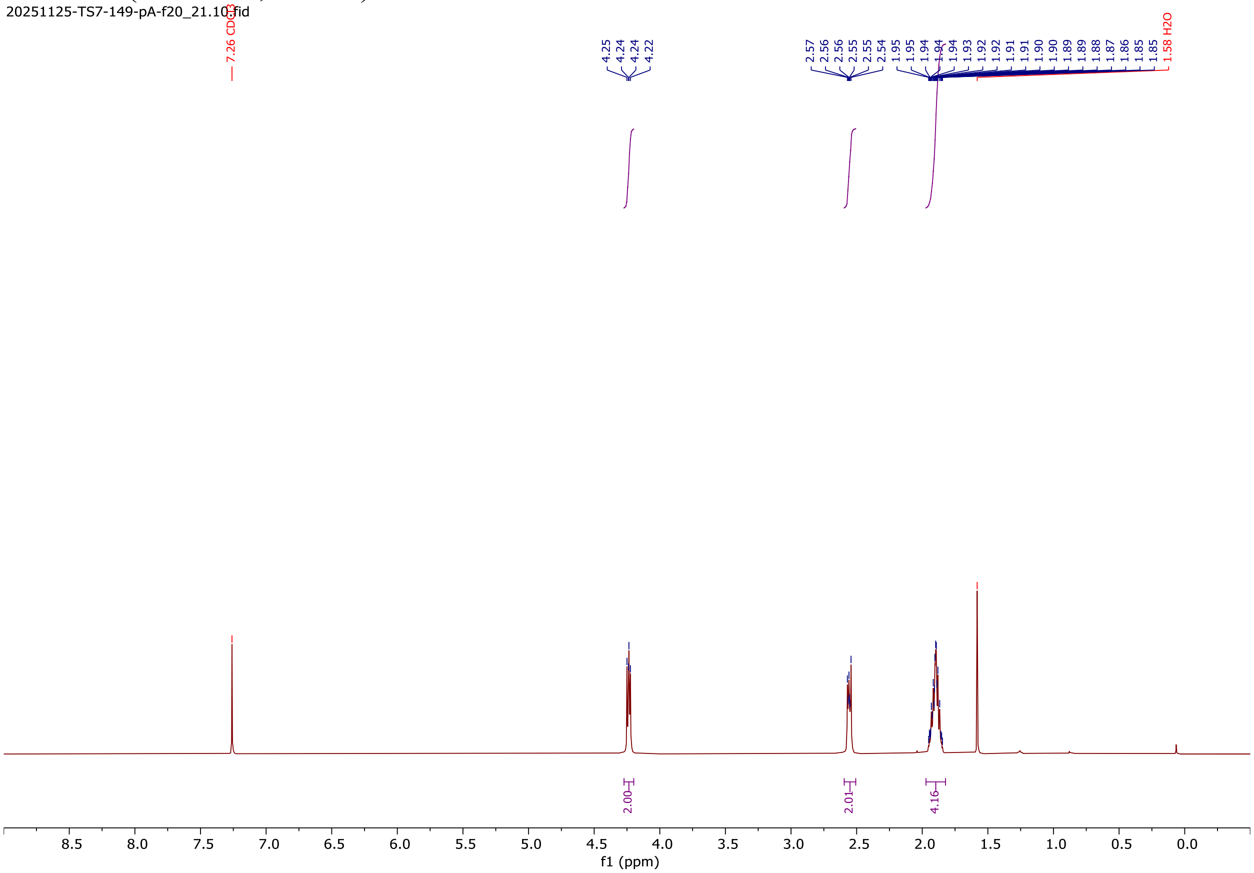

^{13}C NMR (101 MHz, $CDCl_3$)

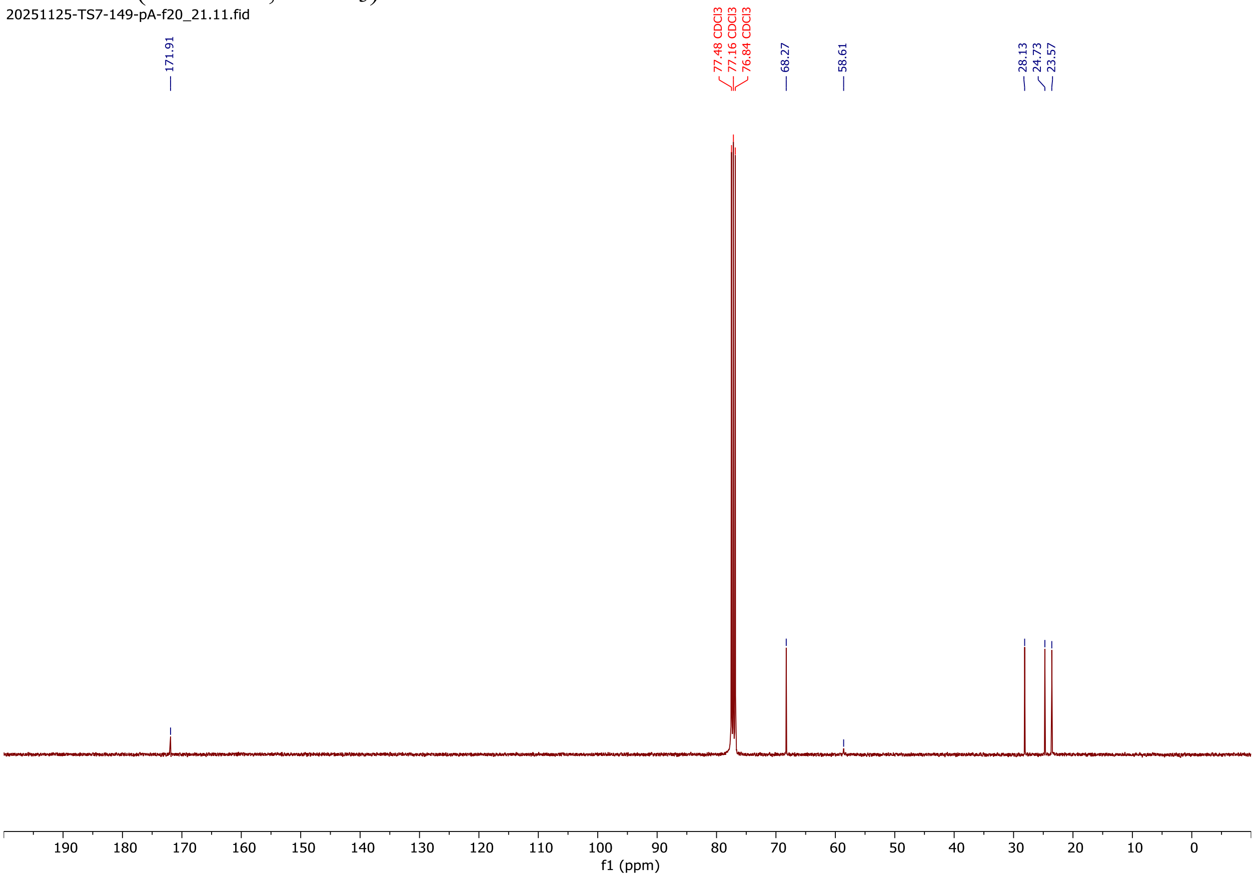

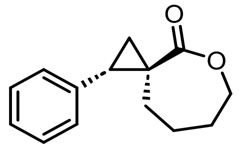

(1*S*,3*R*)-1-phenyl-5-oxaspiro[2.6]nonan-4-one (14a)

^{1}H NMR (400 MHz, $CDCl_3$)

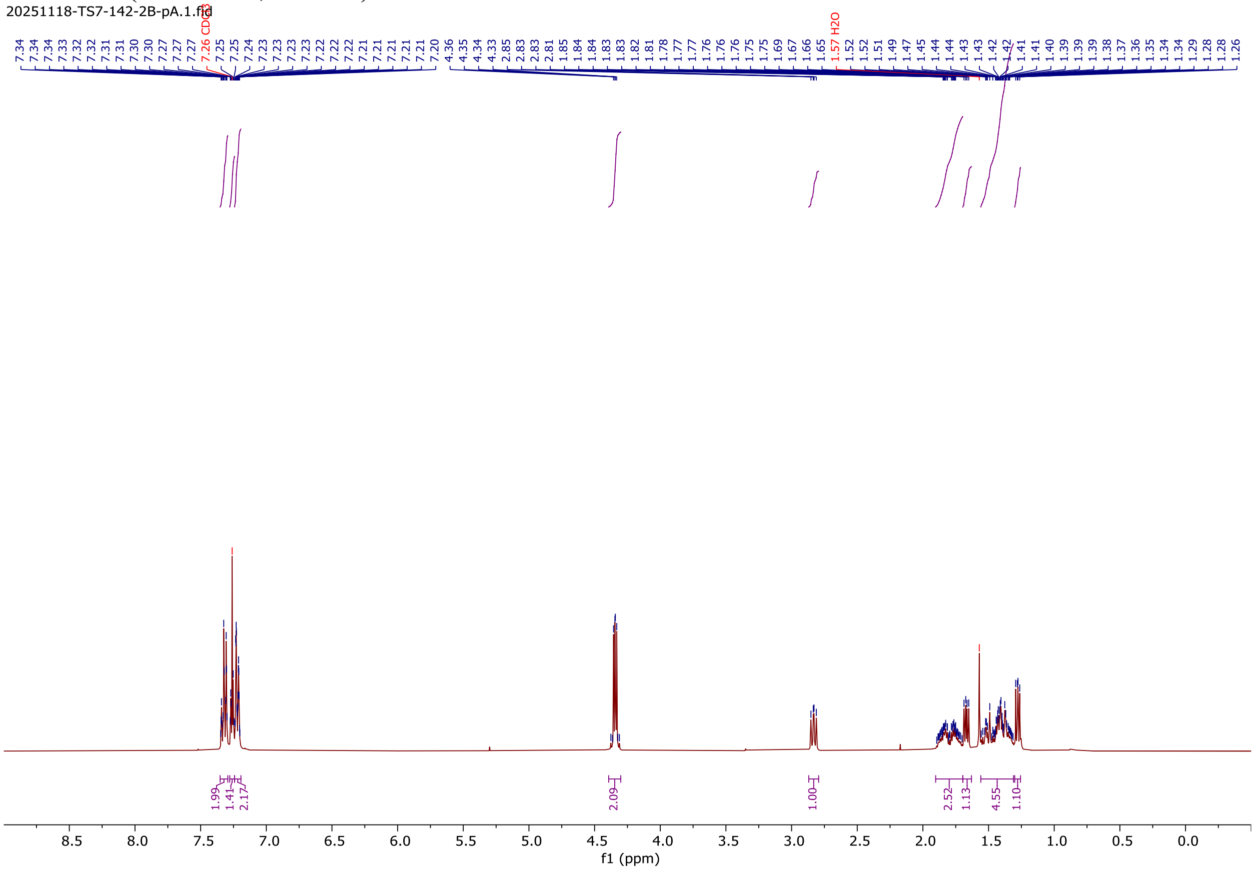

^{13}C NMR (101 MHz, $CDCl_3$)

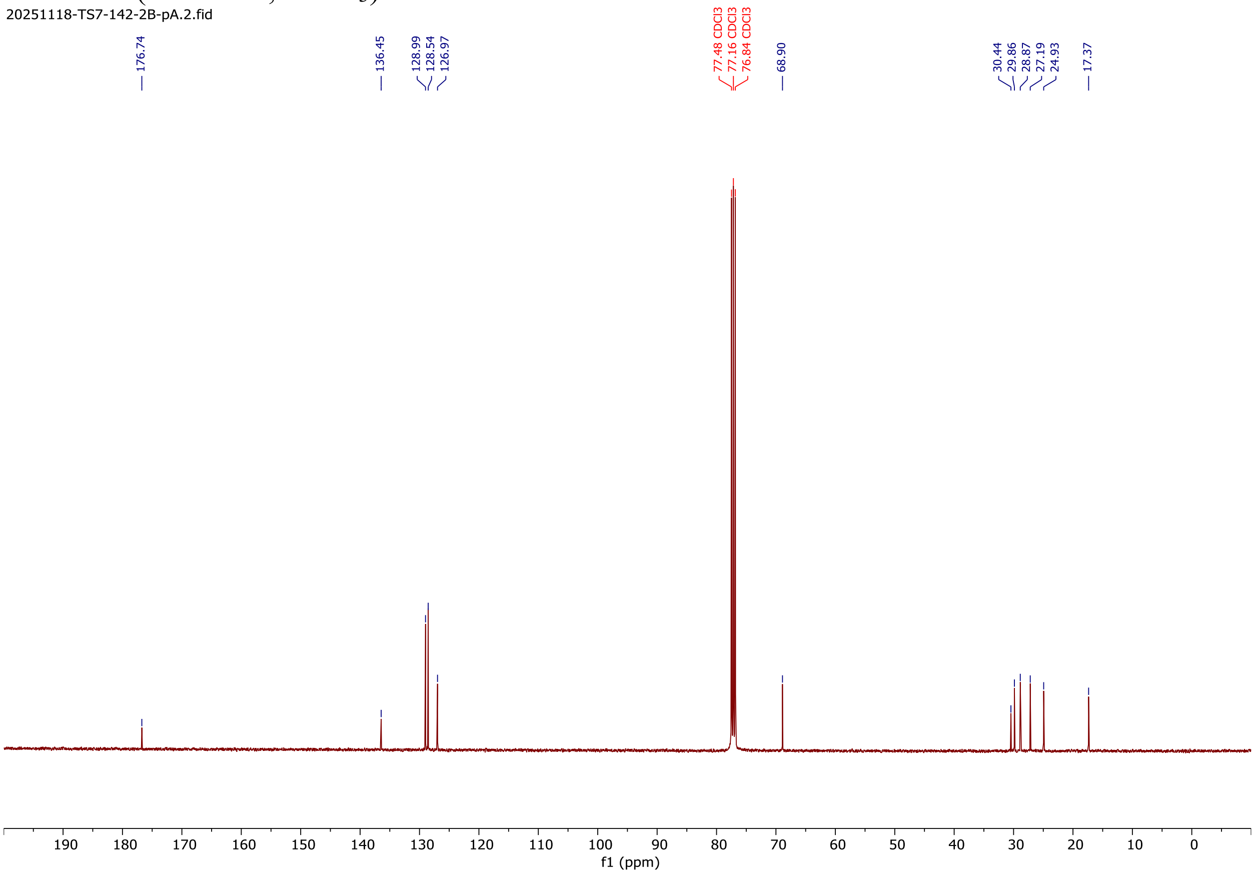

Chiral HPLC (210 nm trace):
Racemate, synthesized from $Rh_2(OAc)_4$

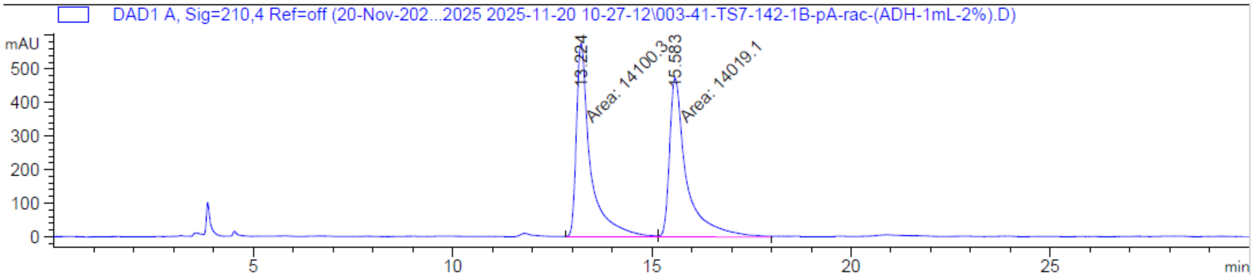

Signal 1: DAD1 A, Sig=210,4 Ref=off

Peak #	RetTime [min]	Type	Width [min]	Area [mAU*s]	Height [mAU]	Area %
1	13.224	MF	0.4082	1.41003e4	575.68787	50.1444
2	15.583	FM	0.4938	1.40191e4	473.17490	49.8556

Totals : 2.81195e4 1048.86276

Chiral, synthesized from Rh_2(*S*-*p*-PhTPCP)$_4$, 99% *ee*

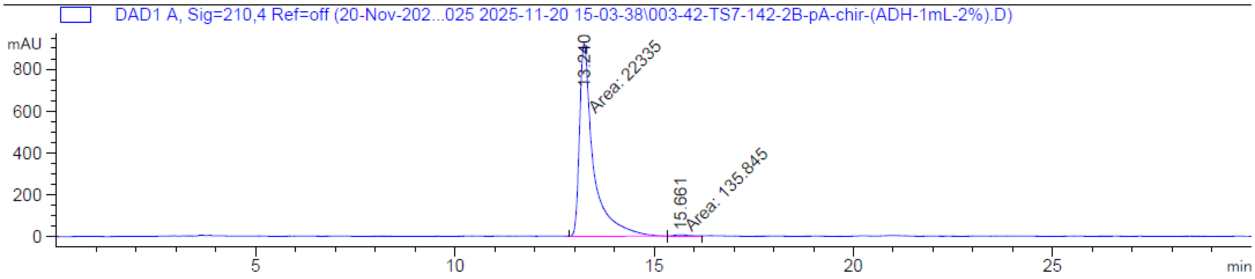

Signal 1: DAD1 A, Sig=210,4 Ref=off

Peak #	RetTime [min]	Type	Width [min]	Area [mAU*s]	Height [mAU]	Area %
1	13.240	MF	0.4040	2.23350e4	921.51917	99.3955
2	15.661	FM	0.3711	135.84544	6.10120	0.6045

Totals : 2.24708e4 927.62036

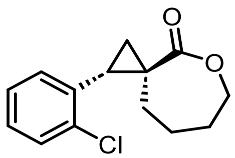

(1*R*,3*R*)-1-(2-chlorophenyl)-5-oxaspiro[2.6]nonan-4-one (14b)

^{1}H NMR (400 MHz, $CDCl_3$)

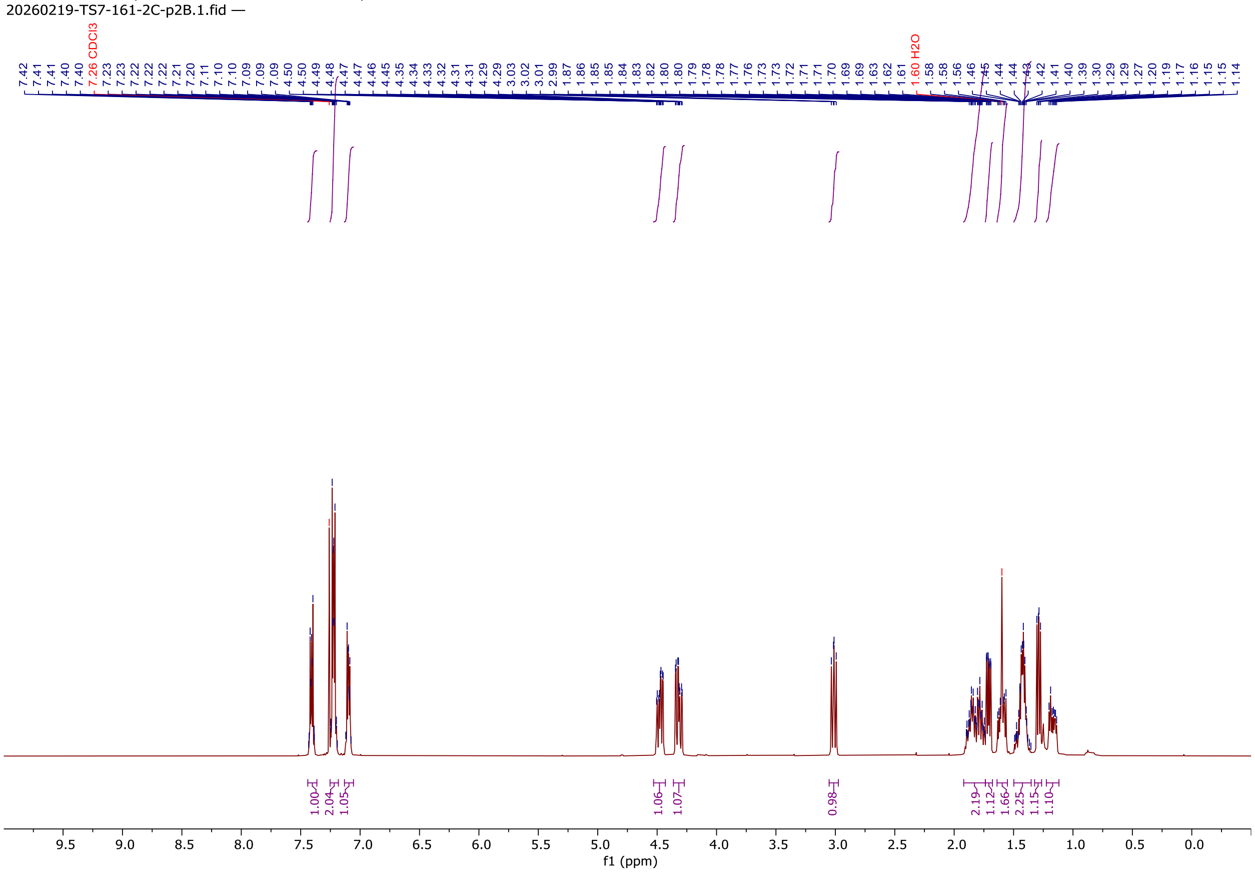

^{13}C NMR (101 MHz, $CDCl_3$)

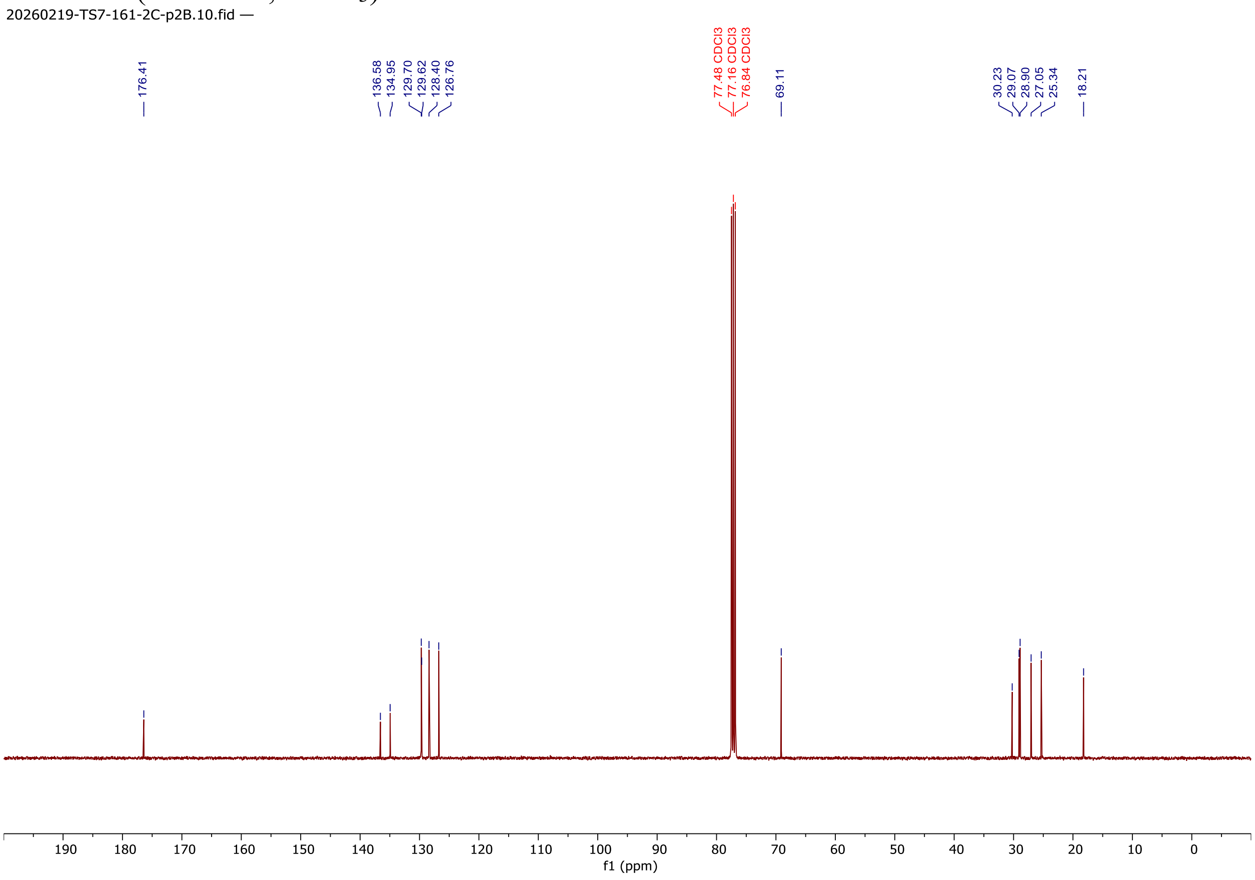

Chiral HPLC (210 nm trace):
Racemate, synthesized from $Rh_2(OAc)_4$

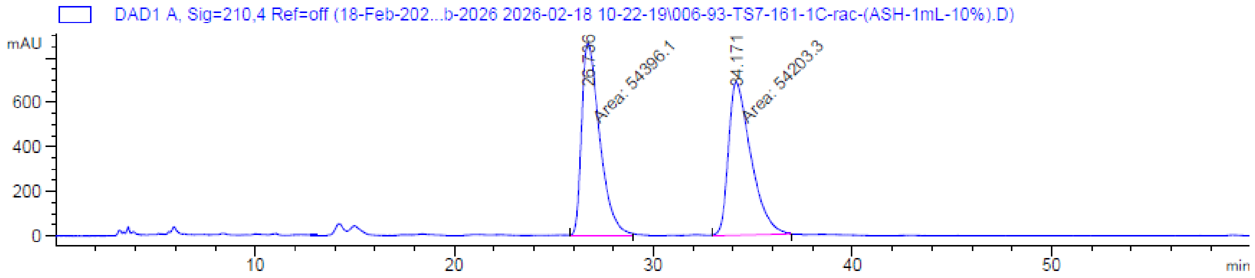

Signal 1: DAD1 A, Sig=210,4 Ref=off

Peak #	RetTime [min]	Type	Width [min]	Area [mAU*s]	Height [mAU]	Area %
1	26.736	MM	1.0507	5.43961e4	862.82281	50.0888
2	34.171	MM	1.3079	5.42033e4	690.73376	49.9112

Totals : 1.08599e5 1553.55658

Chiral, synthesized from Rh_2(*S*-*p*-PhTPCP)$_4$, 99% *ee*

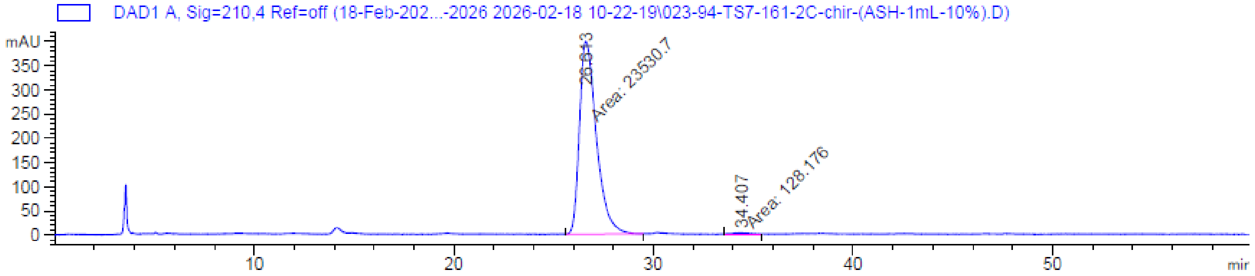

Signal 1: DAD1 A, Sig=210,4 Ref=off

Peak #	RetTime [min]	Type	Width [min]	Area [mAU*s]	Height [mAU]	Area %
1	26.613	MM	0.9827	2.35307e4	399.09406	99.4582
2	34.407	MM	0.7869	128.17557	2.71493	0.5418

Totals : 2.36589e4 401.80898

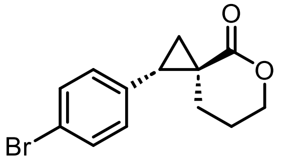

(1*S*,3*R*)-1-(4-Bromophenyl)-5-oxaspiro[2.5]octan-4-one (16a)

^{1}H NMR (400 MHz, $CDCl_3$)

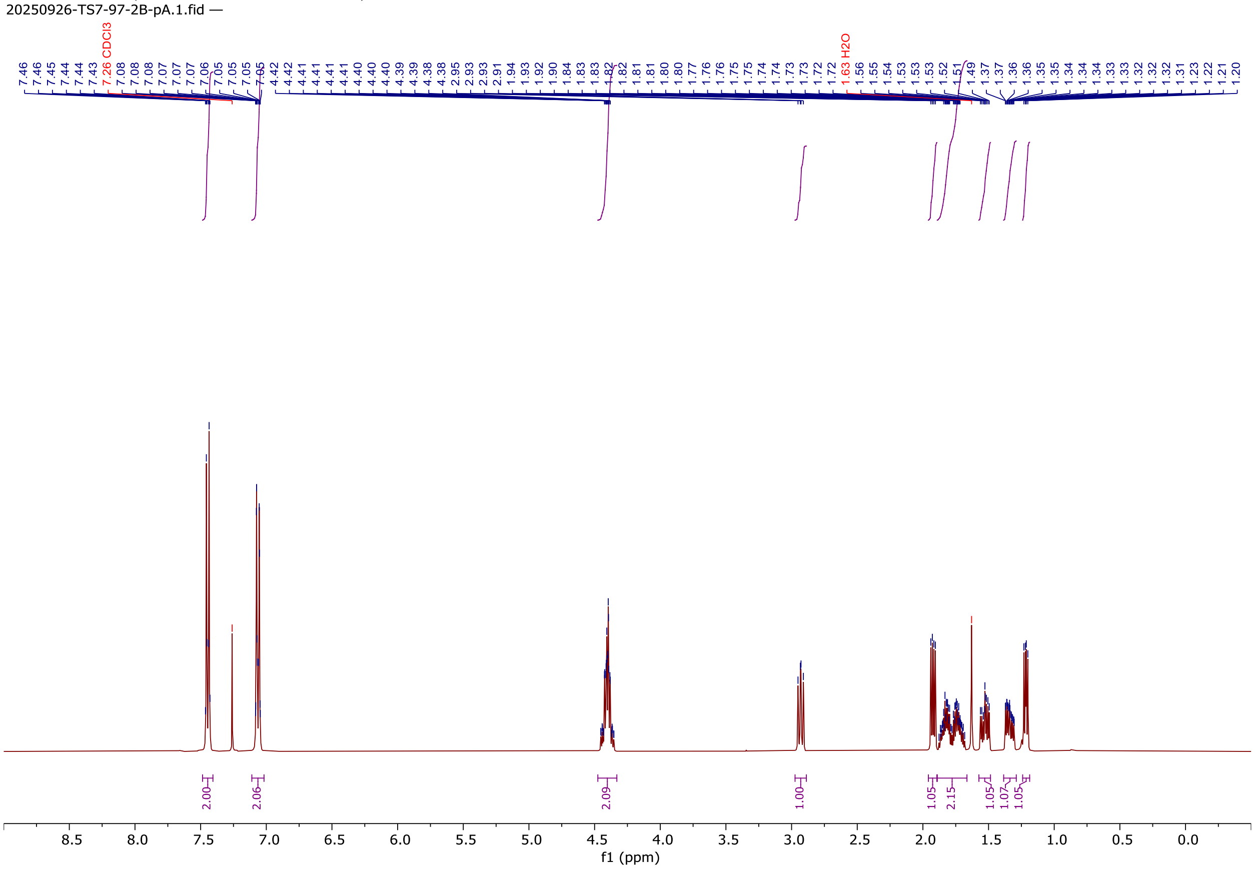

^{13}C NMR (101 MHz, $CDCl_3$)

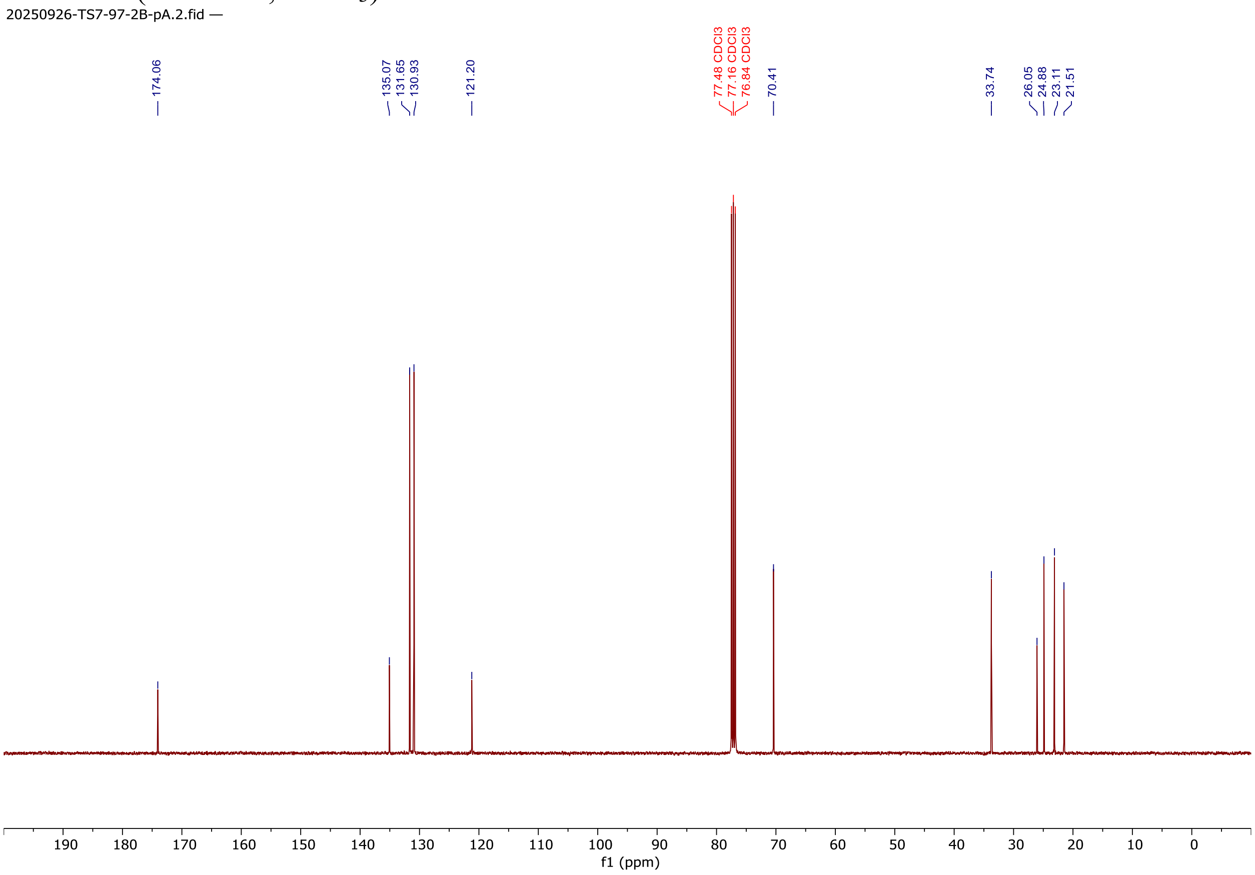

Chiral HPLC (210 nm trace):
Racemate, synthesized from $Rh_2(OAc)_4$

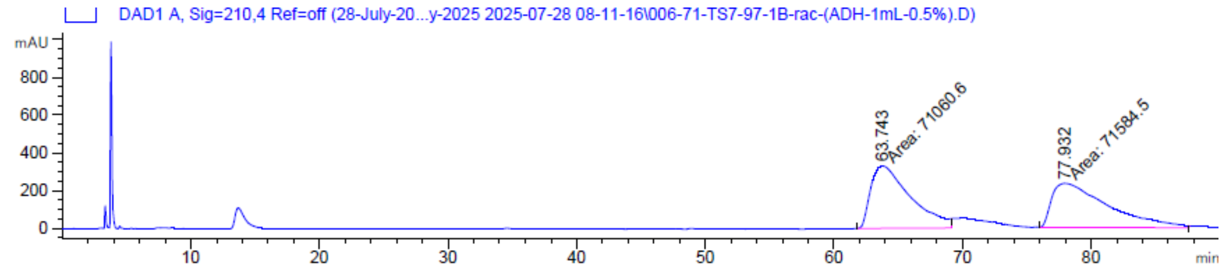

Signal 1: DAD1 A, Sig=210,4 Ref=off

Peak #	RetTime [min]	Type	Width [min]	Area [mAU*s]	Height [mAU]	Area %
1	63.743	MF	3.5876	7.10606e4	330.11880	49.8163
2	77.932	MF	5.0911	7.15845e4	234.34612	50.1837

Totals : 1.42645e5 564.46492

Chiral, synthesized from Rh_2(*S*-*p*-PhTPCP)$_4$, 84% *ee*

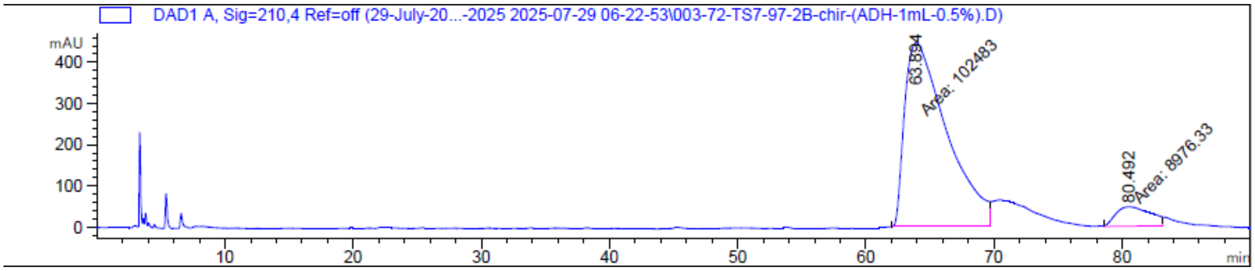

Signal 1: DAD1 A, Sig=210,4 Ref=off

Peak #	RetTime [min]	Type	Width [min]	Area [mAU*s]	Height [mAU]	Area %
1	63.894	MF	3.8249	1.02483e5	446.56631	91.9466
2	80.492	MM	3.1565	8976.32813	47.39595	8.0534

Totals : 1.11459e5 493.96227

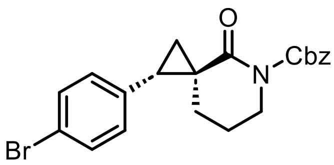

Benzyl (1*S*,3*R*)-1-(4-bromophenyl)-4-oxo-5-azaspiro[2.5]octane-5-carboxylate (16b)

^{1}H NMR (400 MHz, $CDCl_3$)

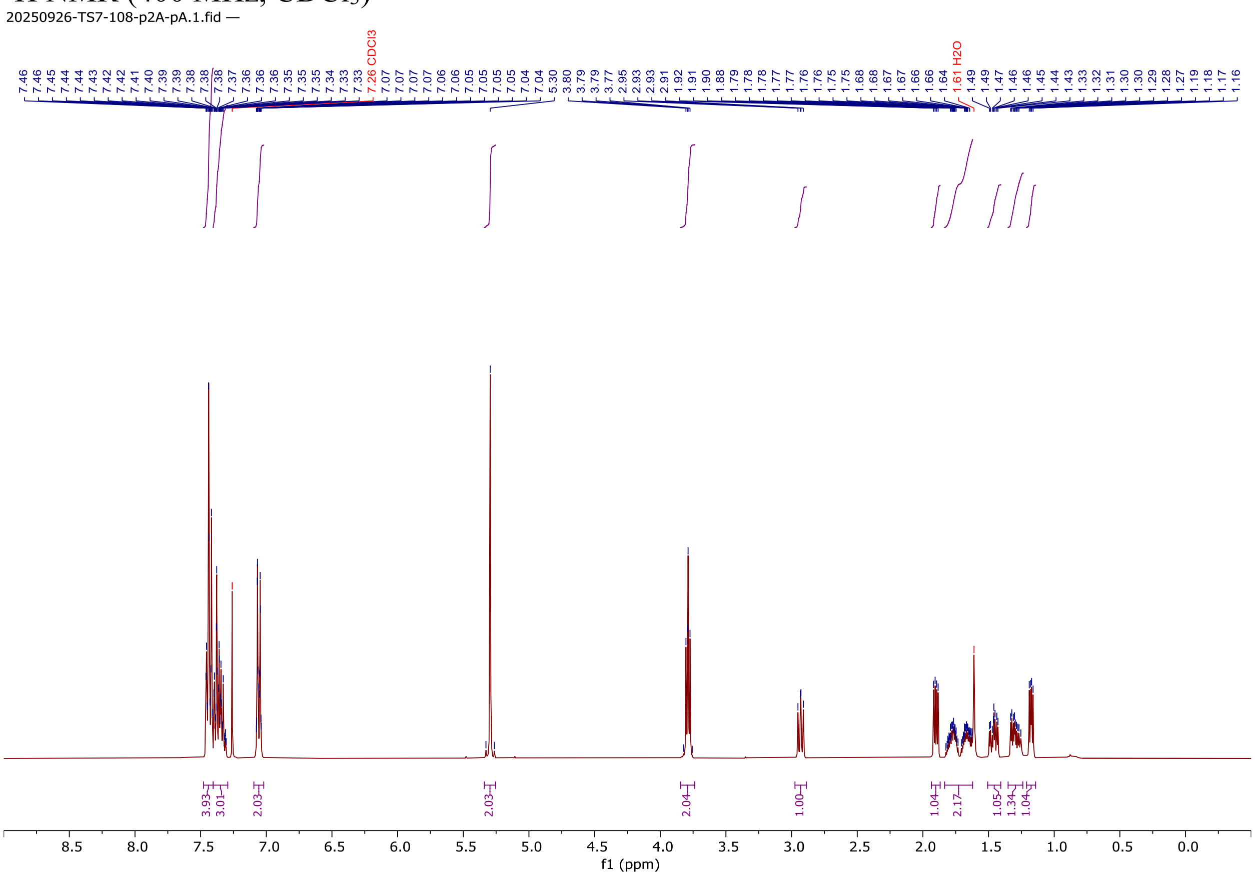

^{13}C NMR (101 MHz, $CDCl_3$)

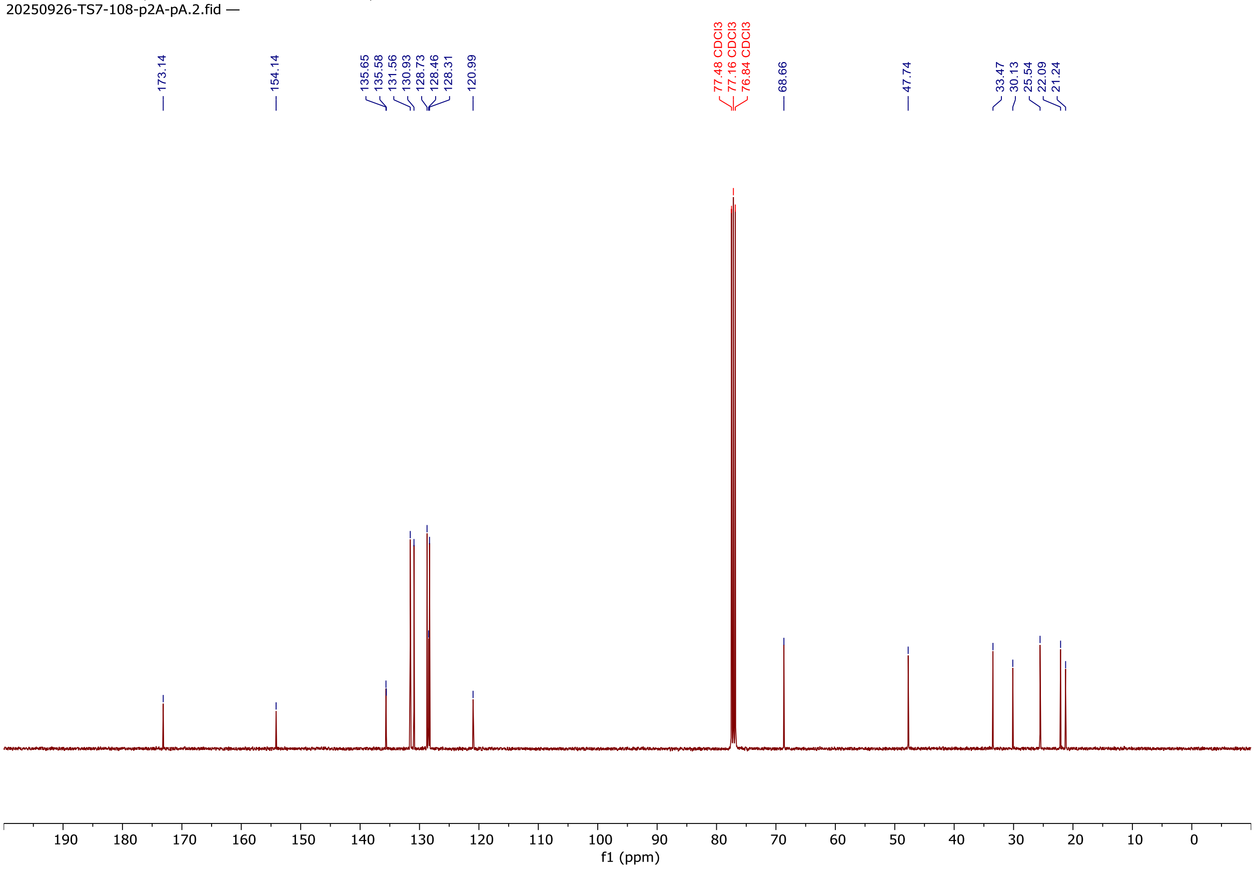

Chiral HPLC (210 nm trace):
Racemate, synthesized from $Rh_2(OAc)_4$

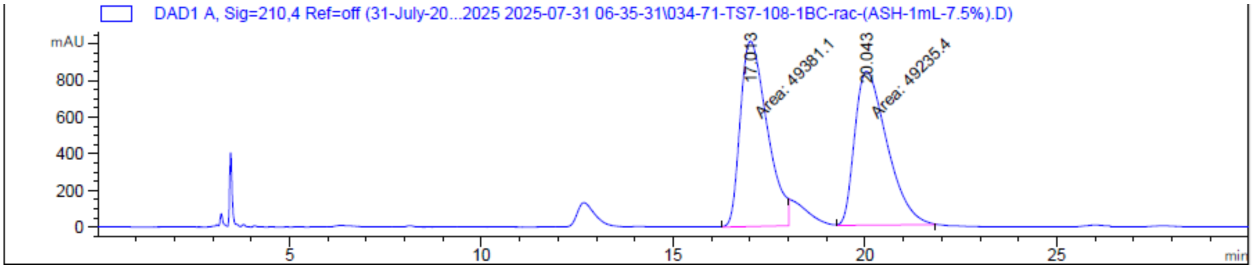

Signal 1: DAD1 A, Sig=210,4 Ref=off

Peak #	RetTime [min]	Type	Width [min]	Area [mAU*s]	Height [mAU]	Area %
1	17.013	MM	0.8133	4.93811e4	1011.96735	50.0739
2	20.043	MM	0.9772	4.92354e4	839.76227	49.9261
Totals :				9.86165e4	1851.72961	

Chiral, synthesized from Rh_2(*S*-*p*-PhTPCP)$_4$, 97% *ee*

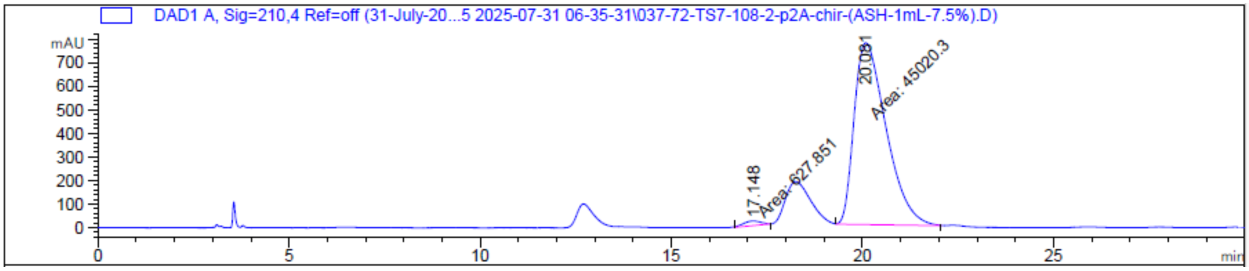

Signal 1: DAD1 A, Sig=210,4 Ref=off

Peak #	RetTime [min]	Type	Width [min]	Area [mAU*s]	Height [mAU]	Area %
1	17.148	MM	0.5412	627.85101	19.33437	1.3754
2	20.081	MM	0.9716	4.50203e4	772.29504	98.6246
Totals :				4.56482e4	791.62942	

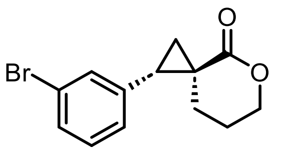

(1*S*,3*R*)-1-(3-Bromophenyl)-5-oxaspiro[2.5]octan-4-one (17a)

^{1}H NMR (400 MHz, $CDCl_3$)

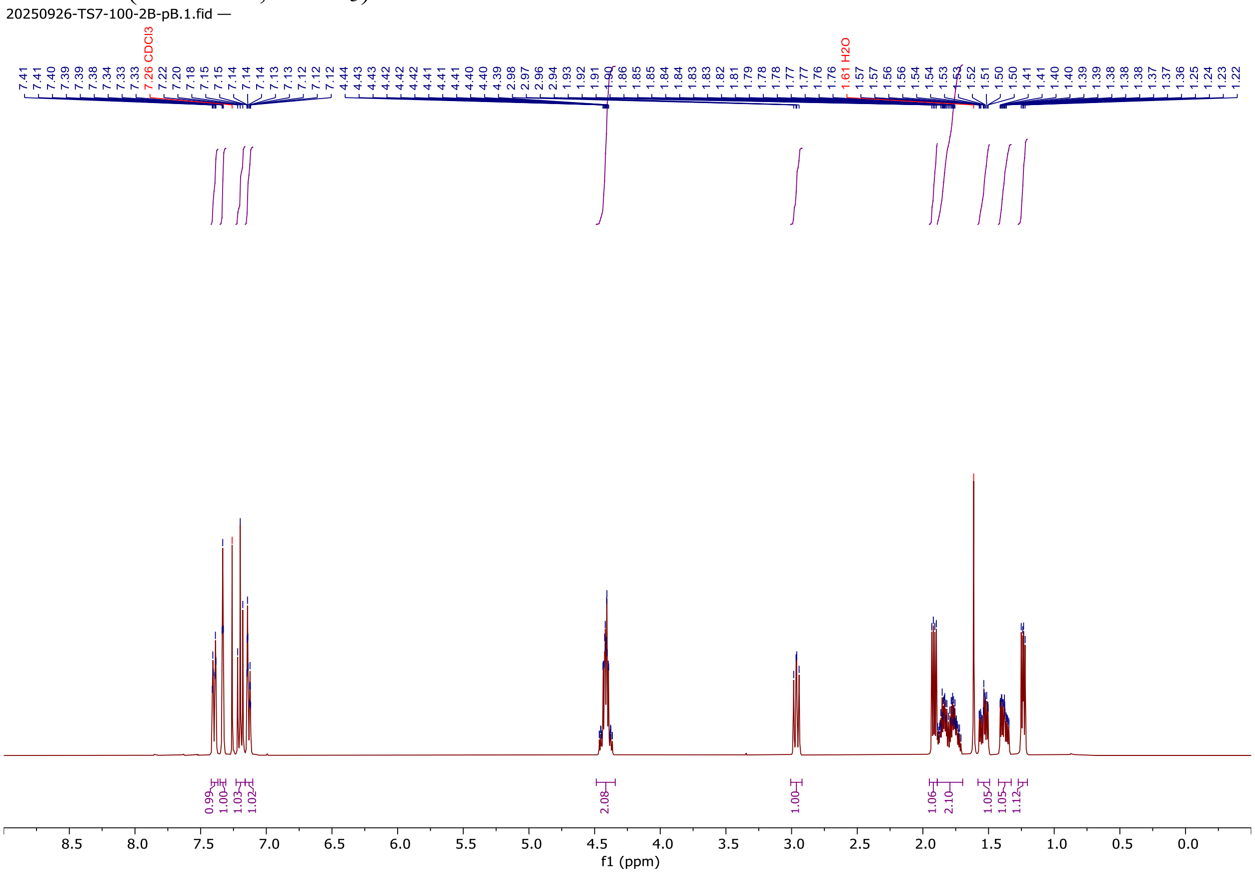

^{13}C NMR (101 MHz, $CDCl_3$)

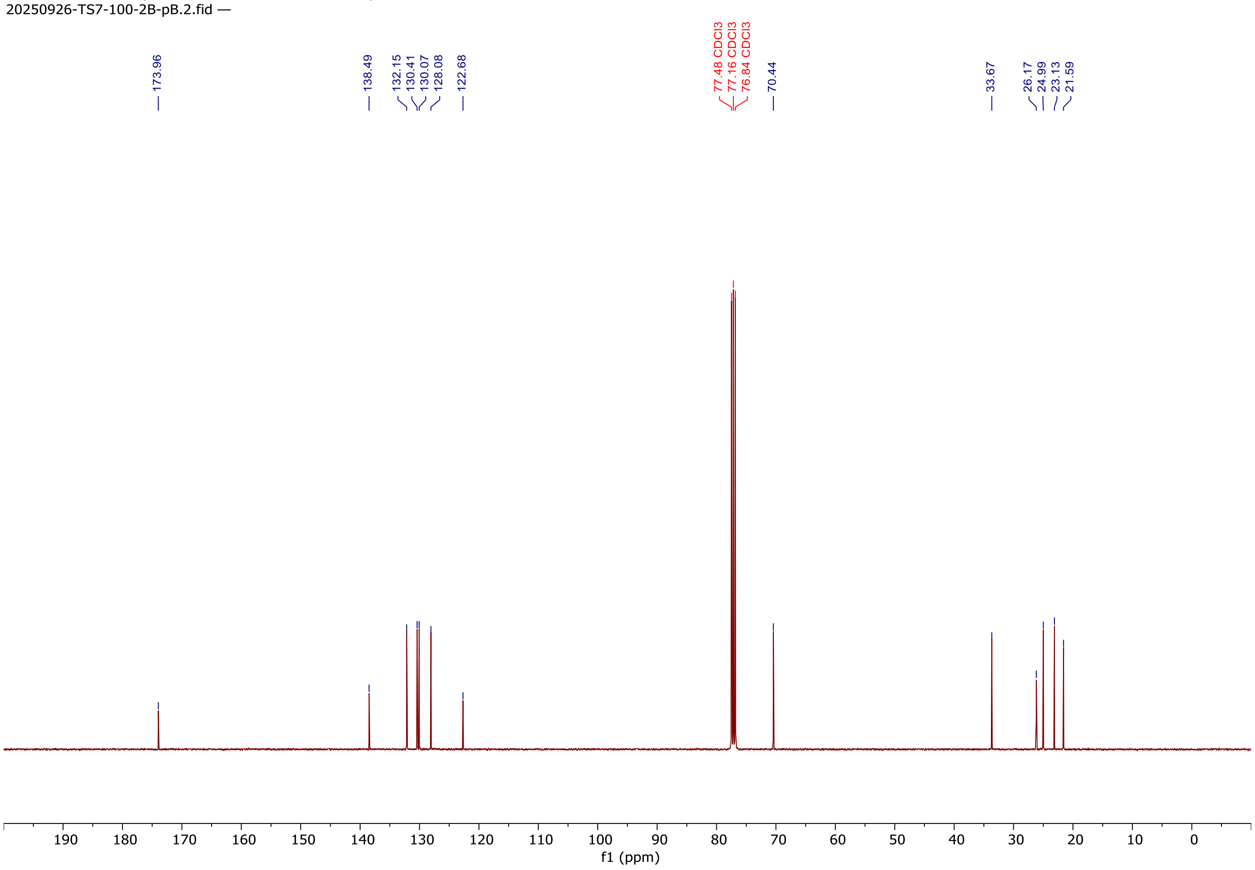

Chiral HPLC (210 nm trace):
Racemate, synthesized from $Rh_2(OAc)_4$

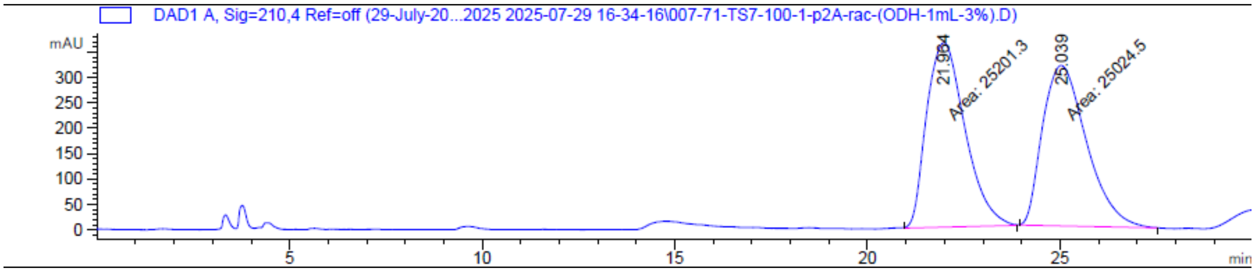

Signal 1: DAD1 A, Sig=210,4 Ref=off

Peak #	RetTime [min]	Type	Width [min]	Area [mAU*s]	Height [mAU]	Area %
1	21.964	MM	1.1561	2.52013e4	363.29318	50.1760
2	25.039	MM	1.3205	2.50245e4	315.84393	49.8240
Totals :				5.02258e4	679.13712	

Chiral, synthesized from Rh_2(*S*-*p*-PhTPCP)$_4$, 94% *ee*

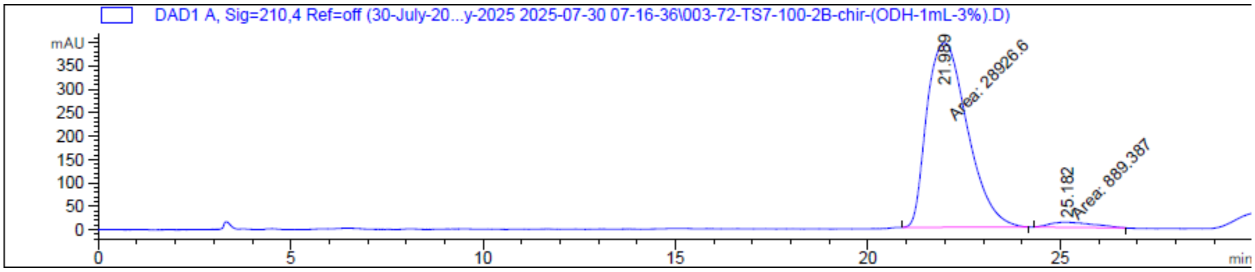

Signal 1: DAD1 A, Sig=210,4 Ref=off

Peak #	RetTime [min]	Type	Width [min]	Area [mAU*s]	Height [mAU]	Area %
1	21.989	MM	1.2215	2.89266e4	394.67584	97.0171
2	25.182	MM	1.3261	889.38654	11.17823	2.9829
Totals :				2.98160e4	405.85407	

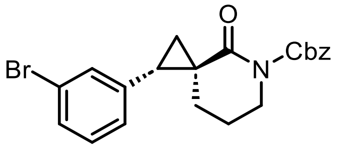

Benzyl (1*S*,3*R*)-1-(3-bromophenyl)-4-oxo-5-azaspiro[2.5]octane-5-carboxylate (17b)

^{1}H NMR (400 MHz, $CDCl_3$)

20251006-TS7-109-1B-pA.1.fid —

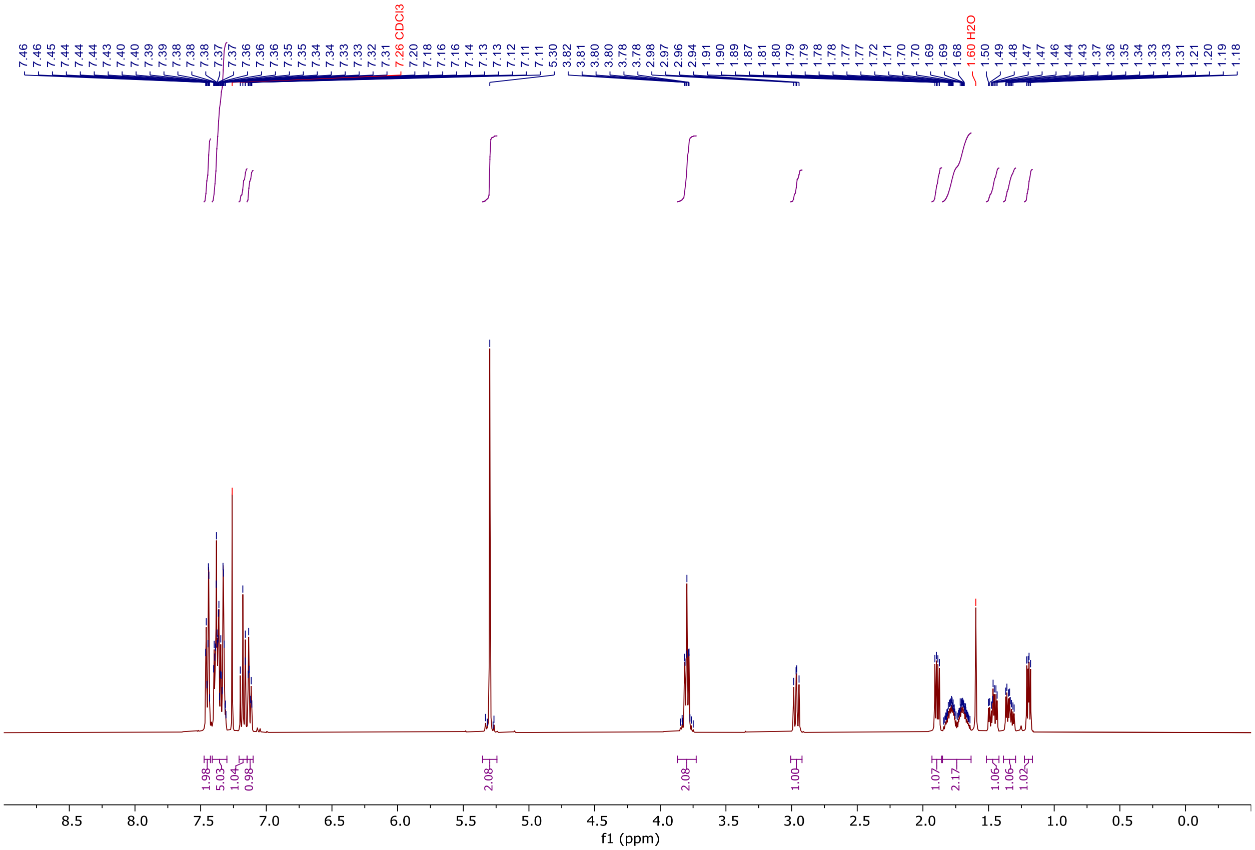

^{13}C NMR (101 MHz, $CDCl_3$)

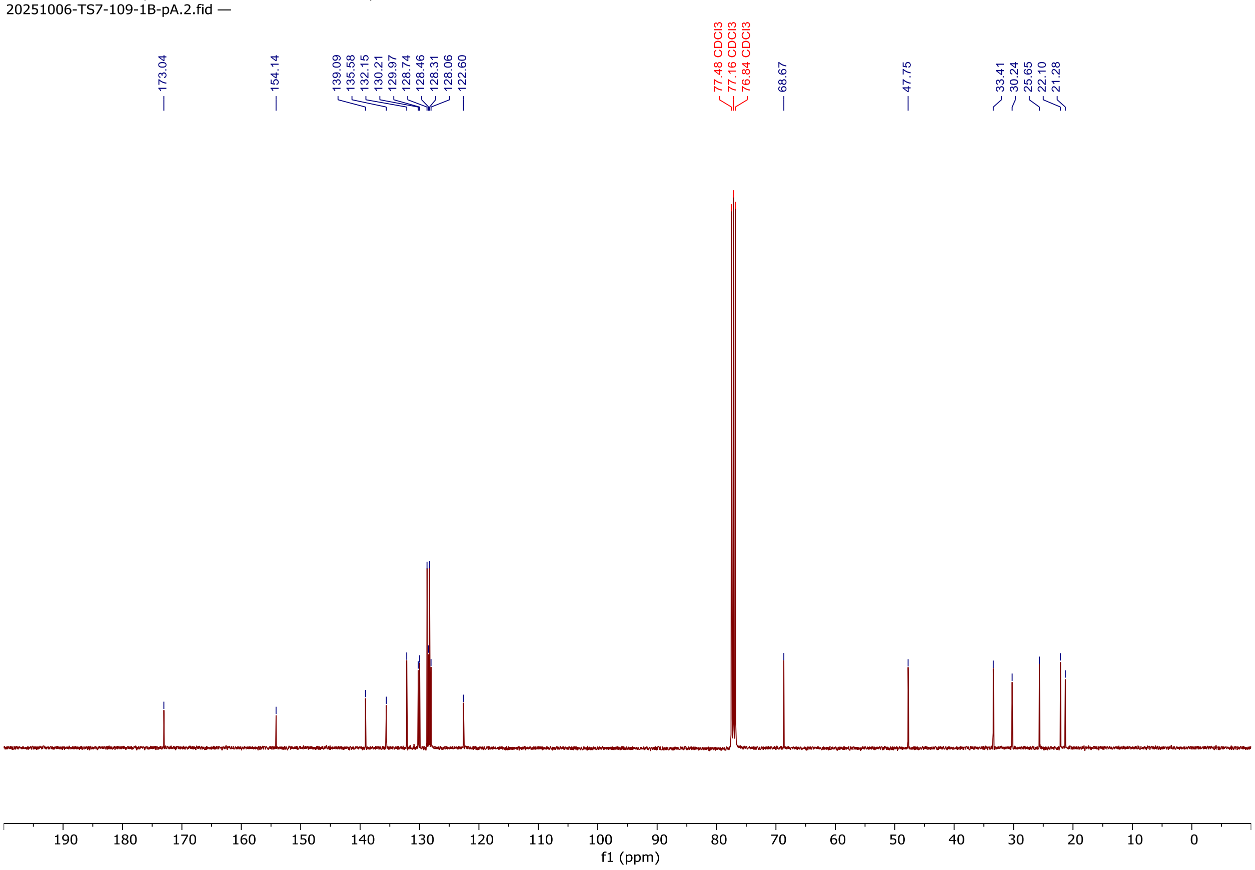

Chiral HPLC (210 nm trace):
Racemate, synthesized from $Rh_2(OAc)_4$

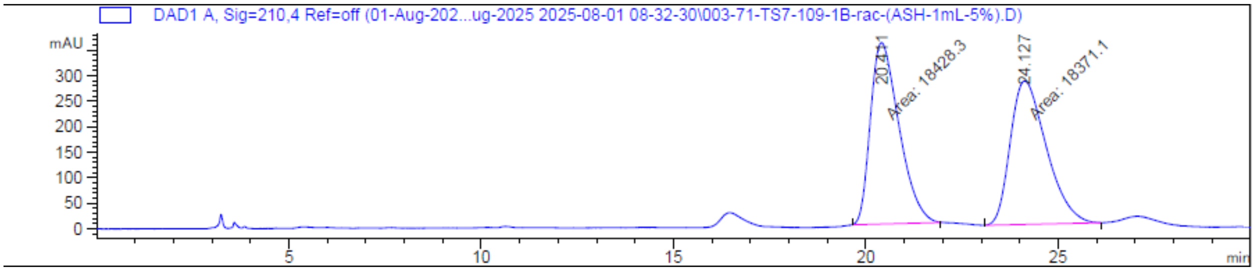

Signal 1: DAD1 A, Sig=210,4 Ref=off

Peak #	RetTime [min]	Type	Width [min]	Area [mAU*s]	Height [mAU]	Area %
1	20.411	MM	0.8624	1.84283e4	356.14673	50.0777
2	24.127	MM	1.0873	1.83711e4	281.61328	49.9223
Totals :				3.67995e4	637.76001	

Chiral, synthesized from Rh_2(*S*-*p*-PhTPCP)$_4$, 98% *ee*

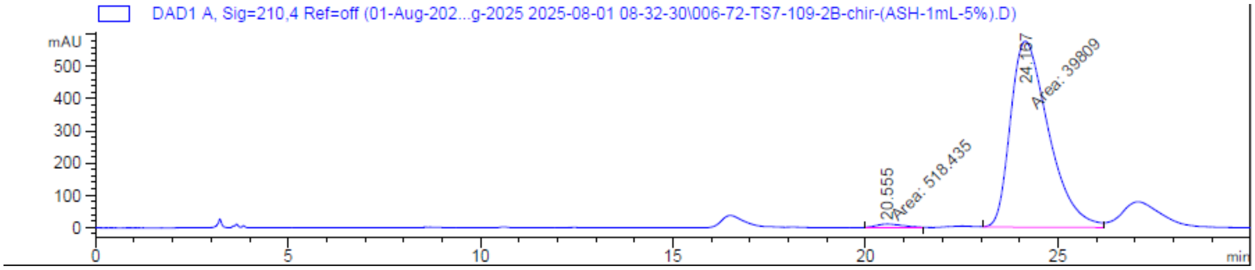

Signal 1: DAD1 A, Sig=210,4 Ref=off

Peak #	RetTime [min]	Type	Width [min]	Area [mAU*s]	Height [mAU]	Area %
1	20.555	MM	0.7909	518.43500	10.92500	1.2856
2	24.167	MM	1.1477	3.98090e4	578.11218	98.7144
Totals :				4.03274e4	589.03718	

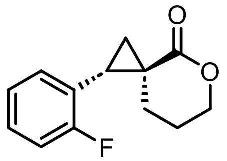

(1*S*,3*R*)-1-(2-Fluorophenyl)-5-oxaspiro[2.5]octan-4-one (18a)

^{1}H NMR (400 MHz, $CDCl_3$)

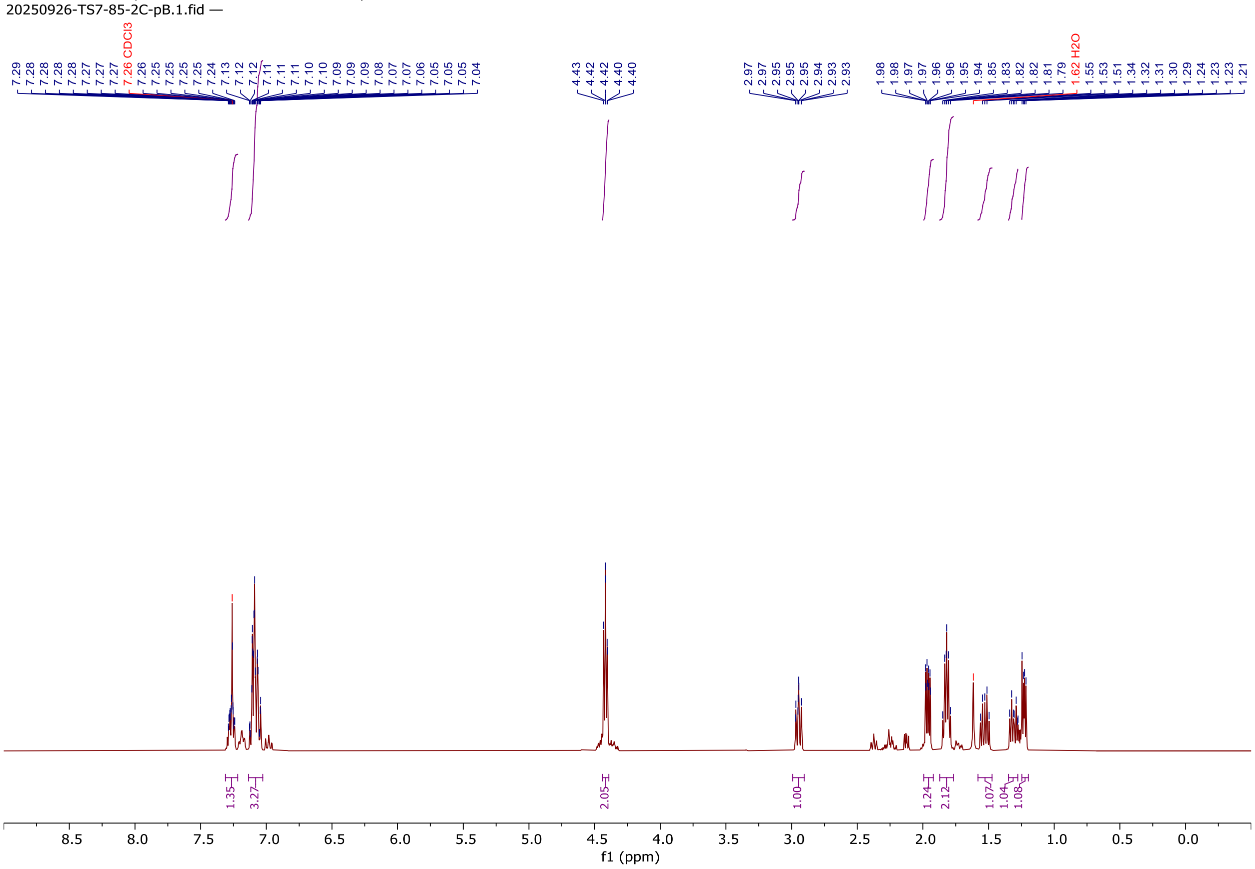

^{19}F NMR (376 MHz, $CDCl_3$)

20250926-TS7-85-2C-pB.2.fid —

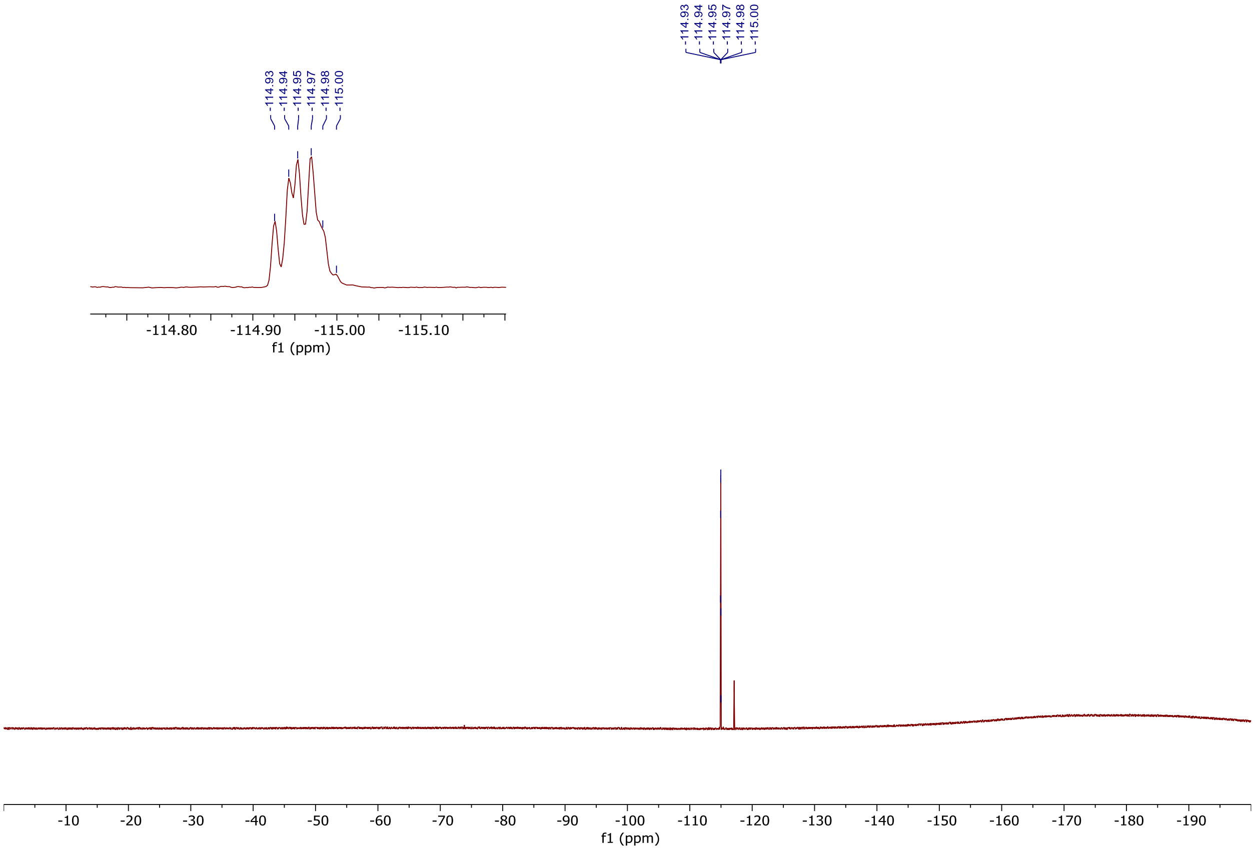

^{13}C NMR (101 MHz, $CDCl_3$)

20250926-TS7-85-2C-pB.3.fid —

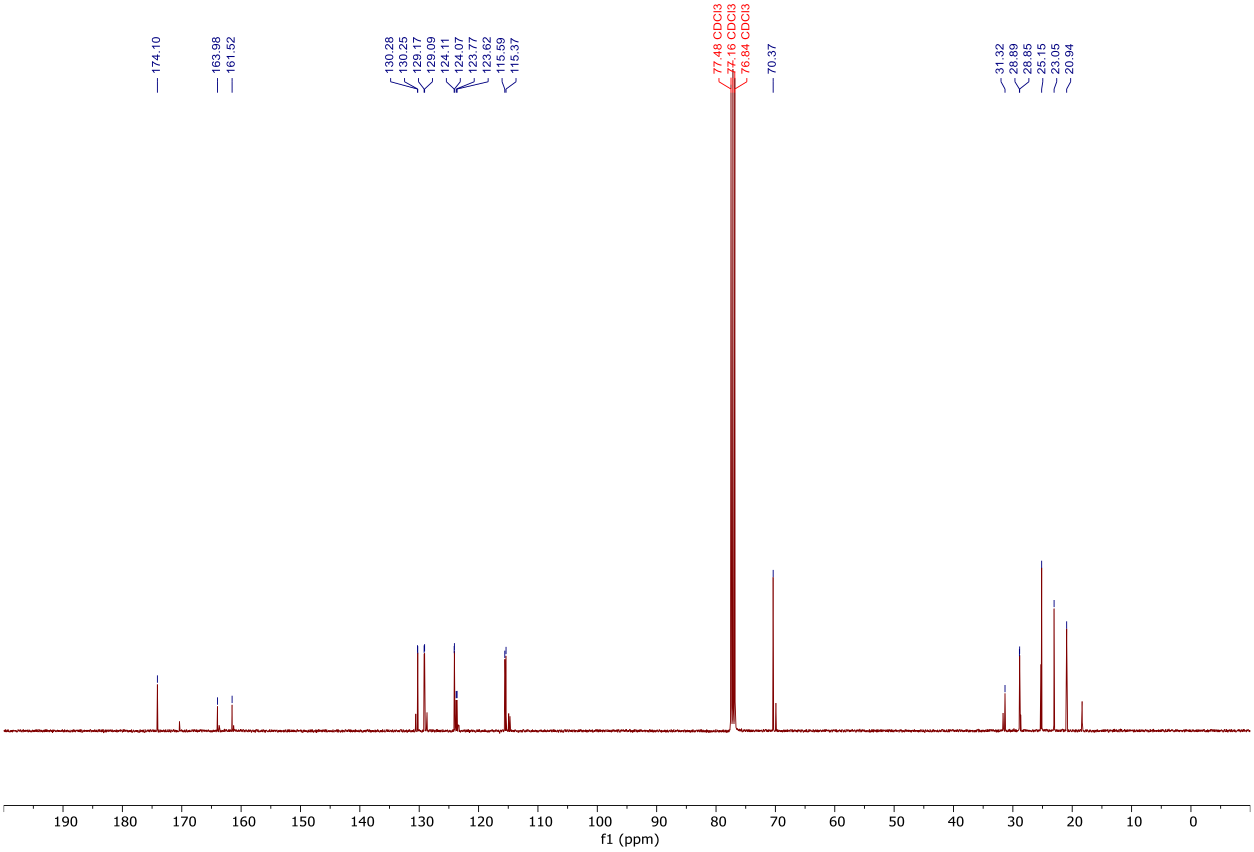

Chiral HPLC (210 nm trace):
Racemate, synthesized from $Rh_2(OAc)_4$

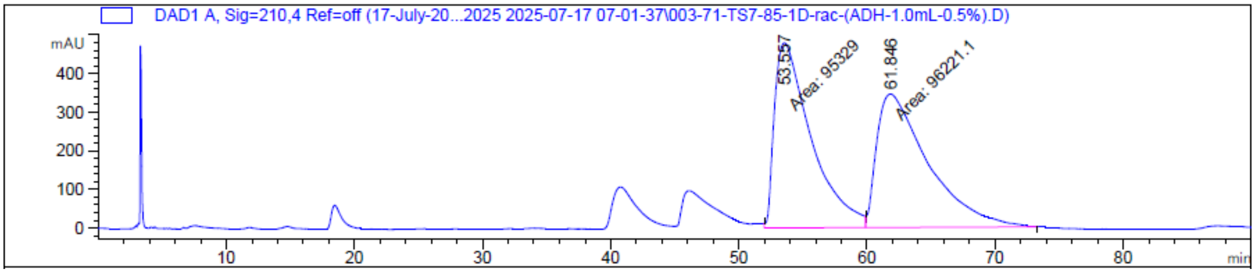

Signal 1: DAD1 A, Sig=210,4 Ref=off

Peak #	RetTime [min]	Type	Width [min]	Area [mAU*s]	Height [mAU]	Area %
1	53.557	MF	3.3220	9.53290e4	478.26425	49.7672
2	61.846	FM	4.6444	9.62211e4	345.29333	50.2328
Totals :				1.91550e5	823.55759	

Chiral, synthesized from Rh_2(*S*-*p*-PhTPCP)$_4$, 93% *ee*

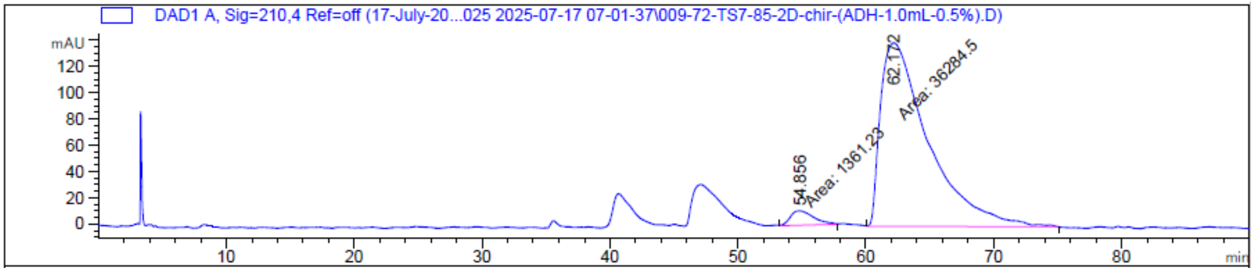

Signal 1: DAD1 A, Sig=210,4 Ref=off

Peak #	RetTime [min]	Type	Width [min]	Area [mAU*s]	Height [mAU]	Area %
1	54.856	MM	2.0726	1361.23035	10.94608	3.6159
2	62.172	MM	4.3312	3.62845e4	139.62558	96.3841
Totals :				3.76457e4	150.57166	

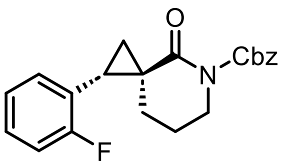

Benzyl (1*S*,3*R*)-1-(2-fluorophenyl)-4-oxo-5-azaspiro[2.5]octane-5-carboxylate (18b)

^{1}H NMR (400 MHz, $CDCl_3$)

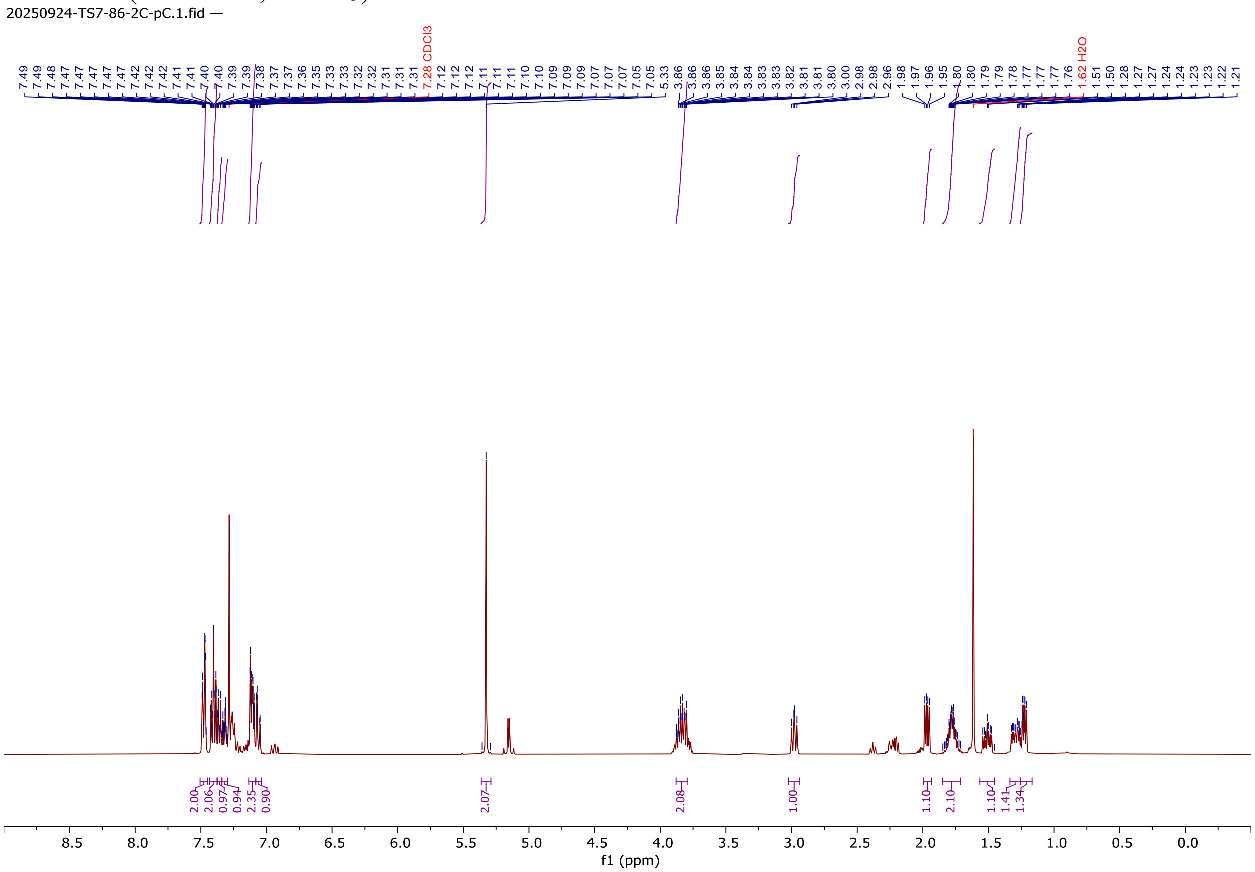

^{19}F NMR (376 MHz, $CDCl_3$)

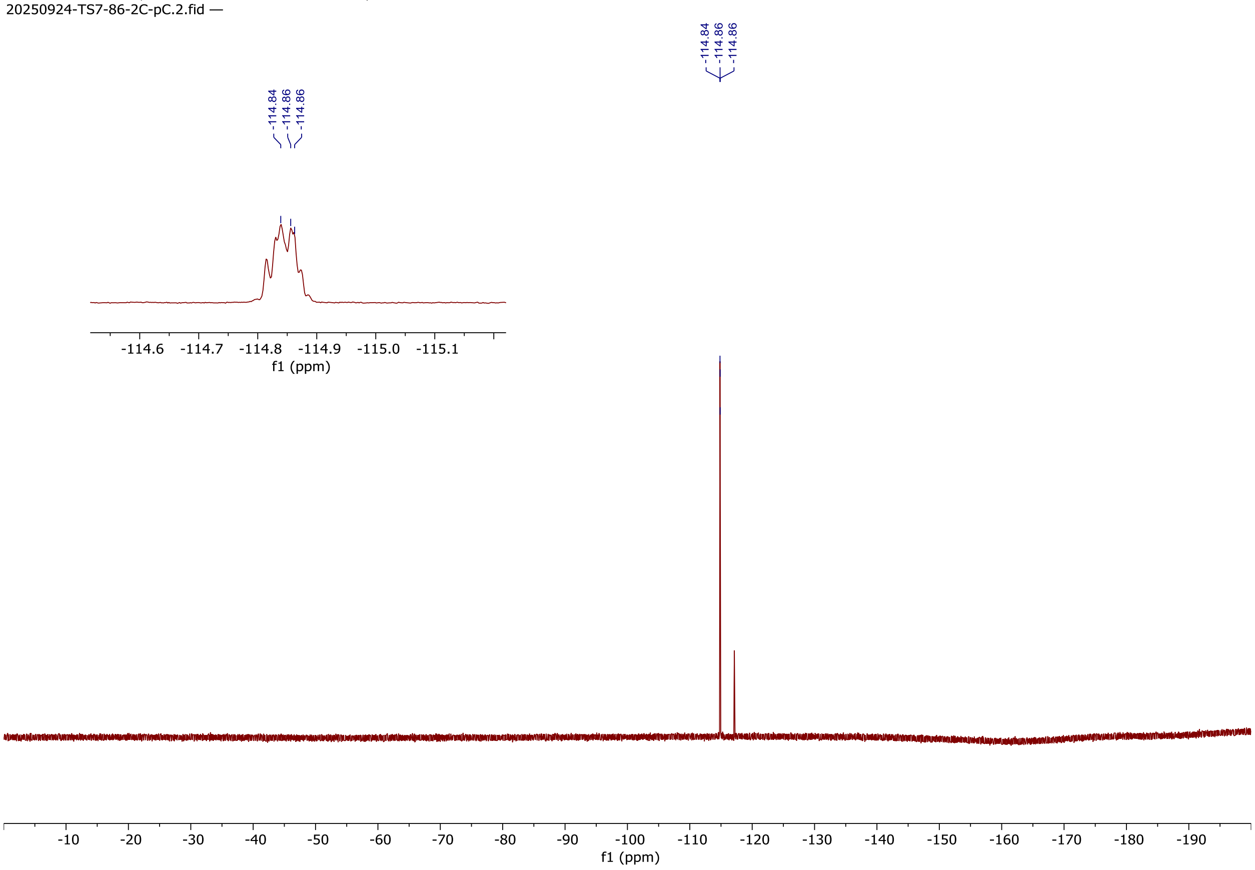

^{13}C NMR (101 MHz, $CDCl_3$)

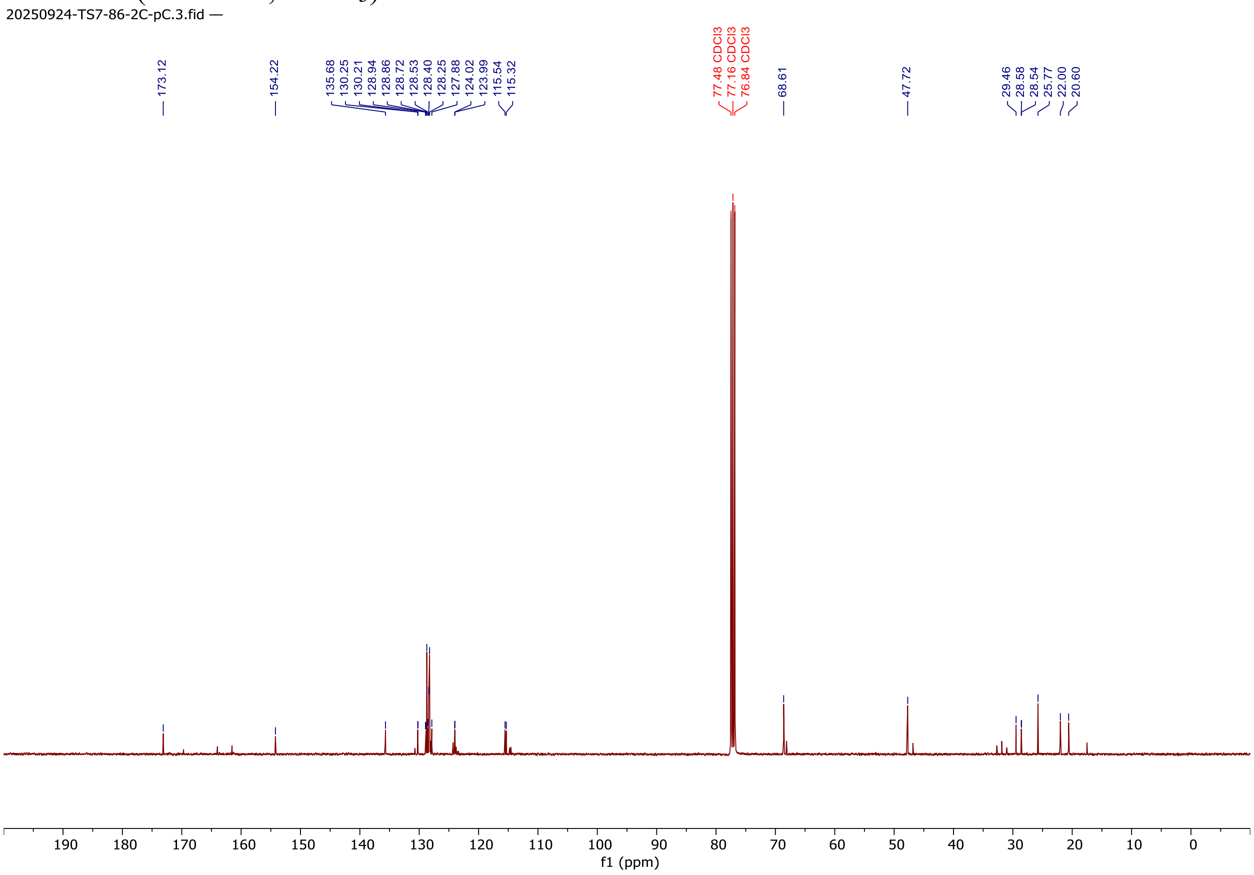

Chiral HPLC (210 nm trace):
Racemate, synthesized from $Rh_2(OAc)_4$

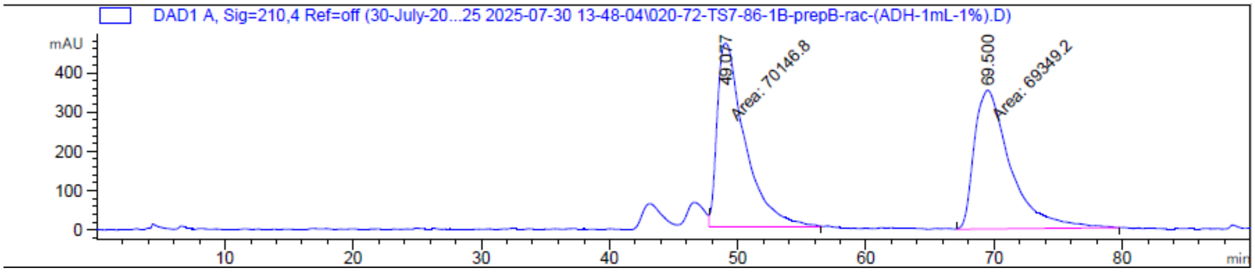


```
Signal 1: DAD1 A, Sig=210,4 Ref=off

Peak RetTime Type  Width     Area       Height     Area
  #   [min]        [min]   [mAU*s]     [mAU]        %
----|-------|----|-------|----------|----------|--------|
   1  49.077 MM    2.5048 7.01468e4  466.74176  50.2859
   2  69.500 MM    3.2764 6.93492e4  352.77625  49.7141

Totals :                  1.39496e5  819.51801
```

Chiral, synthesized from Rh_2(*S*-*p*-PhTPCP)$_4$, 93% *ee*

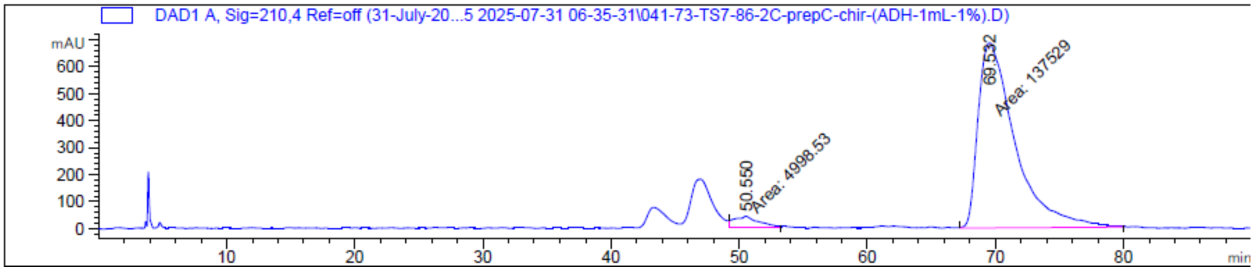


```
Signal 1: DAD1 A, Sig=210,4 Ref=off

Peak RetTime Type  Width     Area       Height     Area
  #   [min]        [min]   [mAU*s]     [mAU]        %
----|-------|----|-------|----------|----------|--------|
   1  50.550 MM    2.1363 4998.52539   38.99646   3.5071
   2  69.532 MM    3.3396 1.37528e5  686.34528  96.4929

Totals :                  1.42527e5  725.34174
```

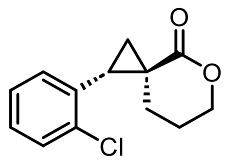

(1*S*,3*R*)-1-(2-Chlorophenyl)-5-oxaspiro[2.5]octan-4-one (19a)

^{1}H NMR (400 MHz, $CDCl_3$)

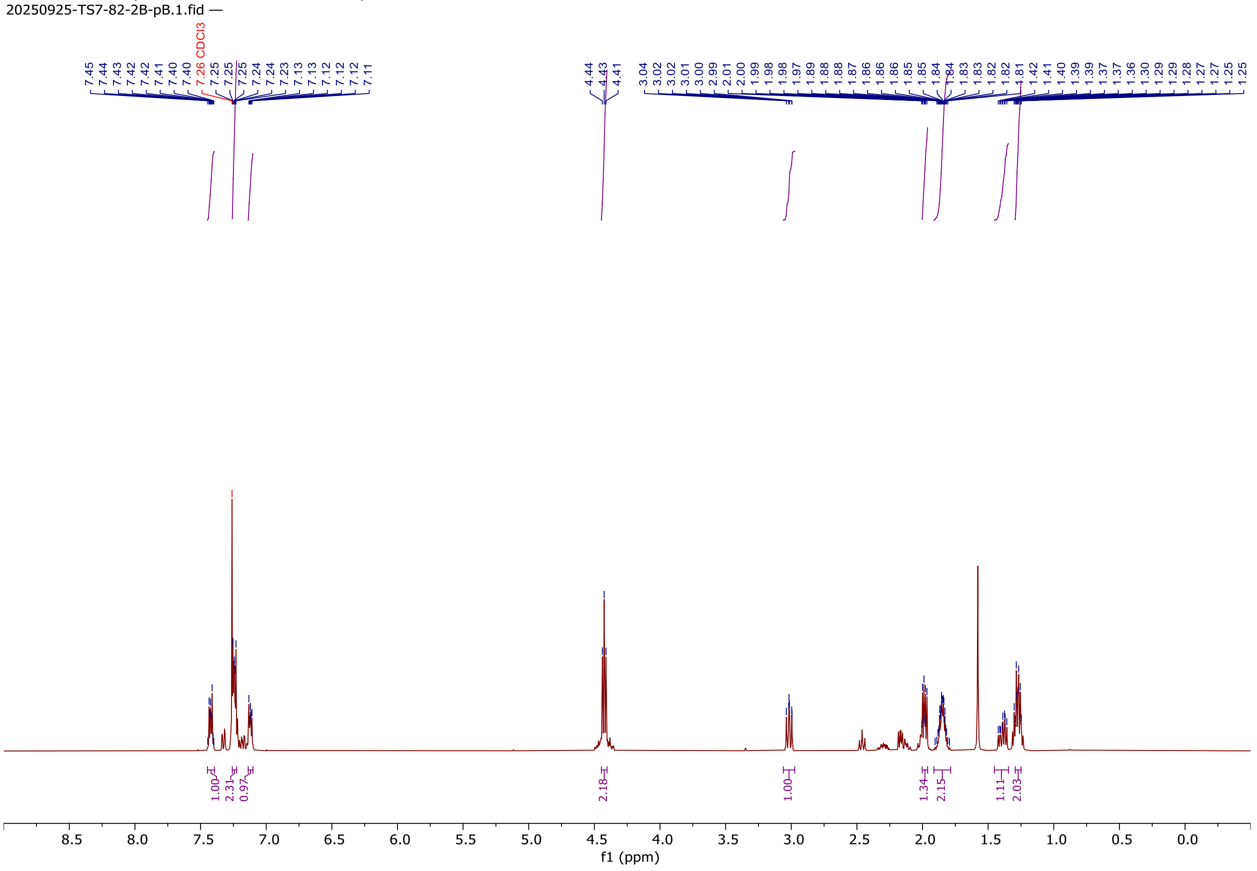

^{13}C NMR (101 MHz, $CDCl_3$)

20250925-TS7-82-2B-pB.2.fid —

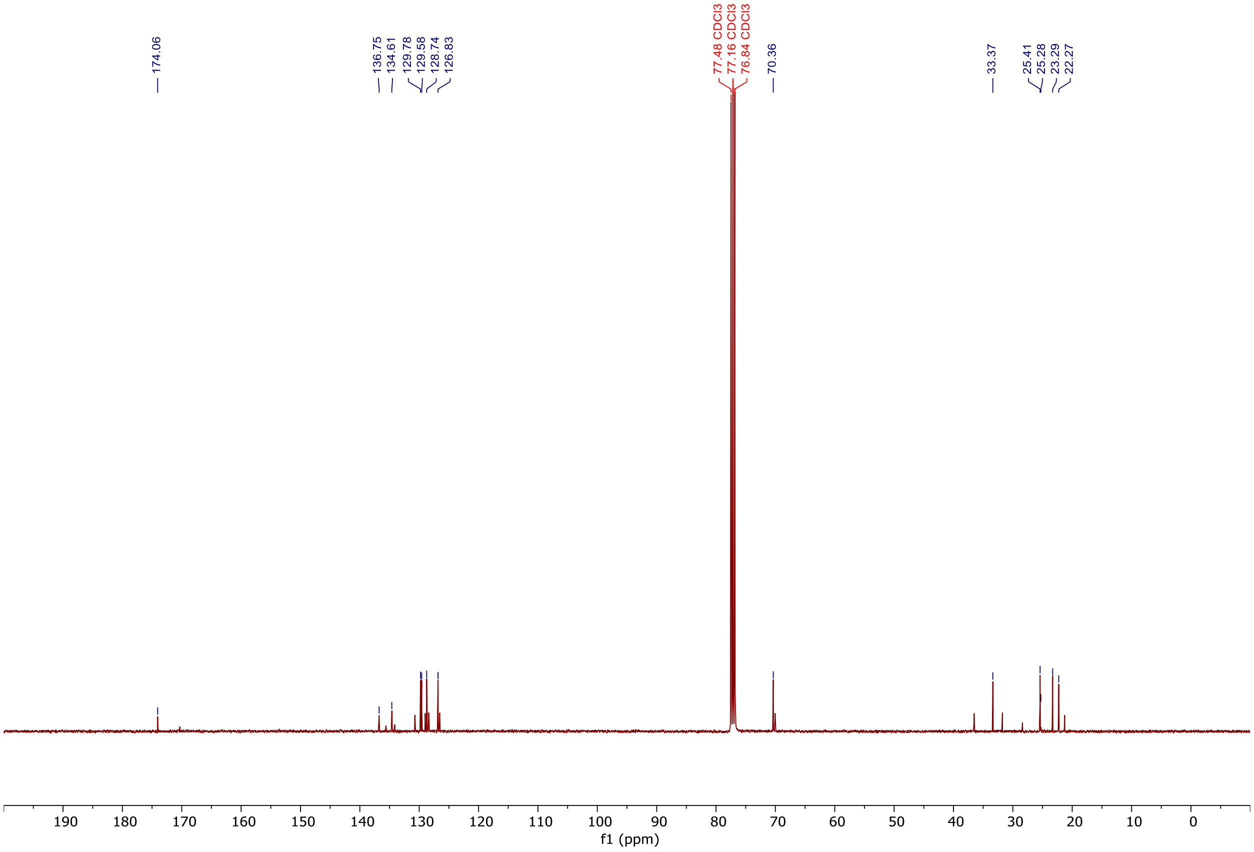

Chiral HPLC (210 nm trace):
Racemate, synthesized from $Rh_2(OAc)_4$

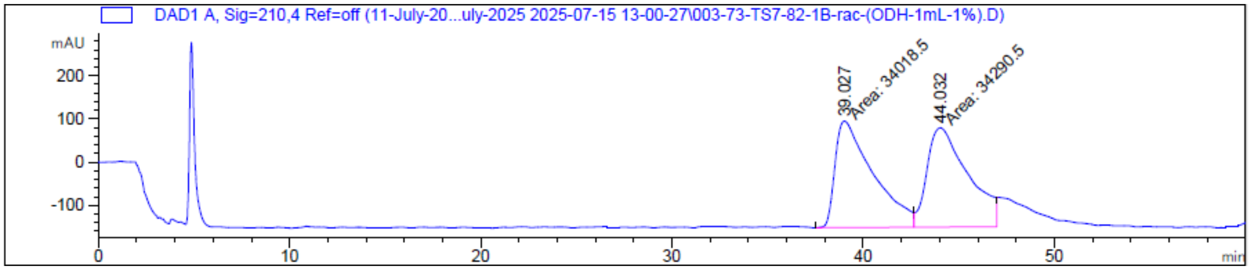

Signal 1: DAD1 A, Sig=210,4 Ref=off

Peak #	RetTime [min]	Type	Width [min]	Area [mAU*s]	Height [mAU]	Area %
1	39.027	MF	2.3027	3.40185e4	246.21761	49.8009
2	44.032	MF	2.4914	3.42905e4	229.39037	50.1991

Totals : 6.83091e4 475.60797

Chiral, synthesized from Rh_2(*S*-*p*-PhTPCP)$_4$, 88% *ee*

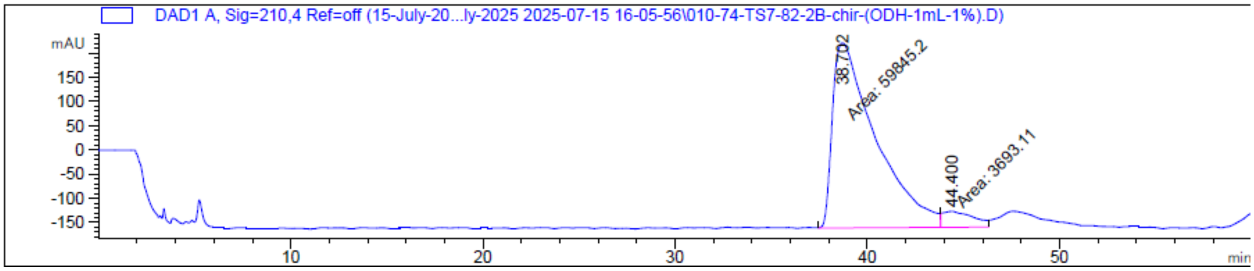

Signal 1: DAD1 A, Sig=210,4 Ref=off

Peak #	RetTime [min]	Type	Width [min]	Area [mAU*s]	Height [mAU]	Area %
1	38.702	MF	2.6102	5.98452e4	382.13000	94.1876
2	44.400	MF	1.8661	3693.11108	32.98363	5.8124

Totals : 6.35383e4 415.11363

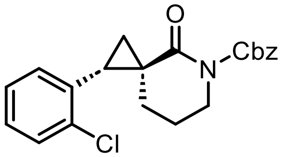

Benzyl (1*S*,3*R*)-1-(2-chlorophenyl)-4-oxo-5-azaspiro[2.5]octane-5-carboxylate (19b)

^{1}H NMR (400 MHz, $CDCl_3$)

20250923-TS7-81-1C.1.fid —

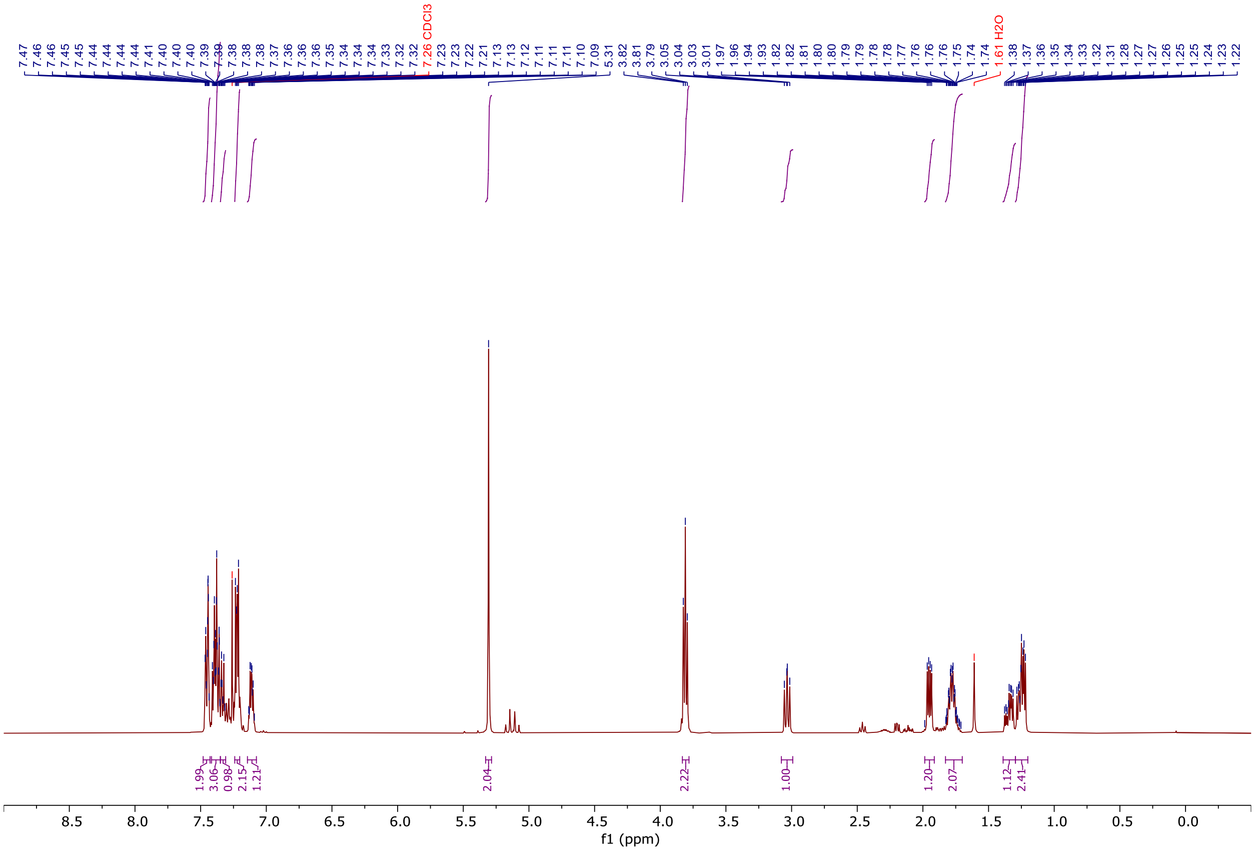

^{13}C NMR (101 MHz, $CDCl_3$)

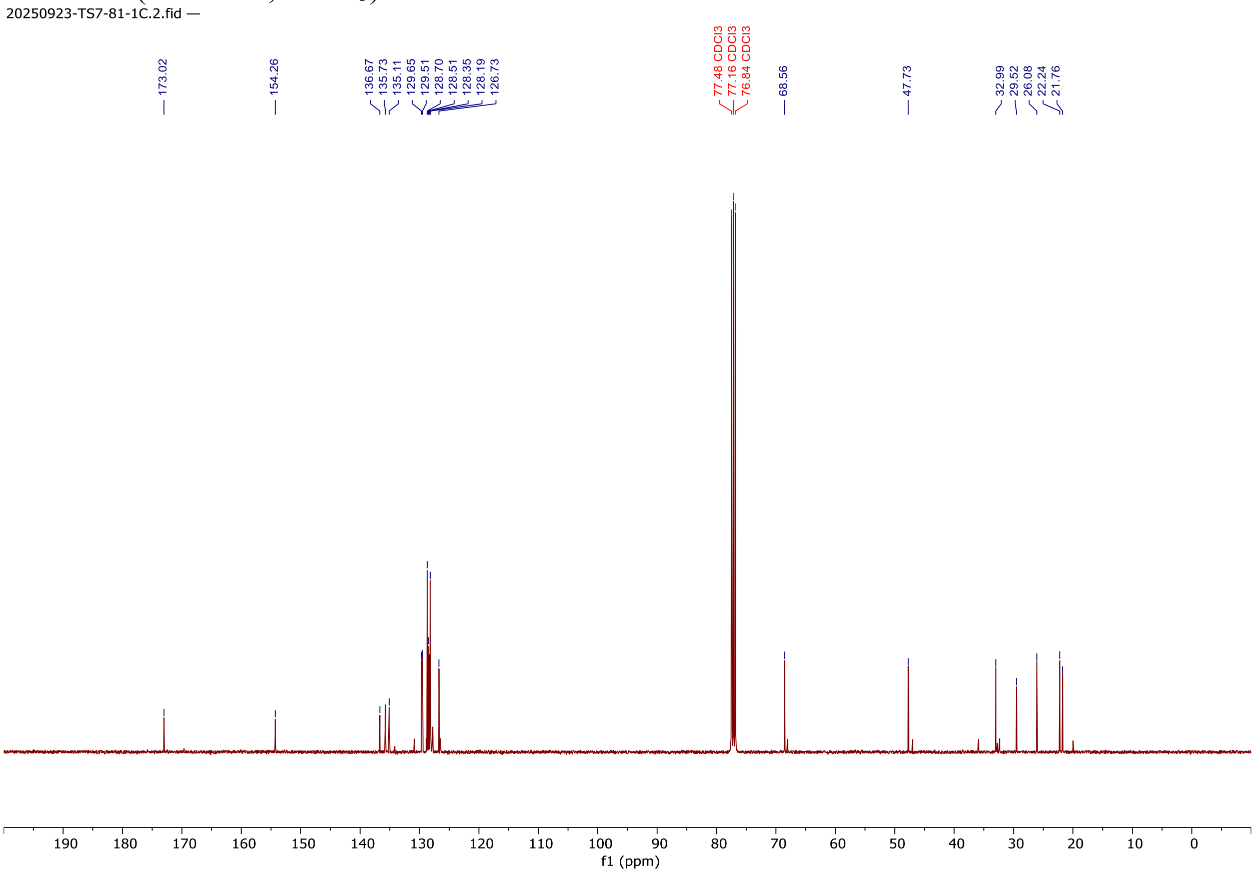

Chiral HPLC (210 nm trace):
Racemate, synthesized from $Rh_2(OAc)_4$

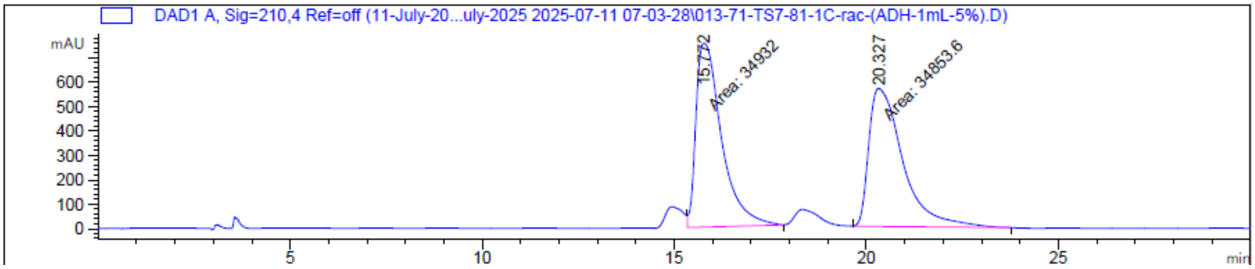

Signal 1: DAD1 A, Sig=210,4 Ref=off

Peak #	RetTime [min]	Type	Width [min]	Area [mAU*s]	Height [mAU]	Area %
1	15.772	FM	0.7719	3.49320e4	754.26135	50.0561
2	20.327	MM	1.0262	3.48536e4	566.04523	49.9439

Totals : 6.97855e4 1320.30658

Chiral, synthesized from Rh_2(*S*-*p*-PhTPCP)$_4$, 96% *ee*

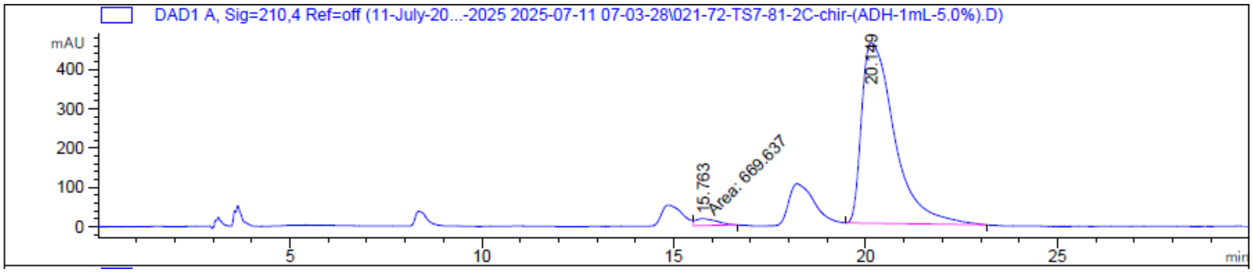

Signal 1: DAD1 A, Sig=210,4 Ref=off

Peak #	RetTime [min]	Type	Width [min]	Area [mAU*s]	Height [mAU]	Area %
1	15.763	FM	0.6294	669.63708	17.73337	2.3841
2	20.149	VV R	0.6990	2.74183e4	459.75781	97.6159

Totals : 2.80879e4 477.49118

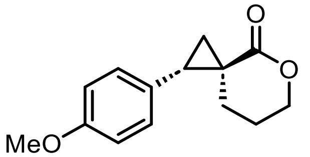

(1*S*,3*R*)-1-(4-Methoxyphenyl)-5-oxaspiro[2.5]octan-4-one (20a)

^{1}H NMR (400 MHz, $CDCl_3$)

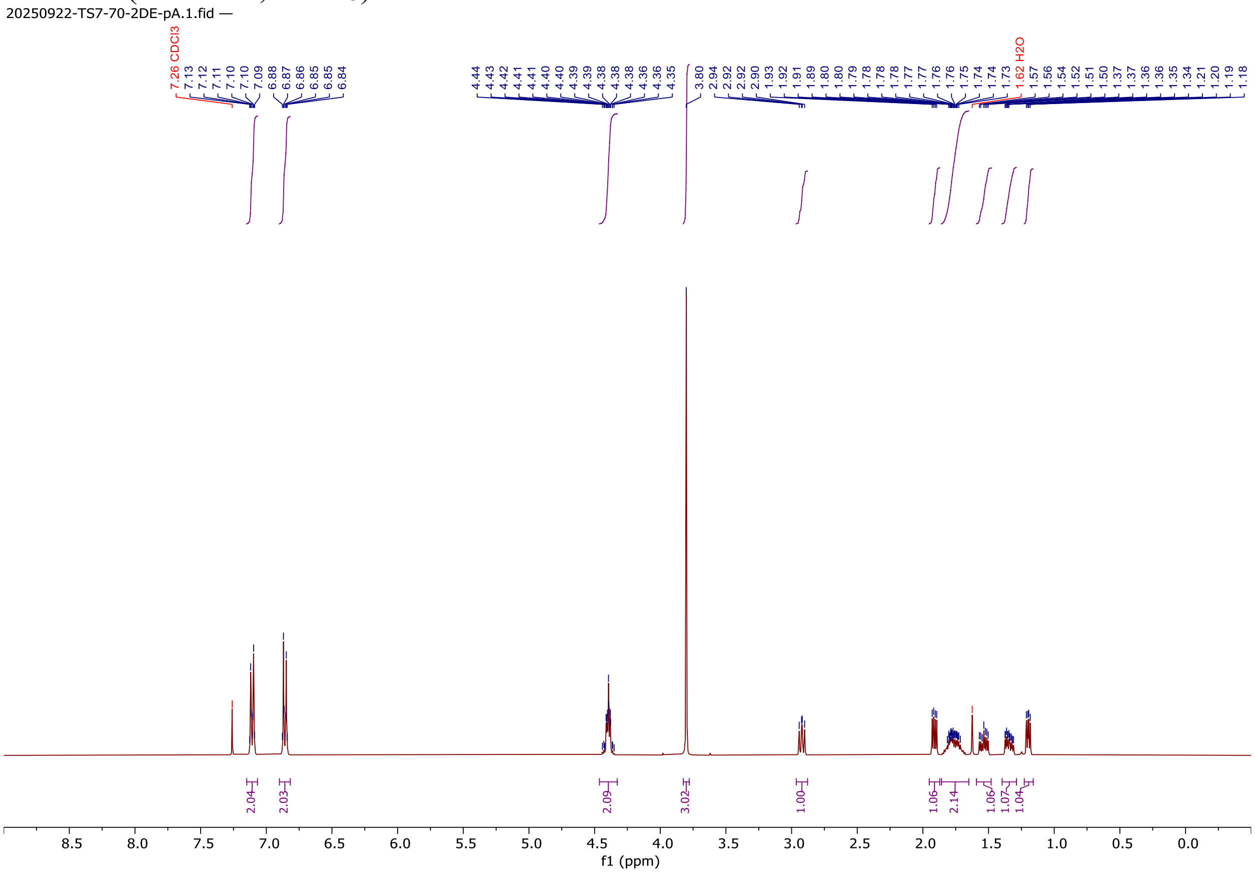

^{13}C NMR (101 MHz, $CDCl_3$)

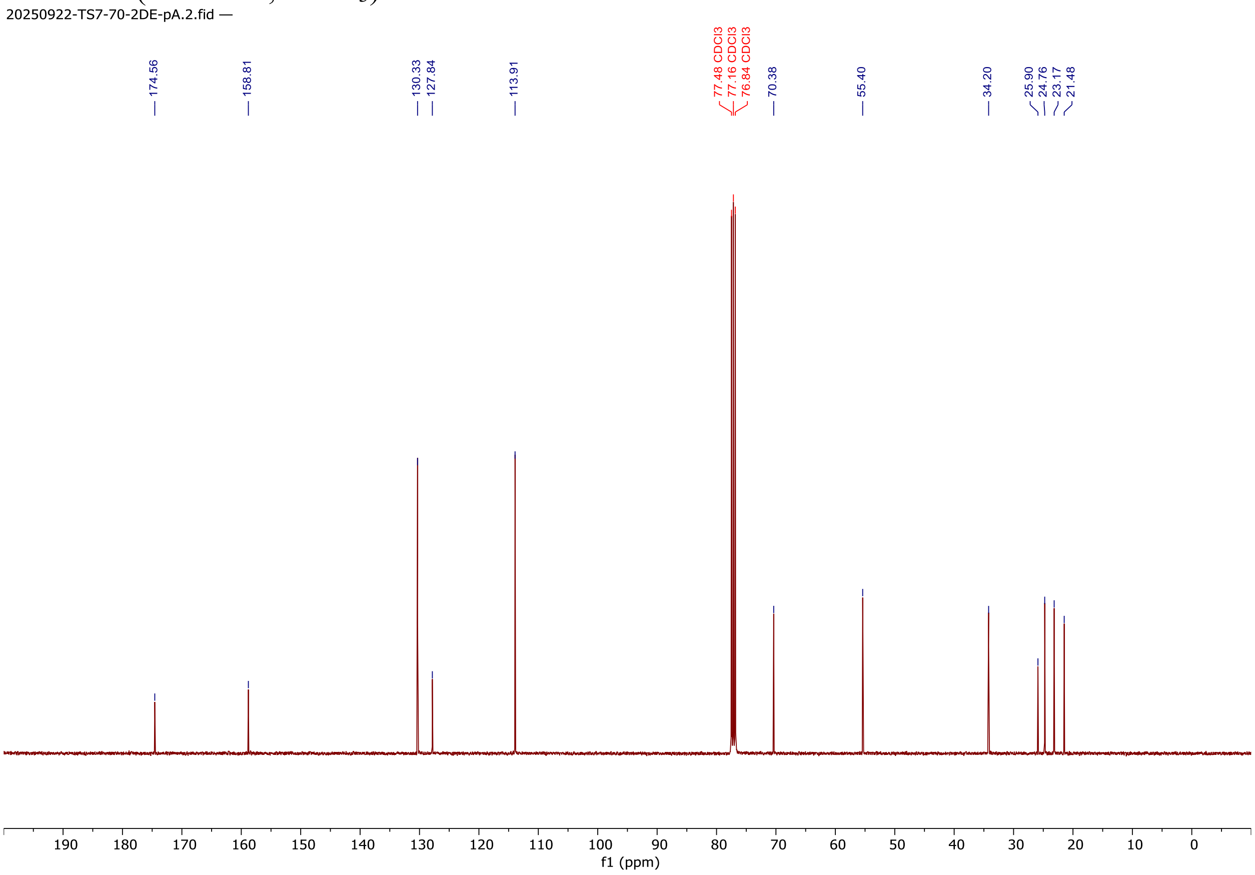

Chiral HPLC (210 nm trace):
Racemate, synthesized from $Rh_2(OAc)_4$

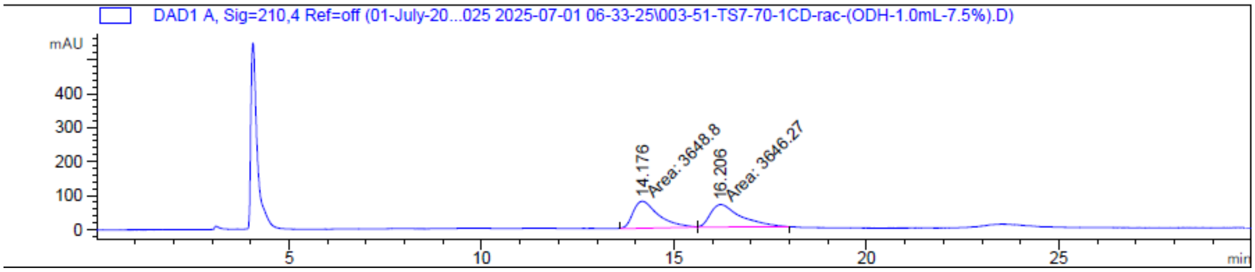

Signal 1: DAD1 A, Sig=210,4 Ref=off

Peak #	RetTime [min]	Type	Width [min]	Area [mAU*s]	Height [mAU]	Area %
1	14.176	MM	0.7647	3648.79565	79.52149	50.0173
2	16.206	MM	0.9199	3646.26929	66.06460	49.9827
Totals :				7295.06494	145.58609	

Chiral, synthesized from $Rh_2(S\text{-}p\text{-}PhTPCP)_4$, 94% *ee*

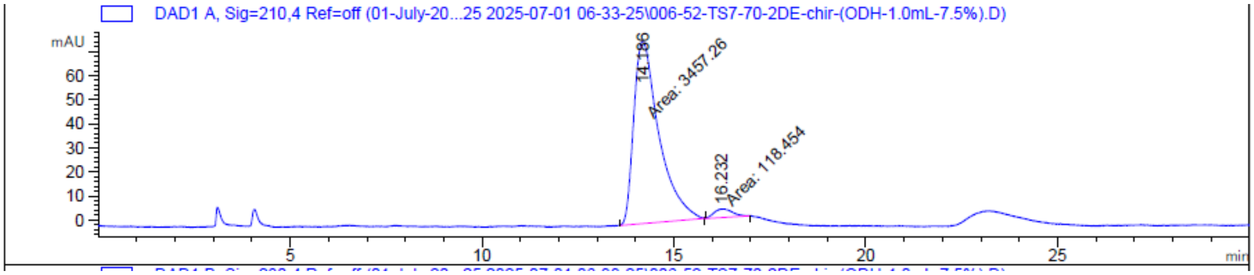

Signal 1: DAD1 A, Sig=210,4 Ref=off

Peak #	RetTime [min]	Type	Width [min]	Area [mAU*s]	Height [mAU]	Area %
1	14.186	MM	0.7617	3457.25513	75.65036	96.6873
2	16.232	MM	0.5399	118.45371	3.65657	3.3127
Totals :				3575.70884	79.30693	

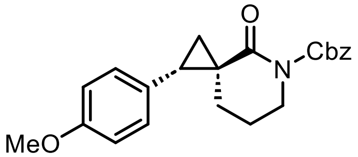

Benzyl (1*S*,3*R*)-1-(4-methoxyphenyl)-4-oxo-5-azaspiro[2.5]octane-5-carboxylate (20b)

^{1}H NMR (400 MHz, $CDCl_3$)

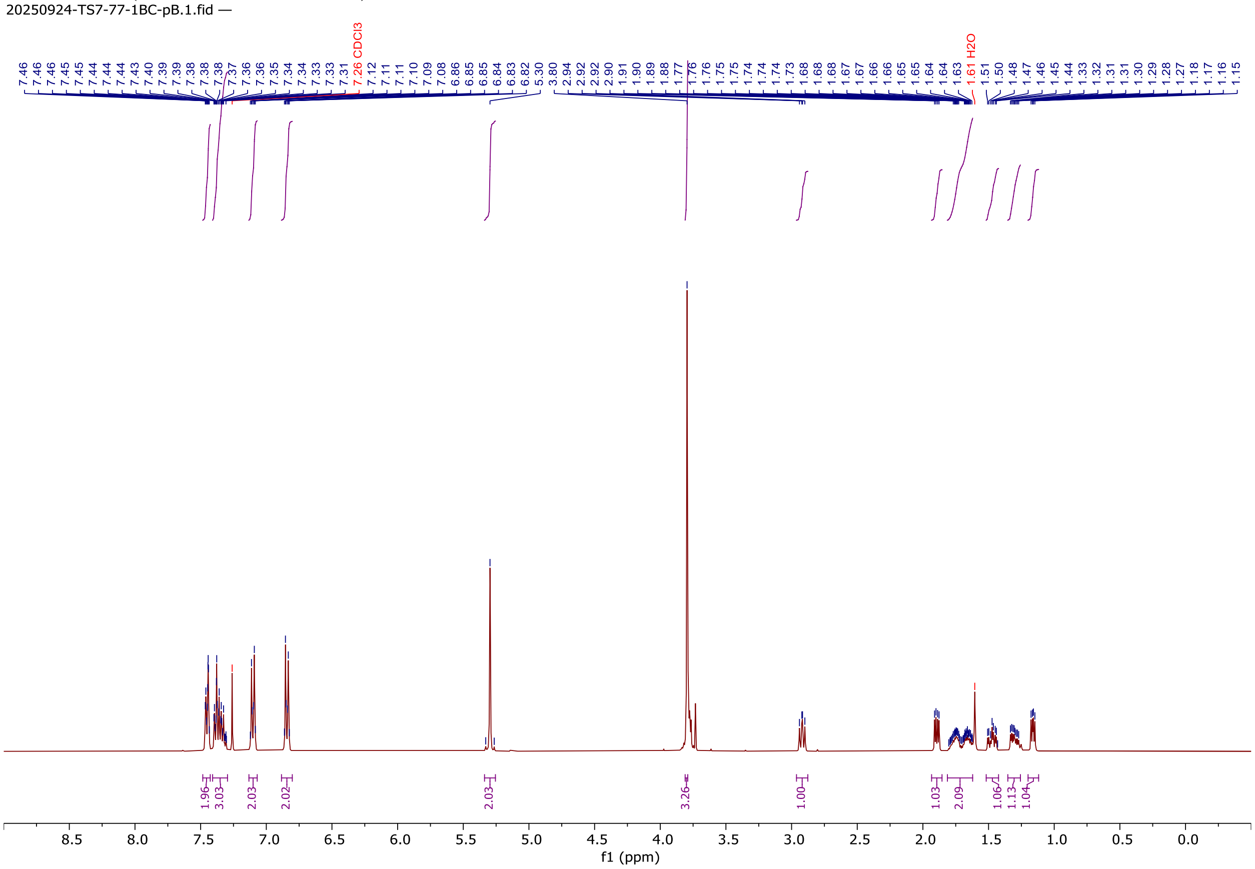

^{13}C NMR (101 MHz, $CDCl_3$)

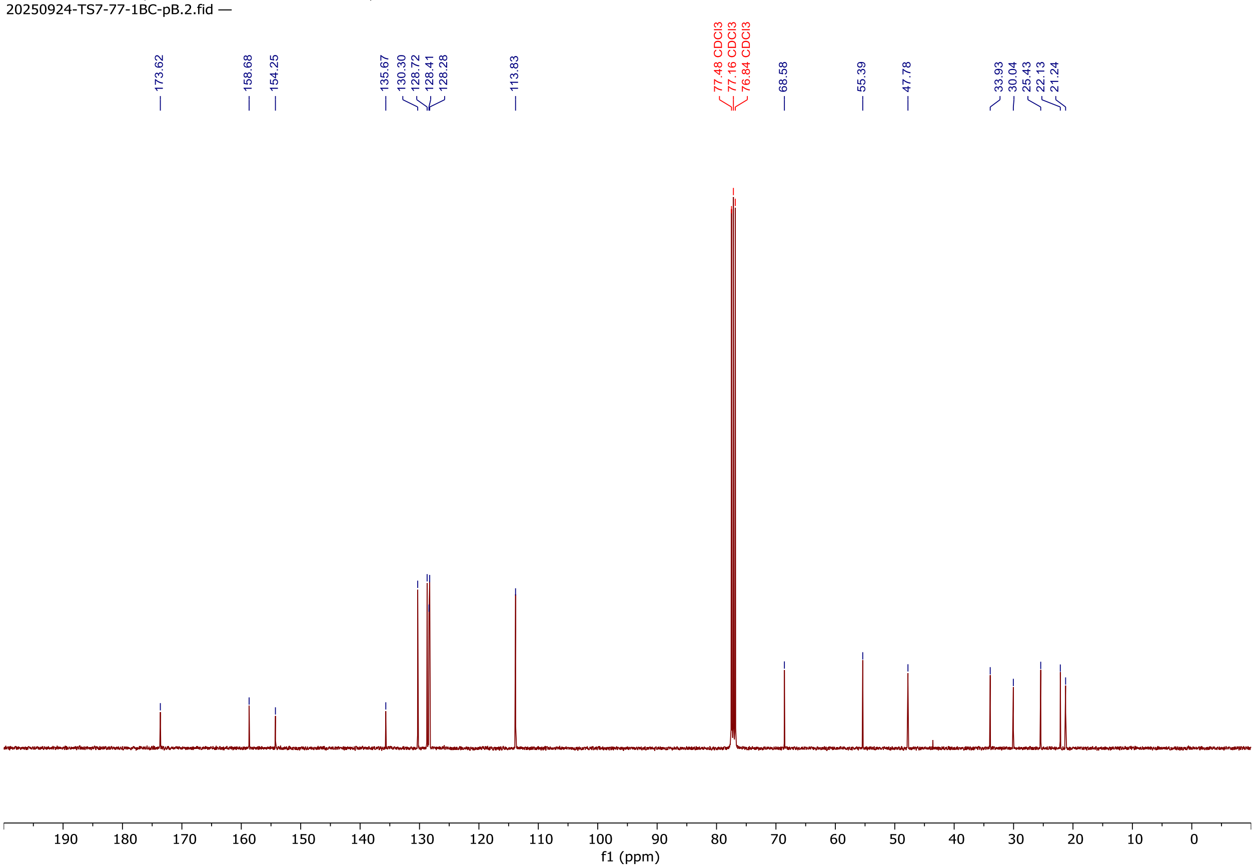

Chiral HPLC (210 nm trace):
Racemate, synthesized from $Rh_2(OAc)_4$

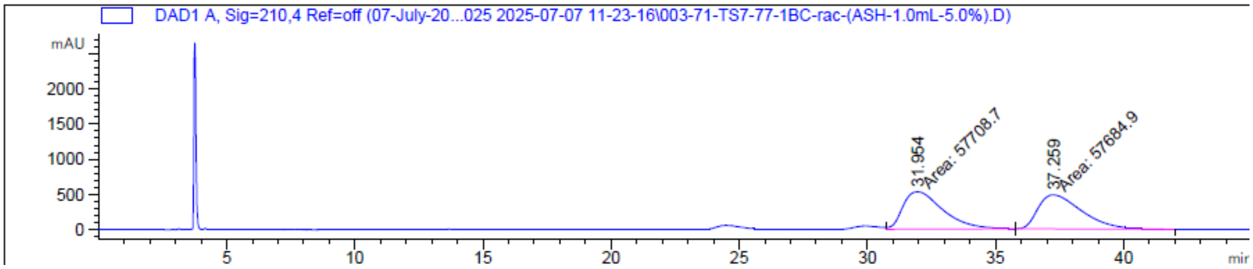

Signal 1: DAD1 A, Sig=210,4 Ref=off

Peak #	RetTime [min]	Type	Width [min]	Area [mAU*s]	Height [mAU]	Area %
1	31.954	FM	1.8157	5.77087e4	529.70636	50.0103
2	37.259	MM	1.9912	5.76849e4	482.83502	49.9897

Totals : 1.15394e5 1012.54138

Chiral, synthesized from Rh_2(*S*-*p*-PhTPCP)$_4$, 93% *ee*

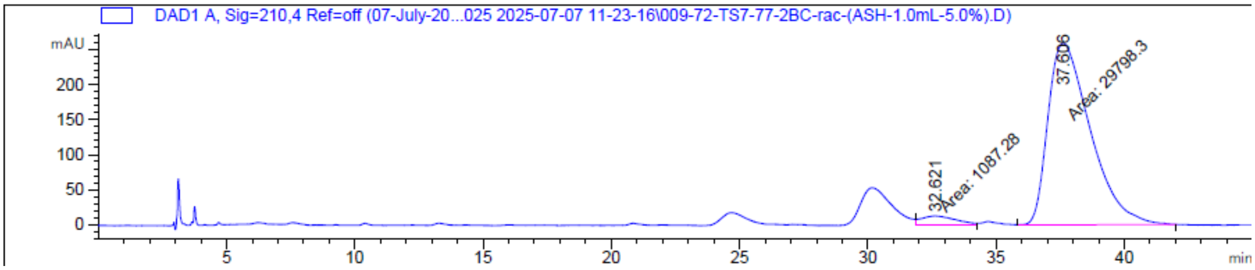

Signal 1: DAD1 A, Sig=210,4 Ref=off

Peak #	RetTime [min]	Type	Width [min]	Area [mAU*s]	Height [mAU]	Area %
1	32.621	MM	1.5073	1087.28491	12.02236	3.5204
2	37.606	MM	1.9114	2.97983e4	259.82562	96.4796

Totals : 3.08856e4 271.84798

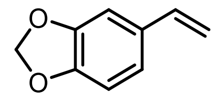

5-vinylbenzo[d][1,3]dioxole (SI-3)

^{1}H NMR (400 MHz, $CDCl_3$)

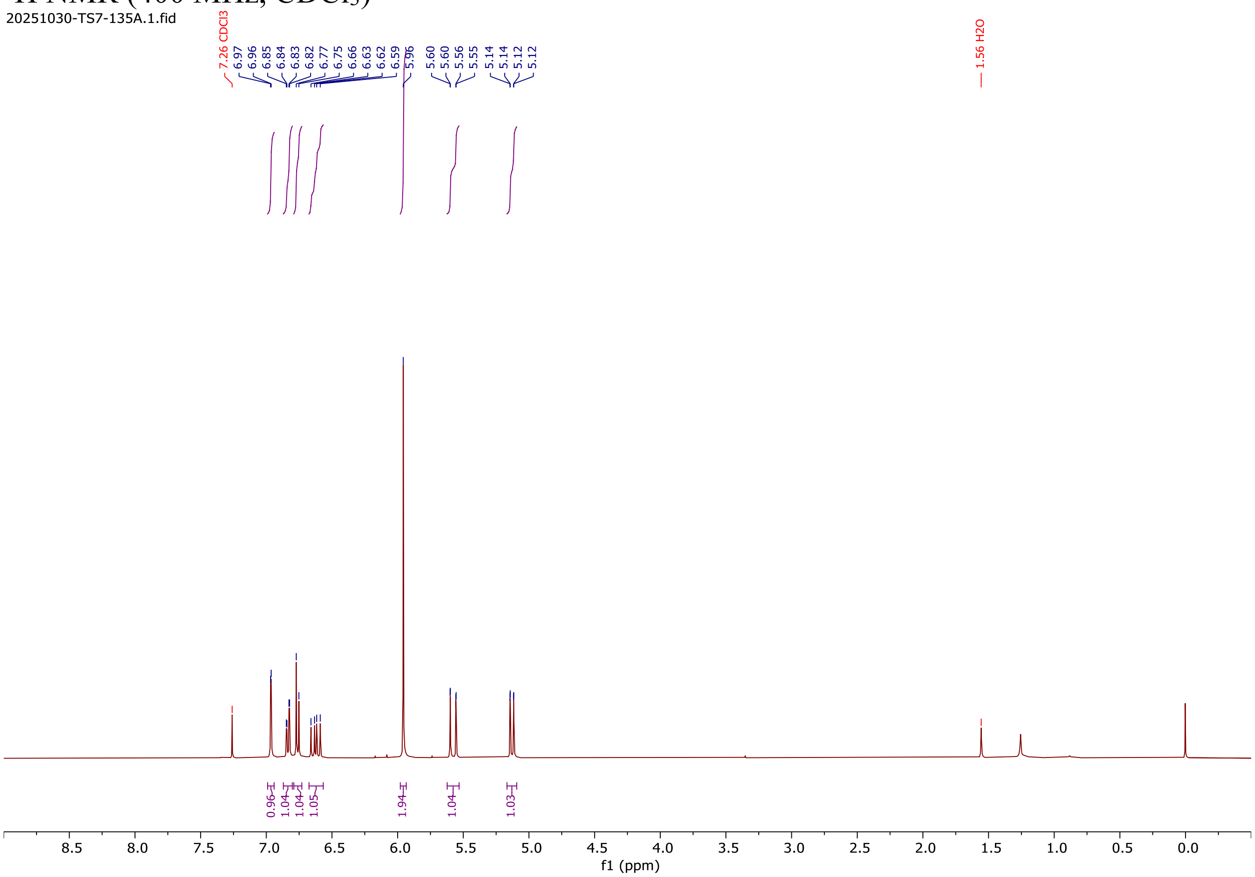

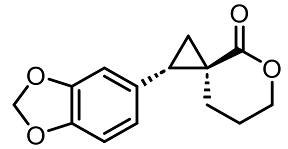

(1*S*,3*R*)-1-(Benzo[d][1,3]dioxol-5-yl)-5-oxaspiro[2.5]octan-4-one (21a)

^{1}H NMR (400 MHz, $CDCl_3$)

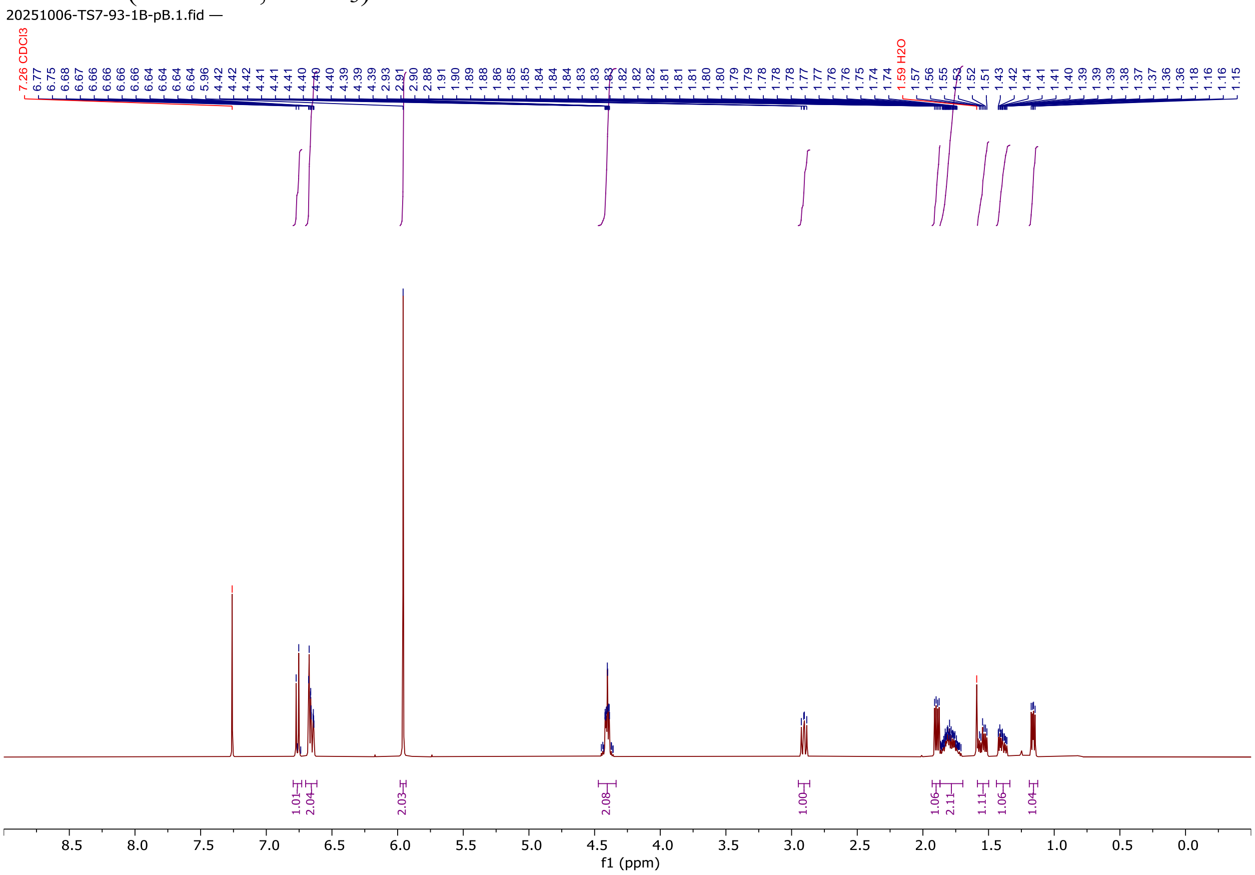

^{13}C NMR (101 MHz, $CDCl_3$)

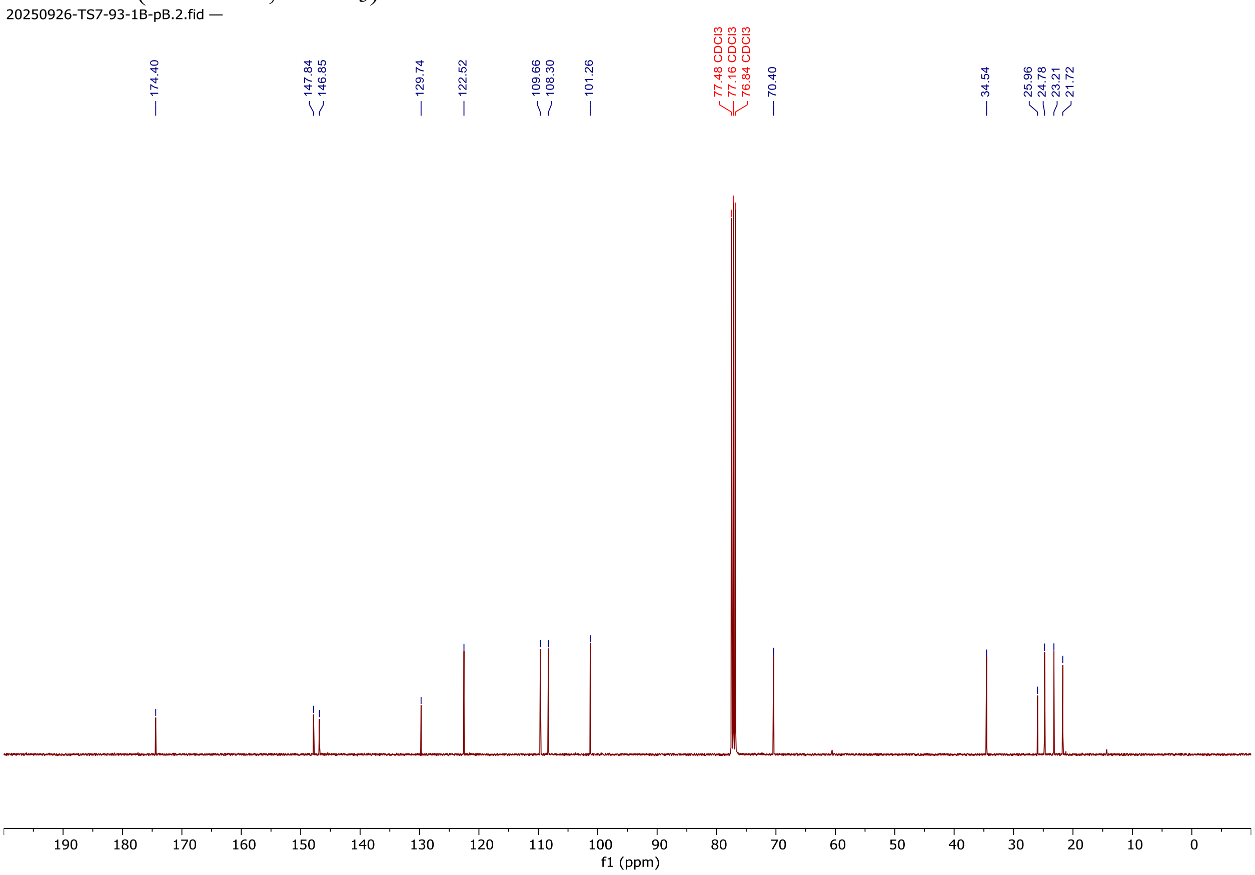

Chiral HPLC (210 nm trace):

Racemate, synthesized from $Rh_2(OAc)_4$

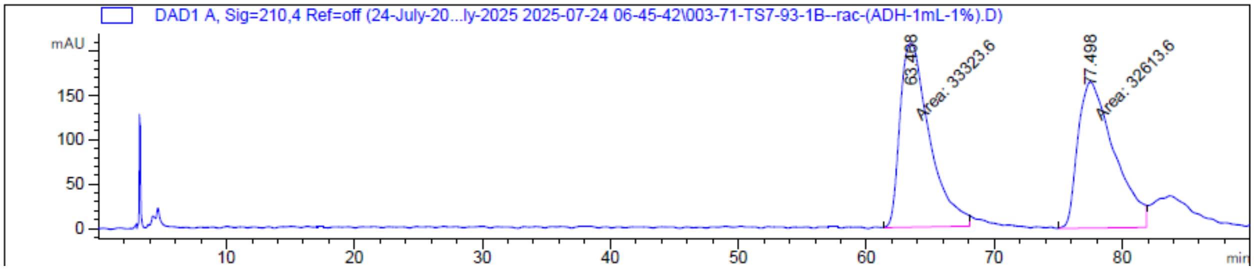

Signal 1: DAD1 A, Sig=210,4 Ref=off

Peak #	RetTime [min]	Type	Width [min]	Area [mAU*s]	Height [mAU]	Area %
1	63.468	MF	2.6686	3.33236e4	208.12183	50.5384
2	77.498	MF	3.2878	3.26136e4	165.32550	49.4616
Totals :				6.59372e4	373.44733	

Chiral, synthesized from Rh_2(*S*-*p*-PhTPCP)$_4$, 91% *ee*

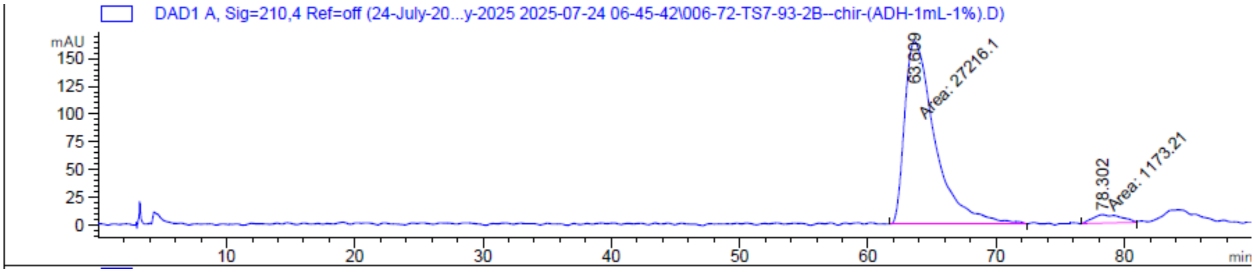

Signal 1: DAD1 A, Sig=210,4 Ref=off

Peak #	RetTime [min]	Type	Width [min]	Area [mAU*s]	Height [mAU]	Area %
1	63.609	MM	2.7521	2.72161e4	164.82159	95.8674
2	78.302	MM	2.5169	1173.21204	7.76898	4.1326
Totals :				2.83893e4	172.59057	

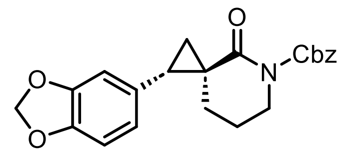

Benzyl (1*S*,3*R*)-1-(benzo[d][1,3]dioxol-5-yl)-4-oxo-5-azaspiro[2.5]octane-5-carboxylate (21b)

^{1}H NMR (400 MHz, CDCl3)

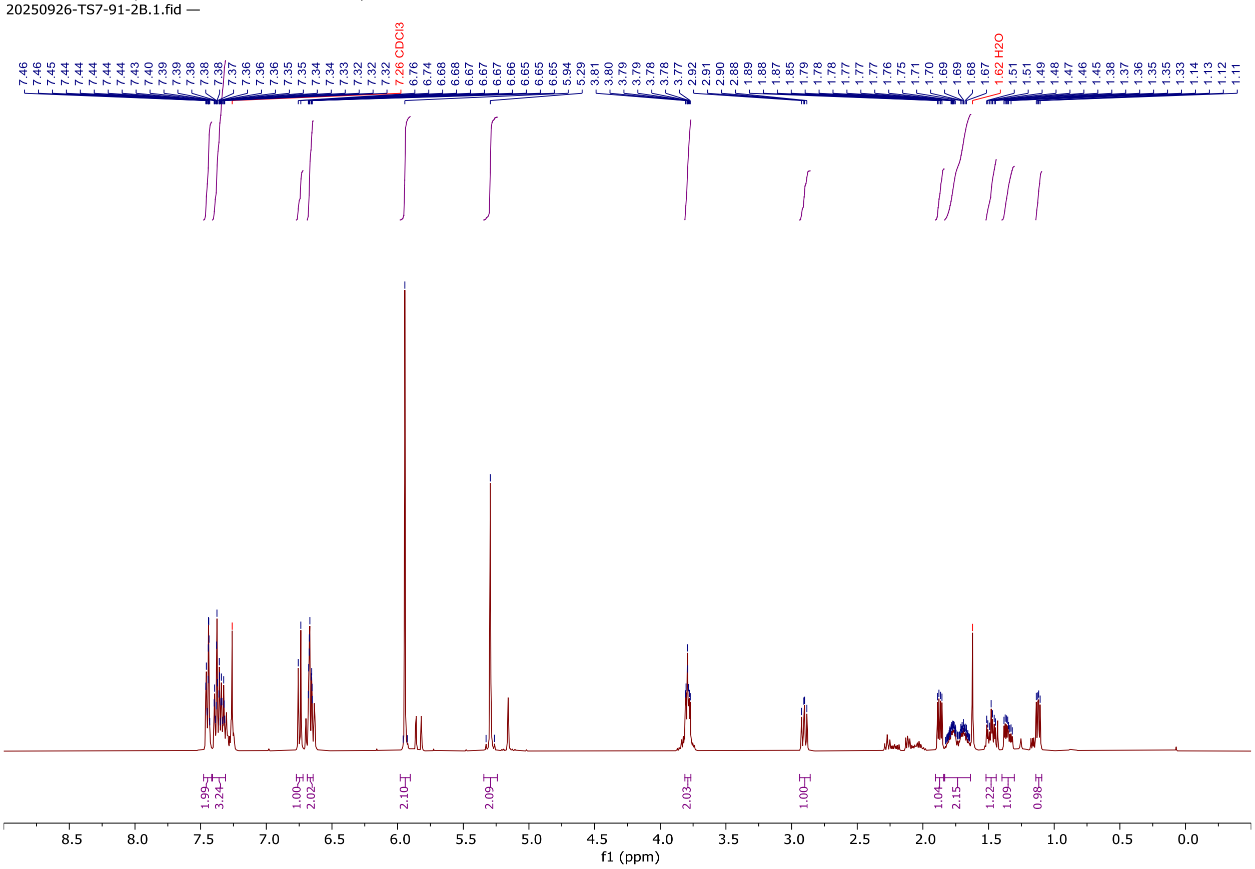

^{13}C NMR (101 MHz, $CDCl_3$)

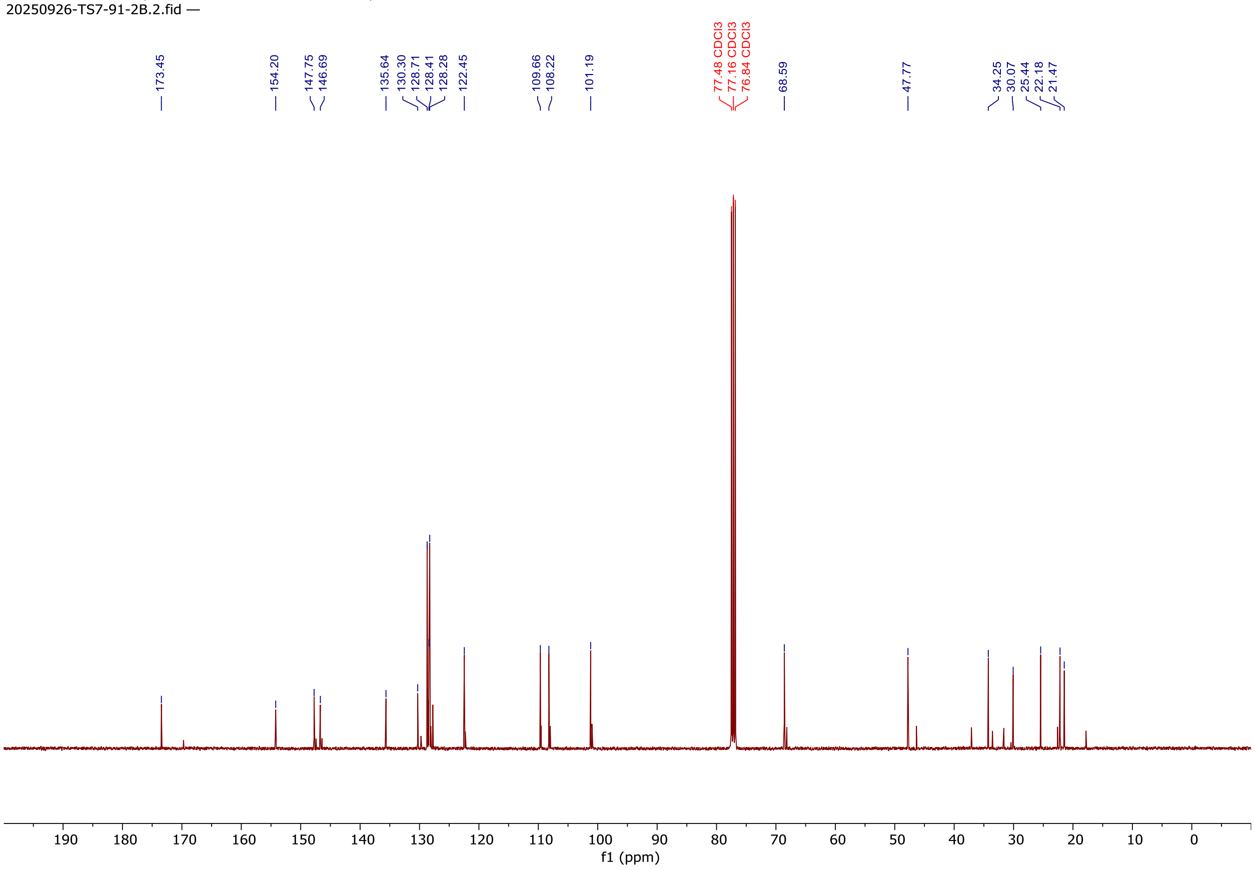

Chiral HPLC (210 nm trace):
Racemate, synthesized from $Rh_2(OAc)_4$

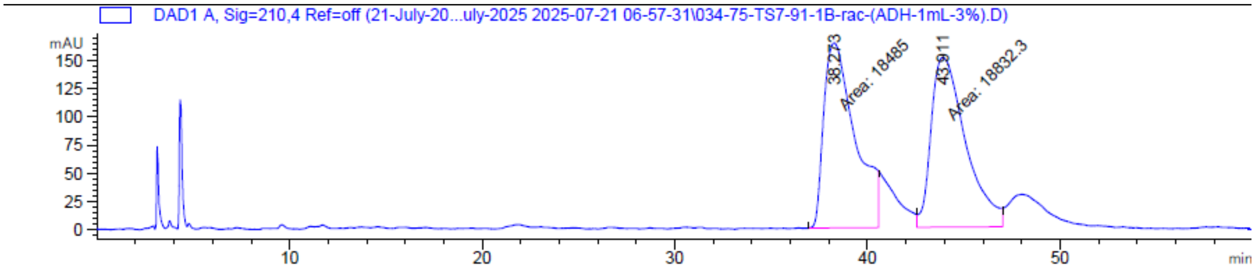


```
Signal 1: DAD1 A, Sig=210,4 Ref=off
```

Peak #	RetTime [min]	Type	Width [min]	Area [mAU*s]	Height [mAU]	Area %
1	38.273	MF	1.8772	1.84850e4	164.11473	49.5347
2	43.911	MF	2.0859	1.88323e4	150.47505	50.4653

Totals : 3.73172e4 314.58978

Chiral, synthesized from Rh_2(*S*-*p*-PhTPCP)$_4$, 97% *ee*

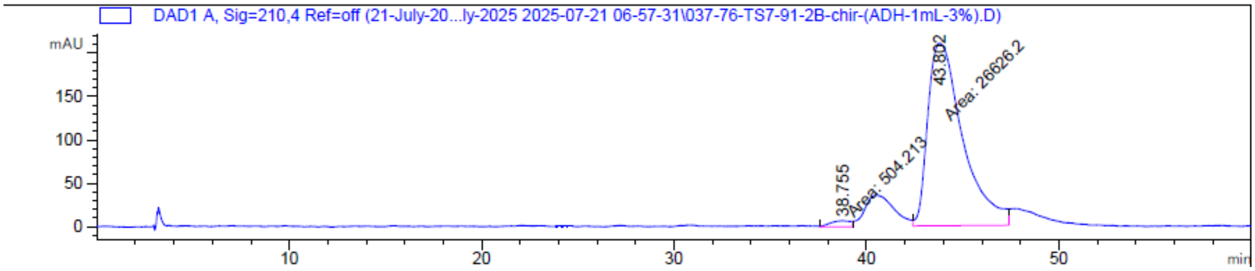


```
Signal 1: DAD1 A, Sig=210,4 Ref=off
```

Peak #	RetTime [min]	Type	Width [min]	Area [mAU*s]	Height [mAU]	Area %
1	38.755	MF	1.1792	504.21295	7.12646	1.8585
2	43.802	MF	2.1085	2.66262e4	210.46365	98.1415

Totals : 2.71304e4 217.59011

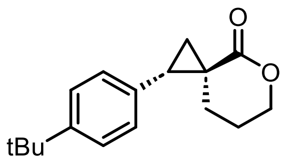

(1*S*,3*R*)-1-(4-(*tert*-Butyl)phenyl)-5-oxaspiro[2.5]octan-4-one (22a)

^{1}H NMR (400 MHz, $CDCl_3$)

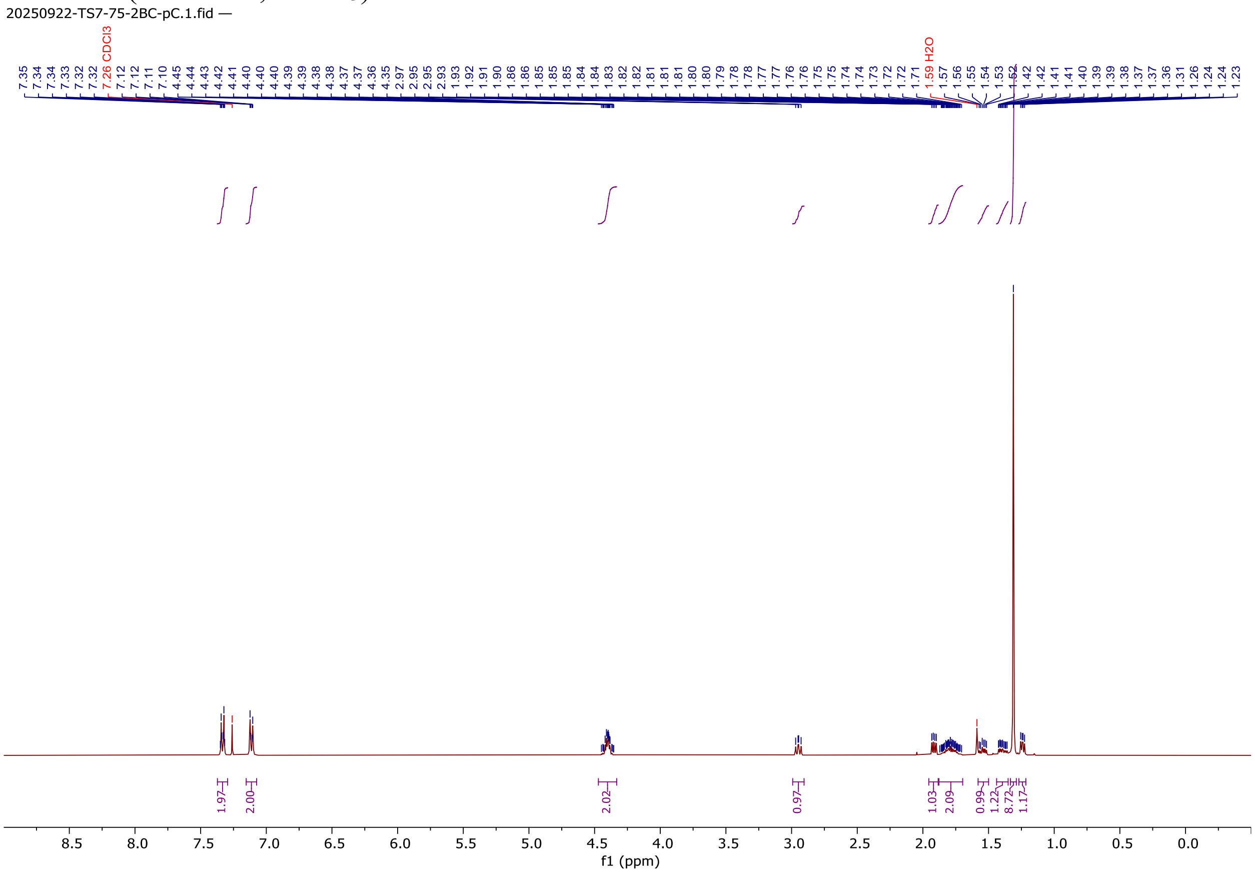

^{13}C NMR (101 MHz, $CDCl_3$)

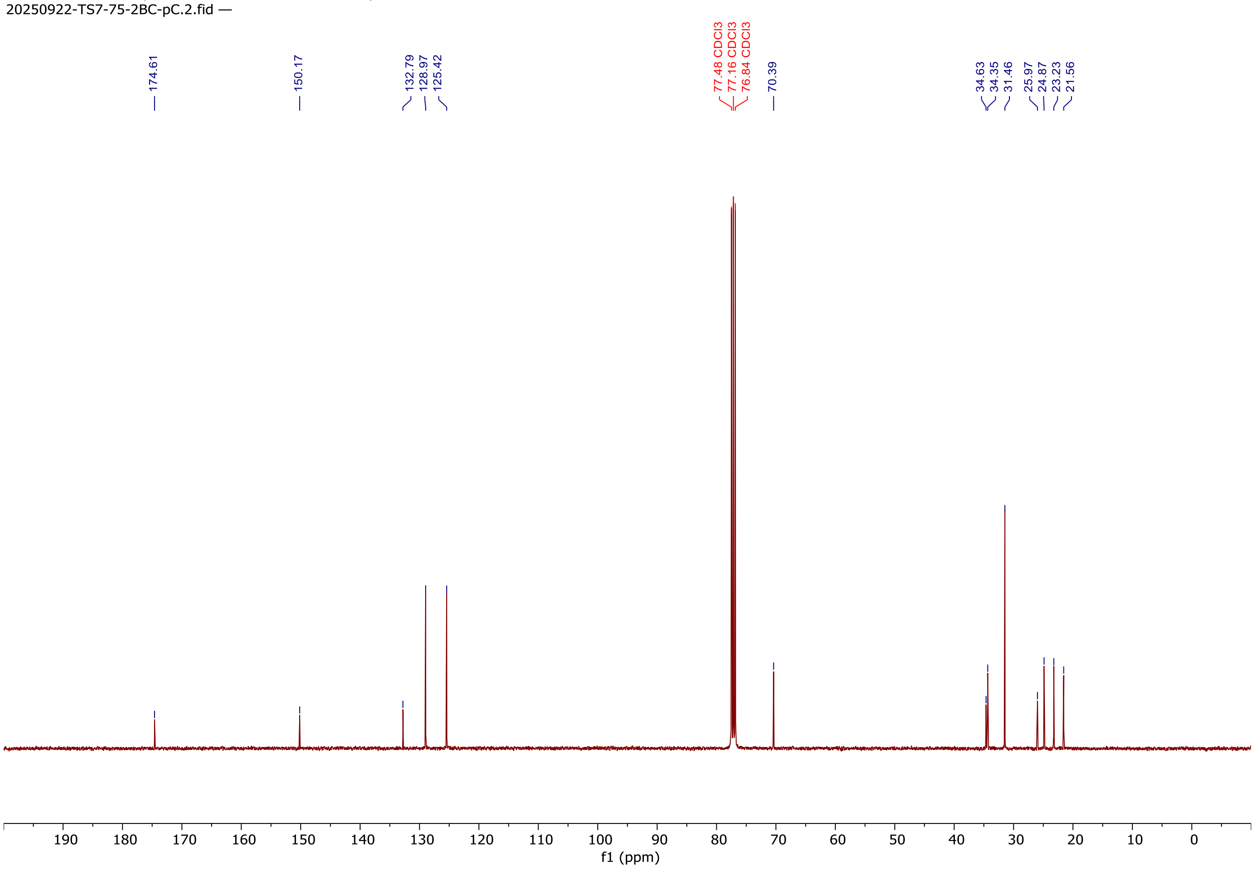

Chiral HPLC (210 nm trace):
Racemate, synthesized from $Rh_2(OAc)_4$

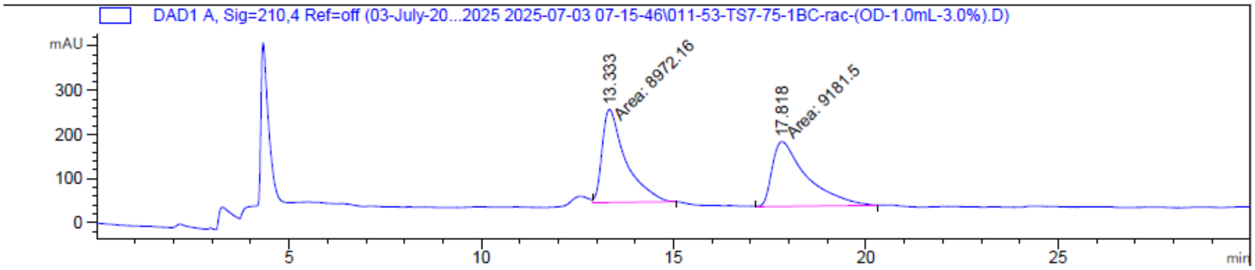

Signal 1: DAD1 A, Sig=210,4 Ref=off

Peak #	RetTime [min]	Type	Width [min]	Area [mAU*s]	Height [mAU]	Area %
1	13.333	MM	0.7103	8972.16309	210.52835	49.4234
2	17.818	MM	1.0447	9181.50488	146.48331	50.5766

Totals : 1.81537e4 357.01166

Chiral, synthesized from Rh_2(*S*-*p*-PhTPCP)$_4$, 94% *ee*

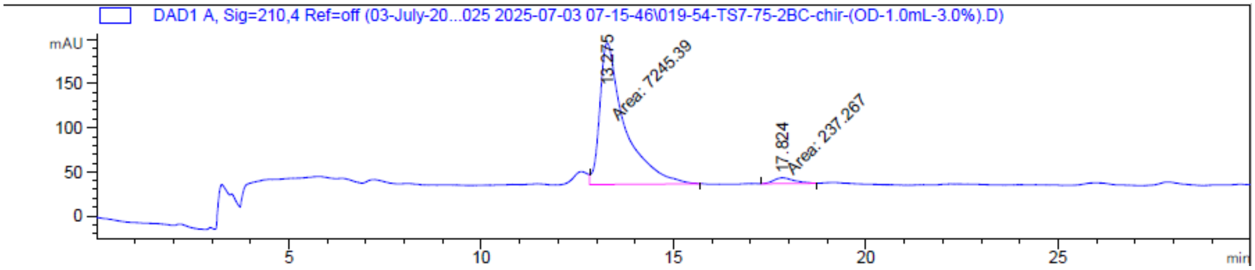

Signal 1: DAD1 A, Sig=210,4 Ref=off

Peak #	RetTime [min]	Type	Width [min]	Area [mAU*s]	Height [mAU]	Area %
1	13.275	FM	0.7534	7245.38525	160.28860	96.8291
2	17.824	MM	0.5909	237.26714	6.69249	3.1709

Totals : 7482.65239 166.98109

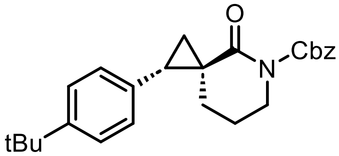

Benzyl (1*S*,3*R*)-1-(4-(*tert*-butyl)phenyl)-4-oxo-5-azaspiro[2.5]octane-5-carboxylate (22b)

^{1}H NMR (400 MHz, $CDCl_3$)

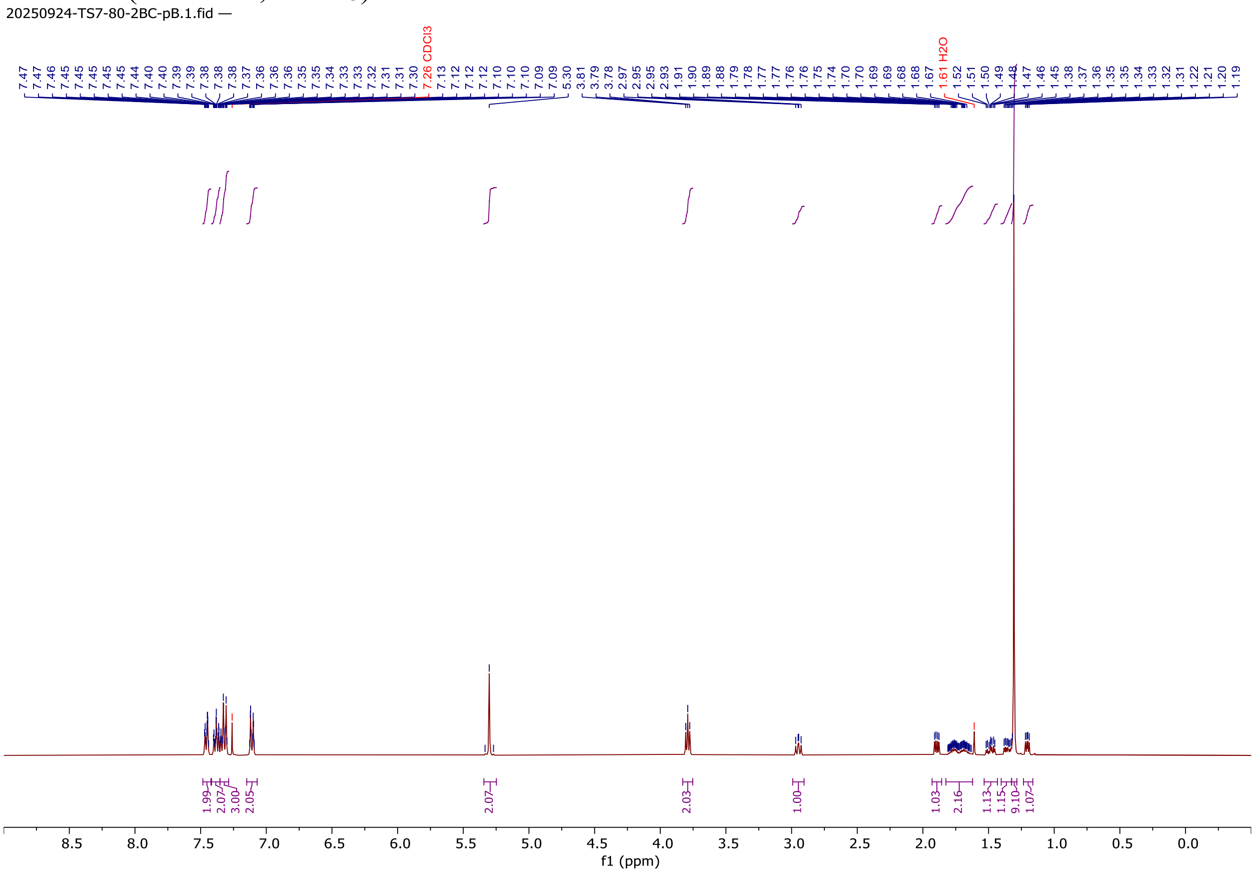

^{13}C NMR (101 MHz, $CDCl_3$)

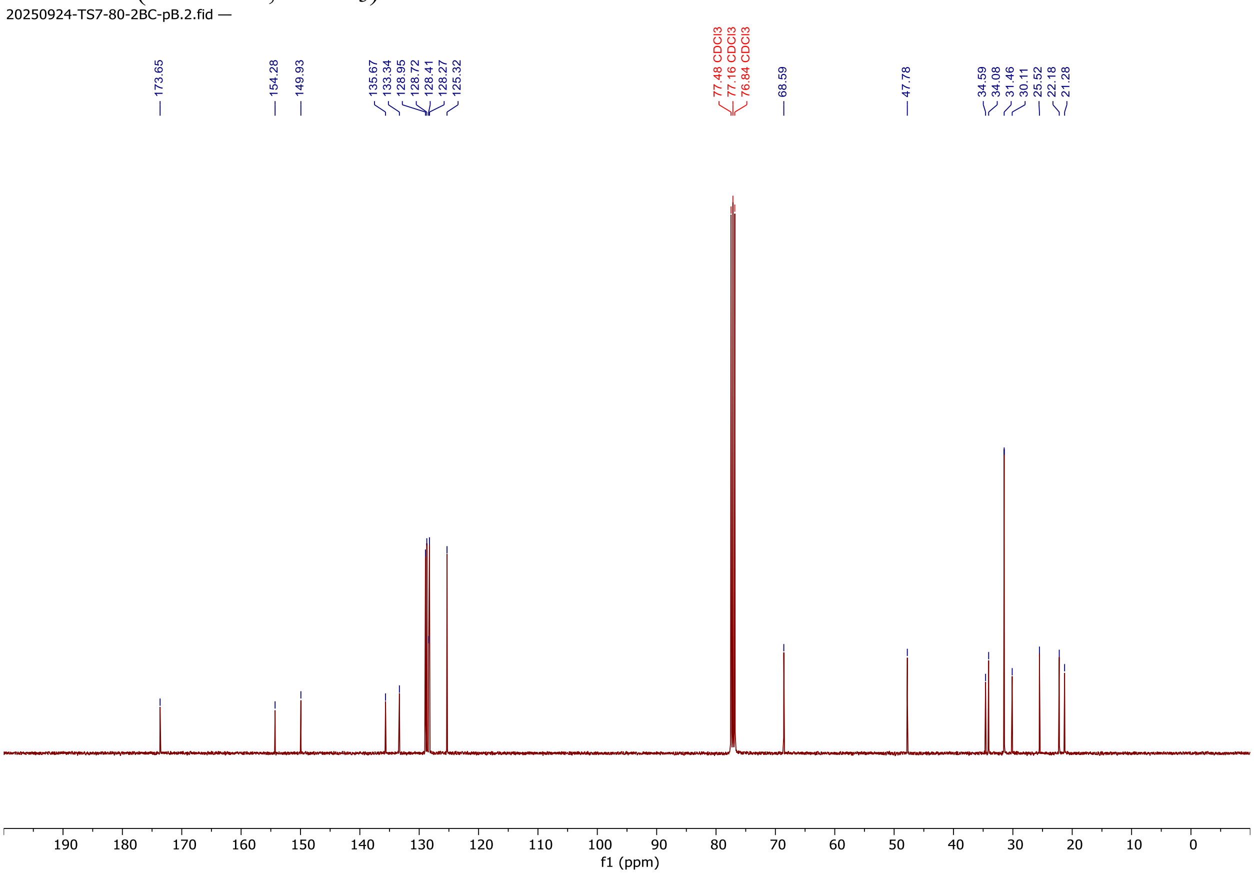

Chiral HPLC (210 nm trace):
Racemate, synthesized from $Rh_2(OAc)_4$

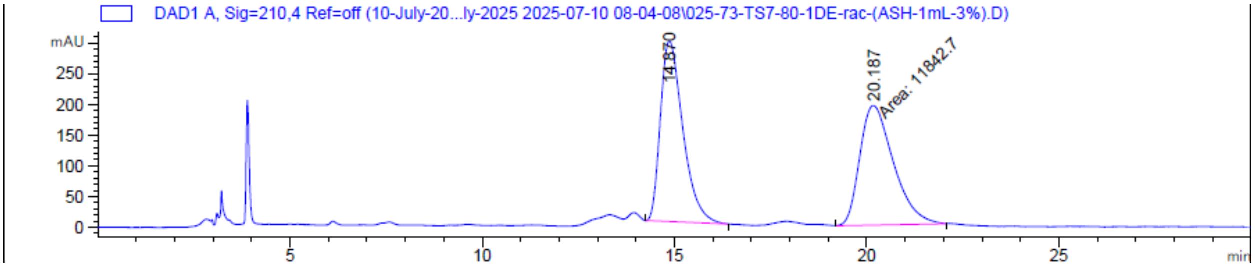

Signal 1: DAD1 A, Sig=210,4 Ref=off

Peak #	RetTime [min]	Type	Width [min]	Area [mAU*s]	Height [mAU]	Area %
1	14.870	VV R	0.4761	1.19562e4	293.90451	50.2385
2	20.187	MM	1.0135	1.18427e4	194.75456	49.7615
Totals :				2.37990e4	488.65907	

Chiral, synthesized from Rh_2(*S*-*p*-PhTPCP)$_4$, 80% *ee*

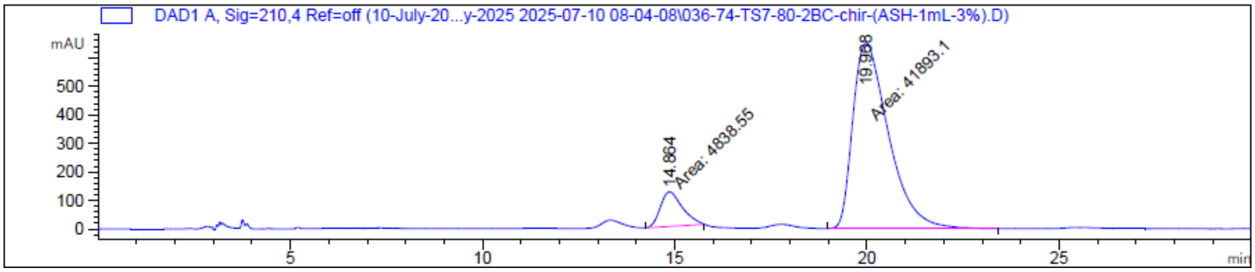

Signal 1: DAD1 A, Sig=210,4 Ref=off

Peak #	RetTime [min]	Type	Width [min]	Area [mAU*s]	Height [mAU]	Area %
1	14.864	MM	0.6631	4838.55322	121.61485	10.3539
2	19.968	MM	1.0749	4.18931e4	649.57672	89.6461
Totals :				4.67316e4	771.19157	

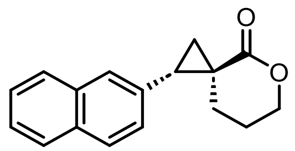

(1*S*,3*R*)-1-(Naphthalen-2-yl)-5-oxaspiro[2.5]octan-4-one (23a)

^{1}H NMR (400 MHz, $CDCl_3$)

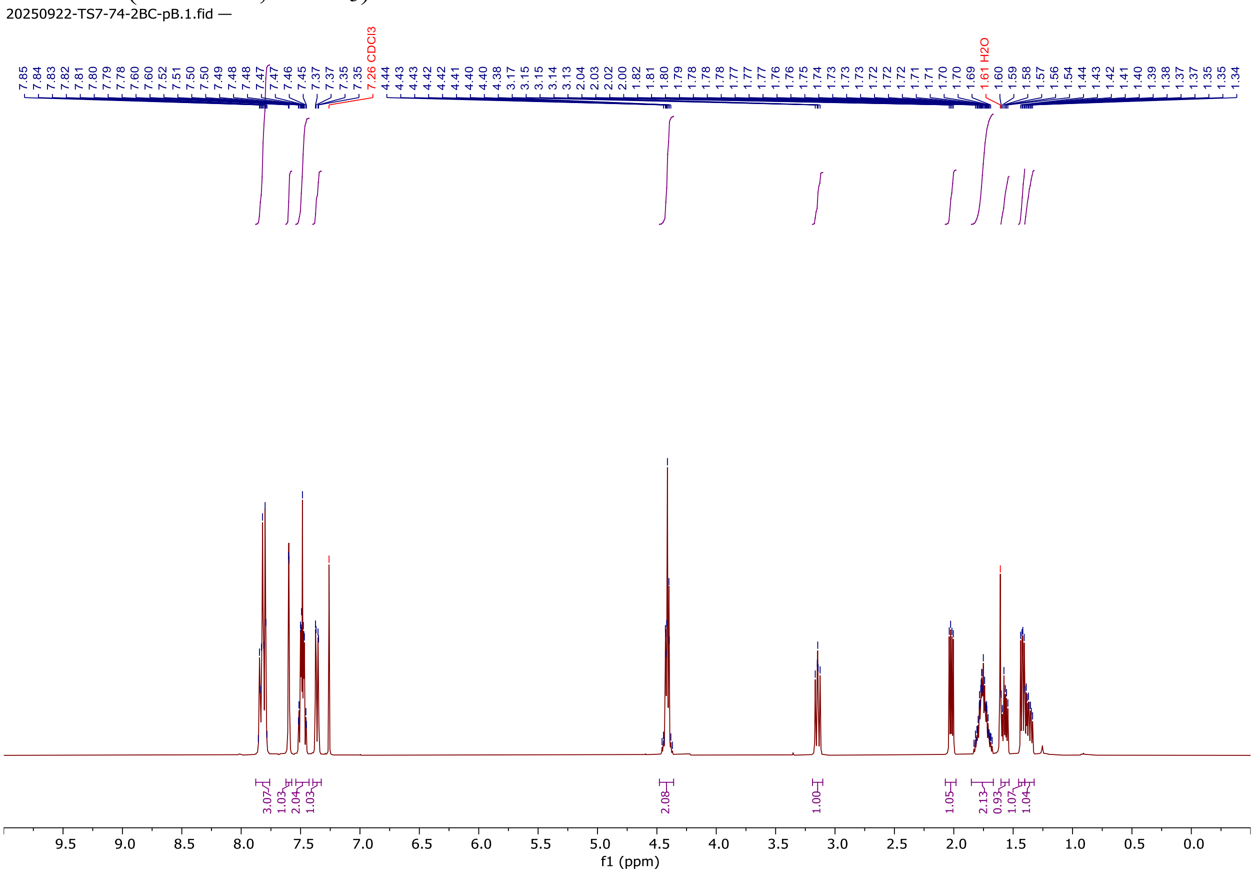

^{13}C NMR (101 MHz, $CDCl_3$)

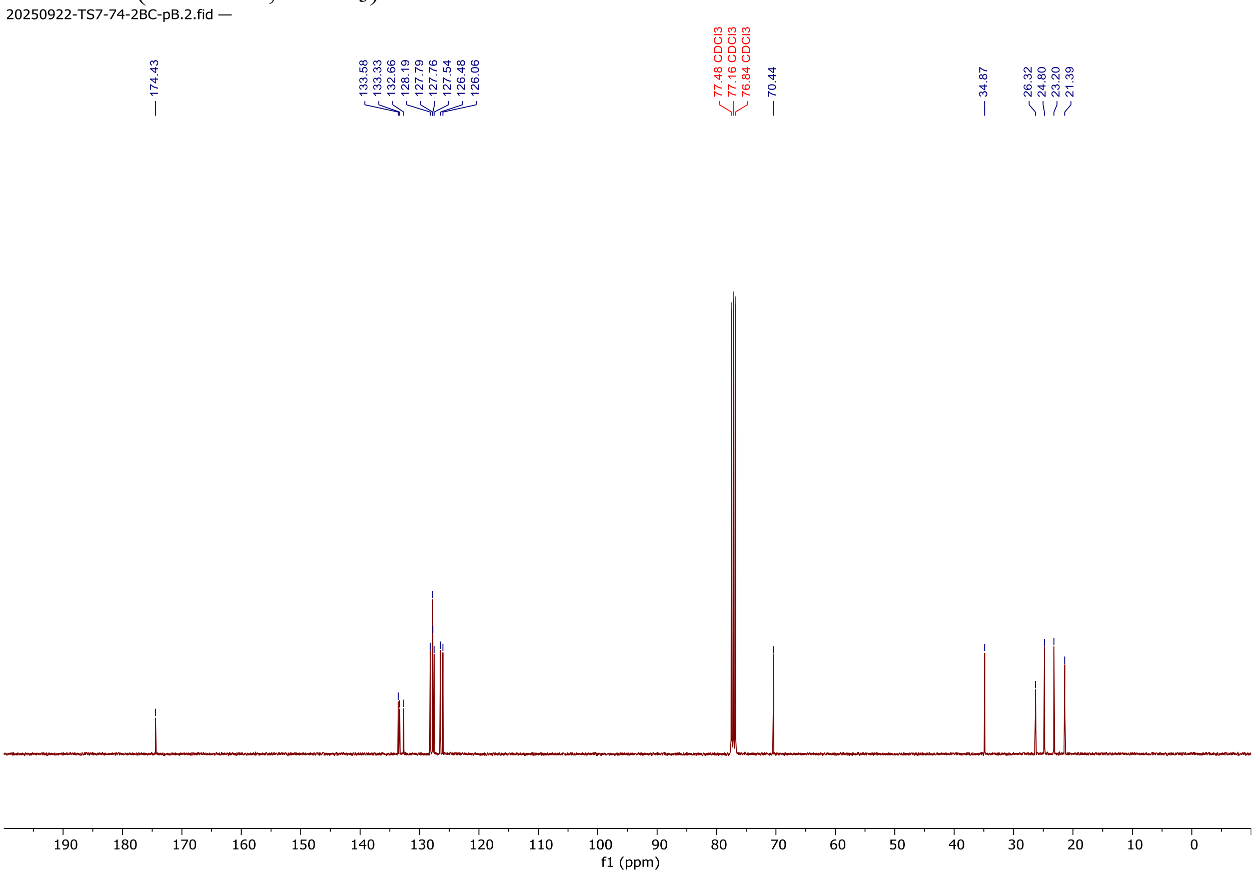

Chiral HPLC (210 nm trace):
Racemate, synthesized from $Rh_2(OAc)_4$

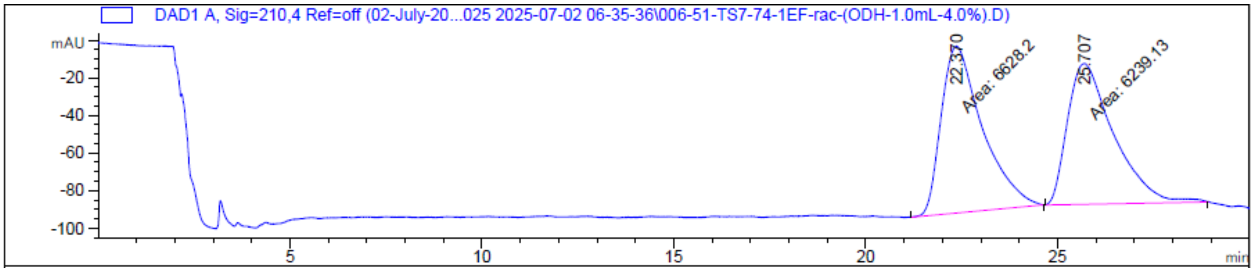

Signal 1: DAD1 A, Sig=210,4 Ref=off

Peak #	RetTime [min]	Type	Width [min]	Area [mAU*s]	Height [mAU]	Area %
1	22.370	MM	1.2446	6628.20117	88.75615	51.5118
2	25.707	MM	1.3888	6239.13330	74.87505	48.4882

Totals : 1.28673e4 163.63120

Chiral, synthesized from Rh_2(*S*-*p*-PhTPCP)$_4$, 94% *ee*

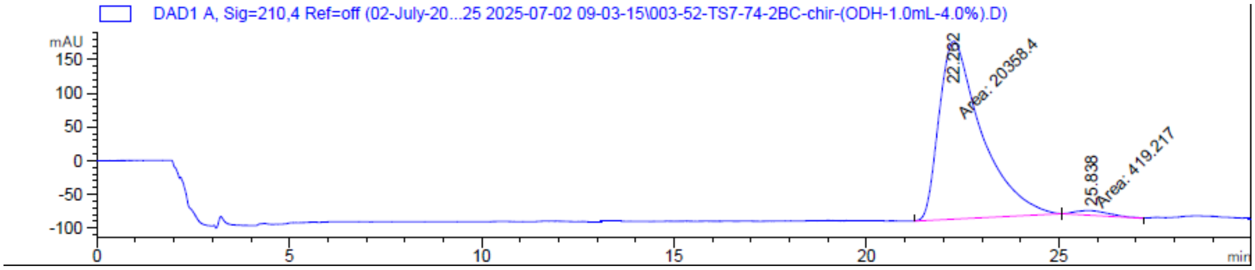

Signal 1: DAD1 A, Sig=210,4 Ref=off

Peak #	RetTime [min]	Type	Width [min]	Area [mAU*s]	Height [mAU]	Area %
1	22.262	MM	1.2837	2.03584e4	264.32751	97.9824
2	25.838	MM	0.9957	419.21680	7.01678	2.0176

Totals : 2.07776e4 271.34430

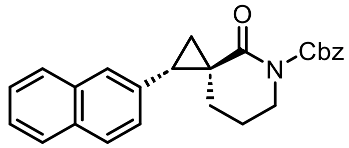

Benzyl (1*S*,3*R*)-1-(naphthalen-2-yl)-4-oxo-5-azaspiro[2.5]octane-5-carboxylate (23b)

^{1}H NMR (400 MHz, $CDCl_3$)

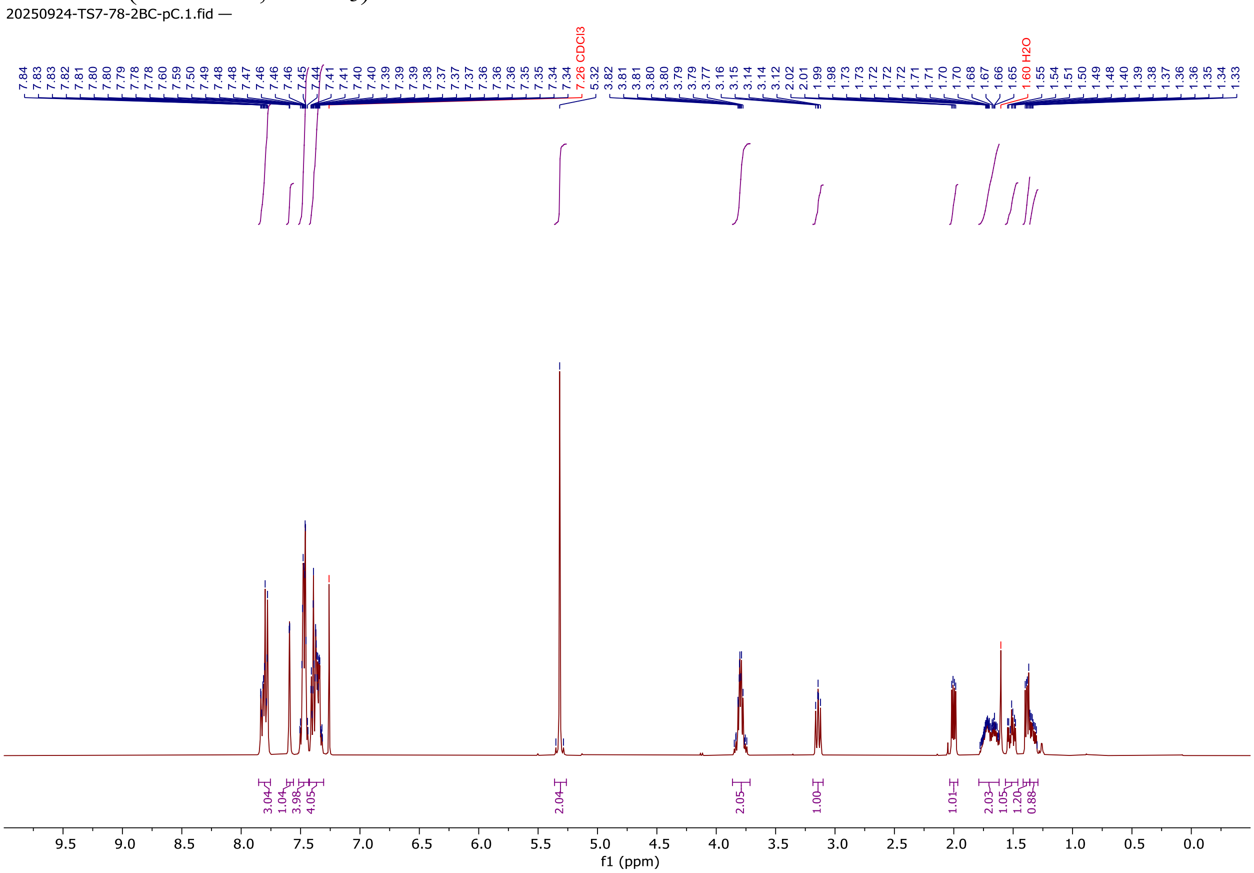

^{13}C NMR (101 MHz, $CDCl_3$)

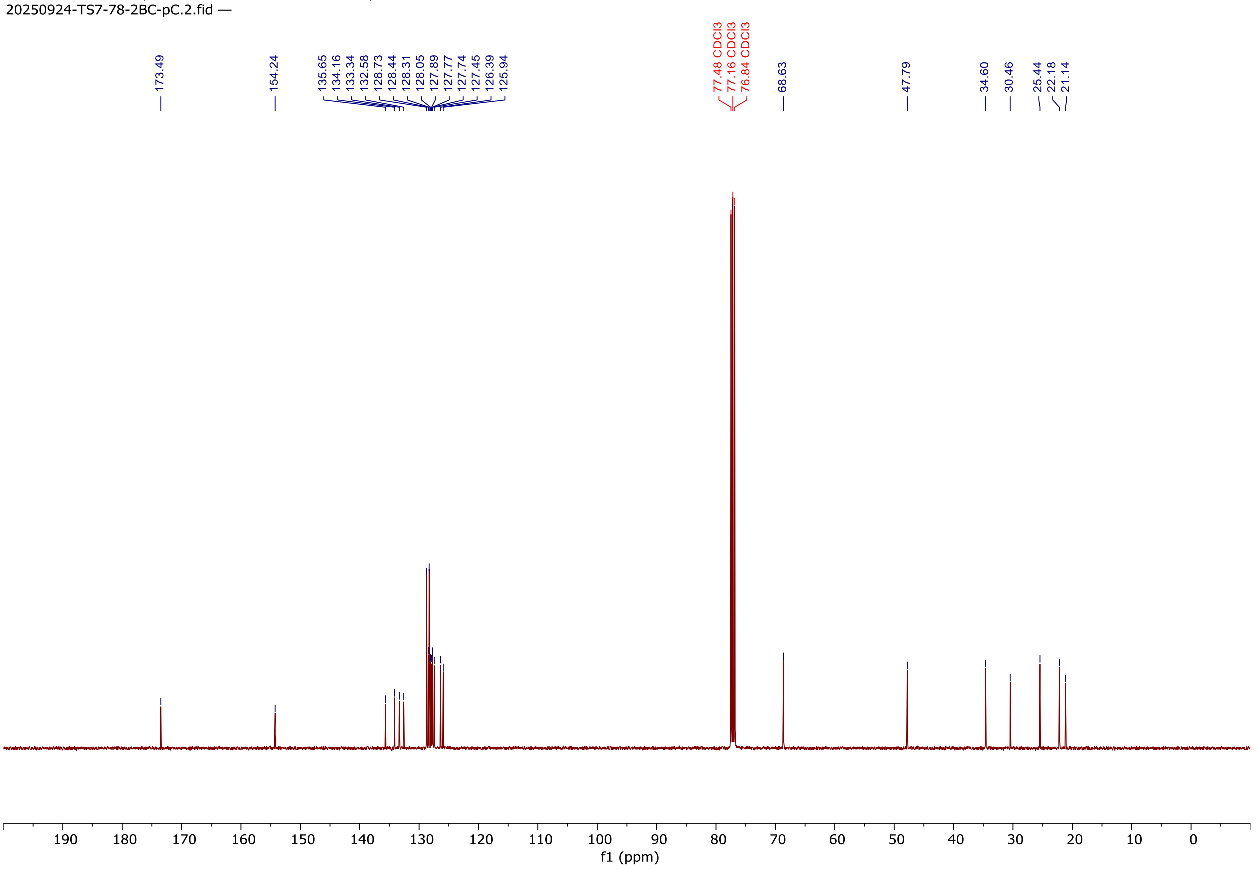

Chiral HPLC (210 nm trace):
Racemate, synthesized from $Rh_2(OAc)_4$

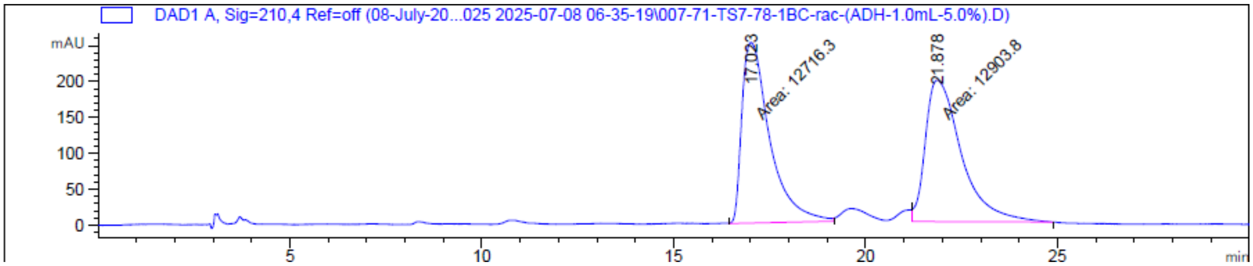

Signal 1: DAD1 A, Sig=210,4 Ref=off

Peak #	RetTime [min]	Type	Width [min]	Area [mAU*s]	Height [mAU]	Area %
1	17.023	MM	0.8414	1.27163e4	251.87694	49.6340
2	21.878	MM	1.0893	1.29038e4	197.42911	50.3660
Totals :				2.56201e4	449.30605	

Chiral, synthesized from Rh_2(*S*-*p*-PhTPCP)$_4$, 96% *ee*

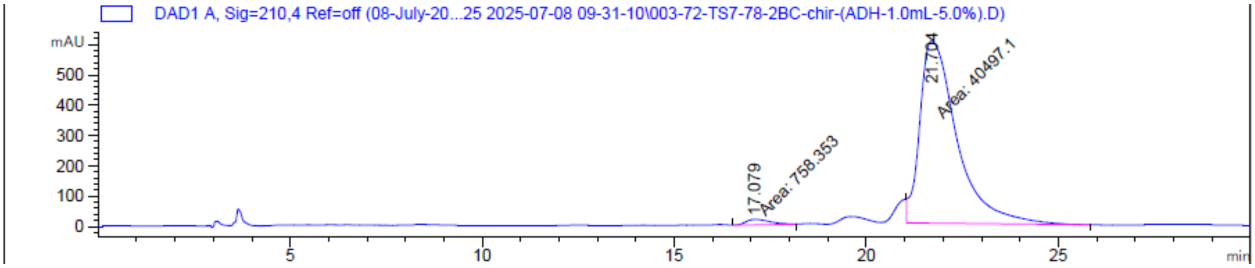

Signal 1: DAD1 A, Sig=210,4 Ref=off

Peak #	RetTime [min]	Type	Width [min]	Area [mAU*s]	Height [mAU]	Area %
1	17.079	MM	0.7131	758.35321	17.72510	1.8382
2	21.704	FM	1.1239	4.04971e4	600.54565	98.1618
Totals :				4.12555e4	618.27075	

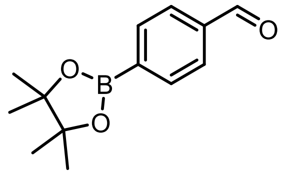

4-(4,4,5,5-tetramethyl-1,3,2-dioxaborolan-2-yl)benzaldehyde (SI-4)

^{1}H NMR (400 MHz, $CDCl_3$)

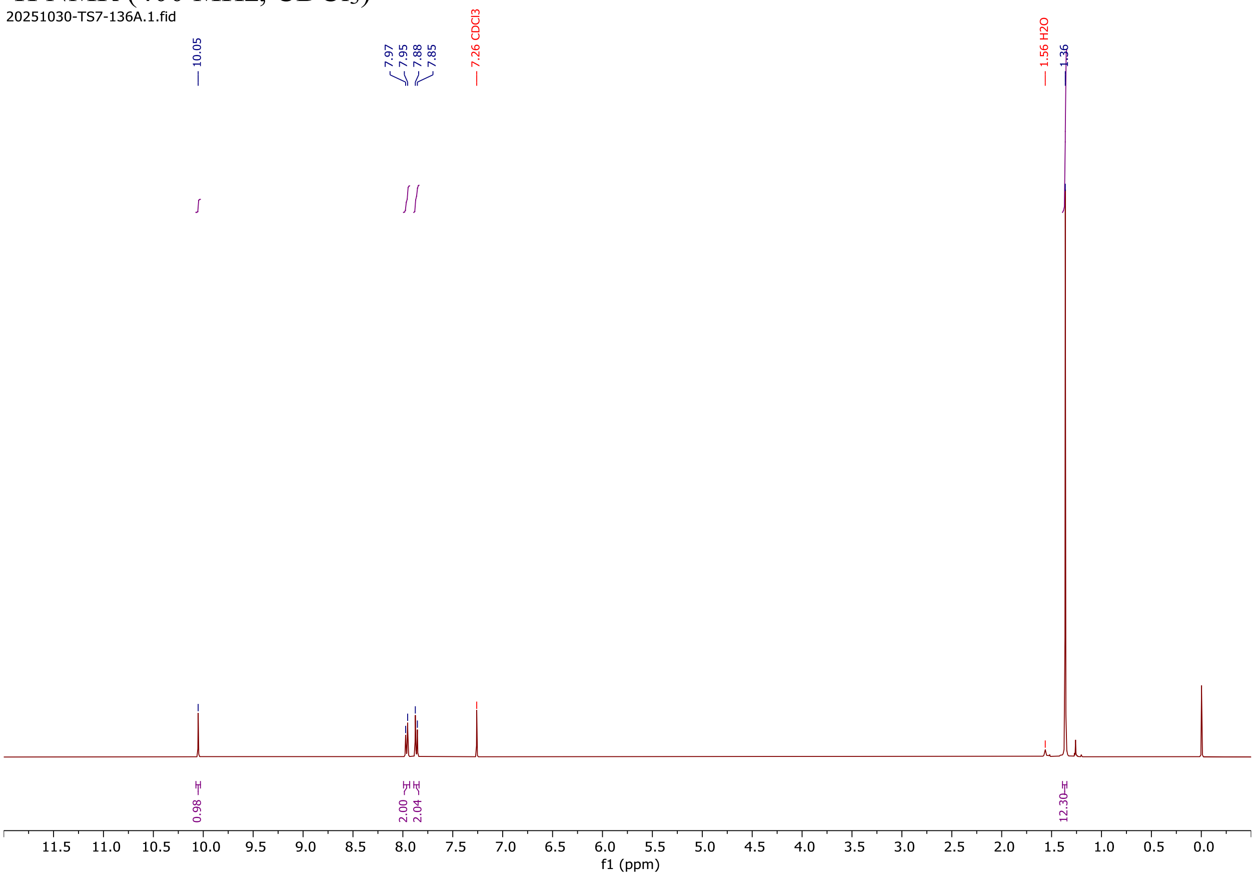

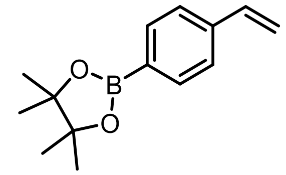

4,4,5,5-tetramethyl-2-(4-vinylphenyl)-1,3,2-dioxaborolane (SI-5)

^{1}H NMR (400 MHz, $CDCl_3$)

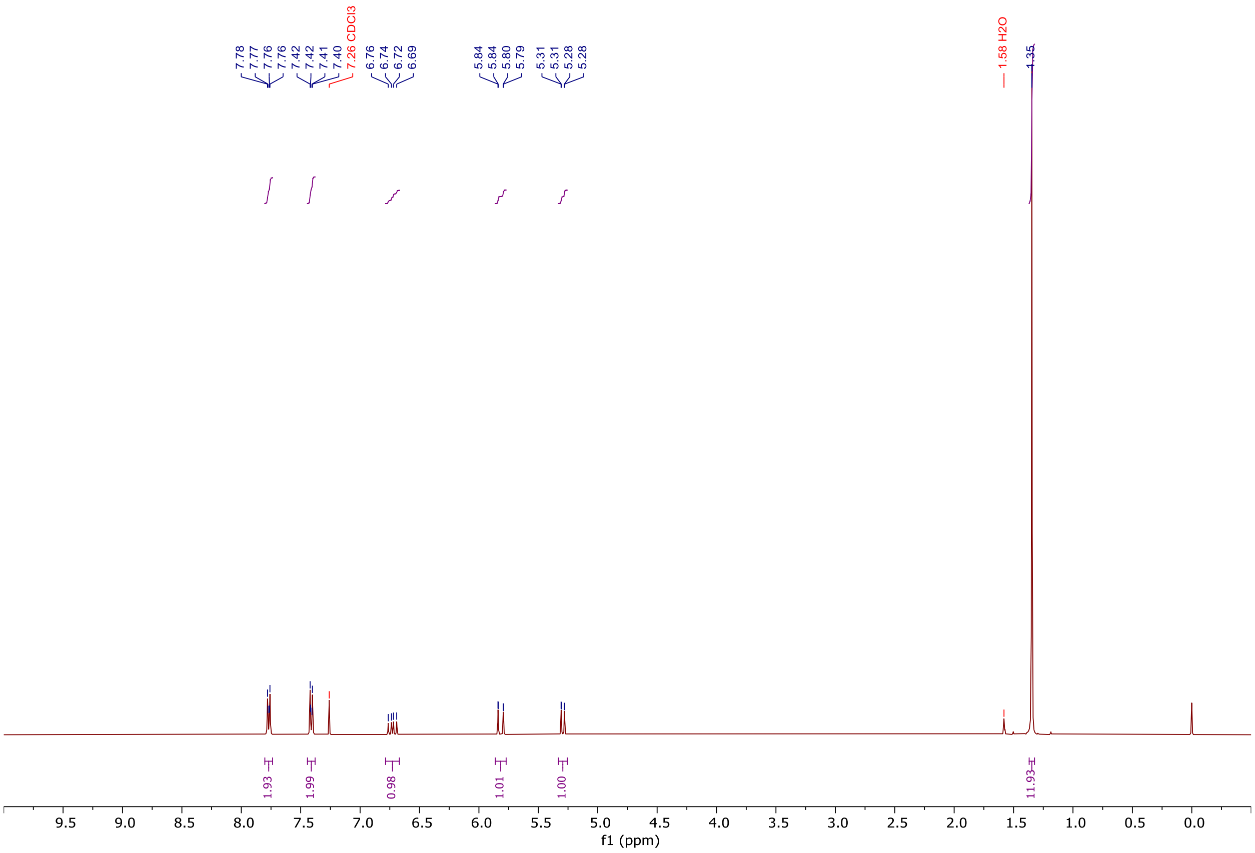

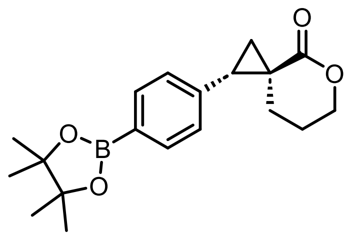

(1*S*,3*R*)-1-(4-(4,4,5,5-tetramethyl-1,3,2-dioxaborolan-2-yl)phenyl)-5-oxaspiro[2.5]octan-4-one (24a)

^{1}H NMR (400 MHz, $CDCl_3$)

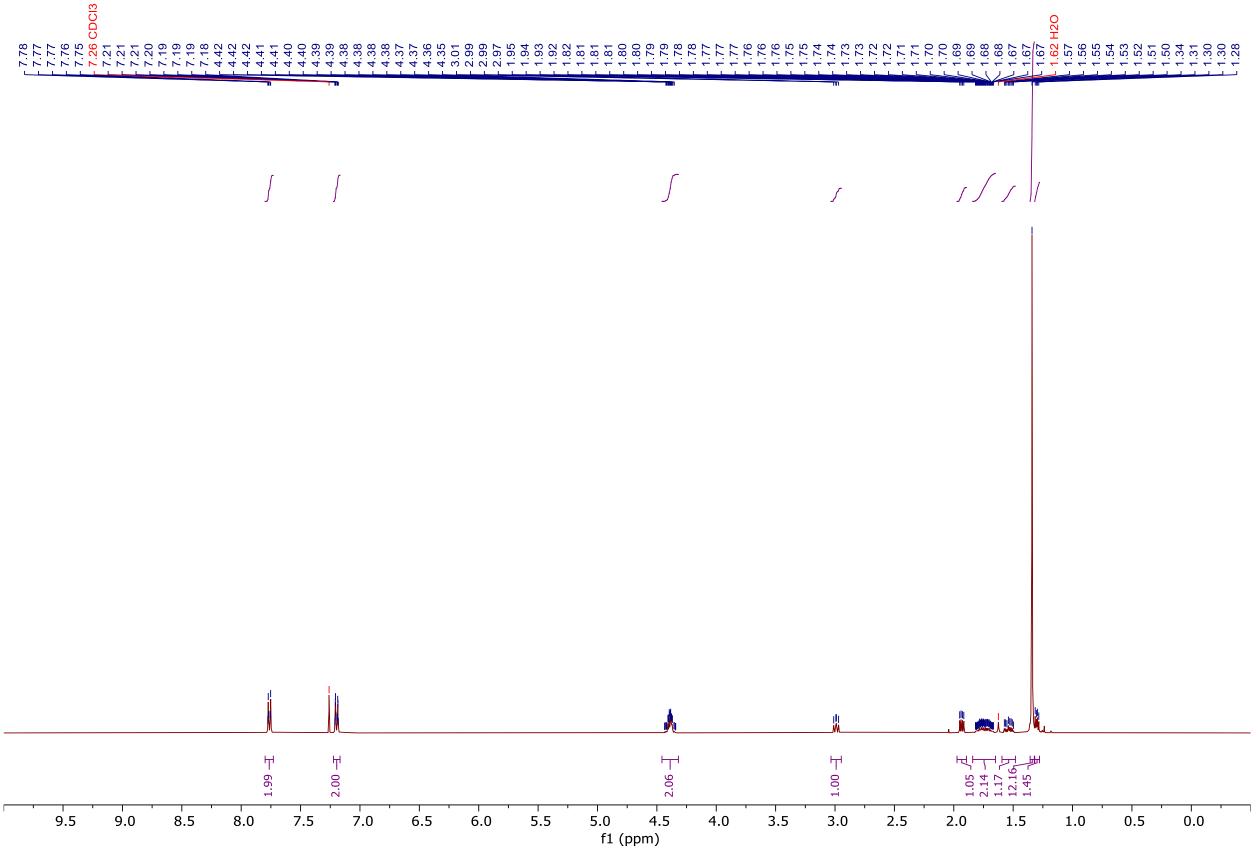

^{13}C NMR (101 MHz, $CDCl_3$)

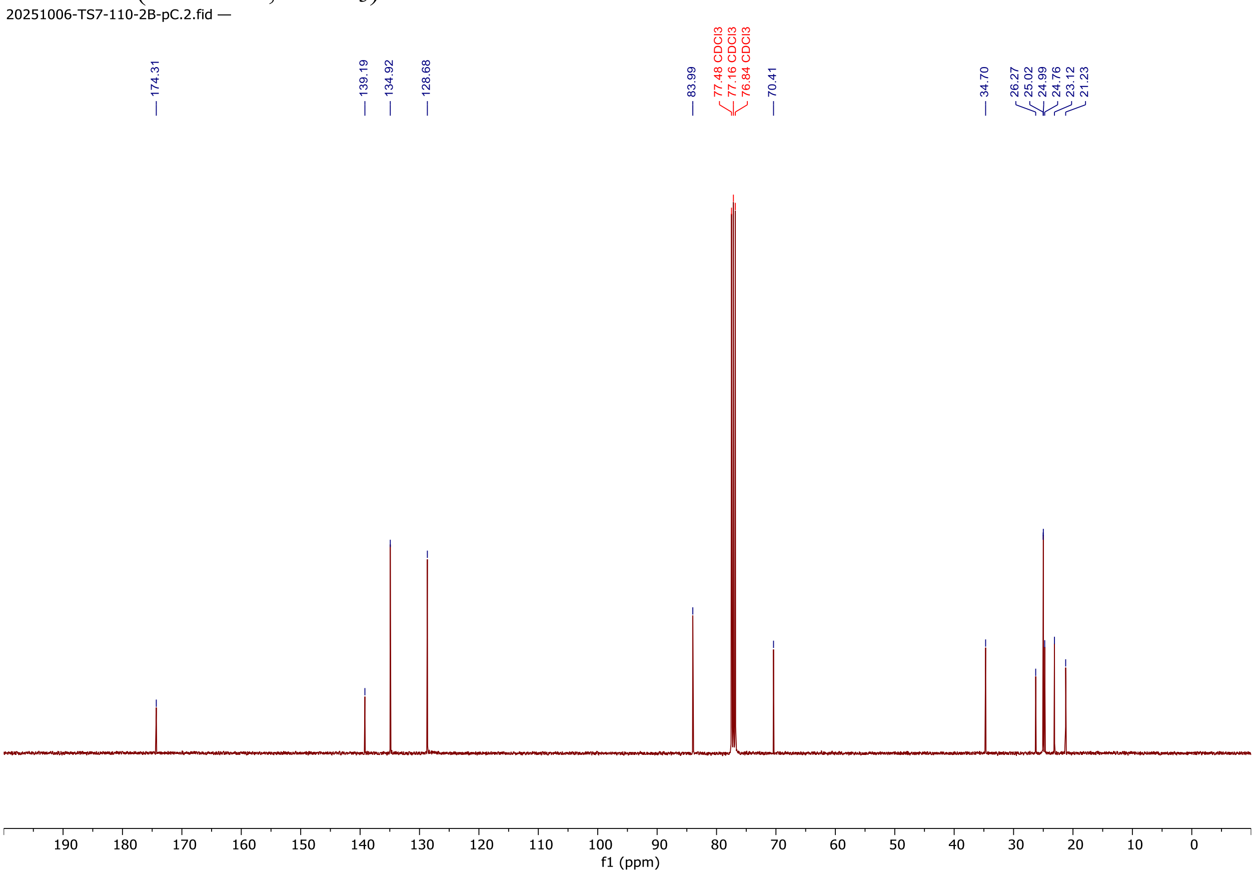

Chiral HPLC (210 nm trace):
Racemate, synthesized from $Rh_2(OAc)_4$

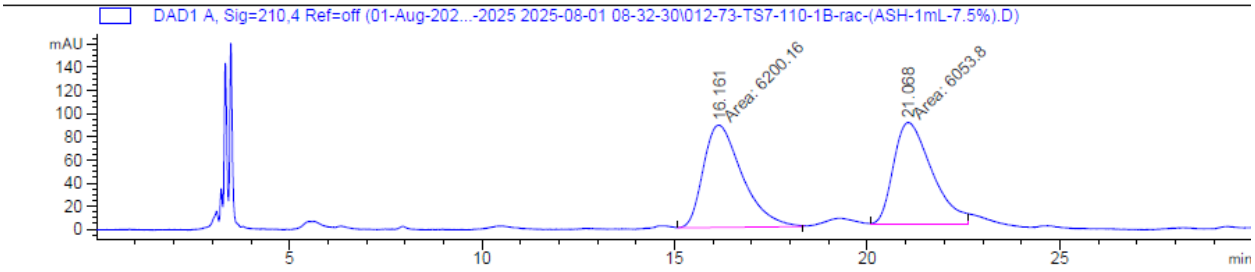

Signal 1: DAD1 A, Sig=210,4 Ref=off

Peak #	RetTime [min]	Type	Width [min]	Area [mAU*s]	Height [mAU]	Area %
1	16.161	MM	1.1695	6200.15967	88.35592	50.5972
2	21.068	MM	1.1524	6053.80078	87.55467	49.4028
Totals :				1.22540e4	175.91059	

Chiral, synthesized from Rh_2(*S*-*p*-PhTPCP)$_4$, 94% *ee*

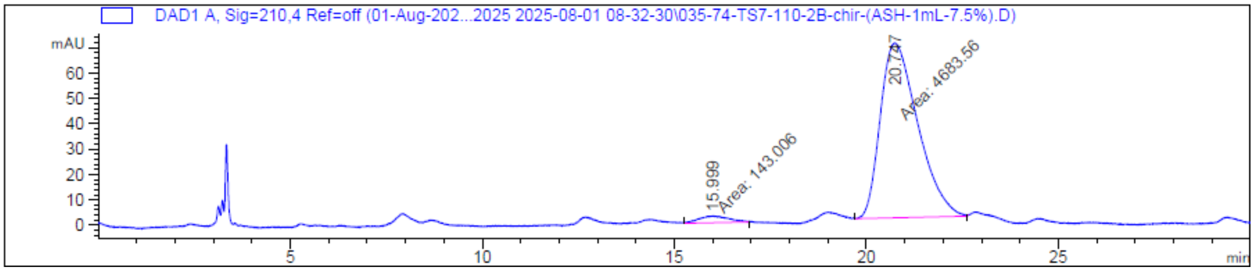

Signal 1: DAD1 A, Sig=210,4 Ref=off

Peak #	RetTime [min]	Type	Width [min]	Area [mAU*s]	Height [mAU]	Area %
1	15.999	MM	0.8767	143.00616	2.71853	2.9629
2	20.747	MM	1.1309	4683.56250	69.02375	97.0371
Totals :				4826.56866	71.74228	

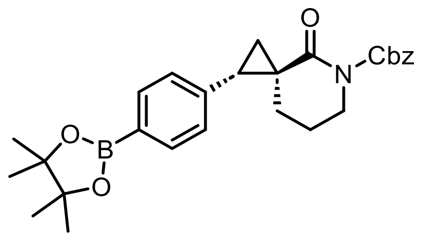

Benzyl (1*S*,3*R*)-4-oxo-1-(4-(4,4,5,5-tetramethyl-1,3,2-dioxaborolan-2-yl)phenyl)-5-azaspiro[2.5]-octane-5-carboxylate (24b)

^{1}H NMR (400 MHz, $CDCl_3$)

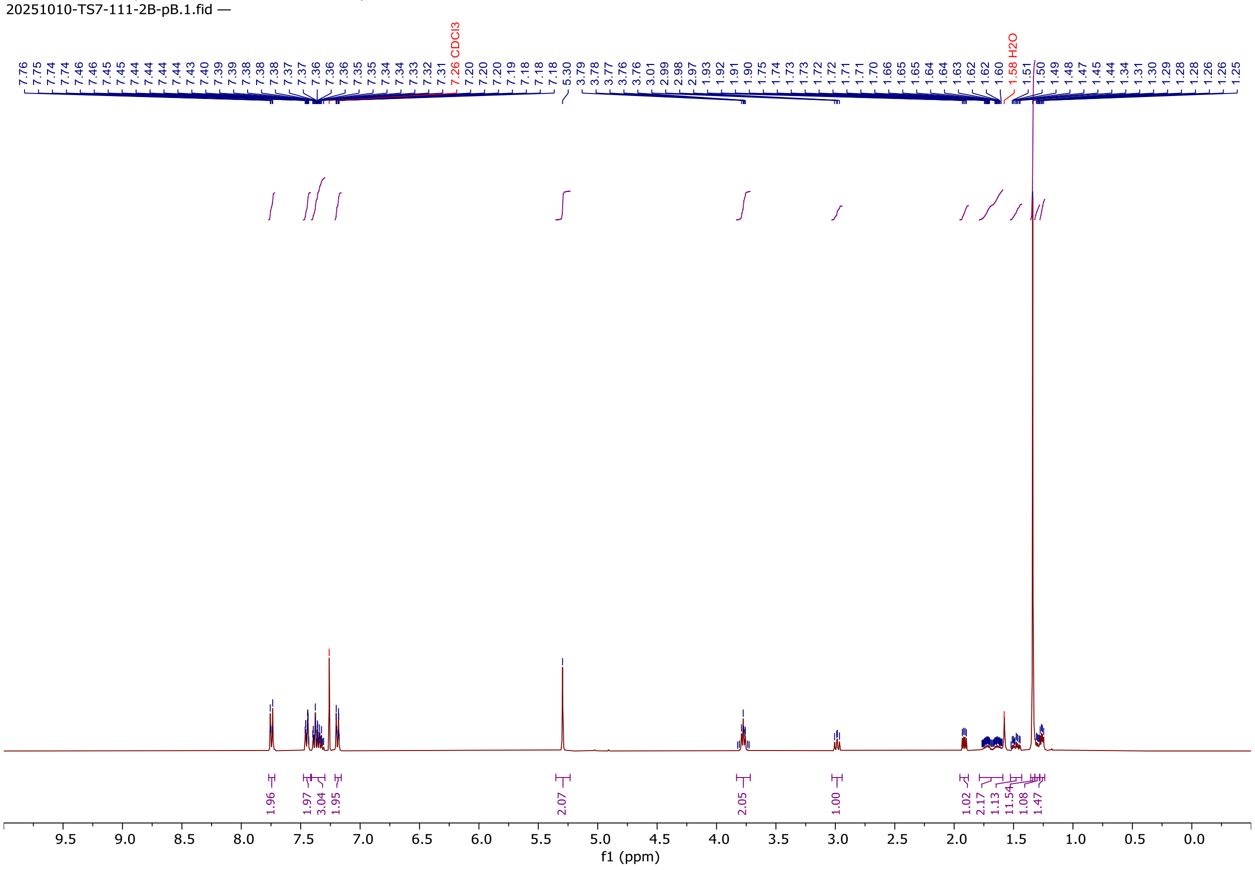

^{13}C NMR (101 MHz, $CDCl_3$)

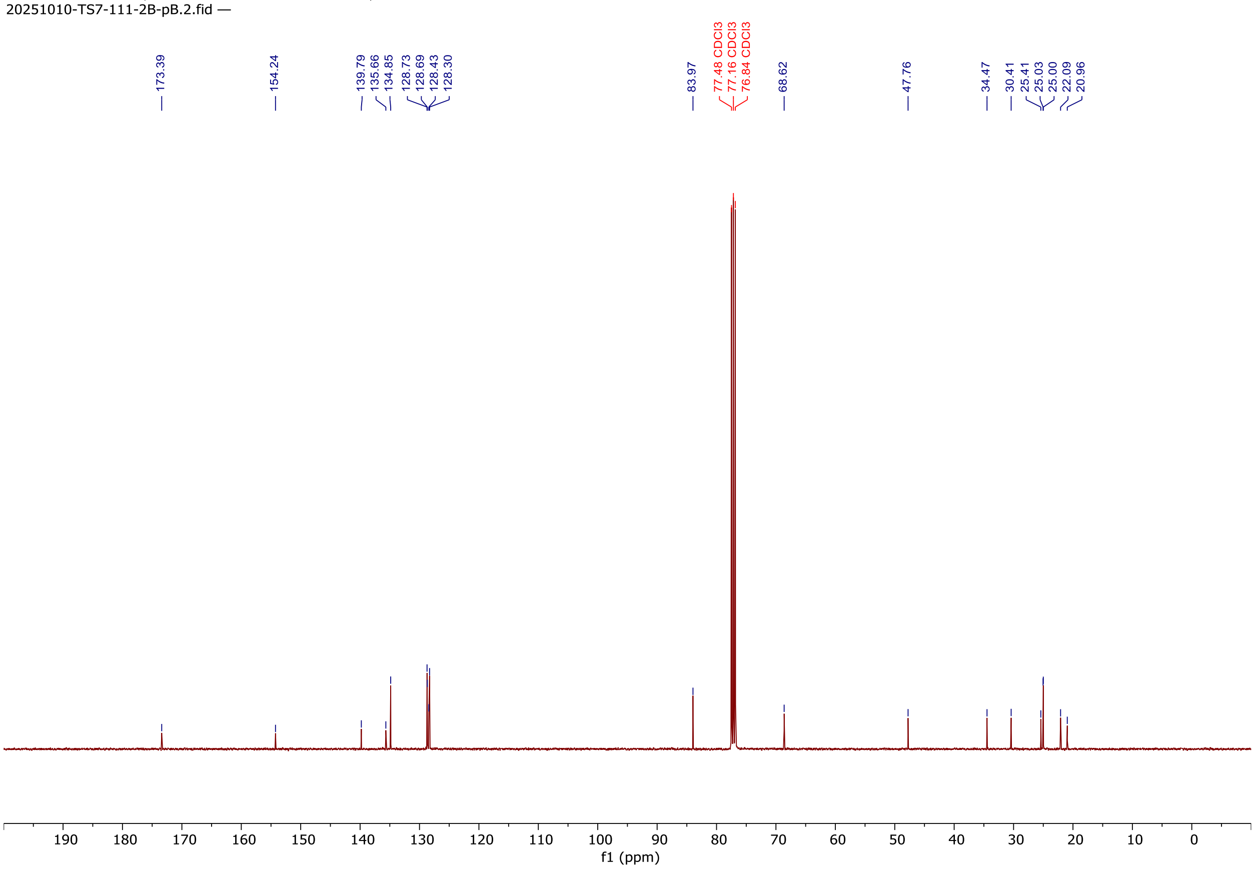

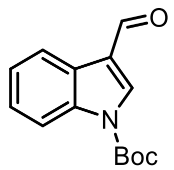

tert-Butyl 3-formyl-1H-indole-1-carboxylate (SI-6)

^1H NMR (400 MHz, $CDCl_3$)

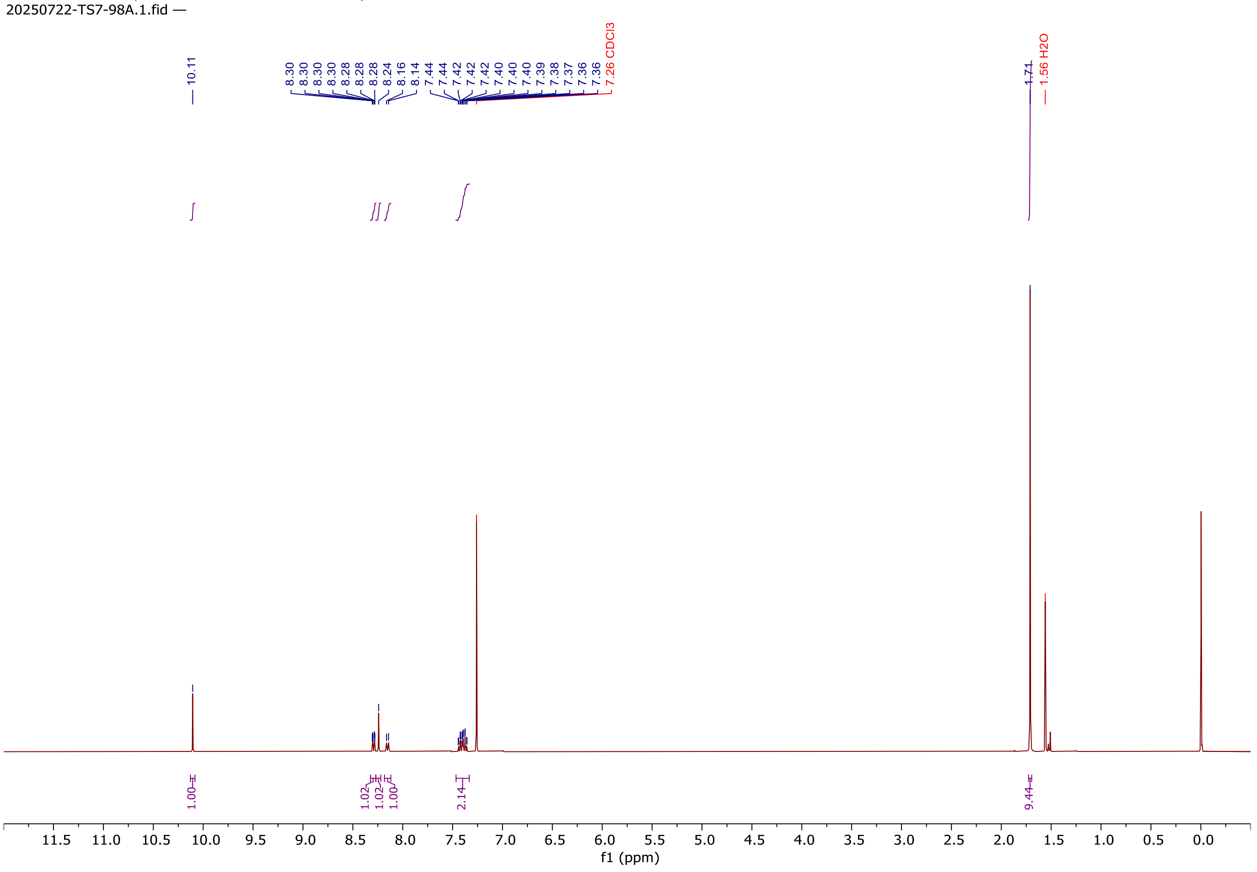

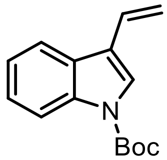

***tert*-Butyl 3-vinyl-1H-indole-1-carboxylate (SI-7)**

^{1}H NMR (400 MHz, $CDCl_3$)

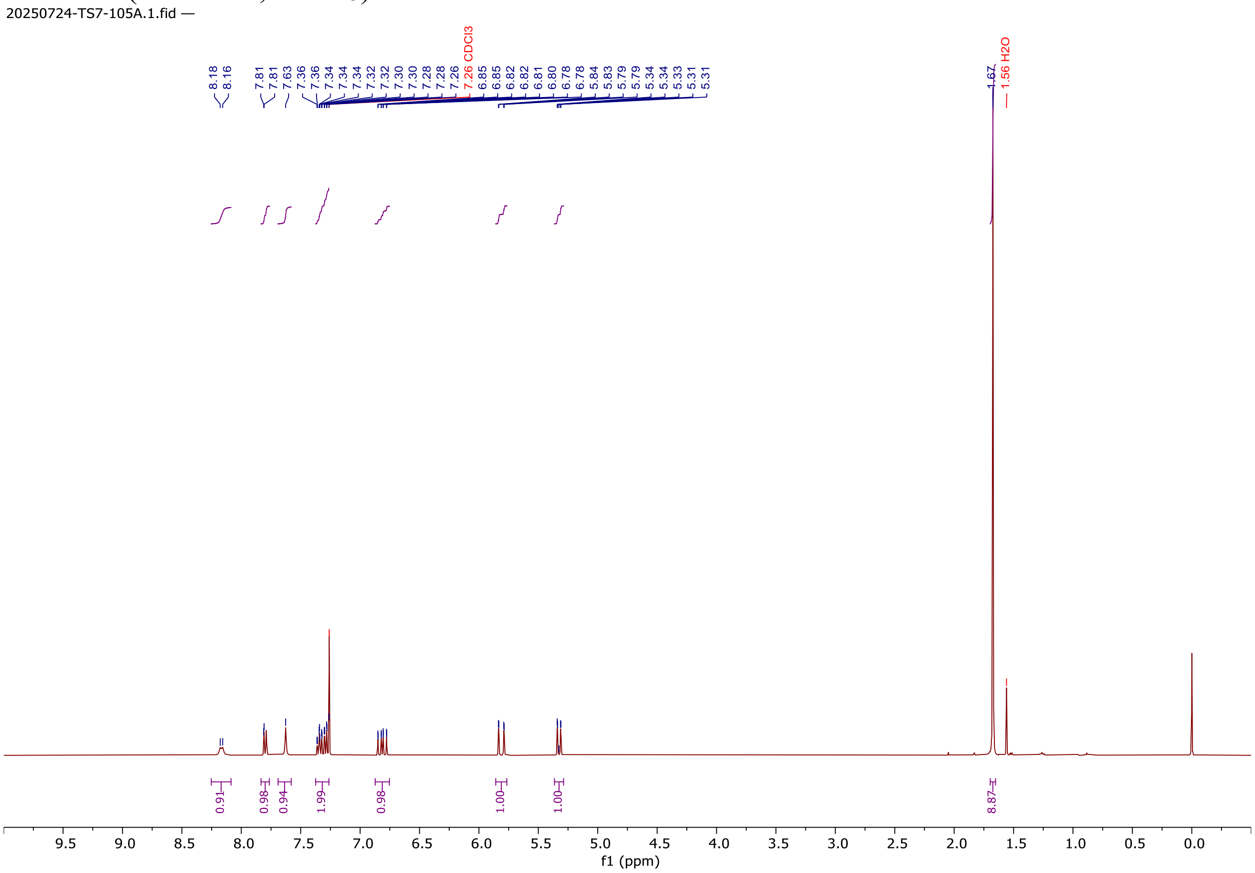

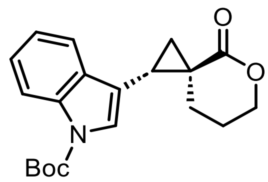

***tert*-Butyl 3-((1*S*,3*R*)-4-oxo-5-oxaspiro[2.5]octan-1-yl)-1H-indole-1-carboxylate (25a)**

^{1}H NMR (400 MHz, $CDCl_3$)

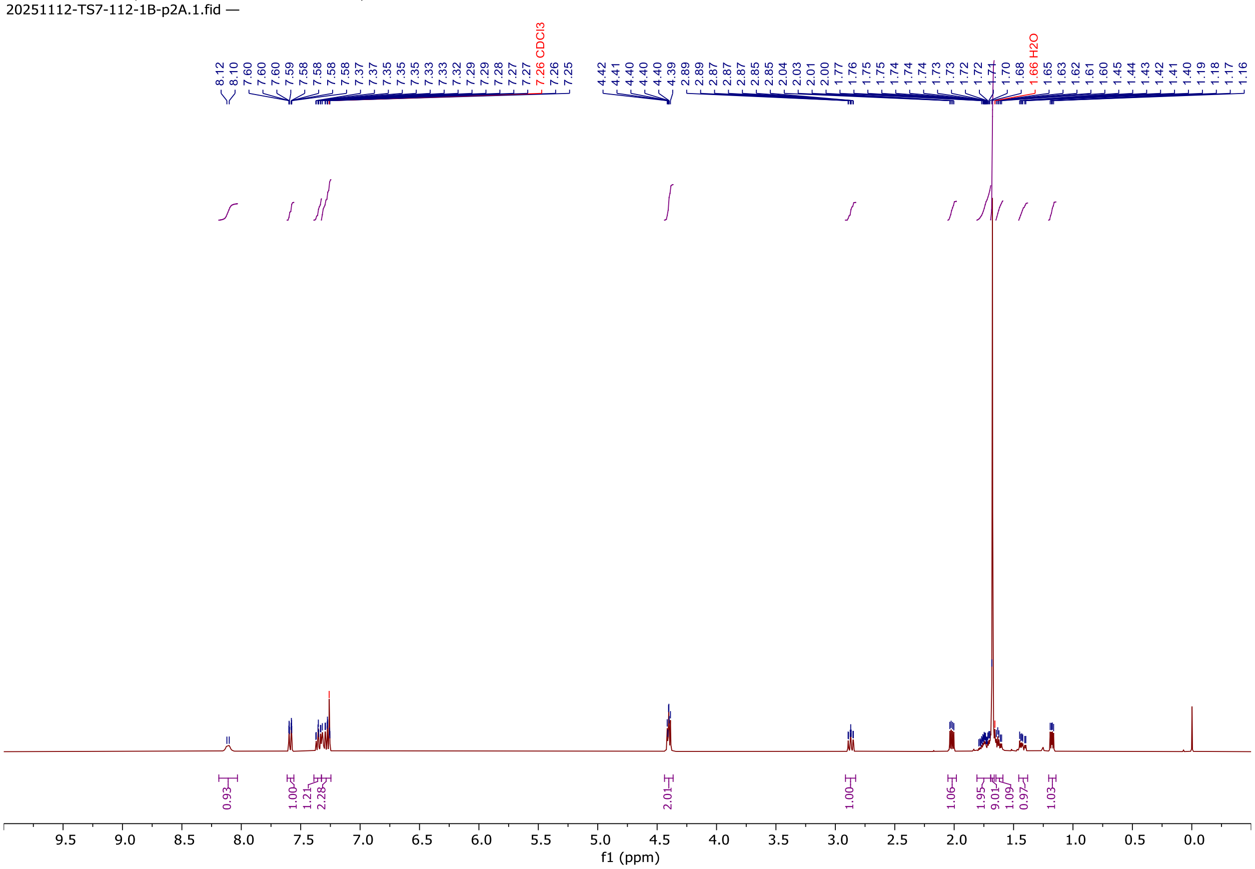

^{13}C NMR (101 MHz, $CDCl_3$)

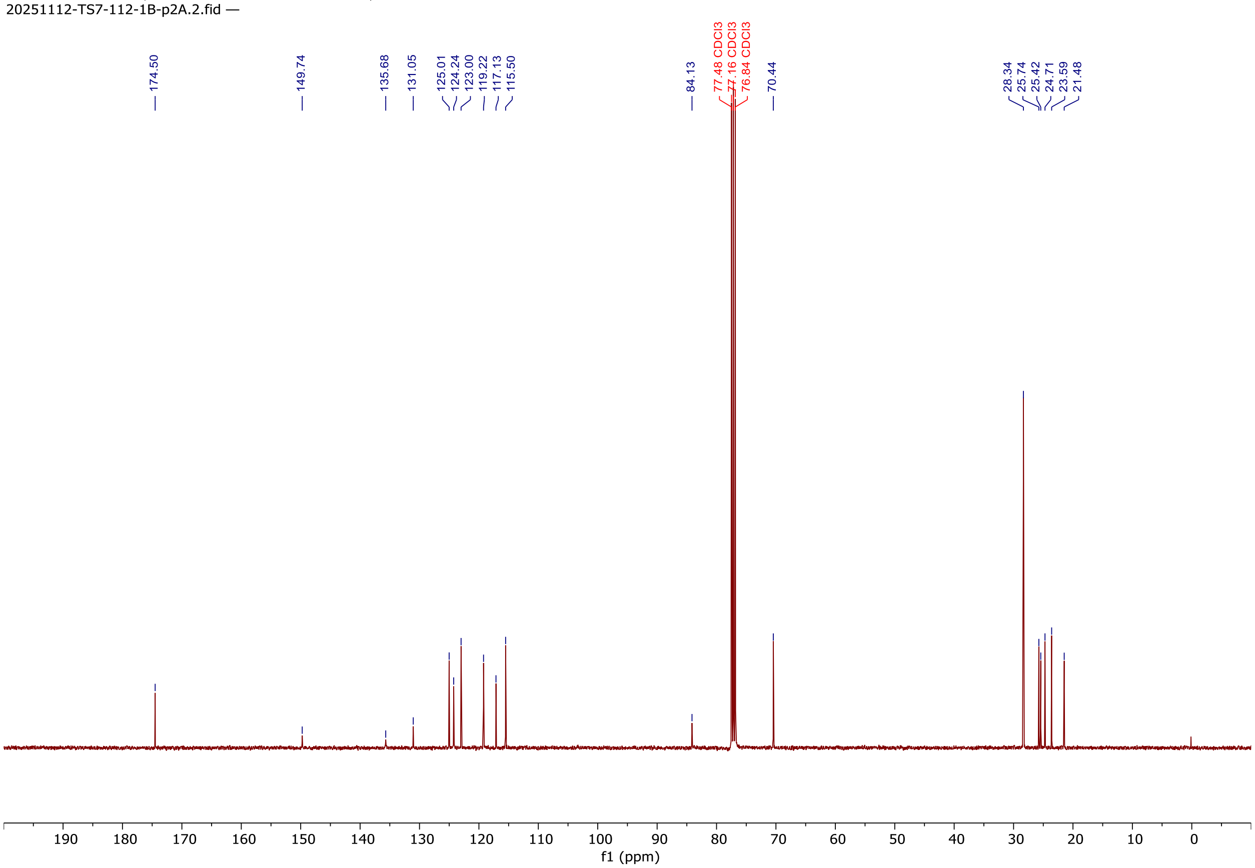

Chiral HPLC (210 nm trace):
Racemate, synthesized from $Rh_2(OAc)_4$

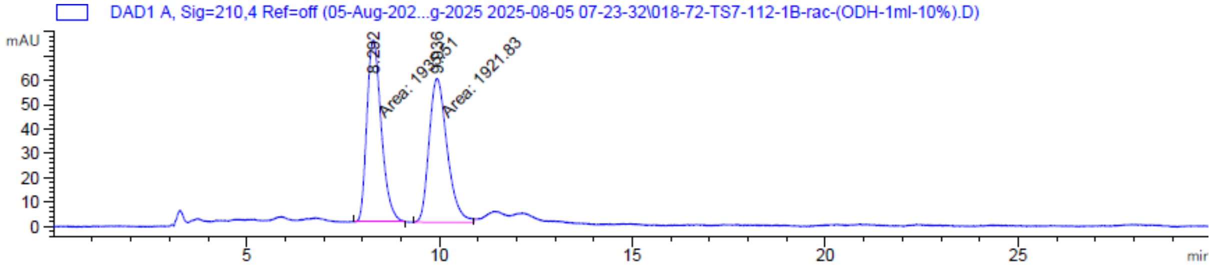

Signal 1: DAD1 A, Sig=210,4 Ref=off

Peak #	RetTime [min]	Type	Width [min]	Area [mAU*s]	Height [mAU]	Area %
1	8.292	MM	0.4322	1935.50586	74.64359	50.1773
2	9.936	MM	0.5424	1921.82971	59.04951	49.8227

Totals : 3857.33557 133.69311

Chiral, synthesized from Rh_2(*S*-*p*-PhTPCP)$_4$, 93% *ee*

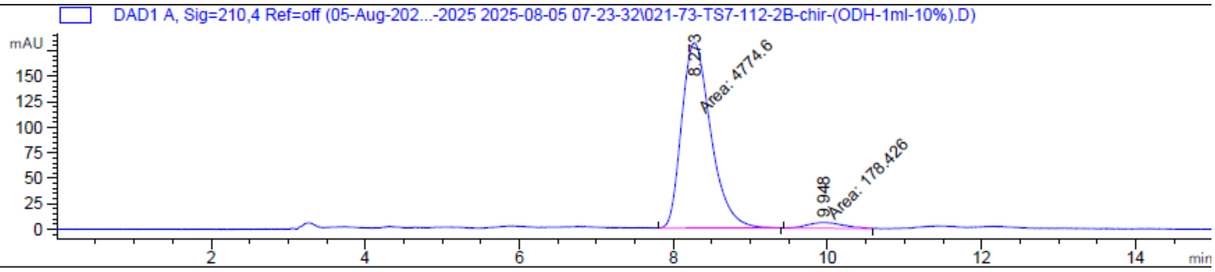

Signal 1: DAD1 A, Sig=210,4 Ref=off

Peak #	RetTime [min]	Type	Width [min]	Area [mAU*s]	Height [mAU]	Area %
1	8.273	MM	0.4376	4774.60107	181.85216	96.3976
2	9.948	MM	0.5192	178.42633	5.72736	3.6024

Totals : 4953.02740 187.57952

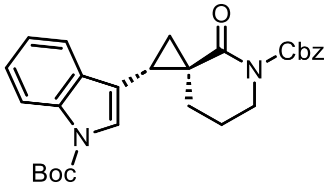

***tert*-Butyl 3-((1*S*,3*R*)-5-((benzyloxy)carbonyl)-4-oxo-5-azaspiro[2.5]octan-1-yl)-1H-indole-1-carboxylate (25b)**

^{1}H NMR (400 MHz, $CDCl_3$)

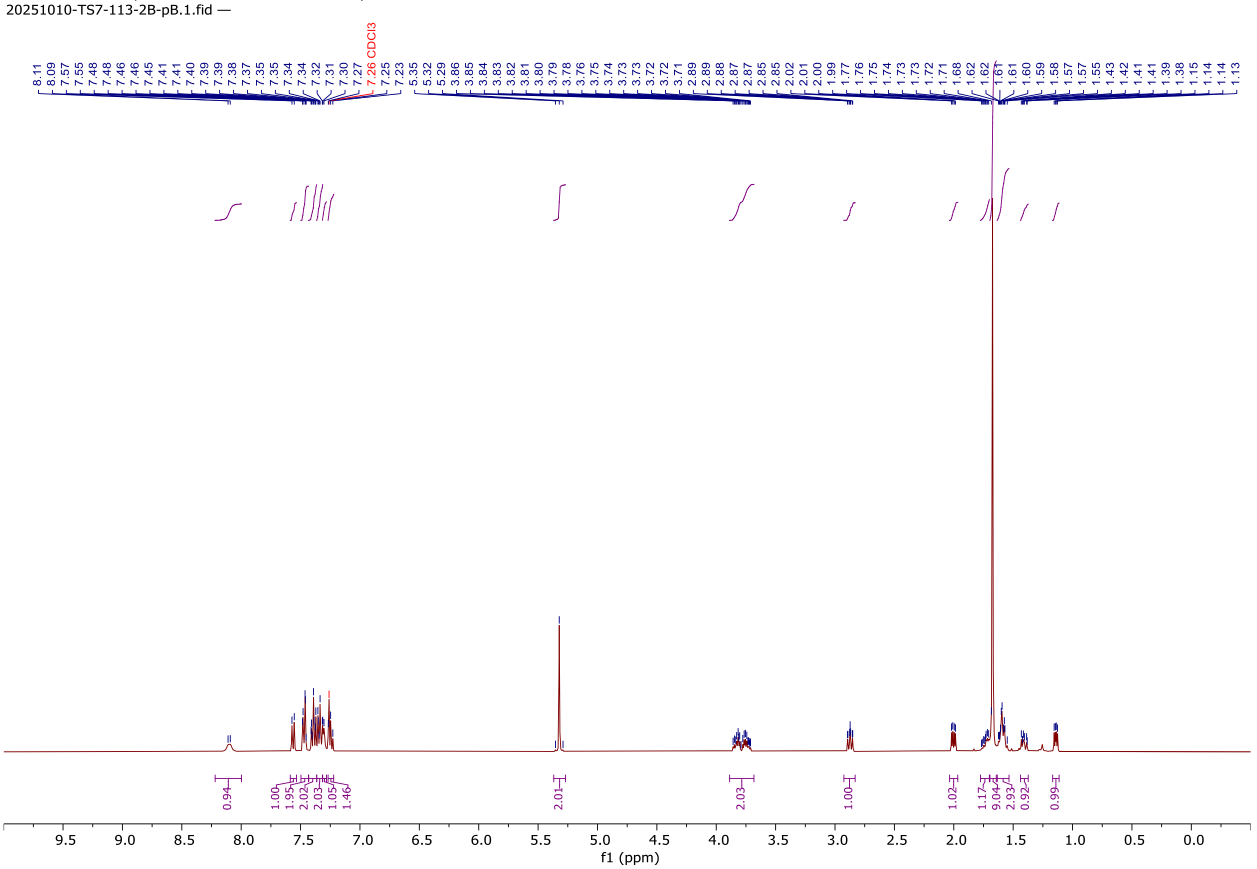

^{13}C NMR (101 MHz, $CDCl_3$)

One missing signal overlaps with another.

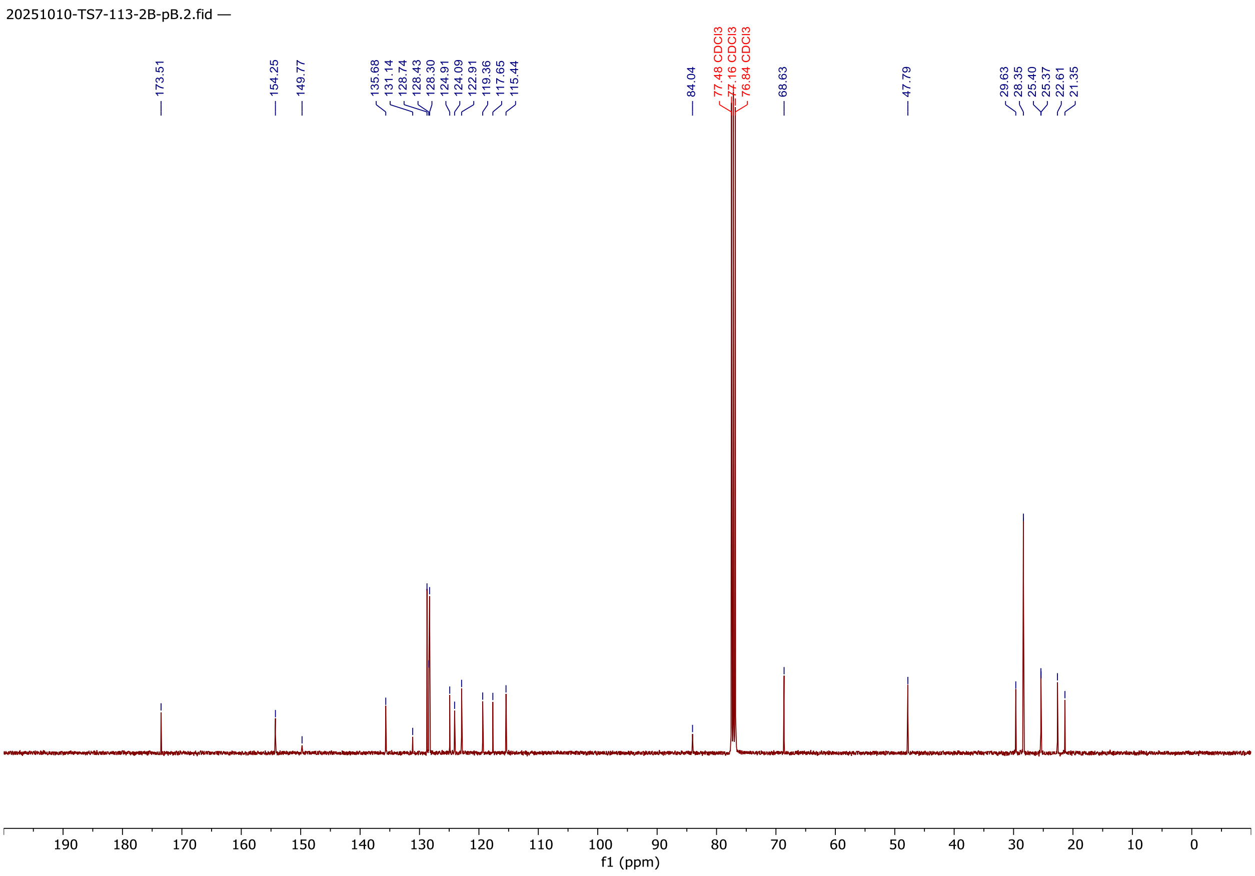

Chiral HPLC (210 nm trace):
Racemate, synthesized from $Rh_2(OAc)_4$

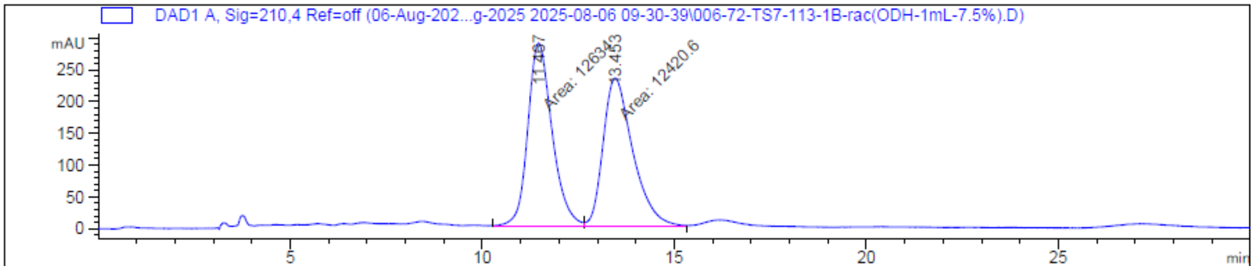

Signal 1: DAD1 A, Sig=210,4 Ref=off

Peak #	RetTime [min]	Type	Width [min]	Area [mAU*s]	Height [mAU]	Area %
1	11.467	MF	0.7310	1.26340e4	288.06686	50.4258
2	13.453	FM	0.8901	1.24206e4	232.57890	49.5742
Totals :				2.50546e4	520.64577	

Chiral, synthesized from Rh_2(*S*-*p*-PhTPCP)$_4$, 94% *ee*

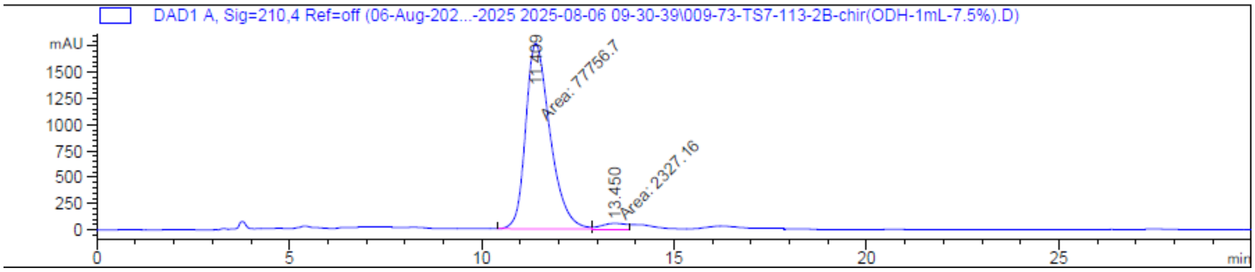

Signal 1: DAD1 A, Sig=210,4 Ref=off

Peak #	RetTime [min]	Type	Width [min]	Area [mAU*s]	Height [mAU]	Area %
1	11.409	MF	0.7336	7.77567e4	1766.65881	97.0941
2	13.450	MF	0.7317	2327.15576	53.00833	2.9059
Totals :				8.00839e4	1819.66714	

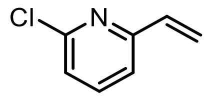

2-Chloro-6-vinylpyridine (SI-8)

^{1}H NMR (400 MHz, $CDCl_3$)

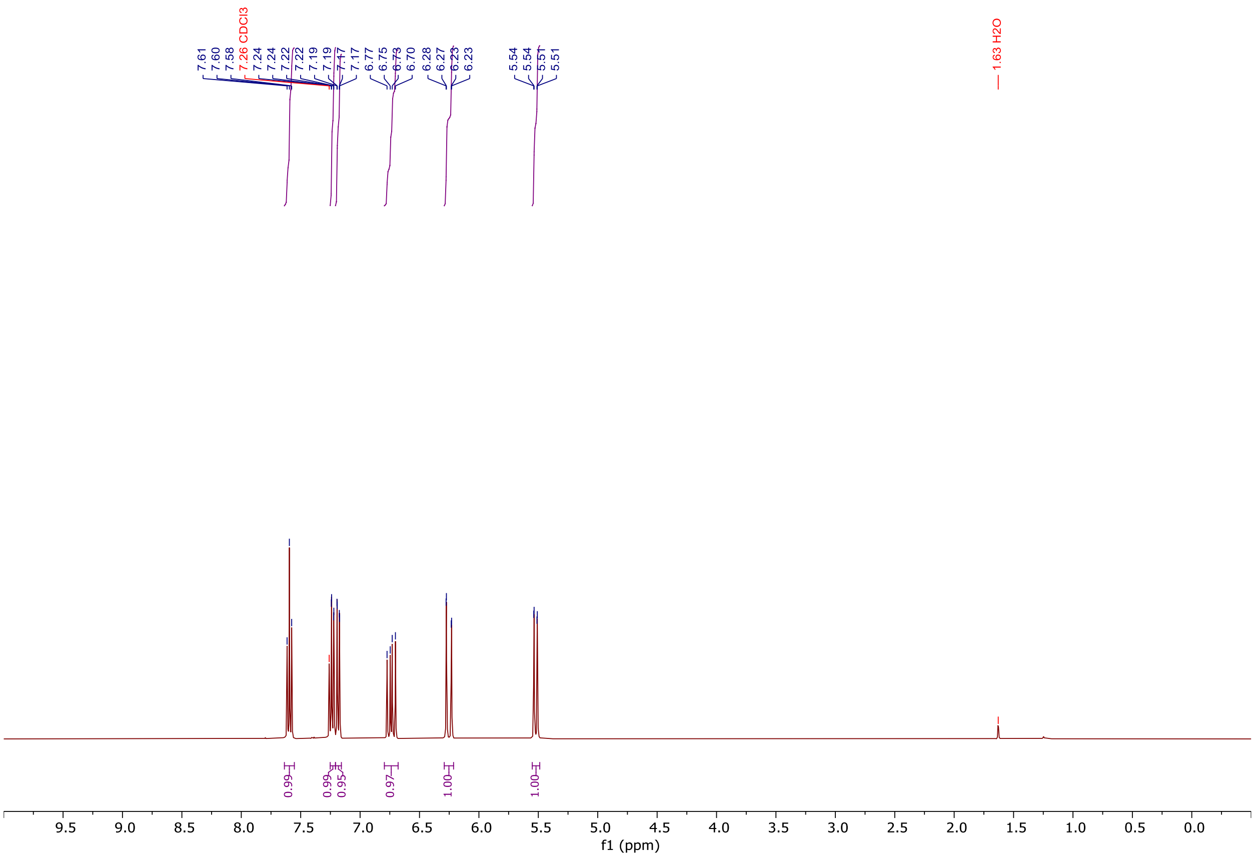

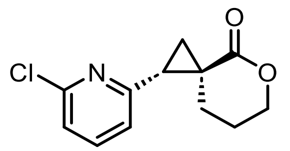

(1*R*,3*R*)-1-(6-chloropyridin-2-yl)-5-oxaspiro[2.5]octan-4-one (26a)

^{1}H NMR (400 MHz, $CDCl_3$)

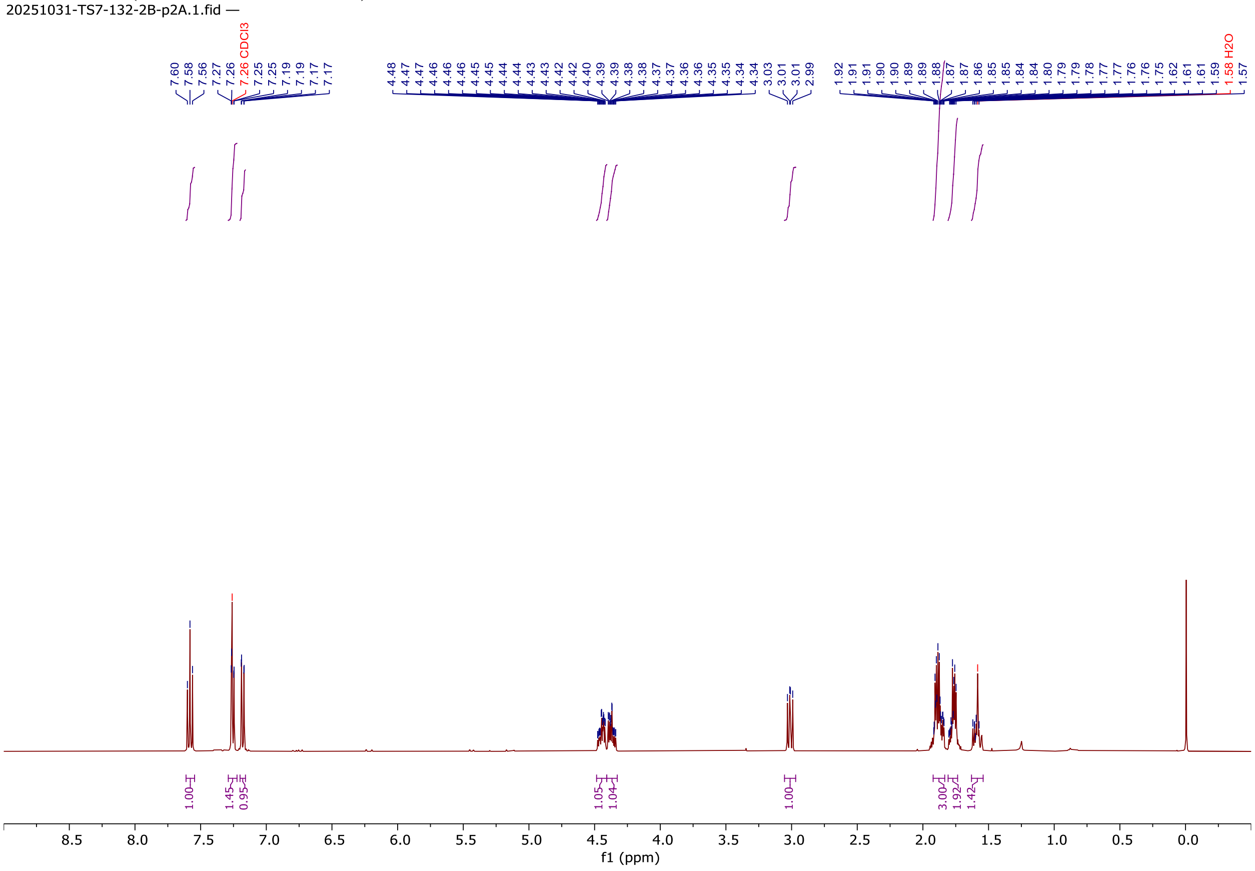

^{13}C NMR (101 MHz, $CDCl_3$)

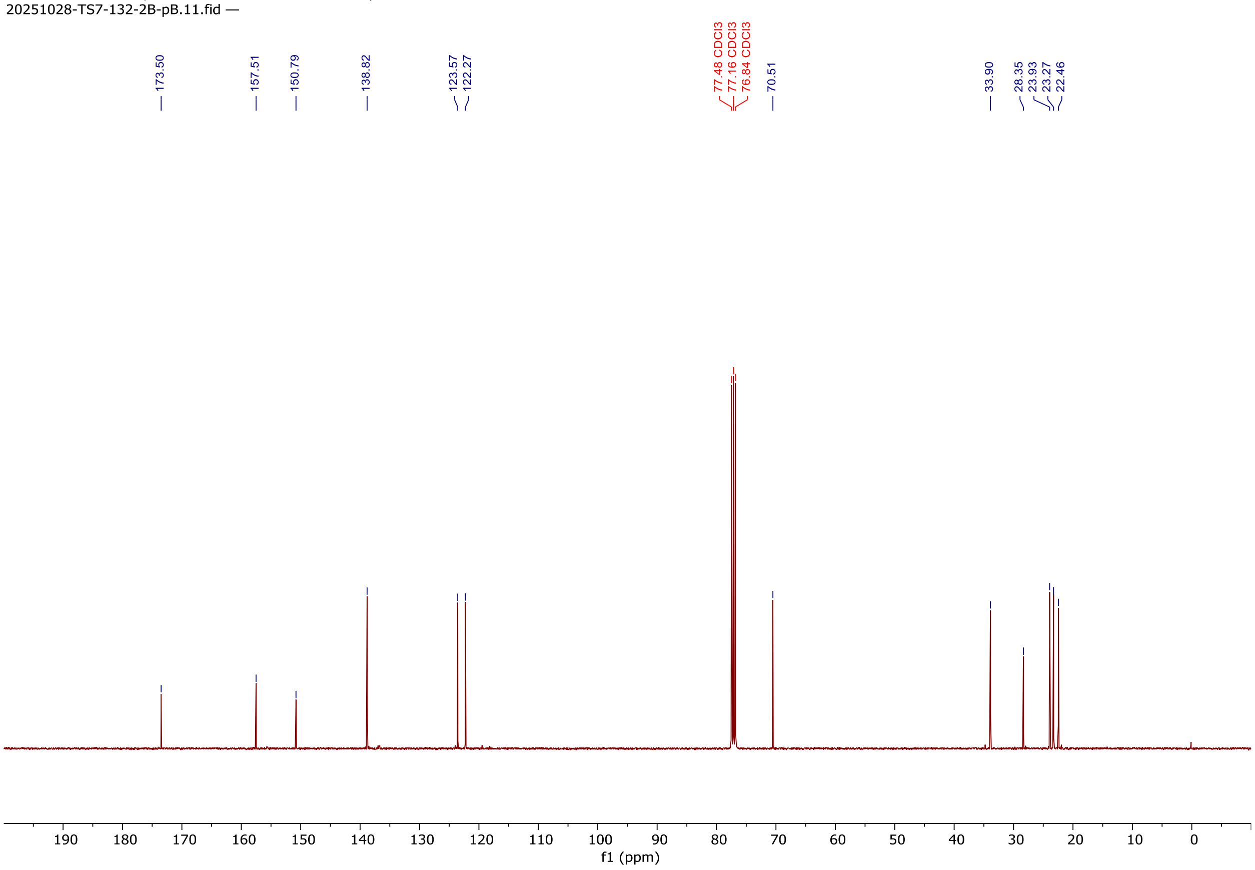

Chiral HPLC (210 nm trace):
Racemate, synthesized from $Rh_2(OAc)_4$

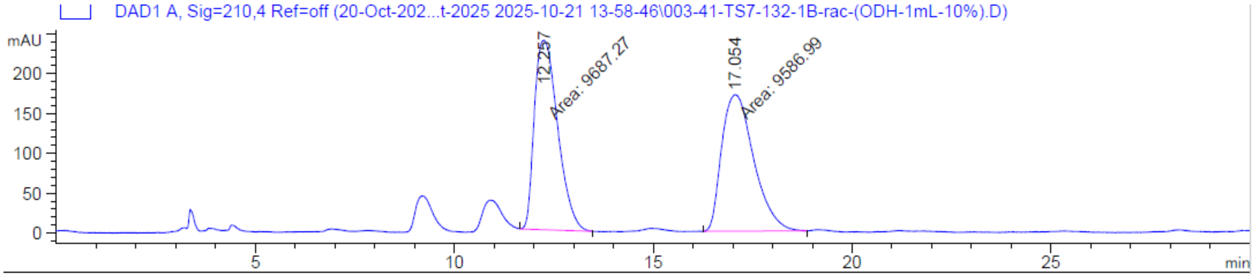

Signal 1: DAD1 A, Sig=210,4 Ref=off

Peak #	RetTime [min]	Type	Width [min]	Area [mAU*s]	Height [mAU]	Area %
1	12.257	MM	0.6794	9687.27344	237.62863	50.2602
2	17.054	MM	0.9343	9586.98633	171.01962	49.7398

Totals : 1.92743e4 408.64825

Chiral, synthesized from Rh_2(*S*-*p*-PhTPCP)$_4$, 83% *ee*

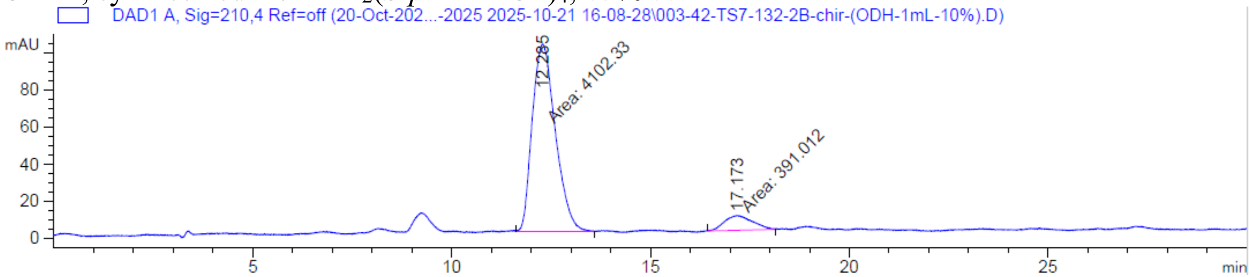

Signal 1: DAD1 A, Sig=210,4 Ref=off

Peak #	RetTime [min]	Type	Width [min]	Area [mAU*s]	Height [mAU]	Area %
1	12.285	MM	0.6752	4102.33154	101.26791	91.2980
2	17.173	MM	0.8056	391.01230	8.08996	8.7020

Totals : 4493.34384 109.35786

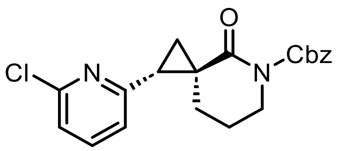

Benzyl (1*R*,3*R*)-1-(6-chloropyridin-2-yl)-4-oxo-5-azaspiro[2.5]octane-5-carboxylate (26b)

^{1}H NMR (400 MHz, $CDCl_3$)

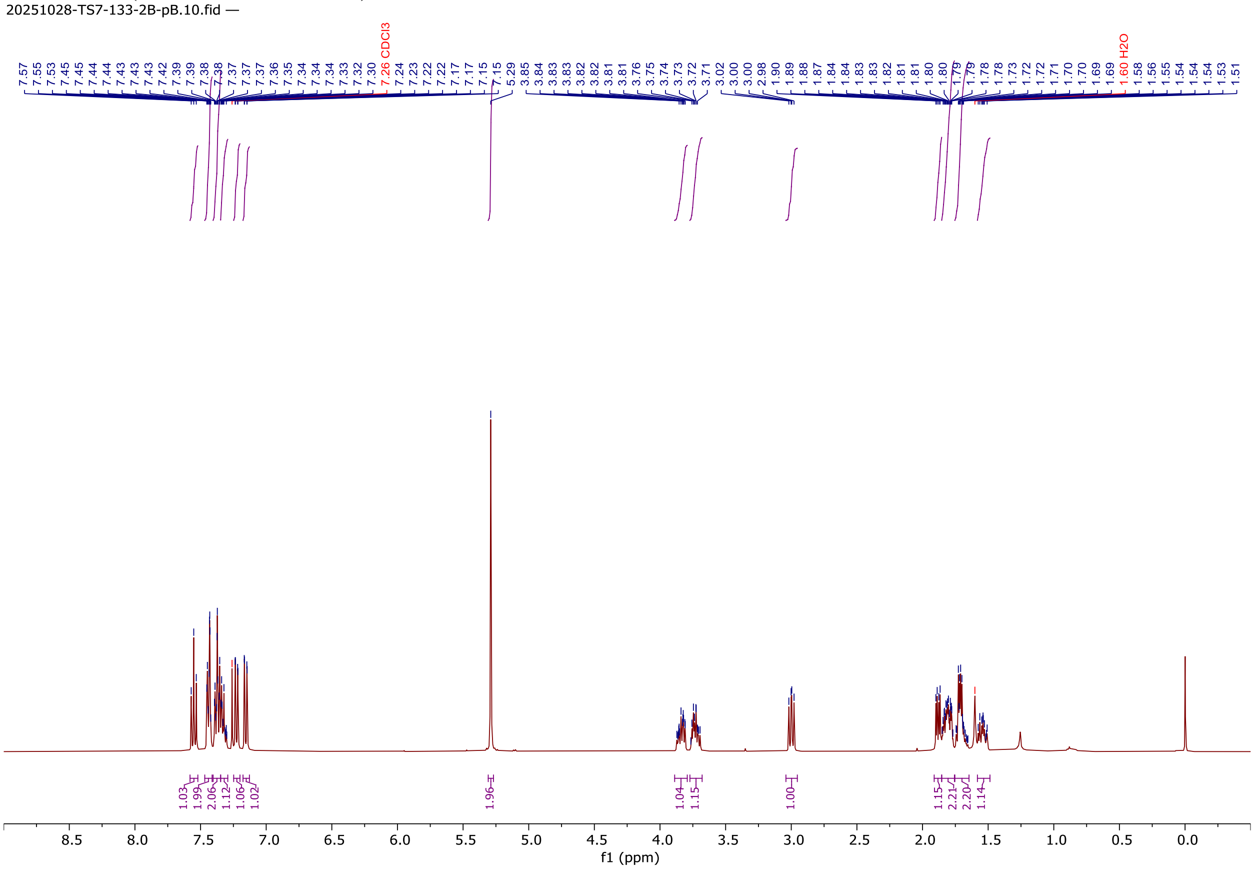

^{13}C NMR (101 MHz, $CDCl_3$)

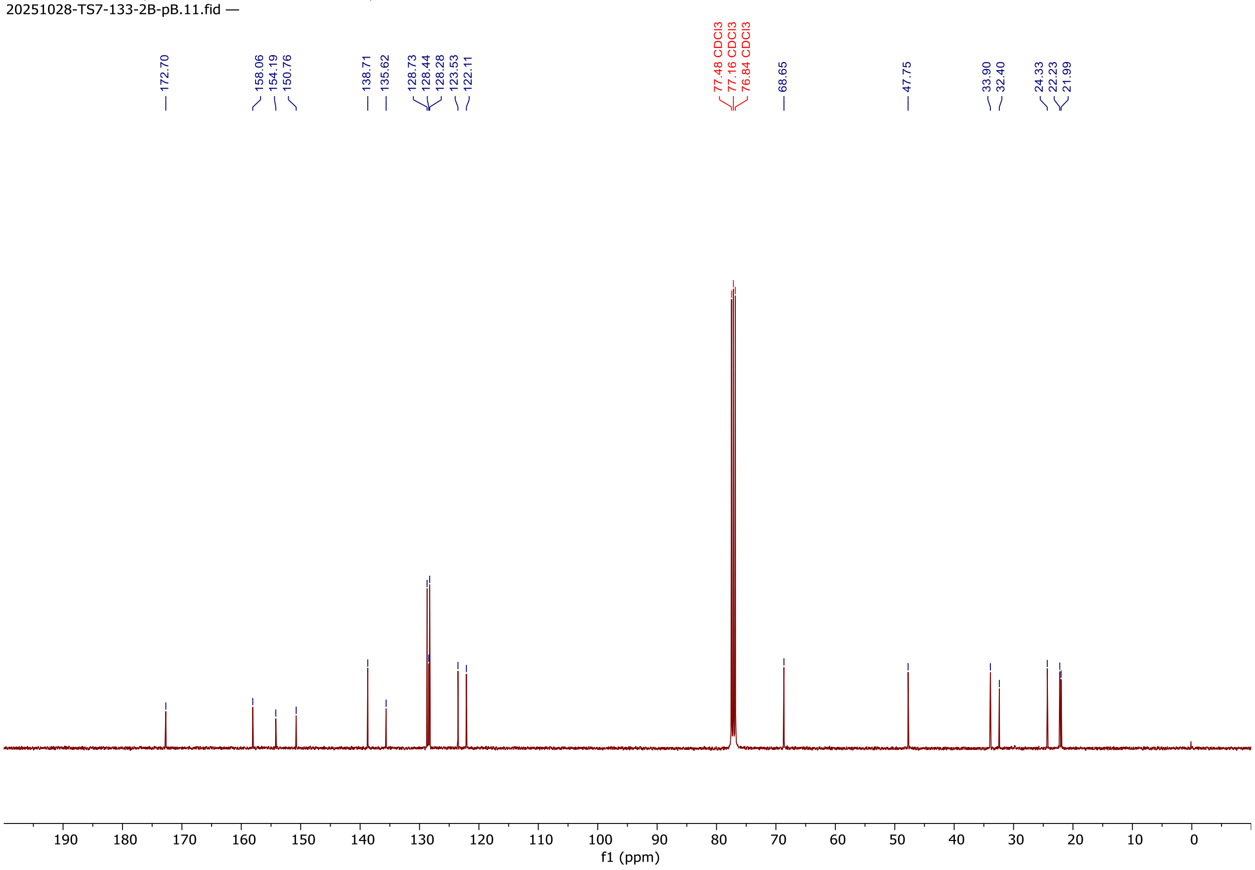

Chiral HPLC (210 nm trace):
Racemate, synthesized from $Rh_2(OAc)_4$

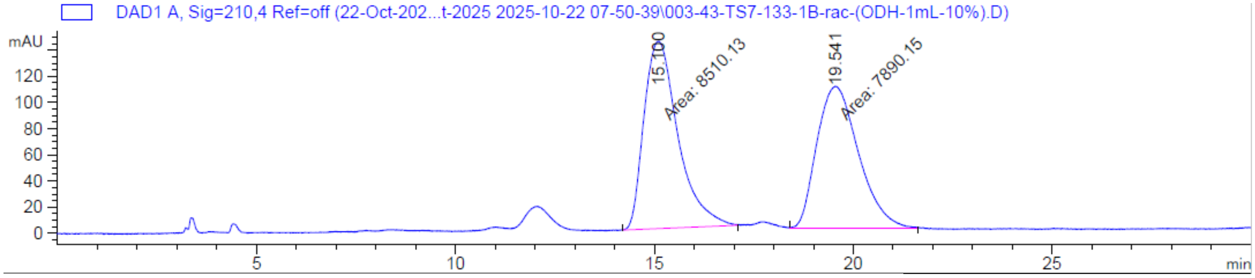

Signal 1: DAD1 A, Sig=210,4 Ref=off

Peak #	RetTime [min]	Type	Width [min]	Area [mAU*s]	Height [mAU]	Area %
1	15.100	MM	0.9895	8510.12988	143.34056	51.8902
2	19.541	MM	1.2132	7890.14893	108.39684	48.1098

Totals : 1.64003e4 251.73740

Chiral, synthesized from Rh_2(*S*-*p*-PhTPCP)$_4$, 92% *ee*

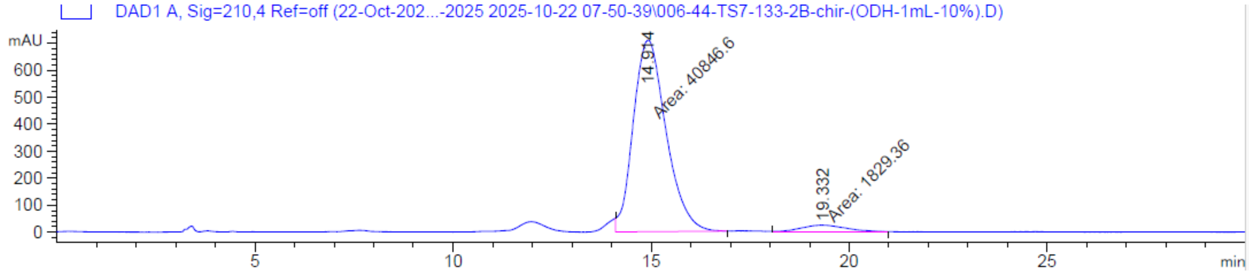

Signal 1: DAD1 A, Sig=210,4 Ref=off

Peak #	RetTime [min]	Type	Width [min]	Area [mAU*s]	Height [mAU]	Area %
1	14.914	FM	0.9583	4.08466e4	710.36591	95.7134
2	19.332	MM	1.2715	1829.36157	23.97888	4.2866

Totals : 4.26759e4 734.34479

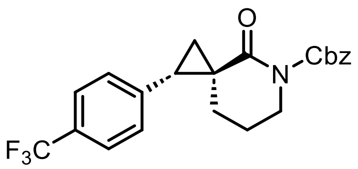

Benzyl (1*S*,3*R*)-4-oxo-1-(4-(trifluoromethyl)phenyl)-5-azaspiro[2.5]octane-5-carboxylate (27b)

^{1}H NMR (400 MHz, $CDCl_3$)

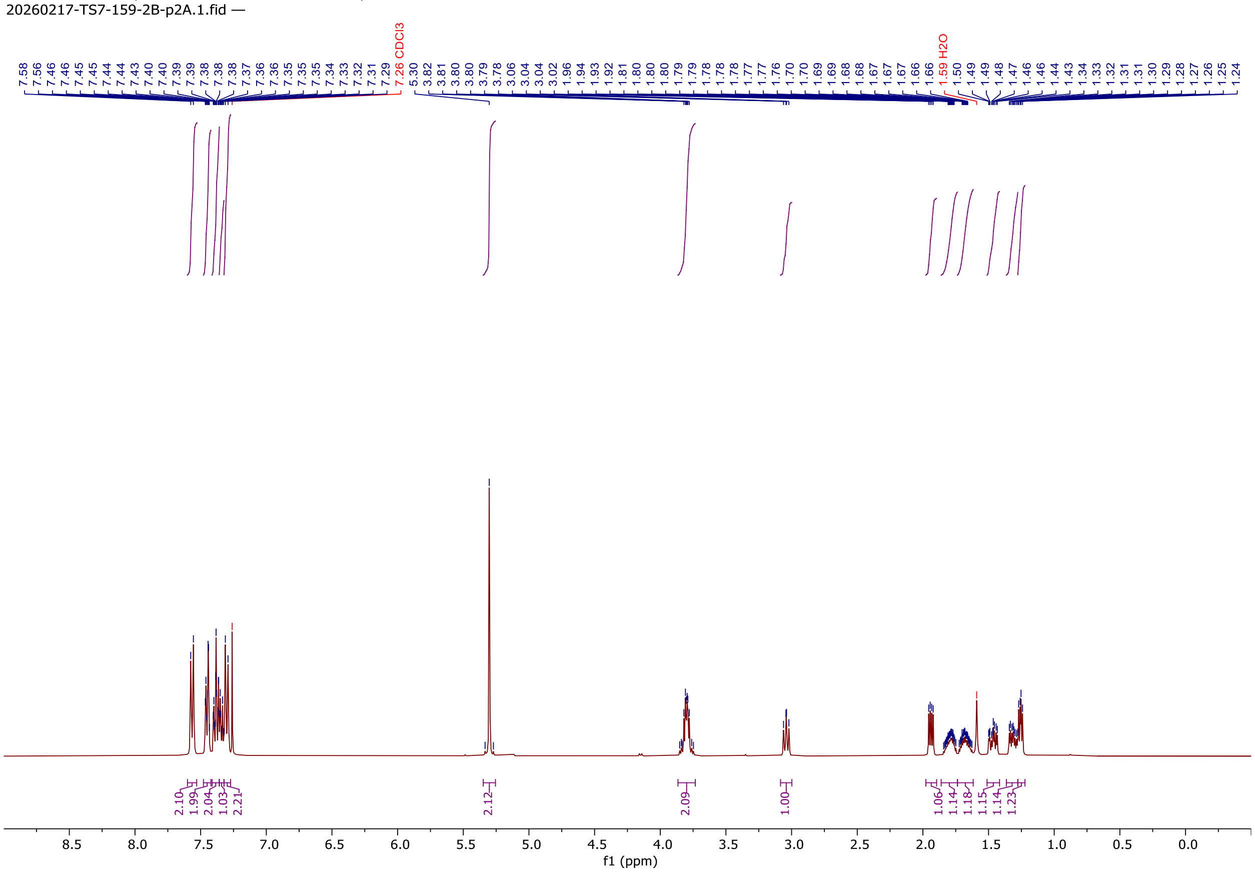

^{19}F NMR (376 MHz, $CDCl_3$)

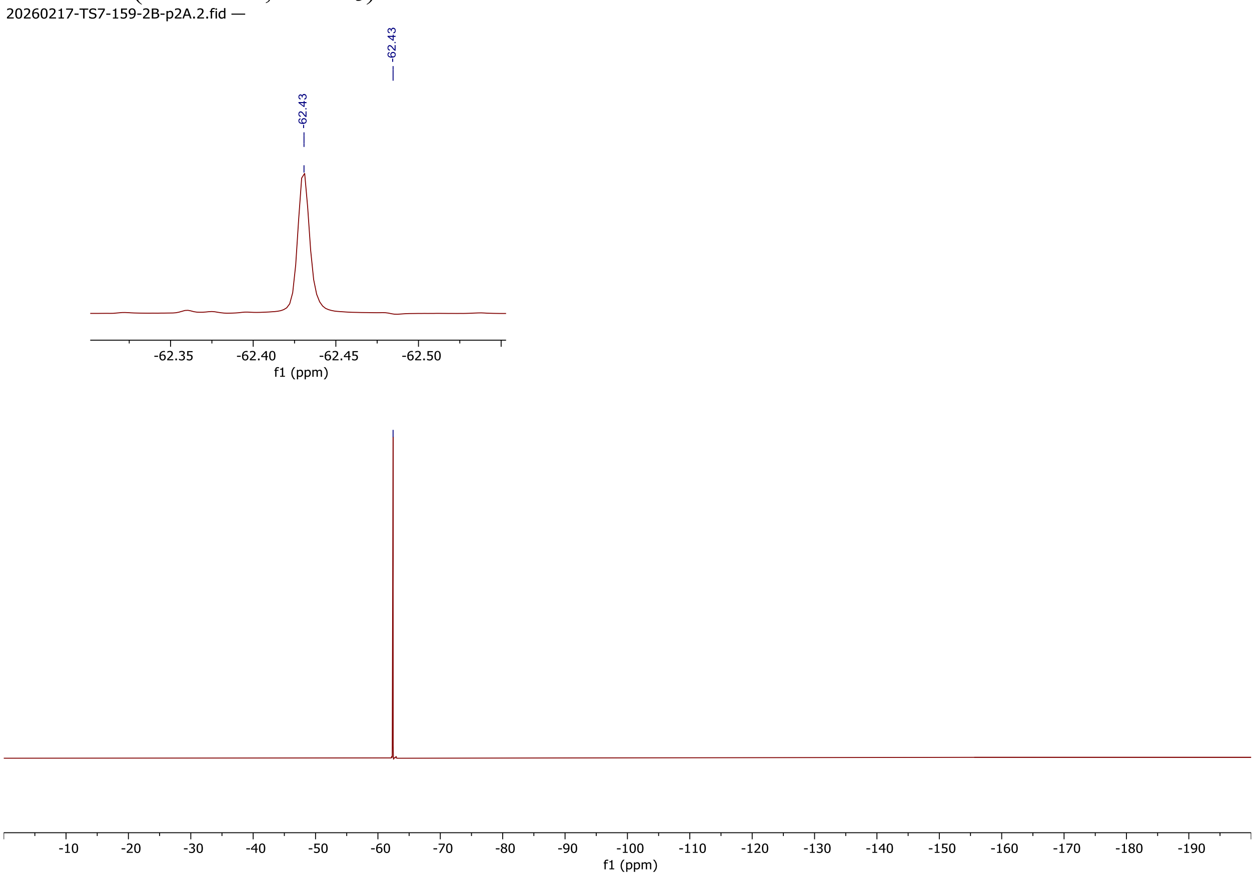

^{13}C NMR (101 MHz, $CDCl_3$)

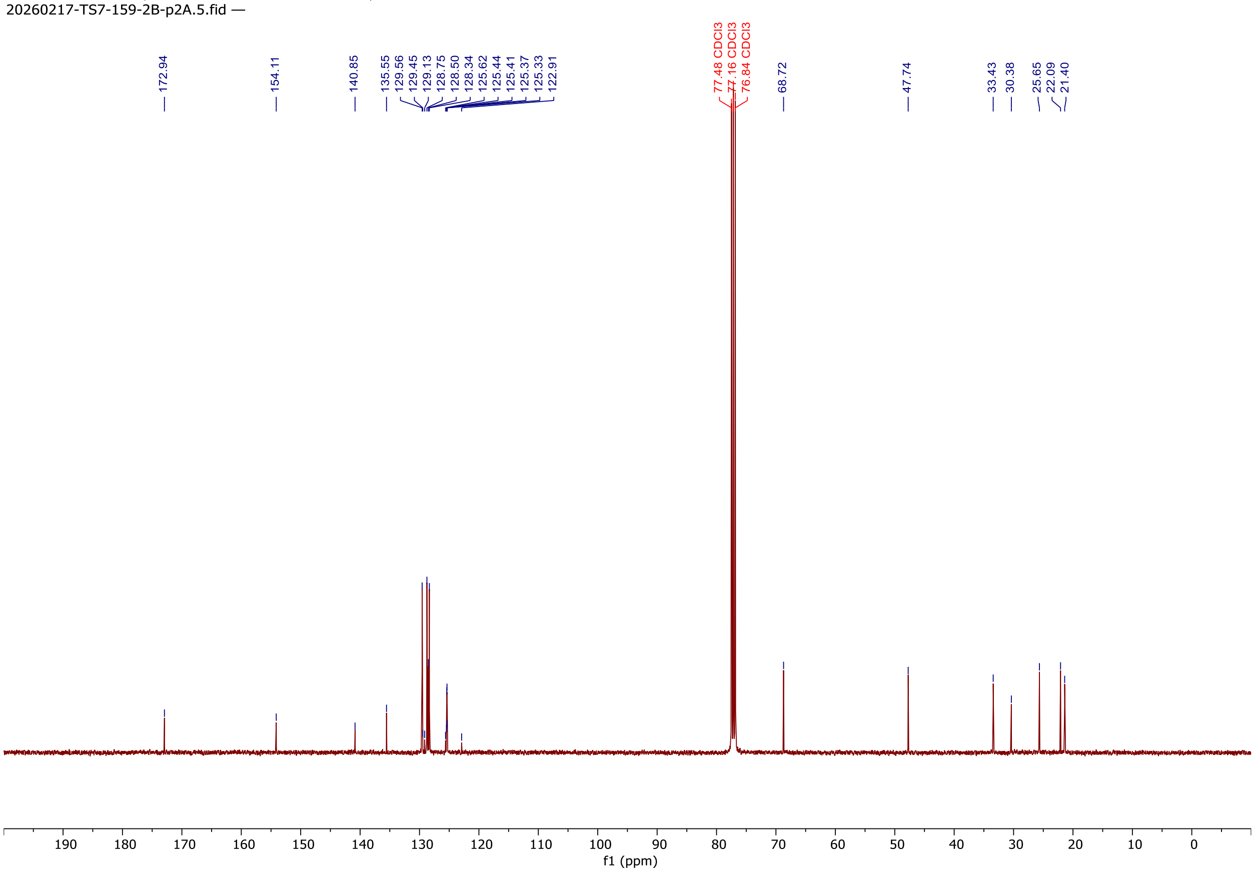

Chiral HPLC (210 nm trace):
Racemate, synthesized from $Rh_2(OAc)_4$

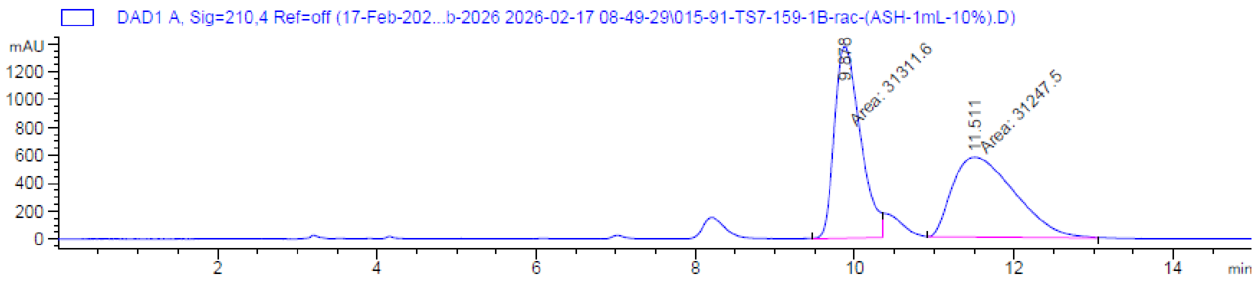


```
Signal 1: DAD1 A, Sig=210,4 Ref=off

Peak RetTime Type  Width     Area      Height     Area
  #   [min]        [min]   [mAU*s]     [mAU]       %
----|-------|----|-------|----------|----------|--------|
   1   9.878 MF    0.3783 3.13116e4 1379.44348  50.0513
   2  11.511 MM    0.9060 3.12475e4  574.80865  49.9487

Totals :                  6.25591e4 1954.25214
```

Chiral, synthesized from Rh_2(*S*-*p*-PhTPCP)$_4$, 96% *ee*

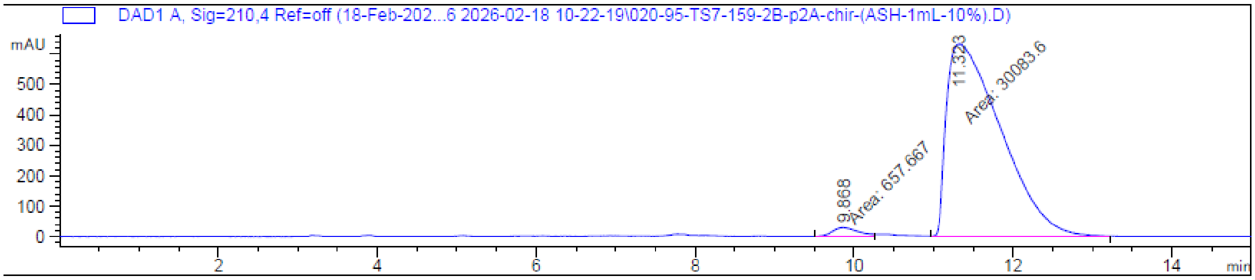


```
Signal 1: DAD1 A, Sig=210,4 Ref=off

Peak RetTime Type  Width     Area      Height     Area
  #   [min]        [min]   [mAU*s]     [mAU]       %
----|-------|----|-------|----------|----------|--------|
   1   9.868 MF    0.3769  657.66705   29.08508   2.1394
   2  11.323 MM    0.7931 3.00836e4  632.23248  97.8606

Totals :                  3.07413e4  661.31756
```

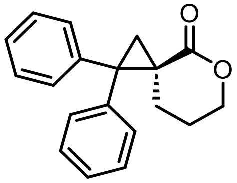

(*R*)-1,1-diphenyl-5-oxaspiro[2.5]octan-4-one (28a)

^{1}H NMR (400 MHz, $CDCl_3$)

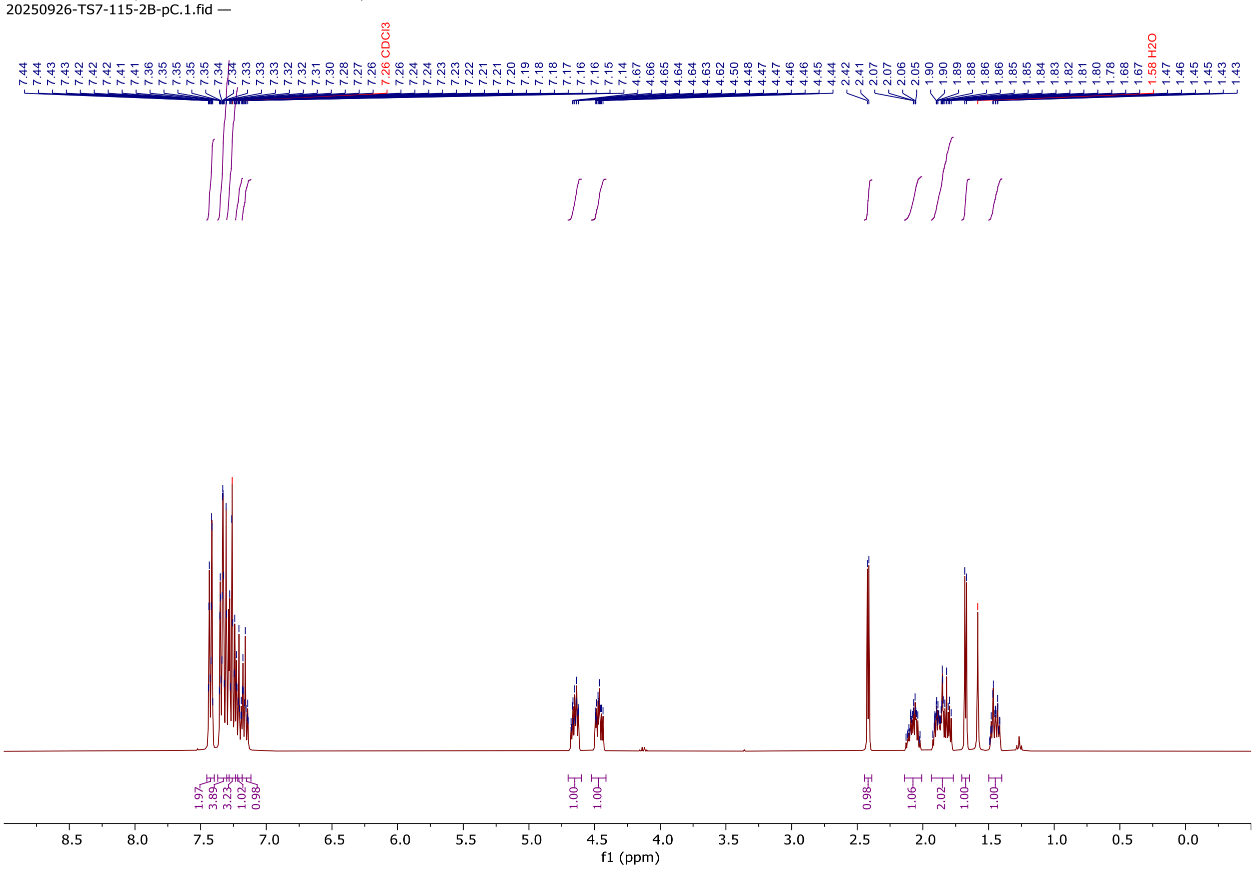

^{13}C NMR (101 MHz, $CDCl_3$)

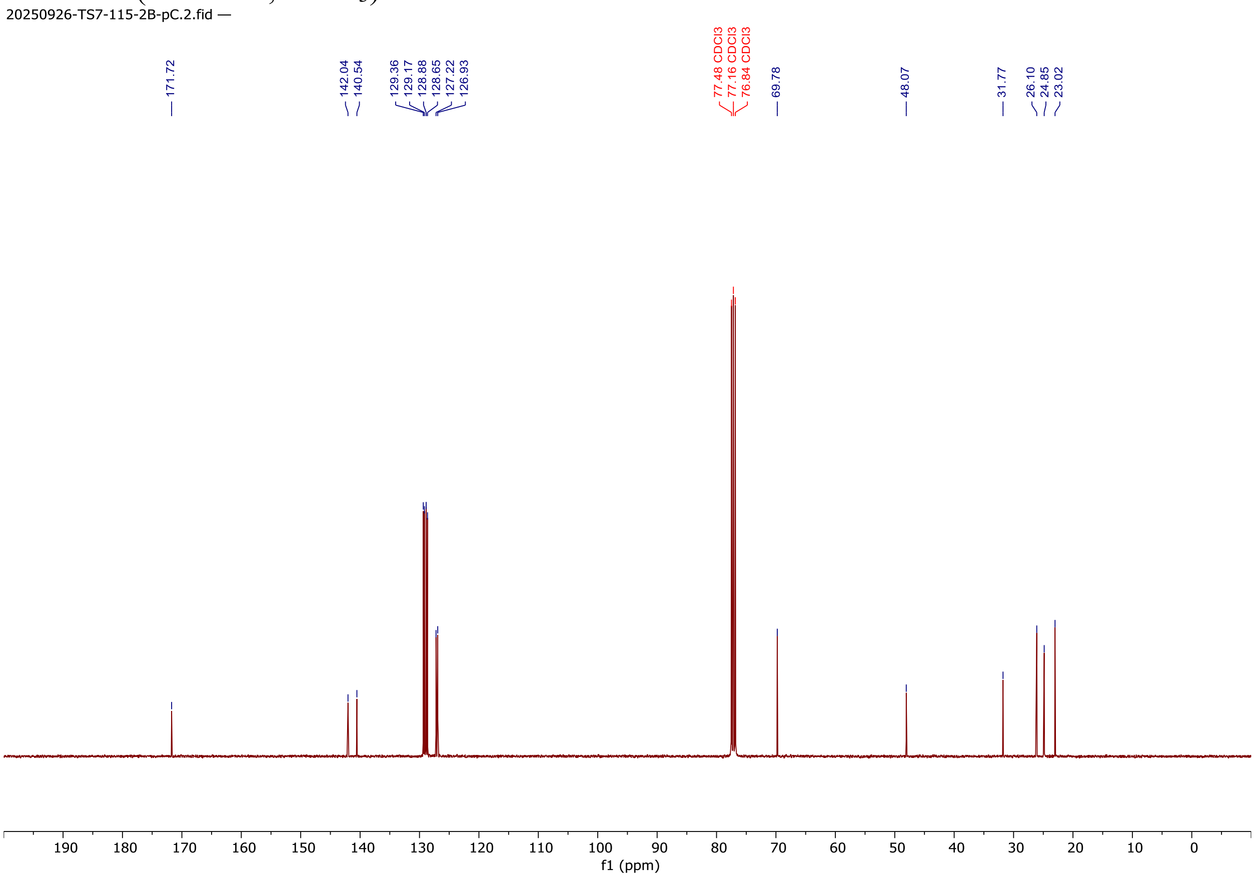

Chiral HPLC (210 nm trace):
Racemate, synthesized from $Rh_2(OAc)_4$

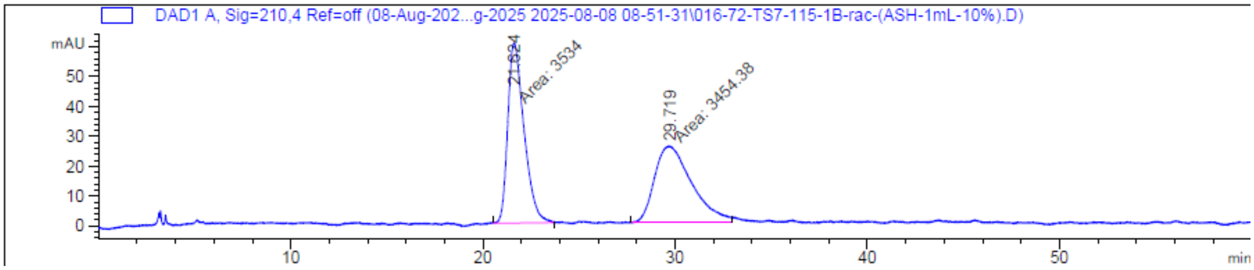

Signal 1: DAD1 A, Sig=210,4 Ref=off

Peak #	RetTime [min]	Type	Width [min]	Area [mAU*s]	Height [mAU]	Area %
1	21.624	MM	0.9768	3534.00024	60.29808	50.5697
2	29.719	MM	2.2410	3454.37744	25.69024	49.4303
Totals :				6988.37769	85.98831	

Chiral, synthesized from Rh_2(*S*-*p*-PhTPCP)$_4$, 90% *ee*

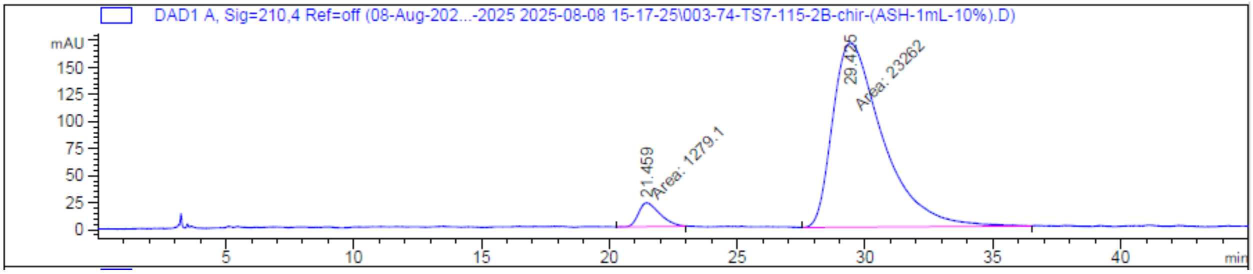

Signal 1: DAD1 A, Sig=210,4 Ref=off

Peak #	RetTime [min]	Type	Width [min]	Area [mAU*s]	Height [mAU]	Area %
1	21.459	MM	0.9593	1279.10364	22.22196	5.2121
2	29.425	MM	2.2757	2.32620e4	170.36620	94.7879
Totals :				2.45411e4	192.58816	

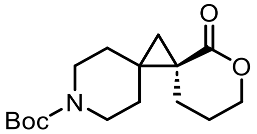

***tert*-Butyl (*R*)-1-oxo-2-oxa-10-azadispiro[5.0.5^{7}.1^{6}]tridecane-10-carboxylate (29a)**

^{1}H NMR (400 MHz, $CDCl_3$)

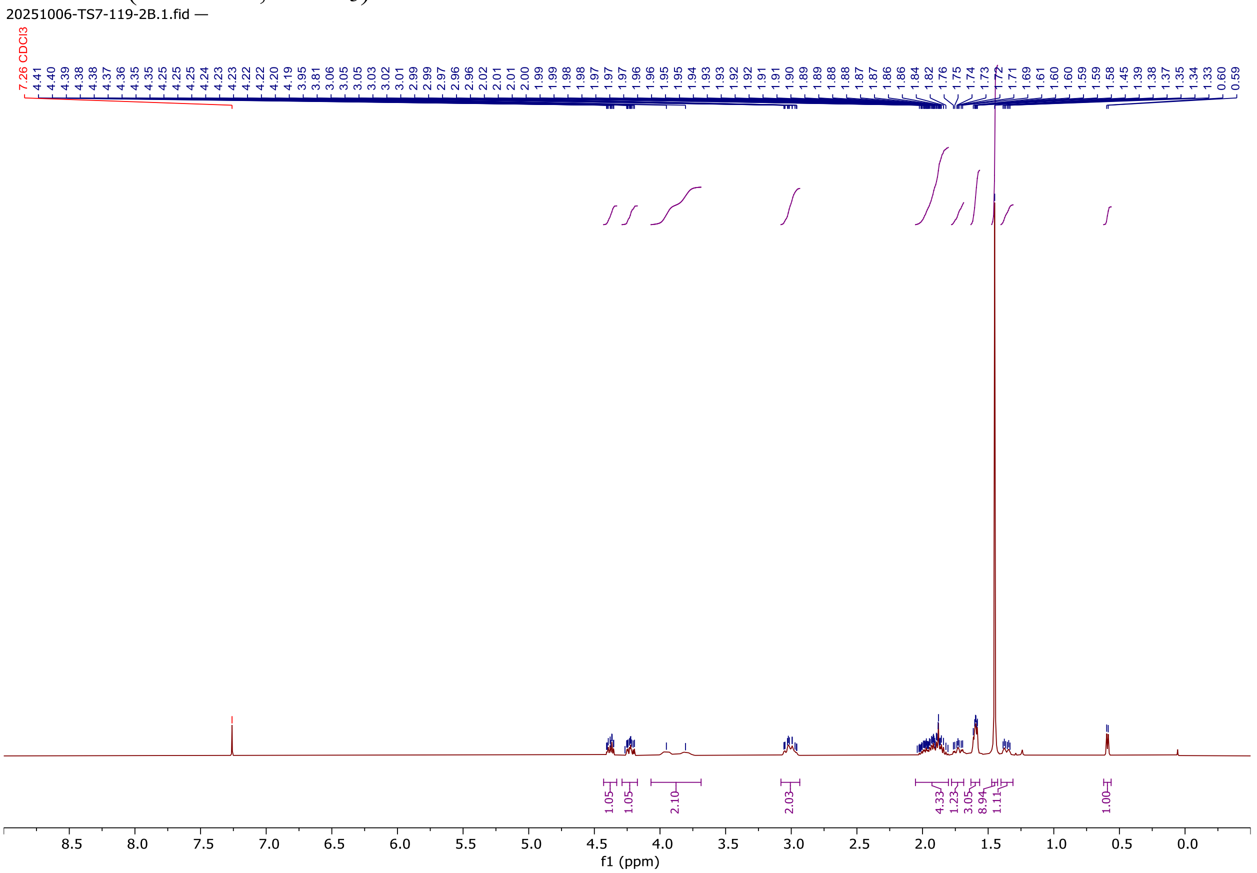

^{13}C NMR (101 MHz, $CDCl_3$)

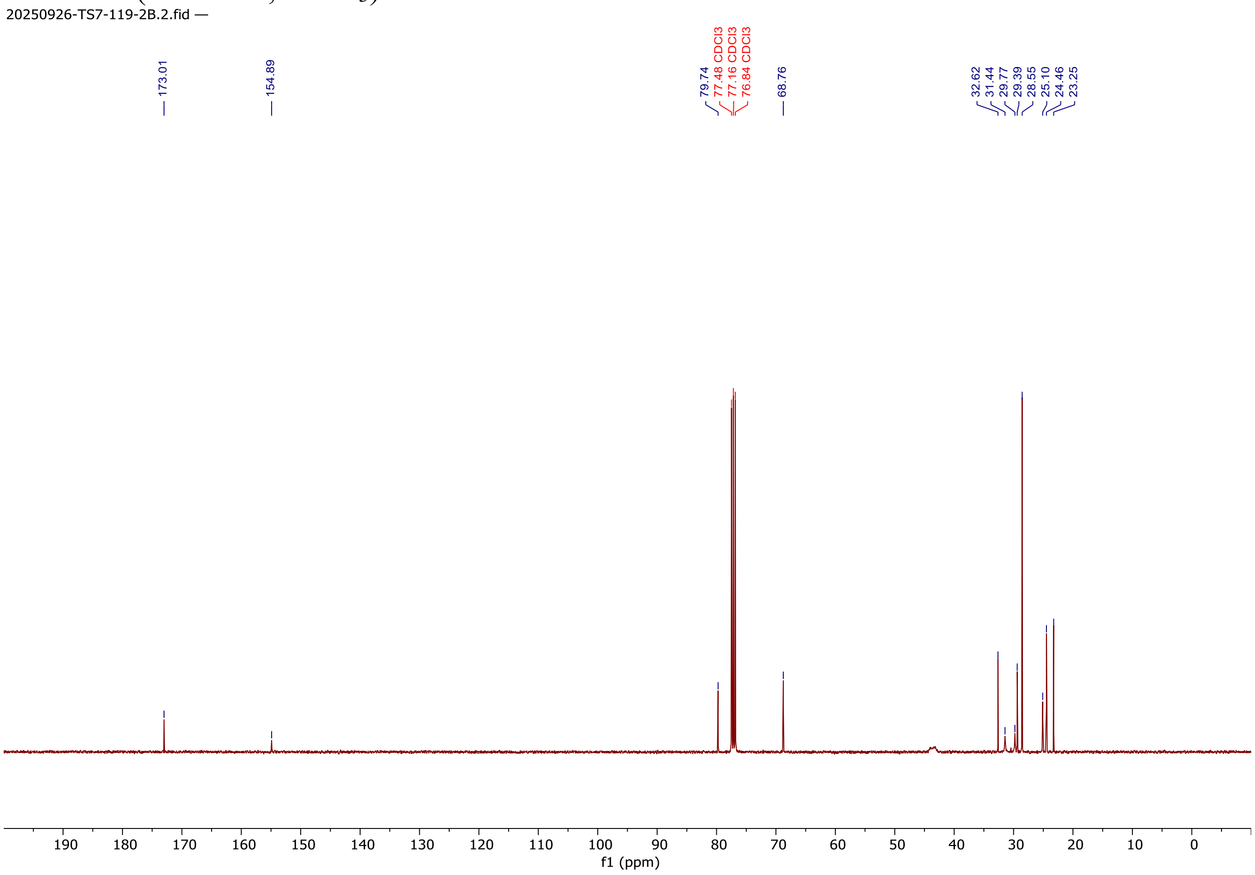

Chiral HPLC (210 nm trace):
Racemate, synthesized from $Rh_2(OAc)_4$

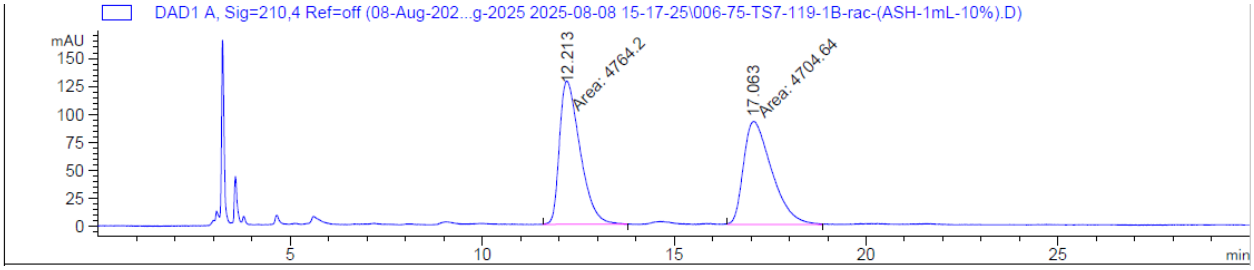

Signal 1: DAD1 A, Sig=210,4 Ref=off

Peak #	RetTime [min]	Type	Width [min]	Area [mAU*s]	Height [mAU]	Area %
1	12.213	MM	0.6200	4764.20264	128.07446	50.3145
2	17.063	MM	0.8517	4704.64063	92.06358	49.6855
Totals :				9468.84326	220.13804	

Chiral, synthesized from Rh_2(*S*-*p*-PhTPCP)$_4$, 77% *ee*

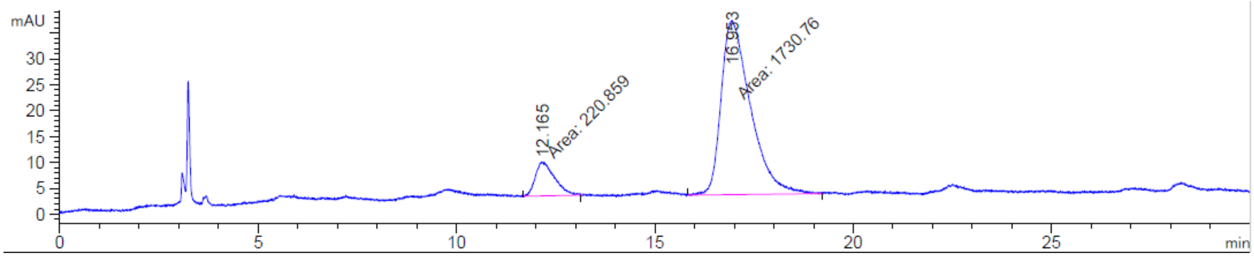

Signal 1: DAD1 A, Sig=210,4 Ref=off

Peak #	RetTime [min]	Type	Width [min]	Area [mAU*s]	Height [mAU]	Area %
1	12.165	MM	0.5585	220.85860	6.59052	11.3167
2	16.953	MM	0.8574	1730.76147	33.64433	88.6833
Totals :				1951.62007	40.23485	

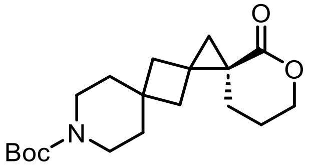

***tert*-Butyl (*R*)-1-oxo-2-oxa-12-azatrispiro[5.0.1.5^{9}.1^{7}.1^{6}]hexadecane-12-carboxylate** (30a)

^{1}H NMR (400 MHz, $CDCl_3$)

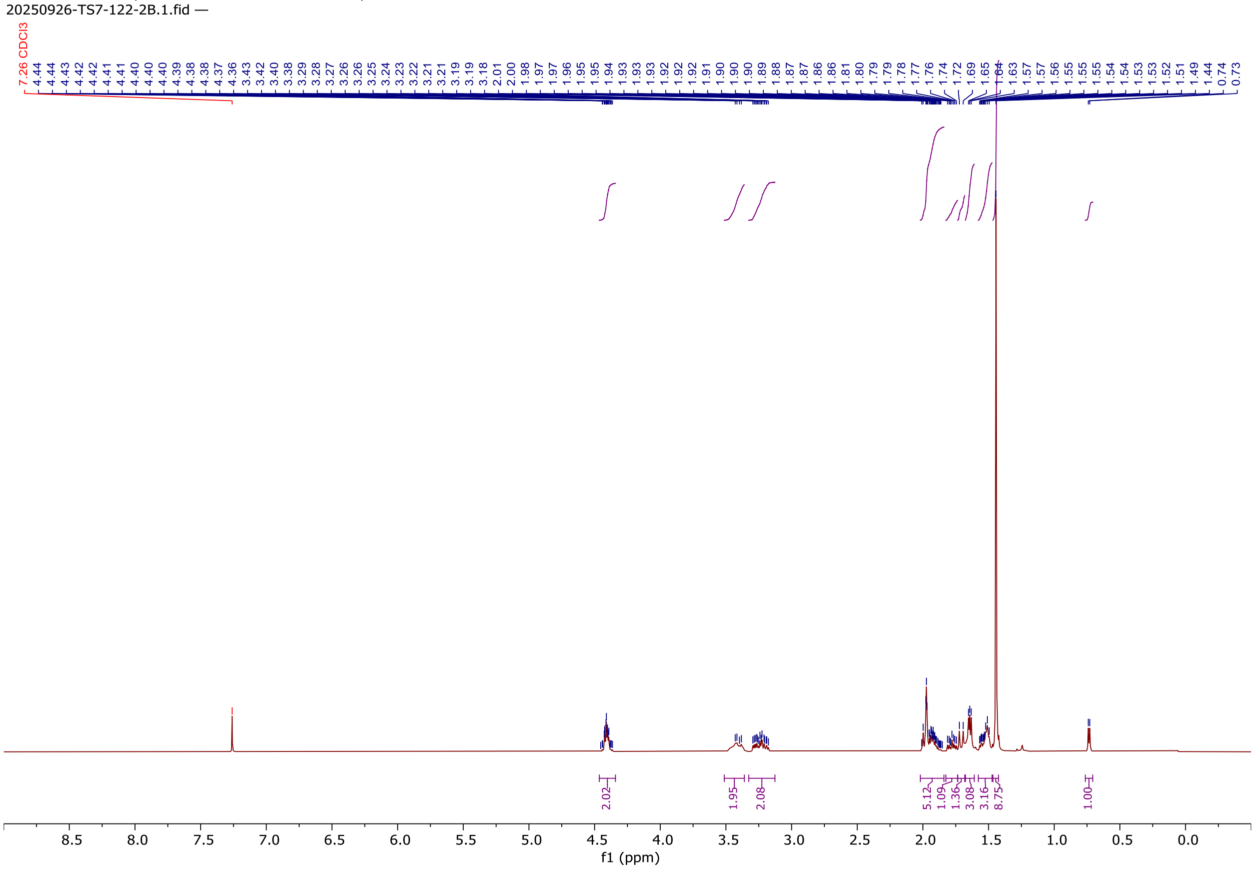

^{13}C NMR (101 MHz, $CDCl_3$)

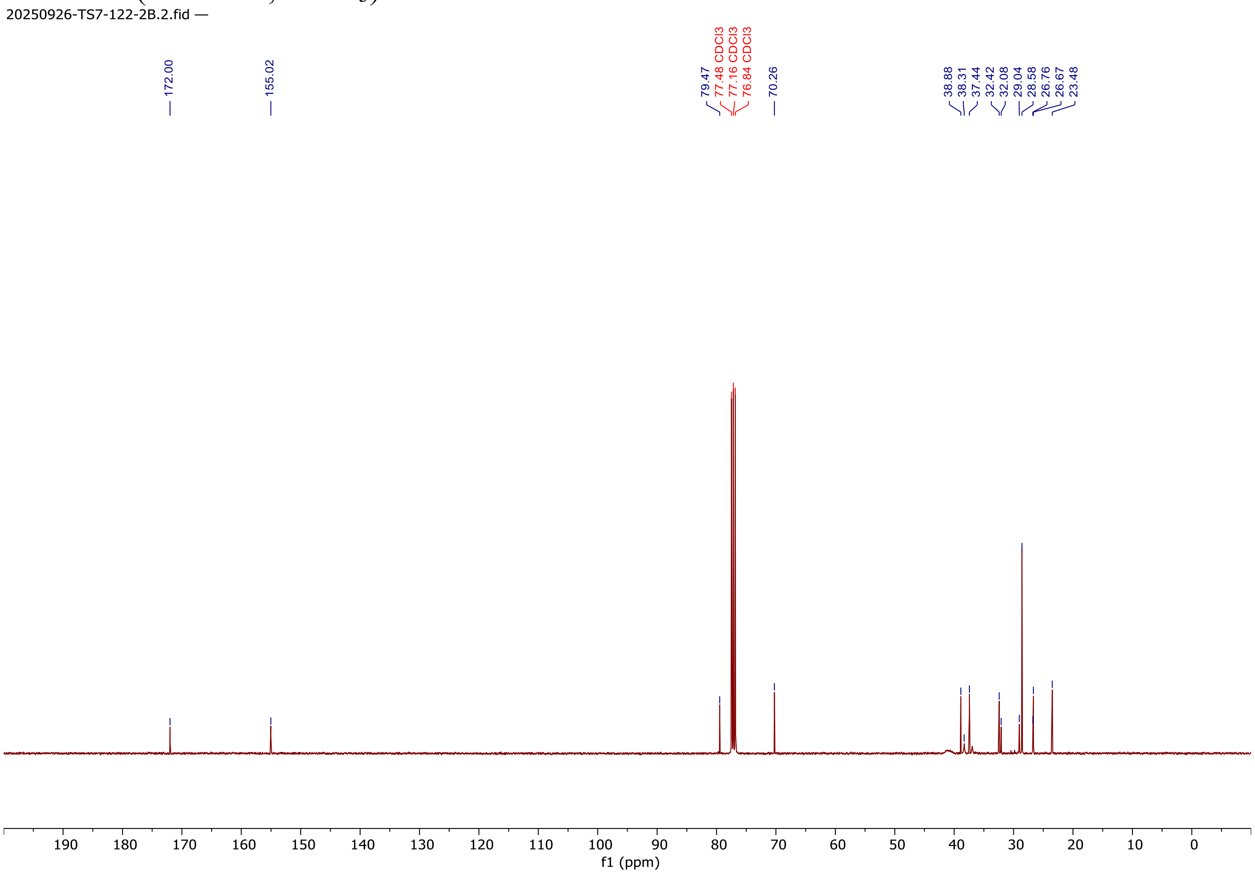

Chiral HPLC (210 nm trace):
Racemate, synthesized from $Rh_2(OAc)_4$

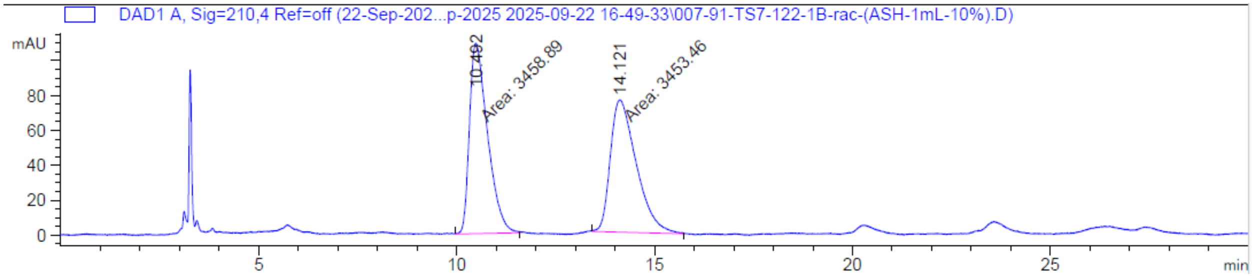

Signal 1: DAD1 A, Sig=210,4 Ref=off

Peak #	RetTime [min]	Type	Width [min]	Area [mAU*s]	Height [mAU]	Area %
1	10.492	MM	0.5284	3458.89282	109.10245	50.0393
2	14.121	MM	0.7584	3453.46069	75.88908	49.9607
Totals :				6912.35352	184.99152	

Chiral, synthesized from Rh_2(*S*-*p*-PhTPCP)$_4$, 77% *ee*

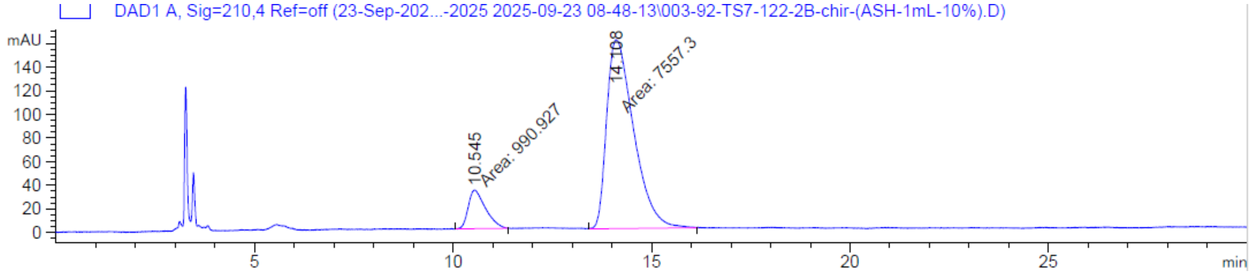

Signal 1: DAD1 A, Sig=210,4 Ref=off

Peak #	RetTime [min]	Type	Width [min]	Area [mAU*s]	Height [mAU]	Area %
1	10.545	MM	0.5023	990.92651	32.88188	11.5922
2	14.108	MM	0.7872	7557.30029	160.01320	88.4078
Totals :				8548.22681	192.89508	

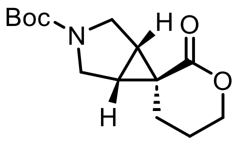

tert-Butyl (1_R_,5_S_,6_r_)-2'-oxodihydro-2'_H_,4'_H_-3-azaspiro[bicyclo[3.1.0]hexane-6,3'-pyran]-3-carboxylate (32a)

^{1}H NMR (400 MHz, $CDCl_3$)

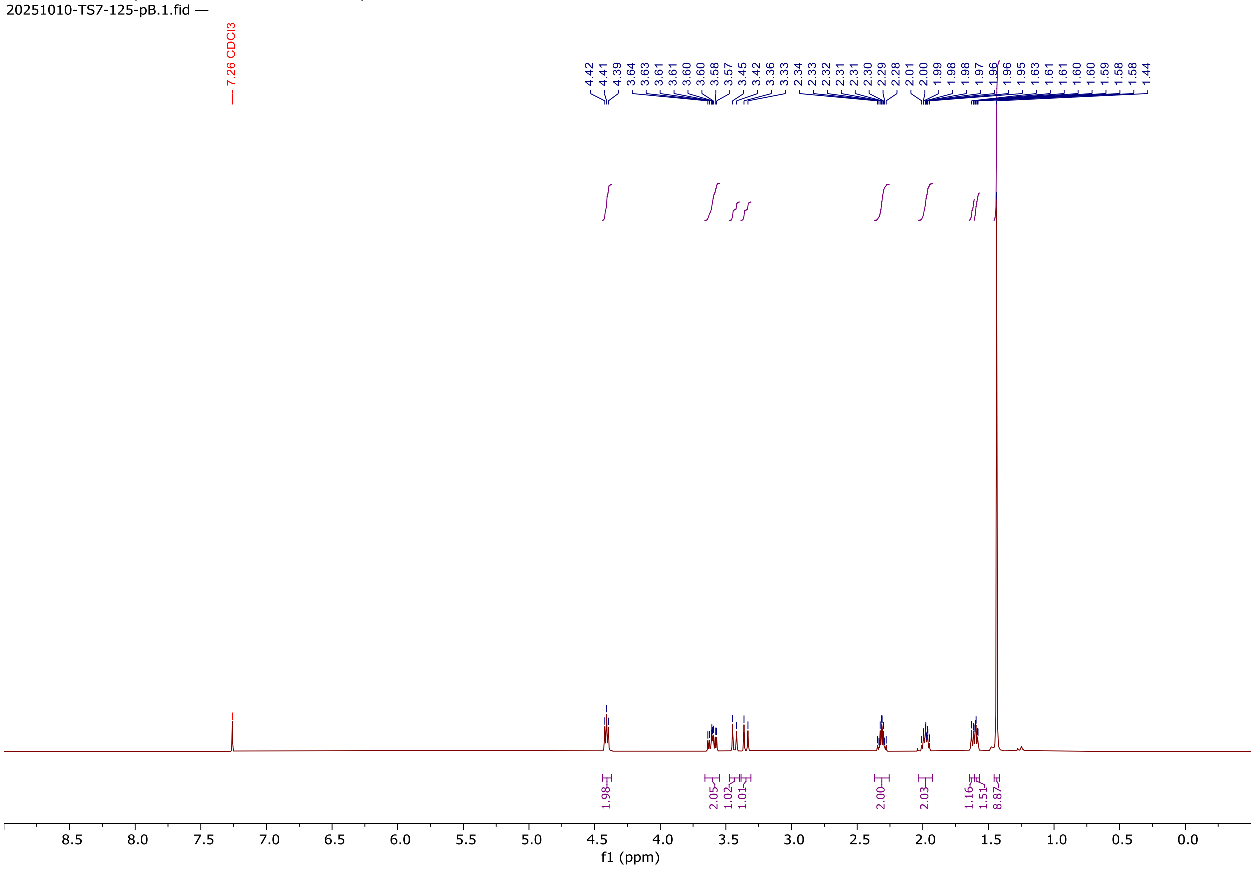

^{13}C NMR (101 MHz, $CDCl_3$)

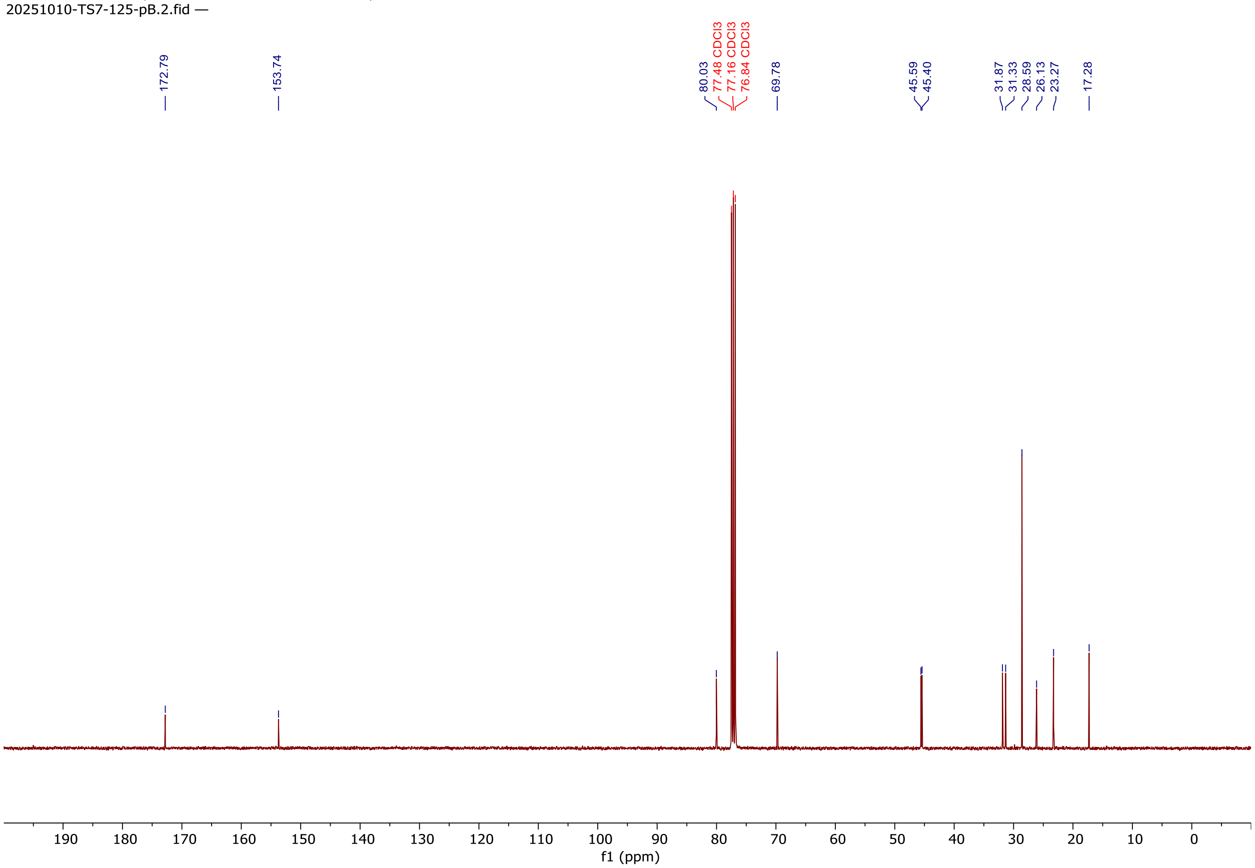

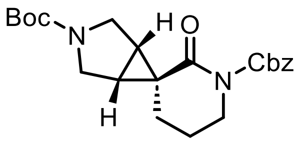

1'-Benzyl 3-(*tert*-butyl) (1*R*,5*S*,6*r*)-2'-oxo-3-azaspiro[bicyclo[3.1.0]hexane-6,3'-piperidine]-1',3-dicarboxylate (32b)

^{1}H NMR (400 MHz, $CDCl_3$)

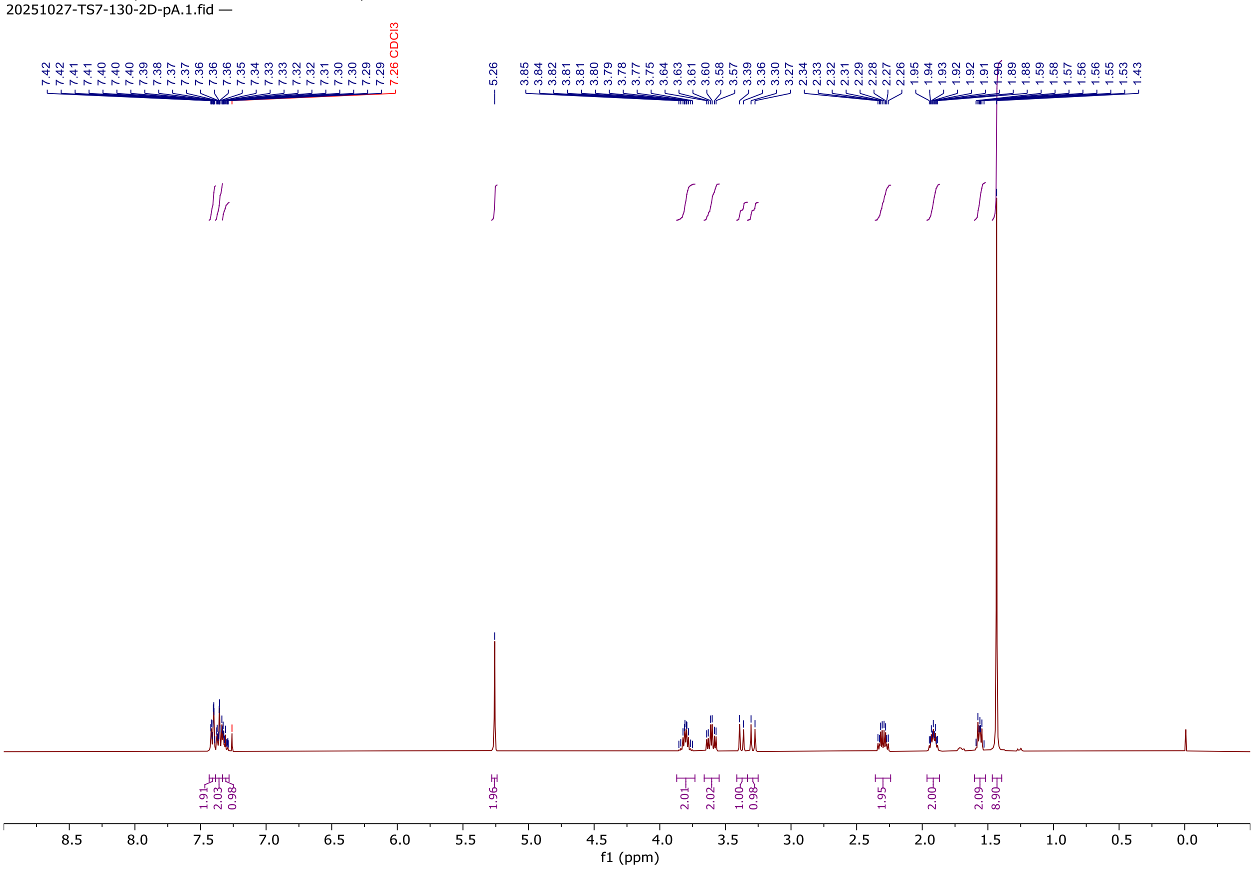

^{13}C NMR (101 MHz, $CDCl_3$)

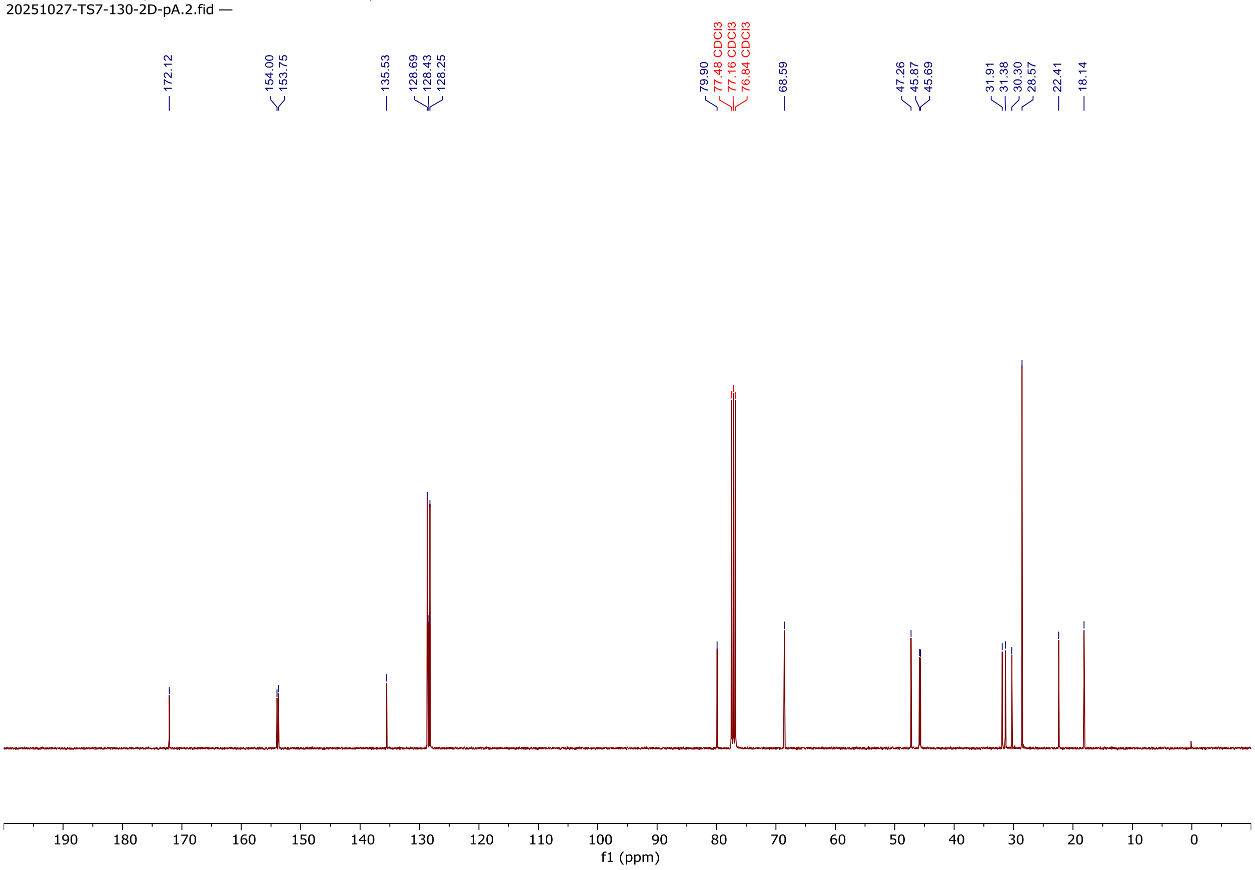

X-ray Structure of 24a

Absolute stereochemistry of compound **24a** was determined by X-ray crystallography as shown below. All other products from the cyclopropanation reaction were tentatively assigned by analogy.

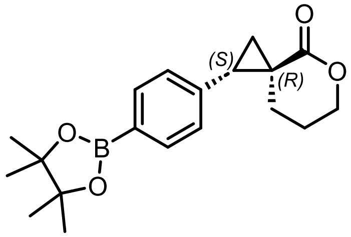

Supplement: Supplementary file 1 [file ja6c01407_si_001.pdf]
